# Posicionamento sobre a Saúde Cardiometabólica ao Longo do Ciclo de Vida da Mulher – 2025

**DOI:** 10.36660/abc.20250615

**Published:** 2025-09-17

**Authors:** Gláucia Maria Moraes de Oliveira, Maria Cristina Costa de Almeida, Cynthia Melissa Valério, Fernando Giuffrida, Larissa Neto Espíndola, Maria Cristina de Oliveira Izar, Celi Marques-Santos, Claudia Maria Vilas Freire, Carlos Japhet da Matta Albuquerque, Antonio Carlos Pallandri Chagas, Dalton Bertolim Précoma, Evandro Tinoco Mesquita, José Francisco Kerr Saraiva, Maria Elizabeth Navegantes Caetano Costa, Viviana de Mello Guzzo Lemke, Alexandre Jorge Gomes de Lucena, Andréa Araujo Brandão, Antonio Aurelio de Paiva Fagundes, Ariane Vieira Scarlatelli Macedo, Carisi Anne Polanczyk, Cristiane Bauermann Leitão, Daniel Souto Silveira, Elaine dos Reis Coutinho, Eliana Aguiar Petri Nahas, Elizabeth Regina Giunco Alexandre, Erika Maria Gonçalves Campana, Erika Olivier Vilela Bragança, Fernanda Marciano Consolim Colombo, Imara Correia de Queiroz Barbosa, Ivan Romero Rivera, Jaime Kulak, João Eduardo Nunes Salles, João Roberto de Sá, José Maria Soares, Larissa de Almeida Dourado, Lidia Zytynski Moura, Lucelia Batista Neves Cunha Magalhães, Luciano de Melo Pompei, Luiz Guilherme Passaglia, Marcelo Heitor Vieira Assad, Marcio Alexandre Hipólito Rodrigues, Maria Alayde Mendonça Rivera, Maria Antonieta Albanez Albuquerque de Medeiros Lopes, Maria Sanali Moura de Oliveira Paiva, Marildes Luiza de Castro, Milena dos Santos Barros Campos, Olga Ferreira de Souza, Orlando Otávio de Medeiros, Rafaela Andrade Penalva Freitas, Regina Coeli Marques de Carvalho, Sheyla Cristina Tonheiro Ferro da Silva, Thais de Carvalho Vieira Rodrigues, Walkiria Samuel Avila, Wellington Santana da Silva, Willyan Issamu Nazima, Lucia Helena Simões da Costa-Paiva, Maria Celeste Osorio Wender

**Affiliations:** 1 Universidade Federal do Rio de Janeiro Rio de Janeiro RJ Brasil Universidade Federal do Rio de Janeiro (UFRJ), Rio de Janeiro, RJ – Brasil; 2 Hospital João XXIII Fundação Hospitalar do Estado de Minas Gerais Belo Horizonte MG Brasil Hospital João XXIII, Fundação Hospitalar do Estado de Minas Gerais, Belo Horizonte, MG – Brasil; 3 Centro Universitário de Belo Horizonte Belo Horizonte MG Brasil Centro Universitário de Belo Horizonte (UNIBH), Belo Horizonte, MG – Brasil; 4 Instituto Estadual de Diabetes e Endocrinologia Luiz Capriglione Rio de Janeiro RJ Brasil Instituto Estadual de Diabetes e Endocrinologia Luiz Capriglione (IEDE-RJ), Rio de Janeiro, RJ – Brasil; 5 Universidade do Estado da Bahia Salvador BA Brasil Universidade do Estado da Bahia, Salvador, BA – Brasil; 6 Hospital Santa Izabel Salvador BA Brasil Hospital Santa Izabel, Salvador, BA – Brasil; 7 Hospital Municipal de Salvador Salvador BA Brasil Hospital Municipal de Salvador, Salvador, BA – Brasil; 8 Universidade Federal de São Paulo São Paulo SP Brasil Universidade Federal de São Paulo (UNIFESP), São Paulo, SP – Brasil; 9 Universidade Tiradentes Aracaju SE Brasil Universidade Tiradentes, Aracaju, SE – Brasil; 10 Hospital São Lucas Rede D’Or São Luiz Aracaju SE Brasil Hospital São Lucas, Rede D’Or São Luiz, Aracaju, SE – Brasil; 11 Universidade Federal de Minas Gerais Belo Horizonte MG Brasil Universidade Federal de Minas Gerais (UFMG), Belo Horizonte, MG –Brasil; 12 Hospital Santa Joana Recife Rede Américas Recife PE Brasil Hospital Santa Joana Recife – Rede Américas, Recife PE – Brasil; 13 Hospital Barão de Lucena – SUS/PE Recife PE Brasil Hospital Barão de Lucena – SUS/PE, Recife PE – Brasil; 14 Centro Universitário Faculdade de Medicina do ABC Santo André SP Brasil Centro Universitário Faculdade de Medicina do ABC, Santo André, SP – Brasil; 15 Universidade de São Paulo (HCFMUSP) Instituto do Coração (Incor) Hospital das Clínicas da Faculdade de Medicina São Paulo SP Brasil Instituto do Coração (Incor) do Hospital das Clínicas da Faculdade de Medicina da Universidade de São Paulo (HCFMUSP), São Paulo, SP – Brasil; 16 Sociedade Hospitalar Angelina Caron Curitiba PR Brasil Sociedade Hospitalar Angelina Caron, Curitiba, PR – Brasil; 17 Universidade Federal Fluminense Rio de Janeiro RJ Brasil Universidade Federal Fluminense (UFF), Rio de Janeiro, RJ – Brasil; 18 Pontifícia Universidade Católica de Campinas Campinas SP Brasil Pontifícia Universidade Católica de Campinas, Campinas, SP – Brasil; 19 Centro Universitário do Estado Pará (CESUPA) Belém PA Brasil Centro Universitário do Estado Pará (CESUPA), Belém PA – Brasil; 20 Cardiocare-Clínica Cardiológica Ltda Curitiba PR Brasil Cardiocare-Clínica Cardiológica Ltda, Curitiba, PR – Brasil; 21 Hospital Agamenon Magalhães Recife PE Brasil Hospital Agamenon Magalhães, Recife, PE – Brasil; 22 Universidade do Estado do Rio de Janeiro Rio de Janeiro RJ Brasil Universidade do Estado do Rio de Janeiro (UERJ), Rio de Janeiro, RJ – Brasil; 23 Instituto D’Or de Pesquisa e Ensino Brasília DF Brasil Instituto D’Or de Pesquisa e Ensino (IDOR), Brasília, DF – Brasil; 24 Universidade de Brasília Brasília DF Brasil Universidade de Brasília (UNB), Brasília, DF – Brasil; 25 Hospital DFStar Brasília DF Brasil Hospital DFStar, Brasília, DF – Brasil; 26 Santa Casa de Misericórdia de São Paulo São Paulo SP Brasil Santa Casa de Misericórdia de São Paulo, São Paulo, SP – Brasil; 27 Universidade Federal do Rio Grande do Sul Hospital de Clínicas Porto Alegre RS Brasil Hospital de Clínicas da Universidade Federal do Rio Grande do Sul (UFRS), Porto Alegre, RS – Brasil; 28 Universidade Federal do Rio Grande do Sul Porto Alegre RS Brasil Universidade Federal do Rio Grande do Sul, Porto Alegre, RS – Brasil; 29 Instituto de Medicina Vascular Hospital Mãe de Deus Porto Alegre RS Brasil Instituto de Medicina Vascular – Hospital Mãe de Deus, Porto Alegre, RS – Brasil; 30 Hospital PUC Campinas Campinas SP Brasil Hospital PUC Campinas, Campinas, SP – Brasil; 31 Universidade Estadual Paulista Julio Mesquita Filho Faculdade de Medicina de Botucatu Botucatu SP Brasil Faculdade de Medicina de Botucatu, Universidade Estadual Paulista Julio Mesquita Filho (UNESP), Botucatu, SP – Brasil; 32 Hospital do Coração São Paulo SP Brasil Hospital do Coração (HCor), São Paulo, SP – Brasil; 33 RitmoCheck São José dos Campos SP Brasil RitmoCheck, São José dos Campos, SP – Brasil; 34 Universidade Federal de Campina Grande Campina Grande PB Brasil Universidade Federal de Campina Grande, Campina Grande, PB – Brasil; 35 Universidade Federal de Alagoas Maceió AL Brasil Universidade Federal de Alagoas, Maceió, AL – Brasil; 36 Universidade Federal do Paraná Curitiba PR Brasil Universidade Federal do Paraná, Curitiba, PR – Brasil; 37 Médicas da Santa Casa de São Paulo Faculdade de Ciências São Paulo SP Brasil Faculdade de Ciências Médicas da Santa Casa de São Paulo, São Paulo, SP – Brasil; 38 Universidade de São Paulo Hospital das Clínicas Faculdade de Medicina São Paulo SP Brasil Faculdade de Medicina da Universidade de São Paulo, Hospital das Clínicas, São Paulo, SP – Brasil; 39 Pontifícia Universidade Católica do Paraná Curitiba PR Brasil Pontifícia Universidade Católica do Paraná, Curitiba, PR – Brasil; 40 Faculdade de Medicina da Unesulbahia Salvador BA Brasil Faculdade de Medicina da Unesulbahia, Salvador, BA – Brasil; 41 Universidade Federal de Minas Gerais Hospital das Clínicas Belo Horizonte MG Brasil Hospital das Clínicas da Universidade Federal de Minas Gerais (UFMG), Belo Horizonte, MG – Brasil; 42 Instituto Nacional de Cardiologia Rio de Janeiro RJ Brasil Instituto Nacional de Cardiologia, Rio de Janeiro, RJ – Brasil; 43 Universidade Federal de Minas Gerais Belo Horizonte MG Brasil Universidade Federal de Minas Gerais (UFMG), Belo Horizonte, MG – Brasil; 44 Real Hospital Português Recife PE Brasil Real Hospital Português, Recife, PE – Brasil; 45 Hospital São Marcos Recife PE Brasil Hospital São Marcos, Recife, PE – Brasil; 46 Cardiointerve Natal RN Brasil Cardiointerve, Natal, RN – Brasil; 47 Universidade Federal de Sergipe Hospital Universitário Aracaju SE Brasil Hospital Universitário da Universidade Federal de Sergipe, Aracaju, SE – Brasil; 48 Ministério da Saúde Brasília DF Brasil Ministério da Saúde, Brasília, DF – Brasil; 49 Instituto Dante Pazzanese de Cardiologia São Paulo SP Brasil Instituto Dante Pazzanese de Cardiologia, São Paulo, SP – Brasil; 50 Hospital Geral de Fortaleza Fortaleza CE Brasil Hospital Geral de Fortaleza, Fortaleza, CE – Brasil; 51 Secretaria da Saúde do Ceará Fortaleza CE Brasil Secretaria da Saúde do Ceará, Fortaleza, CE – Brasil; 52 CEMISE Oncoclínicas Aracaju SE Brasil CEMISE Oncoclínicas, Aracaju, SE – Brasil; 53 Prefeitura Municipal de Aracaju Aracaju SE Brasil Prefeitura Municipal de Aracaju, Aracaju, SE – Brasil; 54 Universidade Federal de Sergipe Aracaju SE Brasil Universidade Federal de Sergipe (UFS), Aracaju, SE – Brasil; 55 Universidade Federal do Maranhão São Luís MA Brasil Universidade Federal do Maranhão (UFMA), São Luís, MA – Brasil; 56 Hospital Evangélico de Londrina Londrina PR Brasil Hospital Evangélico de Londrina, Londrina, PR – Brasil; 57 Universidade Estadual de Campinas Campinas SP Brasil Universidade Estadual de Campinas (UNICAMP), Campinas, SP – Brasil

**Table:** 

Posicionamento sobre a Saúde Cardiometabólica ao Longo do Ciclo de Vida da Mulher – 2025	
O relatório abaixo lista as declarações de interesse conforme relatadas à SBC pelos especialistas durante o período de desenvolvimento deste posicionamento, 2024/2025.	
Especialista	Tipo de relacionamento com a indústria
Alexandre Jorge Gomes de Lucena	Nada a ser declarado
Antonio Aurelio de Paiva Fagundes Junior	Declaração financeira A - Pagamento de qualquer espécie e desde que economicamente apreciáveis, feitos a (i) você, (ii) ao seu cônjuge/ companheiro ou a qualquer outro membro que resida com você, (iii) a qualquer pessoa jurídica em que qualquer destes seja controlador, sócio, acionista ou participante, de forma direta ou indireta, recebimento por palestras, aulas, atuação como proctor de treinamentos, remunerações, honorários pagos por participações em conselhos consultivos, de investigadores, ou outros comitês, etc. Provenientes da indústria farmacêutica, de órteses, próteses, equipamentos e implantes, brasileiras ou estrangeiras: - AstraZeneca: Lokelma; Boehringer-Ingelheim: Metalyse; Novartis: Sybrava; Abbot: Ritmonorm; Mundipharma: Rezzayo; Libbs: Plenance. B - Financiamento de pesquisas sob sua responsabilidade direta/pessoal (direcionado ao departamento ou instituição) provenientes da indústria farmacêutica, de órteses, próteses, equipamentos e implantes, brasileiras ou estrangeiras: - Novartis: insuficiência renal; Eli lilly: Lipoproteína A.
Antonio Carlos Palandri Chagas	Declaração financeira A - Pagamento de qualquer espécie e desde que economicamente apreciáveis, feitos a (i) você, (ii) ao seu cônjuge/ companheiro ou a qualquer outro membro que resida com você, (iii) a qualquer pessoa jurídica em que qualquer destes seja controlador, sócio, acionista ou participante, de forma direta ou indireta, recebimento por palestras, aulas, atuação como proctor de treinamentos, remunerações, honorários pagos por participações em conselhos consultivos, de investigadores, ou outros comitês, etc. Provenientes da indústria farmacêutica, de órteses, próteses, equipamentos e implantes, brasileiras ou estrangeiras: - Instituto de Vita.
Ariane Vieira Scarlatelli Macedo	Declaração financeira A - Pagamento de qualquer espécie e desde que economicamente apreciáveis, feitos a (i) você, (ii) ao seu cônjuge/ companheiro ou a qualquer outro membro que resida com você, (iii) a qualquer pessoa jurídica em que qualquer destes seja controlador, sócio, acionista ou participante, de forma direta ou indireta, recebimento por palestras, aulas, atuação como proctor de treinamentos, remunerações, honorários pagos por participações em conselhos consultivos, de investigadores, ou outros comitês, etc. Provenientes da indústria farmacêutica, de órteses, próteses, equipamentos e implantes, brasileiras ou estrangeiras: - Bayer: anticoagulação e insuficiência cardíaca; Pfizer: anticoagulação e amiloidose; Jannsen: leucemia. Outros relacionamentos Financiamento de atividades de educação médica continuada, incluindo viagens, hospedagens e inscrições para congressos e cursos, provenientes da indústria farmacêutica, de órteses, próteses, equipamentos e implantes, brasileiras ou estrangeiras: - Bayer: insuficiência cardíaca.
Carisi Anne Polanczyk	Nada a ser declarado
Carlos Japhet da Matta Albuquerque	Nada a ser declarado
Celi Marques Santos	Nada a ser declarado
Claudia Maria Vilas Freire	Nada a ser declarado
Cristiane Bauermann Leitão	Declaração financeira A - Pagamento de qualquer espécie e desde que economicamente apreciáveis, feitos a (i) você, (ii) ao seu cônjuge/ companheiro ou a qualquer outro membro que resida com você, (iii) a qualquer pessoa jurídica em que qualquer destes seja controlador, sócio, acionista ou participante, de forma direta ou indireta, recebimento por palestras, aulas, atuação como proctor de treinamentos, remunerações, honorários pagos por participações em conselhos consultivos, de investigadores, ou outros comitês, etc. Provenientes da indústria farmacêutica, de órteses, próteses, equipamentos e implantes, brasileiras ou estrangeiras: - Novo Nordisk. Outros relacionamentos Financiamento de atividades de educação médica continuada, incluindo viagens, hospedagens e inscrições para congressos e cursos, provenientes da indústria farmacêutica, de órteses, próteses, equipamentos e implantes, brasileiras ou estrangeiras: - Lilly.
Cynthia Melissa Valerio	Declaração financeira A - Pagamento de qualquer espécie e desde que economicamente apreciáveis, feitos a (i) você, (ii) ao seu cônjuge/ companheiro ou a qualquer outro membro que resida com você, (iii) a qualquer pessoa jurídica em que qualquer destes seja controlador, sócio, acionista ou participante, de forma direta ou indireta, recebimento por palestras, aulas, atuação como proctor de treinamentos, remunerações, honorários pagos por participações em conselhos consultivos, de investigadores, ou outros comitês, etc. Provenientes da indústria farmacêutica, de órteses, próteses, equipamentos e implantes, brasileiras ou estrangeiras: - Novo Nordisk: Diabetes e Obesidade; Boehringer Ingelheim: Diabetes; Eli Lilly: Diabetes e Obesidade; AstraZeneca: Diabetes; EMS: Diabetes e Obesidade. B - Financiamento de pesquisas sob sua responsabilidade direta/pessoal (direcionado ao departamento ou instituição) provenientes da indústria farmacêutica, de órteses, próteses, equipamentos e implantes, brasileiras ou estrangeiras: - Chiesi: metreleptina. Outros relacionamentos Financiamento de atividades de educação médica continuada, incluindo viagens, hospedagens e inscrições para congressos e cursos, provenientes da indústria farmacêutica, de órteses, próteses, equipamentos e implantes, brasileiras ou estrangeiras: - Chiesi: metreleptina.
Daniel Souto Silveira	Nada a ser declarado
Dalton Bertolim Precoma	Declaração financeira B - Financiamento de pesquisas sob sua responsabilidade direta/pessoal (direcionado ao departamento ou instituição) provenientes da indústria farmacêutica, de órteses, próteses, equipamentos e implantes, brasileiras ou estrangeiras: - Janssen: anticoagulação; Astrazeneca: dislipidemia e inibidores da aldosterona; Novonordisk: insuficiência cardíaca; Arrowhead: dislipidemia; Vertrix: antiagregação plaquetária. Outros relacionamentos Financiamento de atividades de educação médica continuada, incluindo viagens, hospedagens e inscrições para congressos e cursos, provenientes da indústria farmacêutica, de órteses, próteses, equipamentos e implantes, brasileiras ou estrangeiras: - Daiichi Sankyo: anticoagulação; GSK: vacinas; Astrazeneca: cardiometabolismo.
Elaine dos Reis Coutinho	Declaração financeira A - Pagamento de qualquer espécie e desde que economicamente apreciáveis, feitos a (i) você, (ii) ao seu cônjuge/ companheiro ou a qualquer outro membro que resida com você, (iii) a qualquer pessoa jurídica em que qualquer destes seja controlador, sócio, acionista ou participante, de forma direta ou indireta, recebimento por palestras, aulas, atuação como proctor de treinamentos, remunerações, honorários pagos por participações em conselhos consultivos, de investigadores, ou outros comitês, etc. Provenientes da indústria farmacêutica, de órteses, próteses, equipamentos e implantes, brasileiras ou estrangeiras: - Novartis: Sybrava; Biolab: Livalo e Repatha; Daichii sankyo: Nustendi. Outros relacionamentos Financiamento de atividades de educação médica continuada, incluindo viagens, hospedagens e inscrições para congressos e cursos, provenientes da indústria farmacêutica, de órteses, próteses, equipamentos e implantes, brasileiras ou estrangeiras: - Novartis, Daichii, Biolab.
Eliana Aguiar Petri Nahas	Declaração financeira A - Pagamento de qualquer espécie e desde que economicamente apreciáveis, feitos a (i) você, (ii) ao seu cônjuge/ companheiro ou a qualquer outro membro que resida com você, (iii) a qualquer pessoa jurídica em que qualquer destes seja controlador, sócio, acionista ou participante, de forma direta ou indireta, recebimento por palestras, aulas, atuação como proctor de treinamentos, remunerações, honorários pagos por participações em conselhos consultivos, de investigadores, ou outros comitês, etc. Provenientes da indústria farmacêutica, de órteses, próteses, equipamentos e implantes, brasileiras ou estrangeiras: - Libbs: Yumi, Iziz, Libiam, Natifa; Theramex: Estreva Gel, linha Systen; Exeltis: Gynpro; Gedeon: Lenzetto; Besins: Oestrogel; Astellas: Veoza.
Elizabeth Regina Giunco Alexandre	Declaração financeira A - Pagamento de qualquer espécie e desde que economicamente apreciáveis, feitos a (i) você, (ii) ao seu cônjuge/ companheiro ou a qualquer outro membro que resida com você, (iii) a qualquer pessoa jurídica em que qualquer destes seja controlador, sócio, acionista ou participante, de forma direta ou indireta, recebimento por palestras, aulas, atuação como proctor de treinamentos, remunerações, honorários pagos por participações em conselhos consultivos, de investigadores, ou outros comitês, etc. Provenientes da indústria farmacêutica, de órteses, próteses, equipamentos e implantes, brasileiras ou estrangeiras: - Servier: Vastarel MR; Lilly: Mounjaro; Libbs: Ebatz e Stanglitz, NovoNordisk: Ozempic; Astra Zeneca: Breztri; Boehringer-Ingelhein: Glyxambi; Mantecorpp: Nesina/Addera. Outros relacionamentos Financiamento de atividades de educação médica continuada, incluindo viagens, hospedagens e inscrições para congressos e cursos, provenientes da indústria farmacêutica, de órteses, próteses, equipamentos e implantes, brasileiras ou estrangeiras: - Lilly.
Erika Maria Gonçalves Campana	Declaração financeira A - Pagamento de qualquer espécie e desde que economicamente apreciáveis, feitos a (i) você, (ii) ao seu cônjuge/ companheiro ou a qualquer outro membro que resida com você, (iii) a qualquer pessoa jurídica em que qualquer destes seja controlador, sócio, acionista ou participante, de forma direta ou indireta, recebimento por palestras, aulas, atuação como proctor de treinamentos, remunerações, honorários pagos por participações em conselhos consultivos, de investigadores, ou outros comitês, etc. Provenientes da indústria farmacêutica, de órteses, próteses, equipamentos e implantes, brasileiras ou estrangeiras: - Servier, Brace Pharma, Biolab, Momenta. Outros relacionamentos Financiamento de atividades de educação médica continuada, incluindo viagens, hospedagens e inscrições para congressos e cursos, provenientes da indústria farmacêutica, de órteses, próteses, equipamentos e implantes, brasileiras ou estrangeiras: - Servier, Biolab: hipertensão.
Érika Olivier Vilela Bragança	Declaração financeira A - Pagamento de qualquer espécie e desde que economicamente apreciáveis, feitos a (i) você, (ii) ao seu cônjuge/ companheiro ou a qualquer outro membro que resida com você, (iii) a qualquer pessoa jurídica em que qualquer destes seja controlador, sócio, acionista ou participante, de forma direta ou indireta, recebimento por palestras, aulas, atuação como proctor de treinamentos, remunerações, honorários pagos por participações em conselhos consultivos, de investigadores, ou outros comitês, etc. Provenientes da indústria farmacêutica, de órteses, próteses, equipamentos e implantes, brasileiras ou estrangeiras: - Biolab: Dozoito. Outros relacionamentos Financiamento de atividades de educação médica continuada, incluindo viagens, hospedagens e inscrições para congressos e cursos, provenientes da indústria farmacêutica, de órteses, próteses, equipamentos e implantes, brasileiras ou estrangeiras: - Merck.
Evandro Tinoco Mesquita	Declaração financeira A - Pagamento de qualquer espécie e desde que economicamente apreciáveis, feitos a (i) você, (ii) ao seu cônjuge/ companheiro ou a qualquer outro membro que resida com você, (iii) a qualquer pessoa jurídica em que qualquer destes seja controlador, sócio, acionista ou participante, de forma direta ou indireta, recebimento por palestras, aulas, atuação como proctor de treinamentos, remunerações, honorários pagos por participações em conselhos consultivos, de investigadores, ou outros comitês, etc. Provenientes da indústria farmacêutica, de órteses, próteses, equipamentos e implantes, brasileiras ou estrangeiras: - Ache: material educacional e aulas Astra. Outros relacionamentos Financiamento de atividades de educação médica continuada, incluindo viagens, hospedagens e inscrições para congressos e cursos, provenientes da indústria farmacêutica, de órteses, próteses, equipamentos e implantes, brasileiras ou estrangeiras: - Pfizer: amiloidose.
Fernanda Marciano Consolim Colombo	Declaração financeira A - Pagamento de qualquer espécie e desde que economicamente apreciáveis, feitos a (i) você, (ii) ao seu cônjuge/ companheiro ou a qualquer outro membro que resida com você, (iii) a qualquer pessoa jurídica em que qualquer destes seja controlador, sócio, acionista ou participante, de forma direta ou indireta, recebimento por palestras, aulas, atuação como proctor de treinamentos, remunerações, honorários pagos por participações em conselhos consultivos, de investigadores, ou outros comitês, etc. Provenientes da indústria farmacêutica, de órteses, próteses, equipamentos e implantes, brasileiras ou estrangeiras: - Daiichi Sankyo; Merck; Servier; AstraZeneca. Outros relacionamentos Financiamento de atividades de educação médica continuada, incluindo viagens, hospedagens e inscrições para congressos e cursos, provenientes da indústria farmacêutica, de órteses, próteses, equipamentos e implantes, brasileiras ou estrangeiras: - Daiichi Sankyo; Servier.
Fernando M. A. Giuffrida	Nada a ser declarado
Gláucia Maria Moraes de Oliveira	Nada a ser declarado
Imara Correia de Queiroz Barbosa	Declaração financeira A - Pagamento de qualquer espécie e desde que economicamente apreciáveis, feitos a (i) você, (ii) ao seu cônjuge/ companheiro ou a qualquer outro membro que resida com você, (iii) a qualquer pessoa jurídica em que qualquer destes seja controlador, sócio, acionista ou participante, de forma direta ou indireta, recebimento por palestras, aulas, atuação como proctor de treinamentos, remunerações, honorários pagos por participações em conselhos consultivos, de investigadores, ou outros comitês, etc. Provenientes da indústria farmacêutica, de órteses, próteses, equipamentos e implantes, brasileiras ou estrangeiras: - AstraZeneca: insuficiência cardíaca (Forxiga, Selozok); Servier: hipertensão (Triplixam).
Ivan Romero Rivera	Nada a ser declarado
Jaime Kulak Junior	Declaração financeira A - Pagamento de qualquer espécie e desde que economicamente apreciáveis, feitos a (i) você, (ii) ao seu cônjuge/ companheiro ou a qualquer outro membro que resida com você, (iii) a qualquer pessoa jurídica em que qualquer destes seja controlador, sócio, acionista ou participante, de forma direta ou indireta, recebimento por palestras, aulas, atuação como proctor de treinamentos, remunerações, honorários pagos por participações em conselhos consultivos, de investigadores, ou outros comitês, etc. Provenientes da indústria farmacêutica, de órteses, próteses, equipamentos e implantes, brasileiras ou estrangeiras: - Bayer: Mirena; Besins: Vagifem; Biolab: Qlaira; Theramex: Systen, Estreva; Merck: Glifage XR; Astellas: Fezolinetanto. Outros relacionamentos Financiamento de atividades de educação médica continuada, incluindo viagens, hospedagens e inscrições para congressos e cursos, provenientes da indústria farmacêutica, de órteses, próteses, equipamentos e implantes, brasileiras ou estrangeiras: - Astellas: Congresso da International Menopause Society; Besins: Congresso da FIGO e Congresso da International Menopause Society.
João Eduardo Nunes Salles	Nada a ser declarado
João Roberto de Sá	Nada a ser declarado
José Francisco Kerr Saraiva	Declaração financeira C - Financiamento de pesquisa (pessoal), cujas receitas tenham sido provenientes da indústria farmacêutica, de órteses, próteses, equipamentos e implantes, brasileiras ou estrangeiras: - Bayer: finerinona; Novo Nordisk: semaglutida; AstraZeneca: ciclosilicato de Zirconio, dapagliflozina; Amgen: evolocumabe; Boehringer Ingelheimer: empagliflozina; Lilly: tirzepatida, viatris atorvastatina; Daichii Sankyo: ácido bempedoico/Edoxabana; Mantecorp: rosuvastatina. Outros relacionamentos Financiamento de atividades de educação médica continuada, incluindo viagens, hospedagens e inscrições para congressos e cursos, provenientes da indústria farmacêutica, de órteses, próteses, equipamentos e implantes, brasileiras ou estrangeiras: - Bayer: finerinona; Novo Nordisk: Semaglutida; AstraZeneca: ciclosilicato de Zirconio, dapagliflozina; Amgen: evolocumabe; Boehringer Ingelheimer: empagliflozina; Lilly: tirzepatida, viatris atorvastatina; Daichii Sankyo: ácido bempedoico/edoxabana.
José Maria Soares Júnior	Declaração financeira A - Pagamento de qualquer espécie e desde que economicamente apreciáveis, feitos a (i) você, (ii) ao seu cônjuge/ companheiro ou a qualquer outro membro que resida com você, (iii) a qualquer pessoa jurídica em que qualquer destes seja controlador, sócio, acionista ou participante, de forma direta ou indireta, recebimento por palestras, aulas, atuação como proctor de treinamentos, remunerações, honorários pagos por participações em conselhos consultivos, de investigadores, ou outros comitês, etc. Provenientes da indústria farmacêutica, de órteses, próteses, equipamentos e implantes, brasileiras ou estrangeiras: - Pfizer: Abrysvo; Libbs: Zaila.
Larissa de Almeida Dourado	Outros relacionamentos Financiamento de atividades de educação médica continuada, incluindo viagens, hospedagens e inscrições para congressos e cursos, provenientes da indústria farmacêutica, de órteses, próteses, equipamentos e implantes, brasileiras ou estrangeiras: - EMS: Xakilis; Novartis: Sybrava.
Larissa Neto Espíndola Macedo	Declaração financeira A - Pagamento de qualquer espécie e desde que economicamente apreciáveis, feitos a (i) você, (ii) ao seu cônjuge/ companheiro ou a qualquer outro membro que resida com você, (iii) a qualquer pessoa jurídica em que qualquer destes seja controlador, sócio, acionista ou participante, de forma direta ou indireta, recebimento por palestras, aulas, atuação como proctor de treinamentos, remunerações, honorários pagos por participações em conselhos consultivos, de investigadores, ou outros comitês, etc. Provenientes da indústria farmacêutica, de órteses, próteses, equipamentos e implantes, brasileiras ou estrangeiras: - Servier: Trimetazidina.
Lidia Zytynski Moura	Declaração financeira A - Pagamento de qualquer espécie e desde que economicamente apreciáveis, feitos a (i) você, (ii) ao seu cônjuge/ companheiro ou a qualquer outro membro que resida com você, (iii) a qualquer pessoa jurídica em que qualquer destes seja controlador, sócio, acionista ou participante, de forma direta ou indireta, recebimento por palestras, aulas, atuação como proctor de treinamentos, remunerações, honorários pagos por participações em conselhos consultivos, de investigadores, ou outros comitês, etc. Provenientes da indústria farmacêutica, de órteses, próteses, equipamentos e implantes, brasileiras ou estrangeiras: - Bayer, Merck, Novartis, Novo Nordisk, Lilly, Viatris. Outros relacionamentos Participação societária de qualquer natureza e qualquer valor economicamente apreciável de empresas na área de saúde, de ensino ou em empresas concorrentes ou fornecedoras da SBC: - Novo Nordisk, Astra.
Lucelia Batista Neves Cunha Magalhães	Nada a ser declarado
Lucia Helena Simões da Costa Paiva	Declaração financeira A - Pagamento de qualquer espécie e desde que economicamente apreciáveis, feitos a (i) você, (ii) ao seu cônjuge/ companheiro ou a qualquer outro membro que resida com você, (iii) a qualquer pessoa jurídica em que qualquer destes seja controlador, sócio, acionista ou participante, de forma direta ou indireta, recebimento por palestras, aulas, atuação como proctor de treinamentos, remunerações, honorários pagos por participações em conselhos consultivos, de investigadores, ou outros comitês, etc. Provenientes da indústria farmacêutica, de órteses, próteses, equipamentos e implantes, brasileiras ou estrangeiras: - BAYER: diu mirena; Astellas Fezolinetanto; Theramex Systen e Estreva Gel, Besisn Vagifem. Outros relacionamentos Financiamento de atividades de educação médica continuada, incluindo viagens, hospedagens e inscrições para congressos e cursos, provenientes da indústria farmacêutica, de órteses, próteses, equipamentos e implantes, brasileiras ou estrangeiras: - Astellas; Congresso da International Menopaus Society 2024.
Luciano de Melo Pompei	Declaração financeira A - Pagamento de qualquer espécie e desde que economicamente apreciáveis, feitos a (i) você, (ii) ao seu cônjuge/ companheiro ou a qualquer outro membro que resida com você, (iii) a qualquer pessoa jurídica em que qualquer destes seja controlador, sócio, acionista ou participante, de forma direta ou indireta, recebimento por palestras, aulas, atuação como proctor de treinamentos, remunerações, honorários pagos por participações em conselhos consultivos, de investigadores, ou outros comitês, etc. Provenientes da indústria farmacêutica, de órteses, próteses, equipamentos e implantes, brasileiras ou estrangeiras: - Abbott, Aché, Astellas, Bayer, Besins, Biolab, Mantecorp, Libbs, Theramex. Outros relacionamentos Financiamento de atividades de educação médica continuada, incluindo viagens, hospedagens e inscrições para congressos e cursos, provenientes da indústria farmacêutica, de órteses, próteses, equipamentos e implantes, brasileiras ou estrangeiras: - Besins.
Luiz Guilherme Passaglia	Declaração financeira A - Pagamento de qualquer espécie e desde que economicamente apreciáveis, feitos a (i) você, (ii) ao seu cônjuge/ companheiro ou a qualquer outro membro que resida com você, (iii) a qualquer pessoa jurídica em que qualquer destes seja controlador, sócio, acionista ou participante, de forma direta ou indireta, recebimento por palestras, aulas, atuação como proctor de treinamentos, remunerações, honorários pagos por participações em conselhos consultivos, de investigadores, ou outros comitês, etc. Provenientes da indústria farmacêutica, de órteses, próteses, equipamentos e implantes, brasileiras ou estrangeiras: - Conselho Médico Consultivo da DASA em Minas Gerais. Outros relacionamentos Participação em órgãos governamentais de regulação, ou de defesa de direitos na área de cardiologia: - Membro do Comitê de Cardiologia do CRM MG e membro da Comissão Municipal de Cardiologia da Secretaria Municipal de Saúde de BH.
Marcelo Heitor Vieira Assad	Declaração financeira A - Pagamento de qualquer espécie e desde que economicamente apreciáveis, feitos a (i) você, (ii) ao seu cônjuge/ companheiro ou a qualquer outro membro que resida com você, (iii) a qualquer pessoa jurídica em que qualquer destes seja controlador, sócio, acionista ou participante, de forma direta ou indireta, recebimento por palestras, aulas, atuação como proctor de treinamentos, remunerações, honorários pagos por participações em conselhos consultivos, de investigadores, ou outros comitês, etc. Provenientes da indústria farmacêutica, de órteses, próteses, equipamentos e implantes, brasileiras ou estrangeiras: - AstraZeneca: Forxiga; BAYER: Firialta; Biolab: Repatha; Boerhringer Ingelheim: Glyxambi; Daiichy Sankyo: Benicar, Nustendi; EMS: Bramicar; GSK: Shingrix; Libbs: Stanglit; Lilly: Mounjaro; Novo Nordisk: Wegovy e Wegov: Rybelusus e Ozempic; Novartis: Sybrava; Pfizer: Prevenar 20; Viatris: Lipitor, Inspra. B - Financiamento de pesquisas sob sua responsabilidade direta/pessoal (direcionado ao departamento ou instituição) provenientes da indústria farmacêutica, de órteses, próteses, equipamentos e implantes, brasileiras ou estrangeiras: - AMGEN: Olpasirana. Outros relacionamentos Financiamento de atividades de educação médica continuada, incluindo viagens, hospedagens e inscrições para congressos e cursos, provenientes da indústria farmacêutica, de órteses, próteses, equipamentos e implantes, brasileiras ou estrangeiras: - Bayer: Firialta; Daiichi Sankyo: Benicar; Novo Nordisk: Wegovy.
Marcio Alexandre Hipólito Rodrigues	Declaração financeira B - Financiamento de pesquisas sob sua responsabilidade direta/pessoal (direcionado ao departamento ou instituição) provenientes da indústria farmacêutica, de órteses, próteses, equipamentos e implantes, brasileiras ou estrangeiras: - Besins, Theramex.
Maria Alayde Mendonça Rivera	Nada a ser declarado
Maria Antonieta Albanez Albuquerque de Medeiros Lopes	Declaração financeira A - Pagamento de qualquer espécie e desde que economicamente apreciáveis, feitos a (i) você, (ii) ao seu cônjuge/ companheiro ou a qualquer outro membro que resida com você, (iii) a qualquer pessoa jurídica em que qualquer destes seja controlador, sócio, acionista ou participante, de forma direta ou indireta, recebimento por palestras, aulas, atuação como proctor de treinamentos, remunerações, honorários pagos por participações em conselhos consultivos, de investigadores, ou outros comitês, etc. Provenientes da indústria farmacêutica, de órteses, próteses, equipamentos e implantes, brasileiras ou estrangeiras: - Boston, Medtronic, Daiichi Sankyo. Outros relacionamentos Financiamento de atividades de educação médica continuada, incluindo viagens, hospedagens e inscrições para congressos e cursos, provenientes da indústria farmacêutica, de órteses, próteses, equipamentos e implantes, brasileiras ou estrangeiras: - Boston.
Maria Celeste Osorio Wender	Nada a ser declarado
Maria Cristina Costa de Almeida	Nada a ser declarado
Maria Cristina de Oliveira Izar	Declaração financeira A - Pagamento de qualquer espécie e desde que economicamente apreciáveis, feitos a (i) você, (ii) ao seu cônjuge/ companheiro ou a qualquer outro membro que resida com você, (iii) a qualquer pessoa jurídica em que qualquer destes seja controlador, sócio, acionista ou participante, de forma direta ou indireta, recebimento por palestras, aulas, atuação como proctor de treinamentos, remunerações, honorários pagos por participações em conselhos consultivos, de investigadores, ou outros comitês, etc. Provenientes da indústria farmacêutica, de órteses, próteses, equipamentos e implantes, brasileiras ou estrangeiras: - Amgen: Repatha; Amryt Pharma: Lojuxta; AstraZeneca: Dapagliflozina; Aché: Trezor, Trezete; Biolab: Livalo, Posicor, Repatha; Abbott: Lipidil; EMS: Rosuvastatina; Eurofarma: Rosuvastatina; Sanofi: Praluent, Zympass, Zympass Eze, Efluelda; Libbs: Plenance, Plenance Eze; NovoNordisk: Ozempic; Servier: Acertamlo, Acertalix; PTCBio: Waylivra; Ultragenyx: Evkeeza; Alnylam: AMVUTTRA; GSK: Shingrix, Arexvy. B - Financiamento de pesquisas sob sua responsabilidade direta/pessoal (direcionado ao departamento ou instituição) provenientes da indústria farmacêutica, de órteses, próteses, equipamentos e implantes, brasileiras ou estrangeiras: - PTCBio: Waylivra; Amgen: Repatha; Novartis: Inclisiran, Pelacarsen; NovoNordisk: Ziltivekimab. Outros relacionamentos Financiamento de atividades de educação médica continuada, incluindo viagens, hospedagens e inscrições para congressos e cursos, provenientes da indústria farmacêutica, de órteses, próteses, equipamentos e implantes, brasileiras ou estrangeiras: - Novo Nordisk: Diabetes, Ziltivekimabe; GSK: vacinas. Possui qualquer outro interesse (financeiro ou a qualquer outro título) que deva ser declarado tendo em vista o cargo ocupado na SBC, ainda que não expressamente elencado anteriormente: - Membro do Comitê Gestor da Rede Hipertri Brasil.
Maria Elizabeth Navegantes Caetano Costa	Declaração financeira A - Pagamento de qualquer espécie e desde que economicamente apreciáveis, feitos a (i) você, (ii) ao seu cônjuge/ companheiro ou a qualquer outro membro que resida com você, (iii) a qualquer pessoa jurídica em que qualquer destes seja controlador, sócio, acionista ou participante, de forma direta ou indireta, recebimento por palestras, aulas, atuação como proctor de treinamentos, remunerações, honorários pagos por participações em conselhos consultivos, de investigadores, ou outros comitês, etc. Provenientes da indústria farmacêutica, de órteses, próteses, equipamentos e implantes, brasileiras ou estrangeiras: - Libbs: Plenance Enze; Servier: Vastarel. Outros relacionamentos Financiamento de atividades de educação médica continuada, incluindo viagens, hospedagens e inscrições para congressos e cursos, provenientes da indústria farmacêutica, de órteses, próteses, equipamentos e implantes, brasileiras ou estrangeiras: - Libbs; Servier: Participação em congresso.
Maria Sanali Moura de Oliveira Paiva	Outros relacionamentos Participação societária de qualquer natureza e qualquer valor economicamente apreciável de empresas na área de saúde, de ensino ou em empresas concorrentes ou fornecedoras da SBC: - Cardiologia.
Marildes Luiza de Castro	Declaração financeira A - Pagamento de qualquer espécie e desde que economicamente apreciáveis, feitos a (i) você, (ii) ao seu cônjuge/ companheiro ou a qualquer outro membro que resida com você, (iii) a qualquer pessoa jurídica em que qualquer destes seja controlador, sócio, acionista ou participante, de forma direta ou indireta, recebimento por palestras, aulas, atuação como proctor de treinamentos, remunerações, honorários pagos por participações em conselhos consultivos, de investigadores, ou outros comitês, etc. Provenientes da indústria farmacêutica, de órteses, próteses, equipamentos e implantes, brasileiras ou estrangeiras: - Novartis: Sacubitril/Valsartana; Pfizer: Patisiran; Merck: Vericiquat; Amgen.
Milena dos Santos Barros Campos	Nada a ser declarado
Olga Ferreira de Souza	Nada a ser declarado
Orlando Otávio de Medeiros	Nada a ser declarado
Rafaela Andrade Penalva Freitas	Declaração financeira A - Pagamento de qualquer espécie e desde que economicamente apreciáveis, feitos a (i) você, (ii) ao seu cônjuge/ companheiro ou a qualquer outro membro que resida com você, (iii) a qualquer pessoa jurídica em que qualquer destes seja controlador, sócio, acionista ou participante, de forma direta ou indireta, recebimento por palestras, aulas, atuação como proctor de treinamentos, remunerações, honorários pagos por participações em conselhos consultivos, de investigadores, ou outros comitês, etc. Provenientes da indústria farmacêutica, de órteses, próteses, equipamentos e implantes, brasileiras ou estrangeiras: - AstraZeneca: diabetes; NovoNordisk: diabetes, obesidade; Daiichi Sankyo Brasil: dislipidemia; Servier: doença coronária crônica; Libbs: diabetes; Mantecorp: diabetes. Outros relacionamentos Financiamento de atividades de educação médica continuada, incluindo viagens, hospedagens e inscrições para congressos e cursos, provenientes da indústria farmacêutica, de órteses, próteses, equipamentos e implantes, brasileiras ou estrangeiras: - AstraZeneca: diabetes; Novartis: dislipidemia; Novo Nordisk: diabetes, obesidade.
Regina Coeli Marques de Carvalho	Nada a ser declarado
Sheyla Cristina Tonheiro Ferro da Silva	Declaração financeira A - Pagamento de qualquer espécie e desde que economicamente apreciáveis, feitos a (i) você, (ii) ao seu cônjuge/ companheiro ou a qualquer outro membro que resida com você, (iii) a qualquer pessoa jurídica em que qualquer destes seja controlador, sócio, acionista ou participante, de forma direta ou indireta, recebimento por palestras, aulas, atuação como proctor de treinamentos, remunerações, honorários pagos por participações em conselhos consultivos, de investigadores, ou outros comitês, etc. Provenientes da indústria farmacêutica, de órteses, próteses, equipamentos e implantes, brasileiras ou estrangeiras: - AstraZeneca: diabetes; NovoNordisk: diabetes, obesidade; Daiichi Sankyo Brasil: dislipidemia; Servier: doença coronária crônica; Libbs: diabetes; Mantecorp: diabetes. Outros relacionamentos Financiamento de atividades de educação médica continuada, incluindo viagens, hospedagens e inscrições para congressos e cursos, provenientes da indústria farmacêutica, de órteses, próteses, equipamentos e implantes, brasileiras ou estrangeiras: - AstraZeneca: diabetes; Novartis: dislipidemia; Novo Nordisk: diabetes, obesidade.
Thais de Carvalho Vieira Rodrigues	Nada a ser declarado
Viviana de Mello Guzzo Lemke	Nada a ser declarado
Walkiria Samuel Avila	Nada a ser declarado
Wellington Santana da Silva Júnior	Declaração financeira A - Pagamento de qualquer espécie e desde que economicamente apreciáveis, feitos a (i) você, (ii) ao seu cônjuge/ companheiro ou a qualquer outro membro que resida com você, (iii) a qualquer pessoa jurídica em que qualquer destes seja controlador, sócio, acionista ou participante, de forma direta ou indireta, recebimento por palestras, aulas, atuação como proctor de treinamentos, remunerações, honorários pagos por participações em conselhos consultivos, de investigadores, ou outros comitês, etc. Provenientes da indústria farmacêutica, de órteses, próteses, equipamentos e implantes, brasileiras ou estrangeiras: - Abbott, AstraZeneca, Brace Pharma, Libbs, Lilly, Novartis, Novo Nordisk. Outros relacionamentos Financiamento de atividades de educação médica continuada, incluindo viagens, hospedagens e inscrições para congressos e cursos, provenientes da indústria farmacêutica, de órteses, próteses, equipamentos e implantes, brasileiras ou estrangeiras: - AstraZeneca, Novo Nordisk.
Willyan Issamu Nazima	Nada a ser declarado

## Sumário

**1. Introdução** 16
**2. Highlights** 19
**3. Inflamação e suas Implicações no Sistema Cardiovascular** 24
**3.1. Inflamação e Aterosclerose** 24
**3.2. Inflamação e Insuficiência Cardíaca na Mulher** 25
**3.3. Aspectos Hormonais na Inflamação** 26
**3.4. Inflamação na Cardio-oncologia** 26
**3.5. Inflamação na Gravidez e Pré-eclâmpsia** 27
**4. Implicação dos Esteroides Sexuais na Saúde Cardiometabólica** 29
**4.1. Origem e Metabolismo dos Esteroides Sexuais** 29
**4.2. Classificação Química dos Estrogênios** 29
**4.2.1. Estrogênios Naturais** 29
**4.2.2. Estrogênios Sintéticos** 29
**4.3. Classificação Química dos Progestagênios** 29
**4.3.1. Progesterona Natural** 29
**4.3.2. Progestagênios** 29
**4.4. Indicações Comuns de Terapêuticas com Estrogênios, Progestagênios e Testosterona na Mulher** 30
**4.4.1. Síndrome dos Ovários Policísticos** 30
**4.4.2. Contracepção Hormonal** 31
**4.4.3. Terapia Hormonal da Menopausa: Regimes, Doses e Vias, com Foco na Saúde Cardiovascular** 33
**4.5. Riscos: Efeitos Metabólicos, Tromboembolismo e Câncer de Mama** 33
**4.5.1. Efeitos Metabólicos** 33
**4.5.2. Tromboembolismo** 33
**4.5.3. Câncer de Mama** 34
**5. Indicadores de Saúde Cardiometabólica** 34
**5.1. Avaliação de Fatores de Risco Reprodutivos com Implicações na Saúde Cardiometabólica** 34
**5.1.1. Puberdade e Período Pré-gestacional** 34
***5.1.1.1. Idade da Menarca*** 34
***5.1.1.2. Características do Ciclo Menstrual*** 34
**5.1.2. Gestação e Período Pós-parto** 34
***5.1.2.1. Alterações de Peso na Gestação*** 34
***5.1.2.2. Disglicemia Gestacional*** 35
***5.1.2.3. Dislipidemia Gestacional*** 36
***5.1.2.4. Comportamento Pós-parto*** 36
**5.1.3. Transição da Menopausa** 36
***5.1.3.1. Idade da Menopausa*** 36
***5.1.3.2. Sintomas Vasomotores*** 36
***5.1.3.3. Terapia Hormonal da Menopausa*** 36
**5.1.4. Saúde Metabólica Pós-menopausa** 37
***5.1.4.1. Mudanças Metabólicas na Menopausa*** 37
***5.1.4.2. Diabetes Mellitus*** 37
***5.1.4.3. Dislipidemia*** 37
**5.2. Indicadores Antropométricos** 37
**5.2.1. Índice de Massa Corpórea** 37
**5.2.2. Circunferência da Cintura – População Brasileira** 37
**5.2.3. Razão Cintura/Quadril** 37
**5.2.4. Percentual de Gordura Corporal** 38
**5.2.5. Razão Cintura/Estatura** 38
**5.3. Biomarcadores** 38
**5.3.1. Perfil Lipídico (Colesterol Total e Frações LDL e HDL; Triglicerídeos)** 38
**5.3.2. Glicemia e Hemoglobina Glicada** 38
**5.3.3. Proteína C Reativa** 38
**5.3.4. Fibrinogênio** 38
**5.3.5. Homocisteína** 39
**5.3.6. Adipocinas** 39
**6. Cardiometabolismo no Período Infanto-juvenil** 39
**6.1. Definições e Critérios Diagnósticos** 40
**6.1.1. Fisiopatologia** 40
**6.1.2. Menarca Precoce, Menarca Tardia e Risco Cardiometabólico** 40
**6.2. Irregularidades Menstruais e Risco Cardiometabólico** 40
**6.2.1. Mecanismos Fisiopatológicos** 41
**6.2.2. Avaliação Clínica e Biomarcadores** 41
**6.2.3. Intervenções Terapêuticas** 41
**6.3. Obesidade, Transtornos Alimentares e Risco Cardiovascular: Impactos a Longo Prazo** 42
**6.3.1. Transtornos Alimentares e Obesidade** 42
**6.3.2. Fatores Hereditários e Prognóstico dos Transtornos Alimentares** 42
**6.3.3. Consequências Futuras dos Transtornos Alimentares** 42
**6.3.4. Obesidade Infantil e Desfechos Cardiovasculares** 42
**6.3.5. Transtornos Alimentares e Risco Cardiovascular** 43
**7. Continuum Cardiometabólico e Idade Reprodutiva** 43
  7.1 Síndrome dos Ovários Policísticos 43
**7.2. Infertilidade e seu Tratamento** 44
**7.3. Modificações do Peso na Gestação** 45
**7.3.1. Impacto Materno do Ganho de Peso Gestacional Excessivo** 45
**7.3.2. Impacto Fetal do Ganho de Peso Gestacional Excessivo** 46
**7.4. Disglicemia Gestacional** 46
**7.5. Dislipidemia Gestacional** 47
**7.5.1. Impacto da Dislipidemia na Gravidez** 48
**7.5.2. Impacto da Gravidez em Pacientes com Hipercolesterolemia Familiar** 49
**7.6. Endometriose e Risco Cardiovascular** 49
**7.6.1. Fatores de Risco** 50
**7.7. Psoríase** 50
**7.8. Distúrbios Hipertensivos da Gestação e Disfunção Endotelial após a Menopausa** 50
**7.9. Alterações Metabólicas no Período Pós-parto** 50
**7.9.1. Fatores de Risco Cardiometabólico no Pós-parto** 51
**7.9.2. Impacto de Diabetes Gestacional e Dislipidemia no Pós-parto** 51
**7.9.3. Prolactina e Metabolismo no Pós-parto** 51
**8. Transição Menopausal, Menopausa e Pós-menopausa e Saúde Cardiometabólica** 52
**8.1. Gravidade dos Sintomas e suas Implicações na Saúde Cardiovascular** 52
**8.2. Fatores de Risco Cardiovascular e Menopausa** 52
**8.3. Terapia de Reposição Hormonal da Menopausa, Implantes Hormonais e Impacto Cardiometabólico** 54
**8.4. Estratificação do Risco Cardiometabólico na Menopausa** 55
**9. Distúrbios Cardiometabólicos nas Mulheres** 55
**9.1. Obesidade e Síndrome Metabólica** 56
**9.2. Diabetes Mellitus Tipo 2** 56
**9.3. Doença Hepática Esteatótica Metabólica** 57
**9.4. Doença Renal Crônica** 59
**10. Estratégias para Abordar os Distúrbios Cardiometabólicos nas Mulheres** 60
**10.1. Medidas Não Farmacológicas** 60
**10.2. Intervenções Nutricionais** 60
**10.3. Atividade Física** 60
**10.4. Intervenções Psicossociais** 60
**10.5. Tabagismo e Consumo de Álcool** 60
**10.6. Condições Clínicas Específicas** 61
**10.7. Estratégias Farmacológicas** 61
**10.7.1. Tratamento da Hipertensão Arterial Sistêmica** 61
**10.7.2. Manejo das Dislipidemias** 61
**10.7.3. Hipolipemiantes Orais** 62
**10.7.4. Controle do Diabetes Mellitus** 64
**10.7.5. Manejo da Obesidade** 64
**10.7.6. Manejo da Doença Hepática Esteatótica Metabólica** 65
**10.7.7. Manejo da Doença Renal Crônica** 65
**10.7.8. Papel dos Análogos de GLP-1 no Tratamento Cardiometabólico em Mulheres** 66
**10.7.9. Especificidades em Mulheres** 66
**10.7.10. Semaglutida** 66
**10.7.11. Tirzepatida** 66
**10.7.12. Perspectivas Futuras** 67
**10.8. Considerações Farmacológicas Específicas** 67
**10.8.1. Interações Medicamentosas** 67
**10.8.2. Importância do Controle de Peso** 67
**10.8.3. Abordagem Individualizada** 67
**10.9. Tratamento Cirúrgico** 67
**10.8.1. Cirurgia Bariátrica** 67
**11. Recomendações para o Manejo dos Distúrbios Cardiometabólicos nas Mulheres** 67
Agradecimentos 69
Suplemento 74
Referências 85

**Figure:**
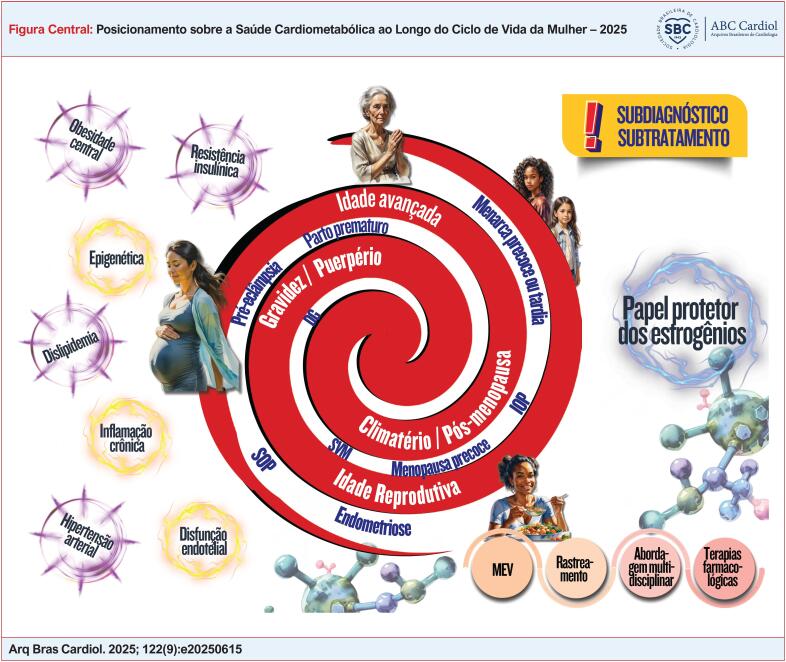
Ciclo de vida da mulher e o continuum cardiometabólico: fatores de risco e estratégias. Espiral representando o ciclo de vida da mulher (fases) e sua associação com o continuum cardiometabólico. Os eventos reprodutivos estão escritos em azul, marcando cada fase em que se apresentam, como idade da menarca (precoce ou tardia), SOP, DG, etc. Os principais fatores de risco cardiometabólico, como resistência à insulina, dislipidemia, obesidade central e inflamação crônica, estão representados ao redor da espiral, onde se destacam ainda os principais pontos de intervenção ao longo da vida, incluindo rastreamento precoce, terapias farmacológicas, mudanças no estilo de vida e abordagem multidisciplinar, visando à prevenção primária e secundária da doença cardiovascular na mulher. DG: diabetes gestacional; SOP: síndrome dos ovários policísticos; IOP: insuficiência ovariana prematura; SVM: sintomas vasomotores; MEV: mudanças no estilo de vida.

## 1. Introdução

**Figura 1.1:**
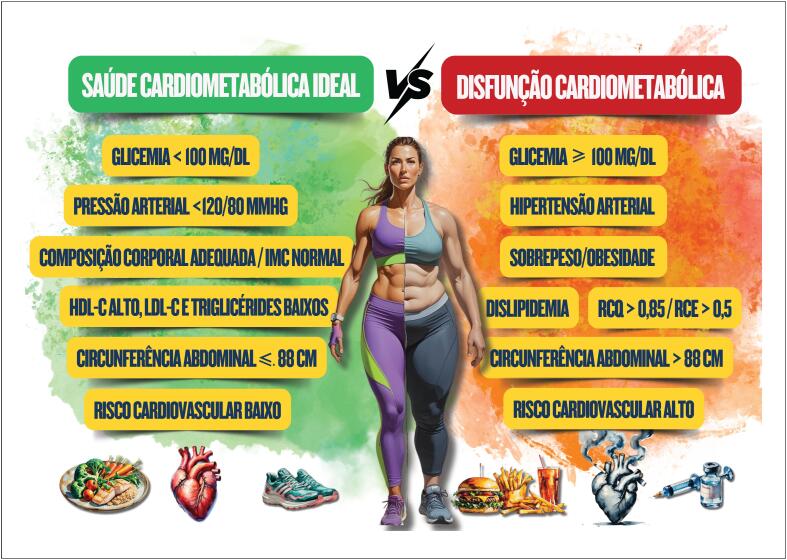
O espectro da saúde cardiometabólica. IMC: índice de massa corpórea; RCQ: razão cintura/quadril; RCE: razão cintura/estatura.

A saúde cardiometabólica pode ser caracterizada por níveis ideais de glicose e lipídios séricos e de pressão arterial (PA), associados com adiposidade e risco cardiovascular (RCV) baixos. Usualmente, a saúde cardiometabólica é a ausência de disfunção metabólica característica de doenças, que incluem doenças cardiovasculares (DCV), diabetes *mellitus* tipo 2 (DM2) e síndrome metabólica (SM). A má saúde metabólica é responsável por uma carga populacional substancial de incapacidade, doença e morte por causas cardiovasculares e neoplásicas e por todas as causas. É cada vez mais reconhecido que diferentes características relacionadas à reprodução estão associadas a doenças metabólicas com base na epidemiologia do curso de vida, que postula que fatores biológicos, comportamentais e sociais durante estágios sensíveis da vida, mediados pela flutuação hormonal, agem de forma independente, cumulativa e interativa para influenciar o risco posterior de saúde e doença^1 , 2^ ( Figura 1.1 ).

A prevalência de distúrbios cardiometabólicos está aumentando em todo o mundo, tanto em homens quanto em mulheres, e foi associada com maiores taxas de obesidade e seus fatores de risco (FR), como hipertensão e DM2. Adicionalmente, evidências crescentes sugerem que hormônios sexuais, mecanismos moleculares específicos do sexo e gênero podem influenciar o metabolismo da glicose e dos lipídios, impactando, assim, nos FR cardiometabólicos. Há também uma predominância emergente de tipos comuns de distúrbios cardiometabólicos, como insuficiência cardíaca (IC), fibrilação atrial e doença isquêmica do coração (DIC), que diferem entre pacientes do sexo masculino e feminino. Variações significativas específicas do sexo foram relatadas nos perfis de risco, ressaltando-se especialmente sua importância nas mulheres, nas quais os escores de risco são inadequados tanto para predizer as doenças aterotrombóticas, quanto para os desfechos relacionados, como morte cardiovascular, infarto do miocárdio (IM), acidente vascular cerebral (AVC), IC, arritmias ventriculares e supraventriculares, e necessidade de revascularização e de hospitalizações.^2^

**Figura 1.2:**
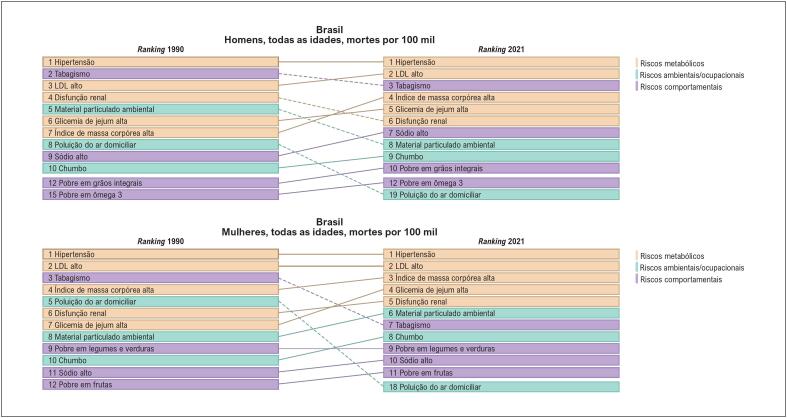
Ranking das taxas de mortalidade por 100 mil habitantes por doença cardiovascular atribuível aos fatores de risco, em homens e mulheres, no Brasil, em 2021.

Se considerarmos o *ranking* das taxas de mortes e DALYs ( *Disability-Adjusted Life Years* - Anos de Vida Ajustados por Incapacidade, uma medida que representa a carga de doença numa população) por 100 mil habitantes por DCV, atribuíveis aos FR em homens e mulheres no Brasil em 2021, poderemos observar que os FR metabólicos representam os cinco primeiros elencados nas mulheres para as mortes, e os quatro primeiros para os DALYs. Destaca-se que o aumento da PA sistólica, do colesterol de lipoproteína de baixa densidade (LDL-c), da massa corporal e da glicose sérica, nessa ordem, é comum para a ocorrência de morte e carga de DCV na população feminina brasileira. Além disso, a disfunção renal se apresenta como importante FR metabólico em homens e mulheres. É importante notar o aumento da massa corporal e da glicose sérica nos últimos 21 anos, representando um incremento relevante de obesidade e diabetes *mellitus* (DM) em homens e mulheres^3^ ( Figuras 1.2 f e 1.3 ).

**Figura 1.3:**
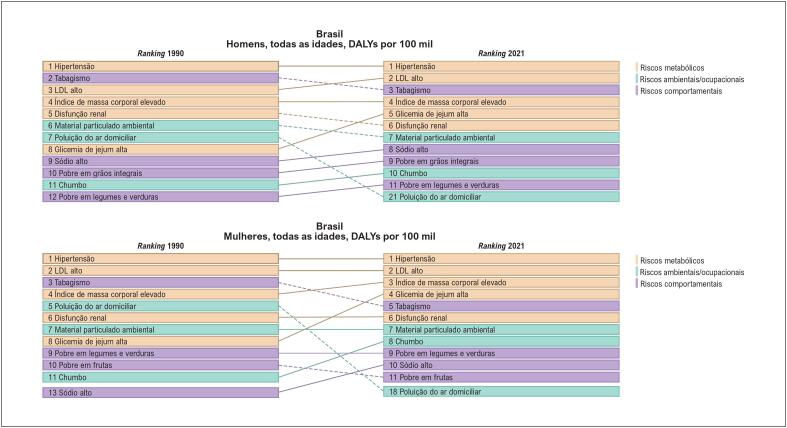
Ranking das taxas de DALYs por 100 mil habitantes por doença cardiovascular atribuível aos fatores de risco, em homens e mulheres, no Brasil, em 2021.^3^

Evidências recentes sugerem que as características reprodutivas femininas podem estar relacionadas com FR que contribuem para a disfunção metabólica posterior com ocorrência de DCV na menopausa. Essas características reprodutivas incluem: idade da menarca, irregularidade menstrual por causas endócrinas, desenvolvimento da síndrome dos ovários policísticos (SOP), ganho de peso excessivo na gestação, disglicemia e dislipidemia gestacionais, distúrbios hipertensivos da gestação, gravidade e momento dos sintomas da menopausa e o efeito do hipoestrogenismo sobre o sistema cardiovascular. Esses FR podem ser marcadores de disfunção futura ou podem ser explicados por etiologias subjacentes compartilhadas que promovem o desenvolvimento da doença a longo prazo. Identificar características potencialmente modificáveis têm uma influência importante nas estratégias para implementação de estilo de vida saudável, de terapias medicamentosas e cirúrgicas que podem aliviar a carga metabólica a longo prazo.^4 - 6^

**Figura 1.4:**
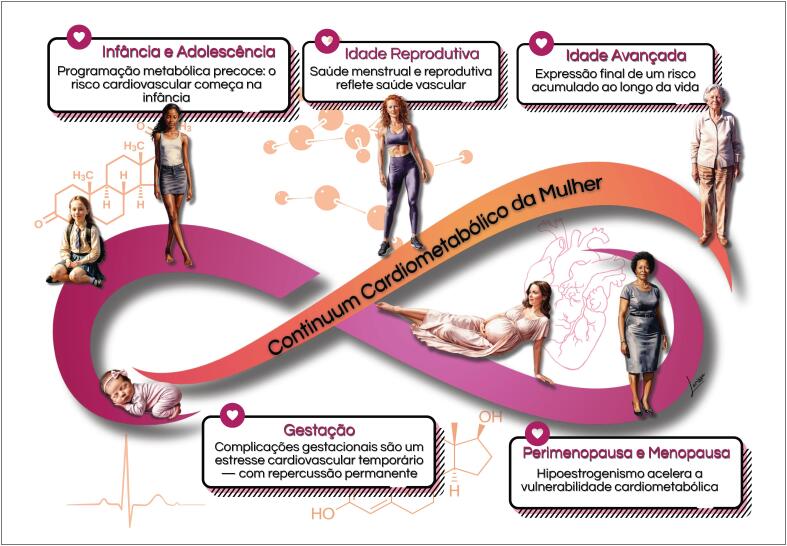
Continuum cardiometabólico nas diversas fases da vida da mulher.

O *continuum* cardiometabólico, sequência de eventos cardiovasculares que decorrem de interações gene-ambientais, influências de estilos de vida pouco saudáveis e doenças metabólicas, como DM e hipertensão arterial sistêmica (HAS), ocorre de forma predominante ao longo do ciclo de vida das mulheres ( Figura 1.4 ). Em estudo recente, o *continuum* cardiometabólico foi investigado para avaliar as diferenças entre sexo e população em duas coortes distintas: o UK Biobank (17.700 participantes) e o Estudo Longitudinal Brasileiro de Saúde do Adulto (ELSA-Brasil, com 7.162 participantes). Os autores estudaram o *continuum* cardiometabólico, empregando *machine learning* , e identificaram cinco padrões. Descreveram desvantagem feminina em termos do tempo de surgimento do *continuum* cardiometabólico. Na coorte do UK Biobank, quando a HAS foi a primeira doença no *continuum* cardiometabólico e foi diagnosticada isoladamente, ela ocorreu mais rapidamente entre as mulheres. Na coorte ELSA-Brasil, não apenas o DM ocorreu com mais frequência como a primeira doença no *continuum* cardiometabólico entre mulheres, mas também foi diagnosticado mais rapidamente quando seguido pela HAS. Além disso, as mulheres apresentaram maior incidência de HAS e DM isolados, e uma porcentagem menor delas foi classificada como saudável. Os autores destacam o acesso desigual a tratamento e diagnóstico adequados para o *continuum* cardiometabólico nas mulheres e enfatizam que políticas de saúde diferenciadas por sexo são particularmente mais necessárias no Brasil para reduzir as desigualdades existentes.^7^

O sistema cardiovascular, os rins e o fígado partilham FR, como dislipidemia, hipertensão, tabagismo, DM e obesidade central/troncal, e distúrbios metabólicos e funcionais compartilhados levam a danos nesses órgãos por meio de vias fisiopatológicas sobrepostas, nas quais são adicionadas as influências hormonais derivadas dos diversos ciclos de vida das mulheres. O crescimento dos FR metabólicos ao longo dos anos alerta para a necessidade de se aprimorarem o reconhecimento e o tratamento da doença cardiometabólica em mulheres, em especial por serem subdiagnosticadas e subtratadas, além de sua pequena participação nos ensaios clínicos que norteiam as estratégias terapêuticas atualmente disponíveis, que incluem modificações no estilo de vida, farmacoterapia e cirurgia. Ademais, o caráter inflamatório, notadamente maior nas mulheres com distúrbios cardiometabólicos, e as novas opções terapêuticas farmacológicas e cirúrgicas, que oferecem abordagens baseadas em mecanismos que podem atingir múltiplas vias fisiopatológicas que sofrem influências hormonais, precisam ser mais estudados no sexo feminino. Há necessidade de programas específicos em nível social para incentivar dieta saudável e atividade física, além da melhora do acesso a políticas públicas voltadas para mulheres com distúrbios cardiometabólicos.^4 , 8^

Desse modo, o *Posicionamento Sobre a Saúde Cardiometabólica ao Longo do Ciclo de Vida das Mulheres* é um esforço conjunto do Departamento de Cardiologia da Mulher da Sociedade Brasileira de Cardiologia (DCM/SBC), da Federação Brasileira das Associações de Ginecologia e Obstetrícia (FEBRASGO) e da Sociedade Brasileira de Endocrinologia e Metabologia (SBEM) para diminuir a lacuna de conhecimento sobre os distúrbios cardiometabólicos nas mulheres.

A seguir apresentamos os *Highlights* desse posicionamento distribuídos por seus respectivos capítulos.

## 2. Highlights

### Capítulo 3

- Na mulher, a menopausa e as complicações da gravidez (particularmente pré-eclâmpsia/eclâmpsia) ativam a inflamação e aceleram a aterosclerose coronariana, promovendo rigidez vascular, disfunção endotelial e isquemia microvascular;
- Variações biológicas entre mulheres e homens decorrem de diferenças na expressão gênica dos cromossomos sexuais moduladas por influências hormonais e ambientais, que resultam em condições cardiovasculares associadas à regulação autonômica e remodelação vascular e cardíaca;
- Mulheres com ciclos irregulares por causas endócrinas frequentemente exibem estados pró-inflamatórios com elevação de marcadores inflamatórios que atuam como intermediadores na gênese aterosclerótica;
- O aumento da prevalência de fatores de risco nas mulheres, como obesidade, diabetes e hipertensão, associados aos efeitos da menopausa, parecem explicar a maior prevalência de insuficiência cardíaca com fração de ejeção preservada;
- A associação entre inflamação crônica e adiposidade na insuficiência cardíaca com fração de ejeção preservada parece estar relacionada a adipocinas no pericárdio e epicárdio, levando a inflamação na microcirculação, fibrose cardíaca e alteração do enchimento diastólico fisiológico;
- O hipoestrogenismo da pós-menopausa promove alterações metabólicas, como o aumento da adiposidade central, piora do metabolismo glicídico e elevação dos níveis de colesterol total, LDL-c e triglicerídeos, apolipoproteínas e lipoproteína a.

### Capítulo 4

- Os hormônios esteroides sexuais ligam-se aos receptores hormonais presentes em diversos tecidos, exercendo seus múltiplos efeitos biológicos;
- Terapias com uso de diferentes hormônios esteroides são indicações frequentes para tratamento de condições como síndrome dos ovários policísticos, contracepção e terapia de reposição hormonal da menopausa, condições que podem ter implicações no risco cardiometabólico;
- Os contraceptivos orais combinados não estão recomendados para mulheres com histórico pessoal de tabagismo acima de 35 anos, doenças cardiovasculares e tromboembolismo venoso, diabetes com complicação vascular, enxaqueca com sinais neurológicos, hepatopatia grave, tumores hepáticos, câncer de mama e lúpus eritematoso sistêmico;
- Os efeitos cardiovasculares da terapia de reposição hormonal da menopausa são influenciados por tipo e dose utilizados, via de administração e momento do início em relação à menopausa ("janela de oportunidade"). Sugerem-se maiores benefícios e menores efeitos adversos quando iniciada em mulheres com até 10 anos de pós-menopausa;
- Os fatores que determinam a escolha do tipo e da dose da terapia de reposição hormonal da menopausa são a preferência da paciente, presença ou ausência do útero, necessidade contraceptiva, intensidade dos sintomas e comorbidades associadas.

### Capítulo 5

- A avaliação do ciclo menstrual pode ser usada como um dado adicional na investigação do estado de saúde cardiometabólica das mulheres;
- Idade da menarca (precoce ou tardia), irregularidades do ciclo menstrual e síndrome dos ovários policísticos estão associadas a maior risco futuro de doenças cardiovasculares;
- Na gravidez, ganho de peso anormal e alterações no perfil lipídico e na glicemia podem estar associados a desfechos adversos na gestação e no pós-parto, tanto para a mulher como para o concepto;
- Hiperlipidemia durante a gravidez está associada a pré-eclâmpsia, parto prematuro e diabetes gestacional, apresentando os conceptos propensão à formação aumentada de estrias de gordura e risco aumentado de aterosclerose progressiva;
- A gravidez é uma condição de resistência à insulina por si só (40% a 50% de aumento) e essa resistência pode ser agravada em mulheres com obesidade pré-gravidez, que também apresentam maior risco de desenvolverem diabetes gestacional;
- Mulheres com intolerância à glicose na gestação correm risco de desfechos gestacionais adversos, mesmo que não tenham desenvolvido diabetes;
- Na menopausa precoce e na presença de sintomas vasomotores de moderada a grave intensidade, existe maior propensão a um perfil lipídico mais aterogênico, resistência à insulina, maior elevação da pressão arterial, maior risco de síndrome metabólica, bem como piora da função endotelial e dos marcadores inflamatórios;
- Os indicadores antropométricos, assim como os biomarcadores (perfil lipídico e glicêmico, proteína C reativa, fibrinogênio, homocisteína e adipocina), fornecem informações sobre a composição e a distribuição de gordura, refletindo o risco cardiometabólico.

### Capítulo 6

- Idade precoce ou tardia da menarca e irregularidades menstruais de causas endócrinas são importantes marcadores de risco metabólico e cardiovascular, cuja identificação precoce direciona para medidas preventivas, visando reduzir risco metabólico e carga de doenças cardiovasculares na população feminina;
- A obesidade e os transtornos alimentares na infância e adolescência envolvem aspectos genéticos, metabólicos, comportamentais e ambientais e impactam de forma expressiva a saúde cardiovascular e mental ao longo da vida adulta;
- A identificação precoce, o monitoramento contínuo e a implementação de estratégias terapêuticas desde a infância são essenciais para interromper o ciclo vicioso de obesidade, transtornos alimentares e suas complicações, minimizando os desfechos adversos a longo prazo.

### Capítulo 7

- Mulheres com síndrome dos ovários policísticos podem apresentar resistência à insulina e dislipidemia, que contribuem para o desenvolvimento de doenças cardiovasculares pelo excesso de androgênios, além do ganho de peso, com acúmulo de gordura visceral e processo inflamatório crônico;
- A endometriose está associada a processo inflamatório crônico com aumento do estresse oxidativo e elevação de fatores de risco cardiovascular, aumento do risco de tromboembolismo venoso, doença isquêmica do coração, insuficiência cardíaca e acidente vascular cerebral;
- Tratamento para infertilidade pode acarretar repercussões no cardiometabolismo, como a síndrome de hiperestimulação ovariana, com elevação do risco de eventos tromboembólicos, pré-eclâmpsia, diabetes gestacional e distúrbios hipertensivos da gestação, aumentando os eventos cardiovasculares em longo prazo;
- A psoríase é doença inflamatória crônica sistêmica associada a obesidade, síndrome metabólica, doenças cardio- e cerebrovasculares, arritmias cardíacas, apneia do sono, entre outras;
- A pré-eclâmpsia é considerada como marcador de risco para doenças cardiovasculares ao longo da vida, que parece aumentar após a menopausa, com piora do perfil cardiometabólico;
- A retenção excessiva de peso no pós-parto está associada a maior risco de dislipidemia e resistência à insulina;
- Mulheres com histórico de diabetes gestacional têm maior probabilidade de apresentar distúrbios metabólicos no período pós-parto e por toda a vida, independentemente de outros fatores de risco cardiovascular tradicionais.

### Capítulo 8

- A diminuição dos níveis endógenos de estradiol durante a transição da menopausa associa-se a risco aumentado de distúrbios cardiometabólicos, como adiposidade abdominal, dislipidemia, diabetes *mellitus* tipo 2 e hipertensão arterial sistêmica, estando relacionada a desregulação do sistema renina-angiotensina-aldosterona, ativação simpática, disfunção endotelial, inflamação e maior sensibilidade ao sódio;
- As mulheres com insuficiência ovariana prematura e menopausa precoce apresentam risco maior de evento cardiovascular não fatal antes dos 60 anos;
- A terapia de reposição hormonal da menopausa é utilizada para aliviar os sintomas da menopausa, não sendo indicada para prevenção primária ou secundária de distúrbio cardiometabólico;
- A terapia de reposição de testosterona não está indicada para a melhora de saúde cardiometabólica ou musculoesquelética, sintomas vasomotores ou alterações de humor;
- Os implantes hormonais na menopausa, notadamente os de testosterona, não são recomendados por não se conhecerem seus efeitos cardiometabólicos nem o risco de câncer de mama e de endométrio.

### Capítulo 9

- O ganho de peso nas gestações e sua manutenção no pós-parto, assim como a síndrome dos ovários policísticos, são fatores de risco para obesidade e aumento da resistência à insulina e dos outros componentes da síndrome metabólica;
- As mulheres com diabetes *mellitus* tipo 2 apresentam maior risco de complicações cardiovasculares comparadas aos homens;
- Menopausa, história de menarca precoce e síndrome dos ovários policísticos estão associadas ao aumento da suscetibilidade das mulheres à doença hepática esteatótica metabólica, cuja taxa de mortalidade por cirrose é mais alta do que a dos homens, sendo a principal causa de transplante de fígado em mulheres sem carcinoma hepatocelular;
- Na doença renal crônica, as mulheres mostram maior prevalência de doença dialítica e maior velocidade de perda da taxa de filtração glomerular em relação aos homens, especialmente as mais idosas e no período pós-menopausa.

### Capítulo 10

- A crescente prevalência de distúrbios cardiometabólicos em mulheres representa um dos maiores desafios da saúde pública devido ao forte vínculo entre obesidade e doenças cardiovasculares;
- A reeducação nutricional é o pilar central na prevenção dos distúrbios cardiometabólicos, sendo que a associação de planos nutricionais à prática regular de atividade física potencializa os efeitos benéficos, favorecendo a função cardiovascular e diminuindo a morbimortalidade nas mulheres;
- Mulheres que participam de programas integrados de suporte psicológico apresentam melhora significativa no estilo de vida e melhores desfechos clínicos, como redução de peso, controle glicêmico e perfil lipídico;
- O tabagismo é importante fator inflamatório crônico, particularmente acentuado em mulheres na pós-menopausa;
- A atuação multidisciplinar tem se mostrado eficaz em gerar mudanças sustentáveis no estilo de vida, reduzindo a morbimortalidade por distúrbios cardiometabólicos;
- A hipertensão constitui o fator de risco mais prevalente para doenças cardiovasculares em mulheres, incidente em todas as fases da vida, com aumento progressivo ao avançar da idade. A escolha e a condução das estratégias farmacológicas devem considerar as fases do ciclo reprodutivo, incluindo a perimenopausa e a pós-menopausa. O uso de inibidores da enzima conversora de angiotensina e bloqueadores dos receptores da angiotensina II em mulheres em idade fértil exige cautela devido ao risco teratogênico;
- A menopausa associa-se a elevações significativas do colesterol total, LDL-c, apolipoproteína B, triglicerídeos e lipoproteína a, além da possível redução do efeito protetor antiaterogênico do HDL-c. Apesar de níveis de HDL-c acima de 50 mg/dL serem desejáveis em mulheres, a prioridade terapêutica permanece na redução do LDL-c;
- Mulheres apresentam progressão distinta do pré-diabetes para o diabetes *mellitus* tipo 2, frequentemente associada a maiores índices de obesidade e risco aumentado de complicações metabólicas. As mulheres podem apresentar maior risco de hipoglicemia com sulfonilureias e resposta distinta às glitazonas, relacionada à função renal e composição corporal. Quanto ao tratamento com análogos de GLP-1, especial cuidado deve ser tomado em mulheres em uso de contracepção oral;
- Existem diversos tratamentos farmacológicos para obesidade com efeitos variáveis sobre o peso corporal, mas apenas tratamentos mais recentes associaram-se à redução de desfechos cardiovasculares e metabólicos;
- O tratamento da doença hepática esteatótica metabólica consiste em modificações no estilo de vida com foco na redução de pelo menos 5% do peso corporal, uma vez que a perda de peso é a medida mais eficaz na melhora histológica da doença;
- A gravidez é uma das principais causas de lesão renal aguda em mulheres em idade fértil e, junto com a pré-eclâmpsia, pode levar a doença renal crônica subsequente. A doença renal crônica tem um efeito negativo na gravidez, mesmo em estágios muito iniciais, e os riscos aumentam com sua progressão e com a concomitância de diabetes *mellitus* tipo 2;
- A cirurgia bariátrica é recomendada para mulheres com índice de massa corpórea ≥ 35 kg/m² e histórico de diabetes, doença hepática esteatótica metabólica ou alto risco de eventos cardiovasculares, assim como para aquelas com índice de massa corpórea ≥ 40 kg/m², independentemente de comorbidades, para a melhoria dos parâmetros cardiometabólicos (perfil lipídico e glicêmico) e marcadores inflamatórios.

### Capítulo 11

**Figure:**
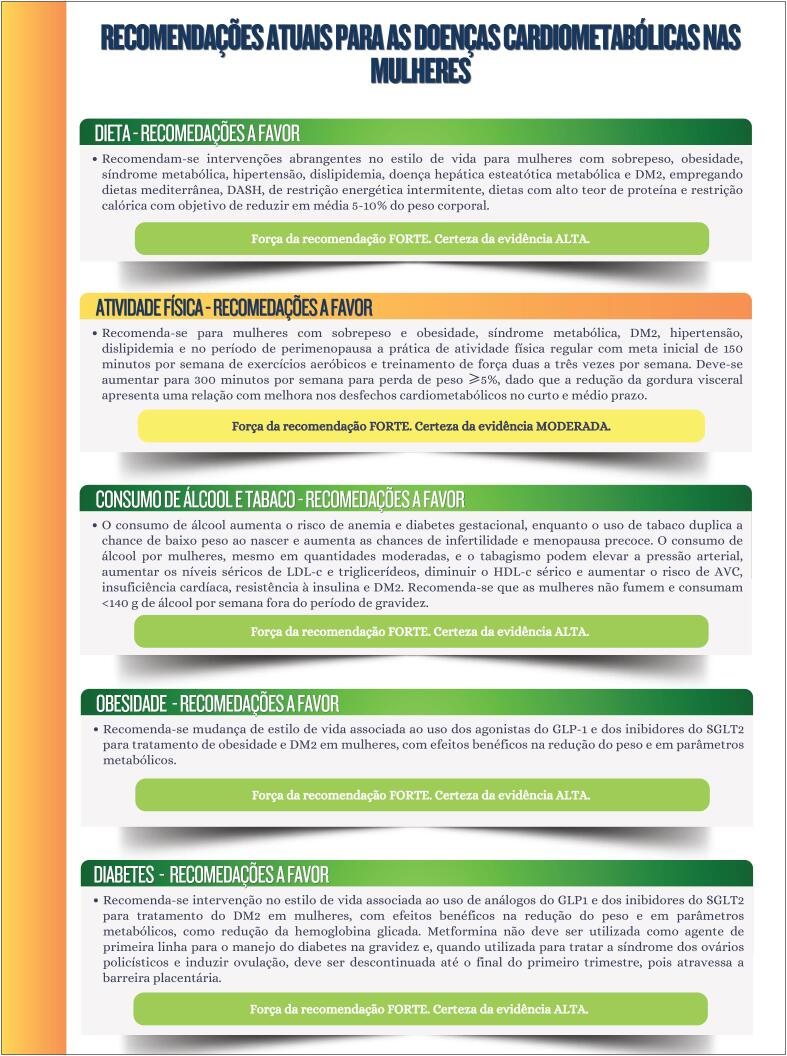


### Capítulo 11

**Figure:**
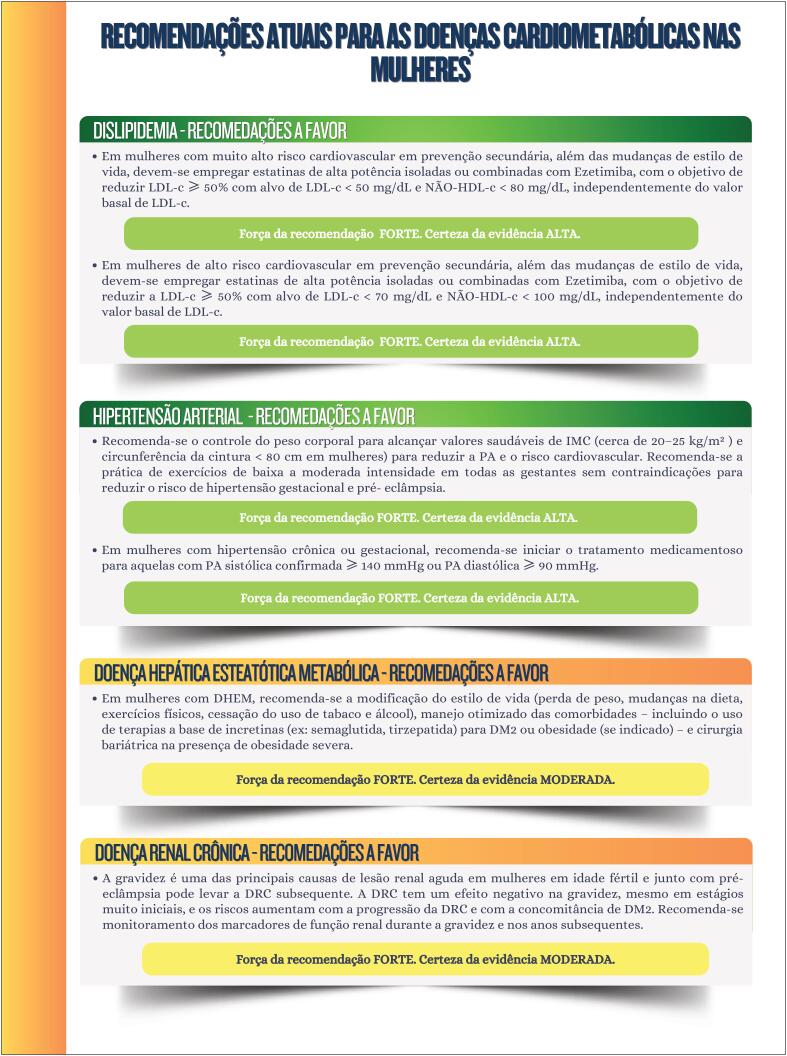


### Capítulo 11

**Figure:**
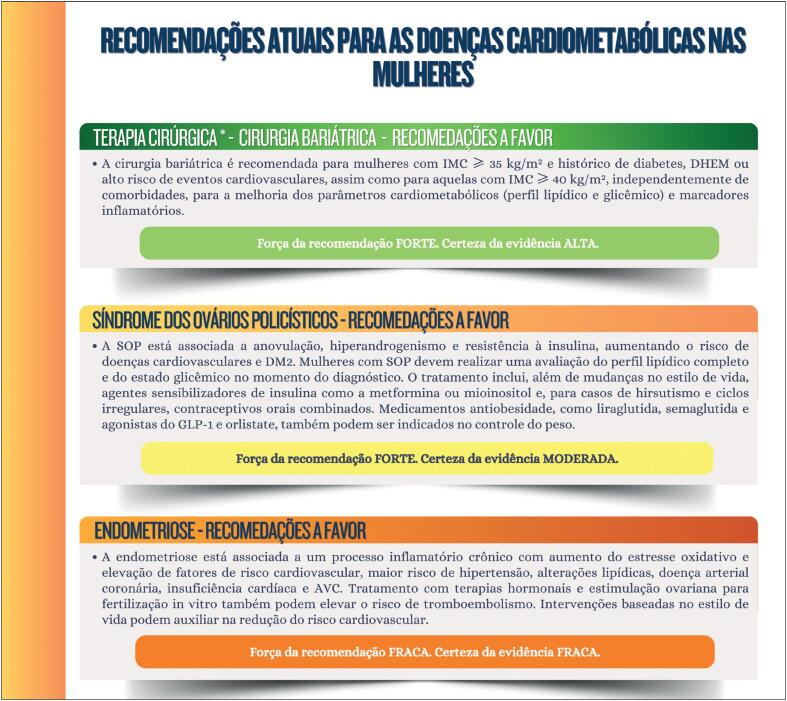


## 3. Inflamação e suas Implicações no Sistema Cardiovascular

Inflamação é um processo de defesa do organismo ativado por agentes infecciosos, doenças autoimunes e inflamatórias, sendo influenciado pelos FR clássicos como DM, hipertensão, dislipidemia, obesidade e tabagismo. Associadas a esses fatores cardiorrenais metabólicos, há também as alterações do envelhecimento, chamado imunossenescência ou *immuno-aging.*

Na mulher, a menopausa e a gravidez, particularmente pré-eclâmpsia /eclâmpsia, ativam a inflamação e aceleram a aterosclerose coronariana, promovendo rigidez vascular, disfunção endotelial e isquemia microvascular. São exemplos relevantes do papel da inflamação nas DCV notadamente nas mulheres: insuficiência cardíaca com fração de ejeção preservada (ICFEp) com seu fenótipo inflamatório; e isquemia/infarto com artérias coronárias normais (INOCA/MINOCA).

### 3.1. Inflamação e Aterosclerose

As DCV, em particular a cardiopatia isquêmica, são a causa de morte mais importante nas mulheres, principalmente entre 45 anos e 75 anos.^9 , 10^ Apesar de as diferenças sexuais no RCV estarem bem estabelecidas, não são completamente compreendidas.^11^ Nesse sentido, é imperativo um melhor entendimento dos mecanismos que contribuem para o agravamento dos perfis de risco em mulheres a fim de reduzir a morbimortalidade por essas doenças.

Muitos fatores contribuem para a desigualdade entre os sexos no diagnóstico e tratamento das DCV. Há consenso que as mulheres são sub-representadas nos grandes ensaios clínicos, como evidenciado no Estudo VIGO, que analisou 2.349 mulheres e 1.152 homens com infarto agudo do miocárdio (IAM) com menos de 55 anos de idade, na presença de três ou mais FR. O estudo demonstrou que as mulheres eram menos propensas a receber orientação sobre a prevenção cardiovascular.^12 , 13^ Esse cenário pode comprometer a capacidade de avaliar com precisão a eficácia e a segurança das diferentes terapias nas mulheres, dificultando, assim, estratégias específicas para a prevenção e o tratamento das DCV.^13^

Além dos FR clássicos, diversas condições emergentes devem ser consideradas no RCV feminino, como depressão, violência doméstica, perfil socioeconômico e cultural, histórico obstétrico e ginecológico, hipertensão gestacional, diabetes gestacional (DG), parto prematuro, menopausa prematura e SOP, câncer de mama, dentre outros.^10 , 14^

Na cardiopatia isquêmica, esses fatores refletem diferenças substanciais entre os sexos em termos de fisiopatologia, apresentação clínica e desfechos. A INOCA é mais frequente nas mulheres, especificamente entre 45 anos e 65 anos,^15^ assim como MINOCA.^16^ Embora o espasmo dos vasos epicárdicos seja mais comum em homens, até 70% dos casos de disfunção da microcirculação coronariana ocorrem em mulheres.^17^ A dissecção espontânea coronariana, embora rara, é causa de síndrome coronariana aguda em mulheres com menos de 50 anos.^18^

O processo aterosclerótico é conhecido há décadas e foi inicialmente associado à inflamação por Rudolph Virchow, que demonstrou que a formação e a evolução da placa aterosclerótica nas síndromes coronarianas, tanto na obstrução crônica quanto na ruptura da placa, têm a inflamação como elemento central.^19^

A aterosclerose inicia-se precocemente com o acúmulo de lipoproteínas modificadas no endotélio vascular, agravando-se com a idade. Nas mulheres, além dos efeitos metabólicos da menopausa, o envelhecimento contribui para a disfunção endotelial, principalmente pelo aumento da produção de espécies reativas de oxigênio (ROS) associadas à senescência. Citocinas e células inflamatórias exercem efeitos pró-aterogênicos ao comprometer a integridade da barreira endotelial, reduzir a vasodilatação, induzir a expressão de moléculas de adesão e quimiocinas e facilitar o recrutamento de leucócitos para as lesões ateroscleróticas.^19 - 21^

As variações biológicas entre mulheres e homens decorrem de diferenças na expressão gênica dos cromossomos sexuais, moduladas por influências hormonais e ambientais que resultam na apresentação de condições cardiovasculares associadas à regulação autonômica e à remodelação vascular e cardíaca.^18^

Fatores epigenéticos também desempenham papel relevante, em especial nas mulheres, cuja expressão é amplamente modulada pela menopausa. Essas alterações podem influenciar a transcrição e tradução gênica por meio de mecanismos, como metilação do DNA, modificação das histonas e regulação de RNAs não codificantes. Um fenômeno relacionado, a hematopoiese clonal de potencial indeterminado (CHIP, na sigla em inglês), refere-se à presença de mutações somáticas em células sanguíneas sem evolução para malignidades hematológicas.^22 , 23^ Essas mutações aumentam com a idade, são detectadas em cerca de 10% das pessoas acima dos 70 anos e estão associadas ao conceito de "inflamação do envelhecimento" ( *inflamm-aging* ), termo proposto por Franceschi *et al* . que explica o aumento do processo inflamatório pela imunossenescência.^22 , 23^

Portanto, as diferenças biológicas e a fisiopatologia subjacente específica do sexo feminino para as DCV não estão bem elucidadas, sendo necessárias novas pesquisas para o desenvolvimento de estratégias preventivas e terapêuticas mais eficazes.

### 3.2. Inflamação e Insuficiência Cardíaca na Mulher

O processo inflamatório local ou sistêmico participa na patogênese da IC, com remodelamento e fibrose cardíaca, além de anormalidades na macro e microcirculação. A inflamação crônica de baixa intensidade está associada a diferentes FR, como obesidade, resistência à insulina (RI), DM2, dislipidemia, doença renal crônica (DRC) e envelhecimento, além de levar à redução na produção do óxido nítrico (ON), alterando a fisiologia diastólica ventricular, promovendo miopatia atrial esquerda.^24 , 25^ O aumento da prevalência desses FR nas mulheres, associados aos efeitos da menopausa, parecem explicar a maior prevalência de ICFEp. A associação entre inflamação crônica e adiposidade na ICFEp decorre de evidências que apontam para o papel da liberação de substâncias produzidas pelo tecido adiposo visceral (adipocinas), particularmente localizado em volta do pericárdio e do epicárdio, levando a inflamação na microcirculação, fibrose cardíaca e alteração do enchimento diastólico fisiológico.^26 , 27^

Nas mulheres, a menopausa induz alterações na função cardíaca, decorrentes da queda da produção do estrogênio e consequente redução na produção do ON. Essa deficiência hormonal acarreta alterações na função diastólica e na fisiologia da circulação coronariana. Clinicamente, a ICFEp pode se apresentar com diferentes fenótipos, sendo dois deles comuns em mulheres: o fenótipo cardiometabólico, no qual obesidade e DM estão associadas à disfunção diastólica; e fibrilação atrial e miopatia atrial, presentes em mulheres idosas.^28 , 29^ Além disso, condições como obesidade, DM e distúrbios hipertensivos da gestação exacerbam o risco de ICFEp em mulheres, provavelmente devido a mecanismos inflamatórios associados. Apesar da maior prevalência dessas condições em mulheres, a maioria dos ensaios clínicos não as inclui na população estudada, o que leva ao menor conhecimento sobre a eficácia do tratamento no sexo feminino.^30^

Marcadores biológicos de inflamação, como a proteína C reativa ultrassensível (PCR-us) acima de 2 mg/L, têm se mostrado úteis na caracterização de indivíduos com ICFEp de fenótipo cardiometabólico, geralmente associado a pior prognóstico.^31^

A pré-eclâmpsia tem sido reconhecida como uma condição que leva a alterações na ativação imune e da inflamação sistêmica, com consequente disfunção endotelial e alteração na função cardíaca, podendo se manifestar ao longo das décadas seguintes e aumentar o risco de IC.^32 , 33^

Nas mulheres, as doenças autoimunes, em particular o lúpus eritematosos sistêmico, podem ocasionar inflamação do miocárdio, pericárdio, endocárdio valvar e da macro e microcirculação coronariana. Portanto, é fundamental que se compreendam as anormalidades fisiopatológicas e os aspectos clínicos da doença, a fim de estabelecer medidas de prevenção e detecção precoce das complicações cardiovasculares.^34^

Diversas evidências destacam o papel da IC nas mulheres. Estudos como o PURSUIT-HFpEF e análises do TOPCAT demonstraram que mulheres com ICFEp apresentam maior disfunção diastólica e piores desfechos clínicos em comparação com homens. Compreender os mecanismos específicos por sexo é essencial para avançar no manejo e na conduta da ICFEp.^30^

A análise *post-hoc* do estudo TOPCAT revelou diferenças significativas nas características basais entre homens e mulheres com ICFEp.^35^ Mulheres (55,5% da coorte) tinham menos comorbidades cardiovasculares (infarto prévio: 18,7% vs. 30,9% em homens, p < 0,001), pior função cardíaca (classe NYHA III/IV: 38,5% vs. 31,5%, p = 0,003) e maior fração de ejeção (63,3% vs. 58,9%, p = 0,001). As mulheres eram mais idosas, tinham maior prevalência de obesidade, fibrilação atrial e HAS, enquanto os homens apresentavam mais doença arterial coronariana e histórico de tabagismo. Apesar dessas diferenças, ambos os grupos tinham sintomas semelhantes de IC e uso comparável de medicamentos cardioprotetores.^35^ Os resultados mostraram que as mulheres tiveram menor mortalidade cardiovascular (8,9% vs. 14,8%, p < 0,001) e menos hospitalizações por IC (13,4% vs. 18,2%, p = 0,008). Após ajuste, o sexo feminino foi fator protetor para mortalidade cardiovascular (HR: 0,53; IC 95%: 0,40–0,73). Entretanto, as mulheres apresentaram pior qualidade de vida relacionada à saúde e maior limitação funcional. Em relação ao tratamento com espironolactona, houve redução da mortalidade global em mulheres (HR: 0,68; IC 95%: 0,48–0,96), mas não em homens (p para interação = 0,190), sugerindo possível benefício sexo-específico.

O estudo PURSUIT-HFpEF, um registro multicêntrico prospectivo no Leste Asiático, investigou diferenças sexuais na disfunção diastólica e desfechos clínicos em pacientes com ICFEp. Mulheres representaram 55,2% da coorte (481 de 871 pacientes).^36^ O estudo mostrou que mulheres apresentaram maior prevalência de disfunção diastólica (52,8% vs. 32,0% em homens, p < 0,001), independentemente de comorbidades como HAS e DM2. Anemia e obesidade foram fatores associados à disfunção diastólica apenas em mulheres. Apesar da pior função diastólica, mulheres tiveram taxa similar de eventos combinados (morte/hospitalização por IC) em análise não ajustada. Porém, após ajuste multivariável, o sexo feminino foi independentemente associado a maior risco de eventos clínicos (HR: 1,54; IC 95%: 1,14–2,07), principalmente por hospitalizações por IC.^30 , 36^

Ambos os estudos reforçam que, do ponto de vista metabólico, a ICFEp é uma condição distinta em mulheres, com:

1. Maior disfunção diastólica associada a fatores como obesidade e anemia, sendo importante o rastreio.
2. Melhor sobrevida global apesar da pior sintomatologia, possivelmente devido a menos comorbidades isquêmicas.
3. Resposta diferencial a terapias, como a espironolactona, que pode ser mais eficaz em mulheres.

### 3.3. Aspectos Hormonais na Inflamação

A saúde cardiovascular da mulher é influenciada por uma complexa interação de fatores hormonais e inflamatórios, que se manifestam de forma particular em diferentes fases da vida, como puberdade, gravidez e menopausa. As flutuações hormonais, especialmente os níveis de estrogênio e progesterona, desempenham um papel importante na manutenção da integridade vascular e na função cardíaca, tornando essencial a compreensão de como essas mudanças se relacionam com o aumento do risco de DCV.

Em mulheres pós-menopáusicas, o hipoestrogenismo promove alterações metabólicas importantes, como o aumento da adiposidade central, piora do metabolismo glicídico e elevação dos níveis de colesterol total, LDL-c e triglicerídeos, apolipoproteínas e lipoproteína a (Lp(a).^37 , 38^ Também se observa disfunção endotelial, com consequente elevação do risco de DIC.

O estrogênio exerce seus efeitos por meio da interação com diferentes receptores, como ERα, ERβ e GPER, ativando vias genômicas e não genômicas.^39^ Essa ação resulta na modulação da resposta inflamatória, reduzindo a produção de citocinas pró-inflamatórias, como a interleucina 6 (IL-6),^40^ e aumentando a expressão de citocinas anti-inflamatórias.^41^ Um mecanismo chave envolve a inibição da atividade do fator nuclear kappa B (NF-κB), reduzindo a transcrição de genes inflamatórios.^41^ Além disso, o estrogênio aumenta a produção de NO nas células endoteliais, o que contribui para a vasodilatação e inibe a expressão de moléculas de adesão, diminuindo a infiltração de leucócitos na parede vascular.^42^

A função endotelial é beneficiada pelo estrogênio ao induzir a expressão da enzima óxido nítrico sintase endotelial (eNOS), aumentando a produção de ON e promovendo a vasodilatação. Além disso, estimula a regeneração das células endoteliais e inibe a proliferação de células musculares lisas vasculares, prevenindo a progressão da aterosclerose.^42^

Os processos inflamatórios crônicos representam outro mecanismo subjacente crucial. Mulheres com ciclos irregulares frequentemente exibem estados pró-inflamatórios caracterizados por elevações de marcadores, como proteína C reativa (PCR), IL-6 e fator de necrose tumoral alfa (TNF-α). Conforme demonstrado, essa inflamação sistêmica atua como intermediária entre a disfunção menstrual e a gênese aterosclerótica.^18^

### 3.4. Inflamação na Cardio-oncologia

**Figura 3.1:**
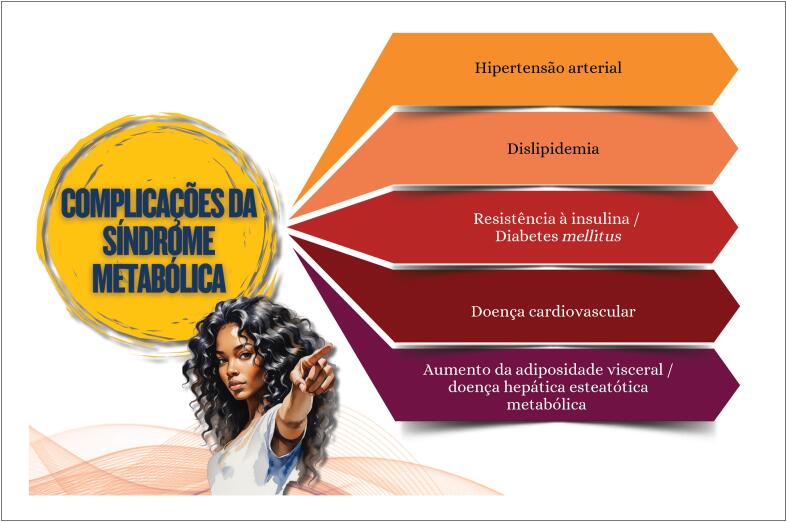
Complicações da síndrome metabólica.

Além dos FR tradicionais e a SM ( Figura 3.1 ), as mulheres com câncer possuem aumento do RCV devido a efeitos cardiotóxicos de terapias oncológicas, como antraciclina e trastuzumabe. Essas terapias podem levar a complicações como IC, IAM e AVC, mesmo anos após o término do tratamento oncológico.

A inflamação crônica sistêmica desempenha um papel central na interseção entre câncer e DCV. O microambiente inflamatório tumoral e os efeitos colaterais do tratamento oncológico contribuem de maneira sinérgica para disfunção endotelial, remodelamento vascular e progressão da aterosclerose, especialmente em mulheres. Por exemplo, a radioterapia torácica tem sido associada a alterações inflamatórias coronarianas detectadas por biomarcadores e pelo índice de atenuação da gordura perivascular (FAI), refletindo atividade inflamatória vascular persistente. Além disso, o uso prolongado de inibidores de aromatase em sobreviventes de câncer de mama foi associado à piora da função endotelial, com maior risco de eventos cardiovasculares.

Em mulheres em tratamento oncológico, é essencial uma abordagem multidisciplinar que inclua avaliação basal do risco cardíaco antes do início da terapia do câncer, que deve ser otimizada para minimizar a cardiotoxicidade, além de ferramentas para intervenções no estilo de vida e manutenção da vigilância cardiovascular de longo prazo. É importante também abordar as disparidades raciais e étnicas nos cuidados de saúde, pois mulheres negras e hispânicas apresentam maior risco de cardiotoxicidade e piores desfechos cardiovasculares em comparação com mulheres brancas devido a fatores socioeconômicos e acesso desigual aos cuidados de saúde.^43 , 44^

### 3.5. Inflamação na Gravidez e Pré-eclâmpsia

A pré-eclâmpsia é uma doença multissistêmica da gravidez, cuja fisiopatologia não é completamente compreendida. No entanto, acredita-se que uma perfusão placentária reduzida, resultante de invasão trofoblástica deficiente da parede uterina materna, aliada à secreção de citocinas inflamatórias e fatores angiogênicos, esteja por trás da doença. Esses fatores contribuem para disfunção endotelial, inflamação vascular e má perfusão materna. A disfunção endotelial tem sido apontada como um dos principais fenômenos responsáveis por pré-eclâmpsia e hipertensão gestacional.

A isquemia placentária está ligada à produção aumentada de fatores circulantes antiangiogênicos, como fator solúvel tipo tirosinaquinase-1 (sFlt-1) e endoglina solúvel (sEng), que causam disfunção endotelial generalizada, comprometimento da via do ON, estresse oxidativo, inflamação excessiva, desequilíbrio nos fatores angiogênicos e perda de reguladores endógenos protetores.^45^ Gestantes com FR associados a inflamação crônica (doenças autoimunes, obesidade, hipertensão pré-gestacional, DM e dislipidemia) têm maior probabilidade de desenvolver pré-eclâmpsia. É provável que mediadores inflamatórios possam ter efeitos autócrinos ou parácrinos locais, além de levarem à amplificação dos efeitos dos fatores antiangiogênicos.^46^

A gravidez, mesmo em condições normais, é um estado de estresse oxidativo devido ao aumento do metabolismo materno e da atividade placentária. Contudo, na pré-eclâmpsia, os mecanismos compensatórios falham, levando à produção aumentada de fatores patogênicos e à disfunção vascular subsequente.^47^ Diversos estudos observacionais e experimentais demonstraram uma associação entre inflamação e disfunção endotelial. Uma característica importante da inflamação sistêmica na pré-eclâmpsia é a predominância da imunidade do tipo Th1 e a ausência de tendência para Th2. Ao contrário, a gravidez normal é caracterizada por uma mudança para a imunidade do tipo Th2. Além disso, os níveis circulantes de citocinas pró-inflamatórias, como IL-6, TNF-α e as quimiocinas IL-8, IP-10 (proteína 10 induzível por interferon gama) e MCP-1 (proteína quimiotática de monócitos 1), estão elevados na pré-eclâmpsia.^45 , 46^

Nesse contexto, McCarthy *et al* . investigaram o papel da disfunção mitocondrial como facilitador de estresse oxidativo, inflamação, apoptose e alterações metabólicas – todos intermediários patogênicos fundamentais da pré-eclâmpsia.^47^

A obesidade aumenta o risco de pré-eclâmpsia. O tecido adiposo branco secreta mediadores pró-inflamatórios que contribuem para o estado inflamatório crônico e as complicações metabólicas da obesidade. A adiposidade visceral também está associada a FR metabólicos e complicações como DG e pré-eclâmpsia.^48^ O sFlt-1, forma solúvel do receptor do fator de crescimento endotelial vascular (VEGF), é sabidamente secretado pelos adipócitos em não gestantes. Huda *et al* .^48^ demonstraram que desregulação das vias inflamatórias ocorre predominantemente no tecido adiposo visceral, com a ativação de macrófagos e o aumento da expressão de TNF-α e IL-6 nesse tecido, mas não na gordura subcutânea, reforçando que, na pré-eclâmpsia, a desregulação das vias inflamatórias ocorre predominantemente no tecido adiposo visceral.

Uma metanálise conduzida por Guan *et al* .^49^ avaliou a relação entre biomarcadores pró- e anti-inflamatórios e suas mudanças dinâmicas ao longo da progressão da pré-eclâmpsia. As mulheres com pré-eclâmpsia apresentaram níveis significativamente maiores de PCR, IL-4, IL-6, IL-8, IL-10 e TNF-α. Os níveis de citocinas pró-inflamatórias foram mais altos do que os anti-inflamatórios. Mulheres com idade gestacional maior do que 34 semanas apresentaram níveis elevados de IL-6 e TNF-α. Pressão arterial sistólica mais alta foi associada a níveis maiores de IL-8, IL-10 e PCR. Esses achados sugerem que o desequilíbrio inflamatório é um FR independente para pré-eclâmpsia e a falha na autorregulação anti-inflamatória leva à progressão da doença.

Por fim, estudos recentes têm demonstrado que a neuroinflamação com a participação do sistema nervoso autônomo pode estar presente na indução e evolução das reações inflamatórias associadas a pré-eclâmpsia. Além disso, a regulação autonômica reduzida em pacientes com pré-eclâmpsia pode estar envolvida em uma maturação neurológica fetal tardia, com déficits cognitivos e distúrbios mentais. Esses achados revelam um elo entre inflamação materna na pré-eclâmpsia e seus impactos na saúde fetal e no neurodesenvolvimento.^50^

**Figura 3.2:**
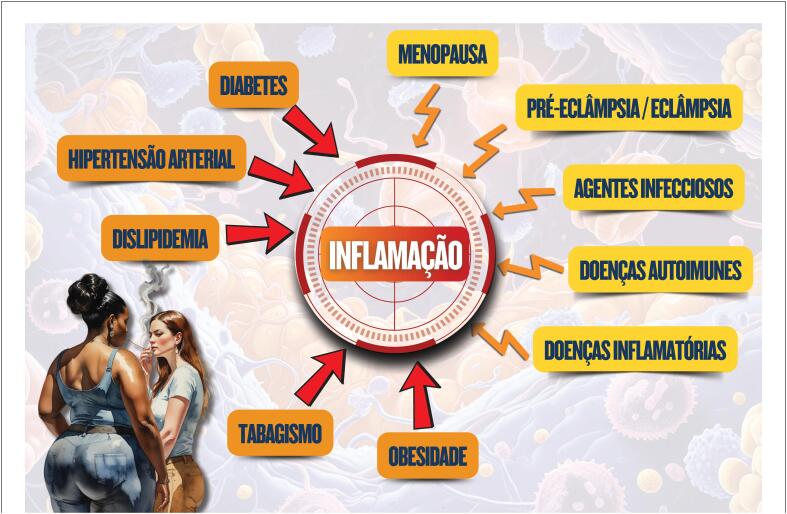
Fatores associados com inflamação nas mulheres.

Os fatores associados com inflamação nas mulheres são resumidos na Figura 3.2.

## 4. Implicação dos Esteroides Sexuais na Saúde Cardiometabólica

### 4.1. Origem e Metabolismo dos Esteroides Sexuais

Os principais esteroides sexuais que atuam no organismo da mulher são os estrogênios, a progesterona e a testosterona. Esses hormônios são produzidos pelo organismo feminino nos ovários, no córtex da suprarrenal e através de conversão periférica. Os esteroides sexuais possuem em comum um anel ciclopentanofenantreno derivado do colesterol e são divididos em três grupos principais, de acordo com o número de átomos de carbono que possuem. A série de 21 carbonos inclui os corticoides e os progestagênios, tendo o núcleo pregnano como estrutura básica. A série de 19 carbonos inclui todos os androgênios e é baseada no núcleo androstano, enquanto os estrogênios são esteroides de 18 carbonos baseados no núcleo estrano.^51^

A partir do colesterol, que é obtido diretamente do sangue circulante, a esteroidogênese origina os esteroides sexuais naturais.^51^ Fisiologicamente nas mitocôndrias, um sistema enzimático de hidroxilação e uma desmolase continuam o processo formando o primeiro esteroide, a pregnenolona. A partir dessa fase, a esteroidogênese pode seguir através da via delta 5, que predomina no folículo, para síntese de estrogênios, ou através da via delta 4, que predomina no corpo lúteo, para síntese de progesterona. A pregnenolona e a progesterona são convertidas em androgênios, androstenediona e testosterona, nas células da teca. A androstenediona, por ação das enzimas 17β-desidrogenases, pode ser convertida em testosterona e, por aromatização nas células da granulosa, resulta na formação de estrona e estradiol.^52^

Uma vez sintetizados, esses hormônios são transportados através do plasma ligados a SHBG ( *sexual-hormone-binding-globulin* ), CBG ( *cortisol-binding-globulin* ) ou albumina, sendo que uma pequena fração se encontra livre no plasma. Dessa forma, os esteroides sexuais ligam-se aos receptores hormonais presentes em diversos tecidos, exercendo seus diversos efeitos biológicos.^53^ Esses esteroides são metabolizados no fígado e excretados através da urina ou da bile.^51^

Os esteroides sexuais também podem ser extraídos de vegetais pela indústria farmacêutica, formando produtos para uso clínico, cujas moléculas têm estrutura idêntica à dos hormônios naturalmente produzidos pelo organismo feminino (isomoleculares), sendo, por isso, chamados de hormônios bioidênticos, cujos efeitos são iguais aos dos hormônios endógenos.

Os esteroides sexuais chamados sintéticos são produzidos em laboratório através de um processo que envolve várias etapas químicas e de engenharia farmacêutica, originando moléculas que possuem diferentes características e potências biológicas.^51 - 53^

### 4.2. Classificação Química dos Estrogênios

#### 4.2.1. Estrogênios Naturais

Os estrogênios naturais presentes na circulação são o estradiol, a estrona, o estriol e o estetrol. O estradiol é o principal e mais potente estrogênio secretado pelo ovário humano. O estradiol surge em grande parte da androstenediona, sendo a própria estrona secretada pelos ovários em quantidades diárias significativas. O estriol é o metabólito periférico da estrona e do estradiol e não um produto secretório do ovário. A formação de estriol é típica da "desintoxicação" metabólica geral, ou seja, a conversão de material biologicamente ativo em formas menos ativas. O estetrol é produzido apenas na vida intrauterina pelo fígado fetal.^51 - 53^

#### 4.2.2. Estrogênios Sintéticos

Os estrogênios sintéticos são classificados como esteroidais e não esteroidais. Seus principais representantes são:

- Esteroidais – etinilestradiol, mestranol, valerato de estradiol
- Não esteroidais – dietilestilbestrol, hexestrol, dienestrol

Desses, os de maior importância clínica atual são certamente o valerato de estradiol e o etinilestradiol.

O valerato de estradiol é um éster de estradiol, um pró-fármaco que é rapidamente metabolizado em 17β-estradiol e ácido valérico. A esterificação do estradiol visa melhorar a absorção e a biodisponibilidade após administração oral. Depois da absorção, os ésteres são clivados, resultando na liberação de estradiol endógeno ou 17β-estradiol. Os pró-fármacos ésteres de estradiol são, portanto, considerados formas bioidênticas de estradiol.^54.55^ Seu uso isolado ou em composições combinadas com progestagênios está relacionado principalmente à terapia de reposição hormonal da menopausa (THM) e à contracepção.

**Figura 4.1:**
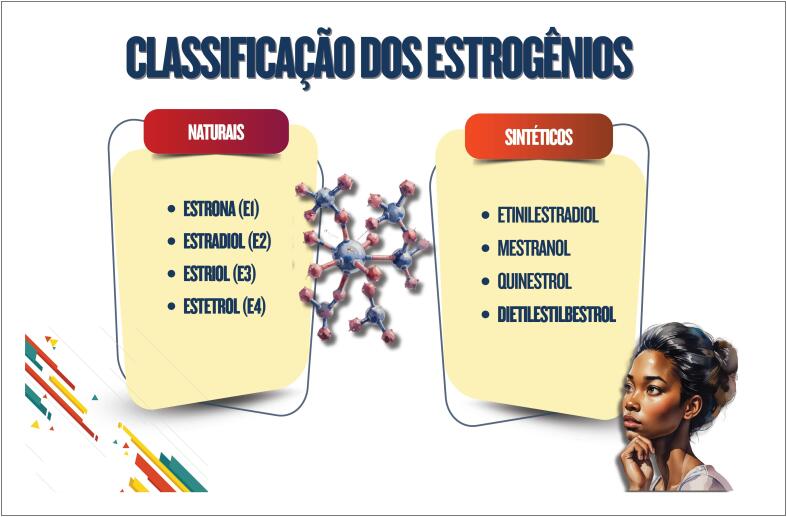
Classificação química dos estrogênios.^55^

O etinilestradiol, um agonista dos receptores de estrogênio, é mais resistente ao metabolismo, em comparação ao estradiol, e tem melhor biodisponibilidade quando usado por via oral. Essas diferenças favorecem o uso do etinilestradiol em pílulas anticoncepcionais combinadas, embora também resultem em aumento do risco de tromboembolismo e alguns outros efeitos adversos raros^55^ ( Figura 4.1 ).

### 4.3. Classificação Química dos Progestagênios

#### 4.3.1. Progesterona Natural

A progesterona originalmente secretada pelos ovários atua nos órgãos-alvo dependentes de hormônios, em especial o endométrio e o tecido mamário. Mas seus efeitos vão além, pois agem também na imunidade, no sistema nervoso central e no sistema cardiovascular, entre outros. Do ponto de vista fisiológico, a progesterona atua em tecidos que foram previamente impregnados com estradiol. Além disso, existe um estereoisômero da progesterona, a didrogesterona, disponível para uso oral.^56^

#### 4.3.2. Progestagênios

**Figura 4.2:**
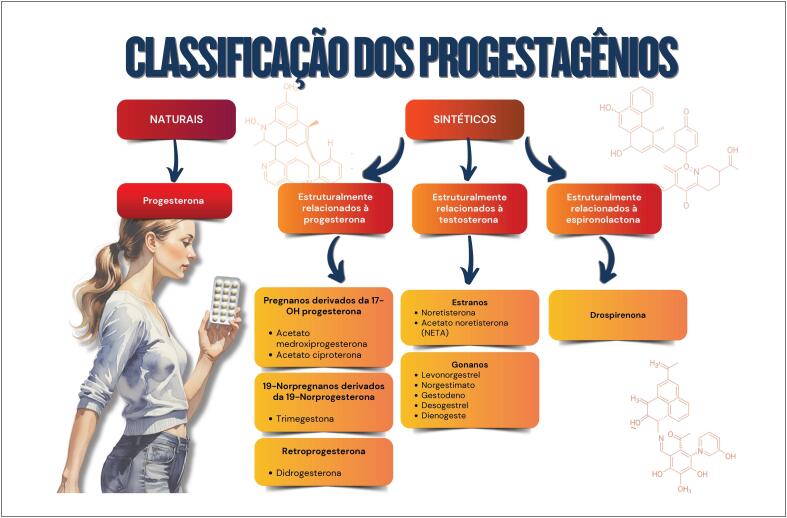
Classificação dos progestagênios.

Os progestagênios são um amplo grupo de moléculas sintéticas que diferem quanto à sua estrutura química e quanto à molécula a partir da qual são criados, o que lhes confere um perfil de efeito diferente, baseado na afinidade e potência em diferentes receptores de esteroides. Existem três grandes grupos: os derivados da testosterona (noretindrona, levonorgestrel, norgestimato, desogestrel, etonogestrel, gestodeno, dienogeste), os derivados da progesterona (medroxiprogesterona, ciproterona, megestrol) e os derivados da espironolactona (drospirenona) ( Figura 4.2 ).

**Figura 4.3:**
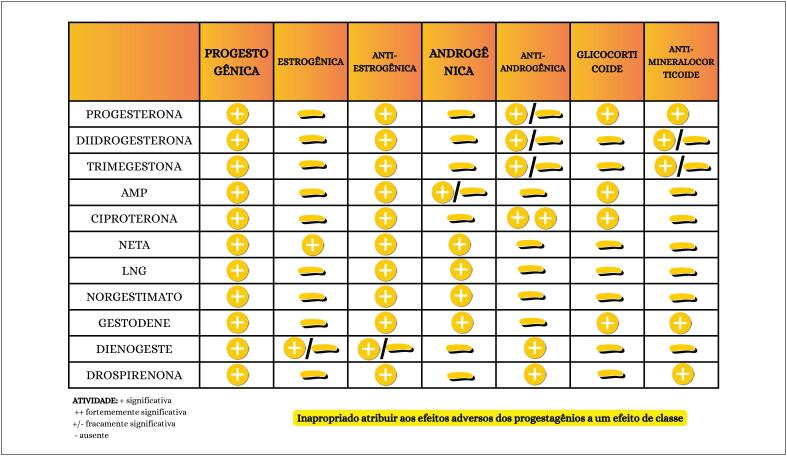
Efeitos biológicos da progesterona endógena e outros progestagênios. Adaptado de Kuhl et al. ^55^ AMP: acetato de medroxiprogesterona; NETA: acetato de noretisterona; LNG: levonorgestrel.

Os progestagênios são caracterizados pela alta afinidade pelo receptor de progesterona, mas podem, de acordo com sua origem, ter afinidade por outros receptores, levando a efeitos como atividade androgênica/antiandrogênica, antiestrogênica/estrogênica, antimineralocorticoide e glicocorticoide. Esse perfil farmacodinâmico dos progestagênios deve orientar sua seleção com base nos benefícios esperados, bem como no perfil de segurança e de efeitos indesejáveis^51 , 52 , 57^ ( Figura 4.3 ).

Os progestagênios possuem diferentes potências progestacionais, determinadas por sua capacidade de transformar o endométrio animal. Considerando-se como 1 a potência da progesterona, a ordem decrescente de potência progestacional dos progestagênios é a seguinte: desogestrel, levonorgestrel, nomegestrol, medroxiprogesterona, noretisterona, drospirenona.^58^

Do ponto de vista farmacocinético, a progesterona e os progestagênios diferem quanto às apresentações farmacêuticas e vias de administração. Por exemplo, progesterona micronizada pode ser usada tanto por via oral quanto vaginal. Por via vaginal apresenta boa absorção, inclusive com menores flutuações na sua concentração plasmática do que por via oral.^52^

Os progestagênios também diferem na sua ligação às proteínas plasmáticas, sendo que a progesterona micronizada se liga à albumina e à CBG, mas não à SHBG. Enquanto a noretindrona, o desogestrel e o norgestrel se ligam à albumina e à SHBG, o acetato de medroxiprogesterona se liga principalmente à albumina.^59^

As diferenças na ligação às proteínas plasmáticas somadas à depuração e à associação ou não com estrogênios determinam diferenças na meia-vida plasmática dos progestagênios. A maioria deles é de administração diária.

### 4.4. Indicações Comuns de Terapêuticas com Estrogênios, Progestagênios e Testosterona na Mulher

#### 4.4.1. Síndrome dos Ovários Policísticos

A SOP é uma indicação frequente de terapêutica com hormônios esteroides. Trata-se de distúrbio ginecológico associado a distúrbios endócrinos frequentes nas mulheres em idade reprodutiva. Além disso, distúrbios metabólicos estão presentes, elevando o RCV.^60^

As mulheres com SOP podem desenvolver RI e dislipidemia, que contribuem para o desenvolvimento de DCV, como aterosclerose, HAS e/ou IAM.^61^ Essas alterações metabólicas são impulsionadas pelo excesso de androgênios e o ganho de peso, que podem promover acúmulo de gordura visceral e processo inflamatório crônico. Esses fatores combinados criam um ambiente metabólico propício ao desenvolvimento de DCV mesmo em mulheres jovens.^62 - 64^

O manejo do RCV na SOP requer abordagens multidisciplinares, incluindo modificações no estilo de vida, como dieta equilibrada e exercícios físicos, além de intervenções farmacológicas quando necessário. Os contraceptivos orais combinados (COC) são muito utilizados no tratamento da SOP para controle das alterações menstruais e do hirsutismo, sendo muito eficazes por reduzirem os níveis circulantes de andrógenos, regular os ciclos e proteger o endométrio. No entanto, esse tratamento não melhora a RI, podendo até piorá-la, dependendo do tipo de progestagênio utilizado.^60^ Sensibilizadores da ação da insulina, como metformina ou alternativas como pioglitazona^65^ e o mio-inositol,^61 , 66^ diminuem a RI, melhorando a função ovariana. As estatinas podem ser indicadas para controle da dislipidemia e os anti-hipertensivos são essenciais para mulheres com HAS. Os tratamentos para obesidade podem ser empregados, como o uso dos análogos do peptídeo semelhante ao glucagon 1 (GLP-1: liraglutida e semaglutida) e outros.^67^ A identificação precoce dessas alterações metabólicas e o tratamento adequado são fundamentais para reduzir o RCV em mulheres com SOP.

#### 4.4.2. Contracepção Hormonal

Os esteroides sexuais são muito utilizados na contracepção hormonal. Os COCs contêm dois tipos de hormônios (estrogênio + progestagênio) em diferentes formulações.^68^ O componente estrogênico fornece estabilidade endometrial cíclica e potencializa o efeito supressor do progestagênio sobre o eixo hipotálamo-hipófise-ovariano. O componente estrogênico mais comumente utilizado é o etinilestradiol, em doses atuais entre 15μg e 30μg. Novos COCs com estrogênios naturais – estradiol (17β-estradiol e valerato de estradiol) e mais recentemente o estetrol – surgiram como alternativas, com potencial para menor impacto hepático e cardiovascular.^69^

**Figura 4.4:**
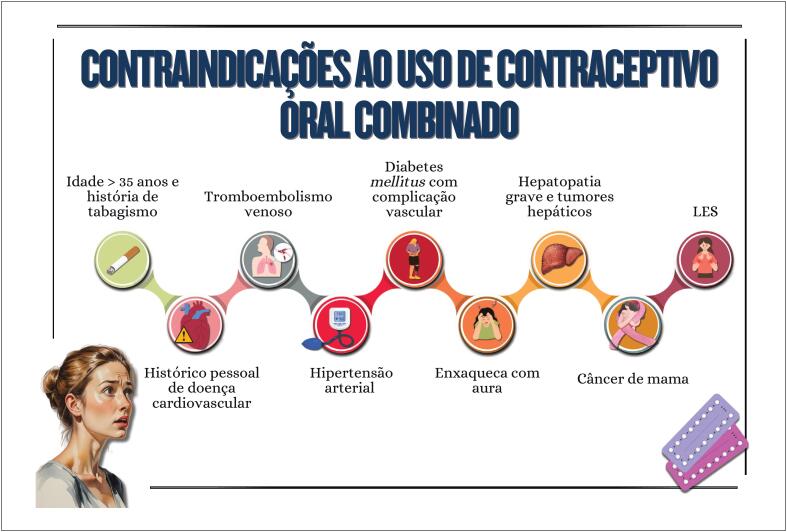
Contraindicações ao uso de contraceptivo oral combinado. LES: lúpus eritematoso sistêmico.

Nos diferentes COCs, esses estrogênios são combinados a um progestagênio. O componente progestagênico exerce seu principal efeito contraceptivo suprimindo a secreção do hormônio luteinizante (LH) e a ovulação. Nas formulações atuais, vários progestagênios são empregados. São progestagênios sintéticos responsáveis por inibir a ovulação/gravidez, tendo os mais modernos sido desenvolvidos para causarem menos efeitos secundários/adversos. As taxas de gravidez no primeiro ano de uso de um COC variam, sendo de 0,3% com uso consistente e correto. Com uso típico, pode chegar a 9%, demonstrando a eficácia do método durante o uso real,^70^ reforçando a importância do aconselhamento contraceptivo. Os COCs não estão recomendados para mulheres com idade acima de 35 anos e histórico pessoal de tabagismo, DCV, tromboembolismo venoso (TEV), HAS, DM com complicação vascular, enxaqueca com sinais neurológicos, hepatopatia grave, tumores hepáticos, câncer de mama e lúpus eritematoso sistêmico. A segurança do método contraceptivo está muito bem estabelecida pelos Critérios de Elegibilidade Médica para Uso de Contraceptivos^71^ ( Figura 4.4 ).

Os contraceptivos hormonais somente de progestagênios contêm apenas um progestagênio sintético, como desogestrel, norestisterona ou drospirenona. Contraceptivos somente de progestagênio de longa duração também podem ser usados sob a forma de implantes na região subdérmica do antebraço, sendo que, no Brasil, o único implante (etonogestrel) aprovado para contracepção é o Implanon NXT^®^, que apresenta alta eficácia contraceptiva por 3 anos.^72^ O sistema intrauterino liberador de levonorgestrel (DIU-LNG) é um DIU hormonal que libera 20 µg de levonorgestrel por dia com ação intrauterina local e alta eficácia contraceptiva por 8 anos. Também há o DIU de liberação de 8 µg de levonorgestrel e duração de 3 anos. Os contraceptivos somente de progestagênio de longa duração apresentam a vantagem de ter alta eficácia por tempo prolongado com taxas de gravidez de menos de 1% ao ano sem depender do uso correto e da motivação da usuária.^72^

#### 4.4.3. Terapia Hormonal da Menopausa: Regimes, Doses e Vias, com Foco na Saúde Cardiovascular

Em mulheres, a flutuação hormonal que se inicia na transição menopausal seguida pela redução gradual e irreversível da produção do estrogênio na menopausa, embora fisiológica, cria uma "janela de vulnerabilidade" e marca ponto de inflexão no risco cardiometabólico, com impacto negativo na saúde vascular, no metabolismo glicídico e lipídico e na distribuição da gordura corporal.^73 , 74^ Nesse contexto, a THM, especialmente o estrogênio, pode interferir nesse risco, desde que prescrita com critério e individualização. A THM deve ser sempre associada a medidas integrais à saúde, com estratégias sobre estilo de vida, dieta e exercícios físicos.^75^

A THM é o tratamento mais efetivo no alívio dos sintomas vasomotores (SVM) e geniturinários e prevenção da perda óssea em mulheres na peri- e pós-menopausa.^76^ Deve ser iniciada quando os sintomas da menopausa começam a interferir na vida diária. Entretanto, para mulheres com insuficiência ovariana prematura (menopausa < 40 anos) ou menopausa precoce (entre 40 e 45 anos), recomenda-se iniciar a THM o mais cedo possível, para reduzir o risco à saúde a longo prazo.^77^ Os efeitos cardiovasculares da THM são fortemente influenciados pelo tipo e dose utilizados, via de administração e momento do início em relação à menopausa ("janela de oportunidade"), sugerindo maiores benefícios e menores taxas de efeitos adversos com o início da THM em até 10 anos pós-menopausa.^77^ Os fatores que determinam a escolha do tipo e da dose da THM são: preferência da paciente, presença ou ausência do útero, necessidade contraceptiva, intensidade dos sintomas e comorbidades associadas.^75^

Os regimes terapêuticos incluem o estrogênio isolado (para mulheres histerectomizadas) e a terapia combinada de estrogênio associado ao progestagênio (mulheres com útero).^76^ No regime combinado, o uso contínuo é indicado para mulheres há mais de 12 meses em amenorreia na pós-menopausa, enquanto o sequencial é mais indicado na perimenopausa. A THM deve ser prescrita na menor dose eficaz para controle dos sintomas.^78^ O estrogênio mais utilizado na THM é o 17β-estradiol, podendo ser administrado por via oral, transdérmica (adesivo, gel, *spray* ) ou vaginal.^75^ Para atingir o alívio adequado dos SVM e proteção óssea, são necessárias, em geral, baixas doses de estradiol (aproximadamente 1 mg de estradiol oral ou equivalentes em outras vias).^75 , 76^ Em mulheres saudáveis sem fatores de risco cardiovascular (FRCV), a THM pode ser prescrita por qualquer via, incluindo a oral.^75^ Contudo, diferentemente da via oral, a transdérmica evita o metabolismo hepático da primeira passagem, resultando em menor impacto nos fatores de coagulação, triglicerídeos e PCR, o que pode reduzir o risco de TEV e eventos cardiovasculares, sendo preferível em mulheres com FR, como obesidade, SM, tabagismo, hipertensão e risco de TEV.^79^

A THM com hormônios "bioidênticos manipulados" não é recomendada devido a preocupações com a qualidade, regulação, segurança, eficácia e falta de padronização dos produtos utilizados.^75 , 76^

A testosterona na pós-menopausa tem como única indicação o tratamento do transtorno do desejo sexual hipoativo, excluindo-se outras causas. Embora dados sobre os efeitos da terapia androgênica na saúde cardiovascular de mulheres na pós-menopausa com desejo sexual hipoativo ainda sejam limitados, evidências indicam que, em doses fisiológicas, a testosterona via transdérmica não aumenta significativamente o RCV.^80^

Abordagem individualizada com decisão baseada na melhor evidência deve ser utilizada. Manutenção da THM e o não início da THM em mulheres ≥ 60 anos parece associar-se a melhor perfil de risco-benefício para eventos cardiovasculares.^75^ A via transdérmica de estrogênio em baixas doses combinada a progestagênios com perfil seguro representa opção favorável quanto ao RCV.^79^

### 4.5. Riscos: Efeitos Metabólicos, Tromboembolismo e Câncer de Mama

#### 4.5.1. Efeitos Metabólicos

A THM diminui a gordura visceral, a RI e a relação LDL-c:HDL-c, porém com pequeno aumento dos triglicerídeos quando usado por via oral.^81 , 82^

Uma revisão também confirmou menores níveis de glicemia, insulinemia e RI entre usuárias de THM.^83^ O estudo *Women’s Health Initiative* (WHI) encontrou menor risco significativo de DM entre as usuárias de THM em comparação àquelas em uso do placebo.^84^

Há evidências de que o tipo de progestagênio empregado na THM pode interferir nos efeitos metabólicos. Sabe-se que medroxiprogesterona tem mais efeitos glicocorticoides do que noretisterona ou progesterona.^85^ Estudos sugerem que progesterona e didrogesterona interferem menos nos benefícios promovidos pelos estrogênios,^86 , 87^ enquanto noretisterona, dependendo da dose, pode levar a alguma perda desses benefícios.^88^

#### 4.5.2. Tromboembolismo

Etinilestradiol, estrogênio sintético amplamente utilizado em associação a um progestagênio em COCs, associa-se a aumento de risco de TEV.^89^ Esse risco é influenciado por fatores como a dose de estrogênio e o tipo de progestagênio presente na formulação. Os COCs contendo levonorgestrel (progestagênio de segunda geração) estão associados a um risco menor de TEV em comparação àqueles que contêm desogestrel, gestodeno, drospirenona ou ciproterona (progestagênios de terceira e quarta geração).

Quando o COC contém estrogênios naturais ao invés do etinilestradiol, o risco apresenta incremento menor.^89^ Além disso, o etinilestradiol dos COCs pode exacerbar a produção de angiotensinogênio hepático, que, por sua vez, causa elevação da PA pelo sistema renina-angiotensina-aldosterona.

Quando administrados por via oral, podem levar a aumento do risco de trombose venosa, *odds ratio* 1,58 (1,52–1,64), enquanto a via transdérmica não se associa a aumento do risco, *odds ratio* 0,93 (0,87–1,01).^90^ Além disso, metanálise recente concluiu que THM com combinação de estrogênios conjugados equinos e progestagênios aumentou a PA sistólica e o risco de hipertensão, enquanto outras formulações, como estradiol oral ou transdérmico com progestagênio, estradiol isolado e tibolona, não apresentaram efeitos significativos sobre a PA, mostrando, portanto, que tais efeitos podem ser influenciados por diferentes vias e formulações.^91^

#### 4.5.3. Câncer de Mama

Sabe-se que o câncer de mama é uma neoplasia hormônio-dependente, sendo os efeitos proliferativos dos esteroides sexuais no tecido mamário conhecidos.^92^

A THM pode se associar a aumento de risco para o desenvolvimento de câncer de mama. O estudo WHI encontrou que a THM com estrogênios conjugados e acetato de medroxiprogesterona se associou a acréscimo de 8 casos extras de câncer de mama a cada 10 mil mulheres-ano.^93^

Entretanto, os efeitos são diferentes a depender do progestagênio presente na formulação. Estudos observacionais não mostraram aumento de risco quando a associação na THM era com progesterona micronizada^94^ ou didrogesterona.^95^

Dessa forma, diferentes formulações e doses da THM também parecem ter diferentes efeitos no risco de câncer de mama.

## 5. Indicadores de Saúde Cardiometabólica

### 5.1. Avaliação de Fatores de Risco Reprodutivos com Implicações na Saúde Cardiometabólica

Este capítulo contempla os FR com relevância no perfil cardiometabólico das mulheres nas fases da puberdade, período pré-gestacional, gestacional e pós-gestacional, além da transição da menopausa, que serão abordados mais detalhadamente nos capítulos 7 e 9.

#### 5.1.1. Puberdade e Período Pré-gestacional

Sugere-se que a avaliação do ciclo menstrual possa ser usada como um sinal vital adicional na investigação do estado geral de saúde das mulheres.^96^ Nesse cenário, a idade da menarca (precoce ou tardia), as irregularidades do ciclo menstrual de causas endócrinas e a SOP têm sido associadas a maior risco futuro de DCV, em especial a doença aterosclerótica.^5 , 97^

##### 5.1.1.1. Idade da Menarca

O estudo CARDIA ( *Coronary Artery Risk Development in Young Adults* ), que iniciou o seguimento de 2.788 mulheres de 18-30 anos em 1985-1986 e acompanhou-as durante 35 anos (idades entre 50 anos e 65 anos), observou que a menarca precoce se associou tardiamente a níveis adversos de glicídios e lipídios e que cada ano de precocidade da menarca em relação à média de 12 anos associou-se a maior índice de massa corpórea (IMC) e adiposidade visceral.^98^

Outros estudos mostraram que a menarca precoce está associada à elevação de glicose, insulina, PA e gordura corporal e ao maior risco futuro de DCV.^5^

Em estudo de coorte de 648 mulheres estratificadas segundo a idade da menarca (≤ 10, 11, 12, 13, 14, ≥ 15 anos) e utilizando a média de 12 anos como referência, observou-se que menarca precoce ou tardia se associou a maior risco de eventos cardiovasculares futuros, representados por morte por todas as causas, IAM não fatal, AVC não fatal ou hospitalização por IC.^99^

Em uma coorte de 1,2 milhão de mulheres, com média de idade de 56±5 anos, sem doença cardíaca prévia e seguidas por 12 anos, aquelas com menarca precoce (≤ 10 anos) e tardia (≥ 17 anos) apresentaram risco mais elevado de DIC, AVC e HAS.^100^

Entretanto, uma metanálise de 12 estudos de coorte realizados até 2018, com 2.341.769 participantes e 79.363 óbitos, demonstrou que para cada aumento de um ano na idade da menarca há uma redução do risco relativo de mortalidade por todas as causas, de mortalidade cardiovascular por cardiopatia isquêmica e por AVC,^101^ evidenciando a necessidade da realização de mais estudos sobre o impacto da idade da menarca na DCV futura.

##### 5.1.1.2. Características do Ciclo Menstrual

Uma revisão sistemática e metanálise de estudos observacionais realizados até 2022 analisou a associação de oligomenorreia e irregularidade menstrual com o RCV, observando que essas alterações estão associadas a DCV, DIC e IAM, mas não a AVC como documentado em mulheres com SOP^5 , 97 , 98 , 102^ (vide capítulo 7).

#### 5.1.2. Gestação e Período Pós-parto

A gravidez é um período de inúmeras transformações fisiológicas, hormonais e metabólicas, fundamentais para garantir o adequado desenvolvimento fetal e a adaptação do organismo materno às novas demandas. Ganho de peso anormal e alterações no perfil lipídico e glicêmico, porém, podem estar associados a desfechos adversos na gestação e no pós-parto, tanto para a mulher como para o concepto.

##### 5.1.2.1. Alterações de Peso na Gestação

Existem evidências de que tanto o ganho de peso gestacional (GPG) baixo quanto o excessivo estão associados a desfechos fetais e neonatais negativos.^103^

A obesidade pré-gestacional favorece o risco de hipertensão gestacional e DG, cesárea e alto peso ao nascer, sendo também reconhecida como FR significativo para aborto espontâneo, parto prematuro, distúrbios metabólicos que complicam a gravidez e maiores taxas de partos anormais, natimortos e morte neonatal. Por outro lado, a desnutrição também pode contribuir para o menor peso neonatal, anomalias placentárias e suas complicações, maiores taxas de partos operatórios e maior mortalidade fetal e neonatal.^103 , 104^

**Figura 5.1:**
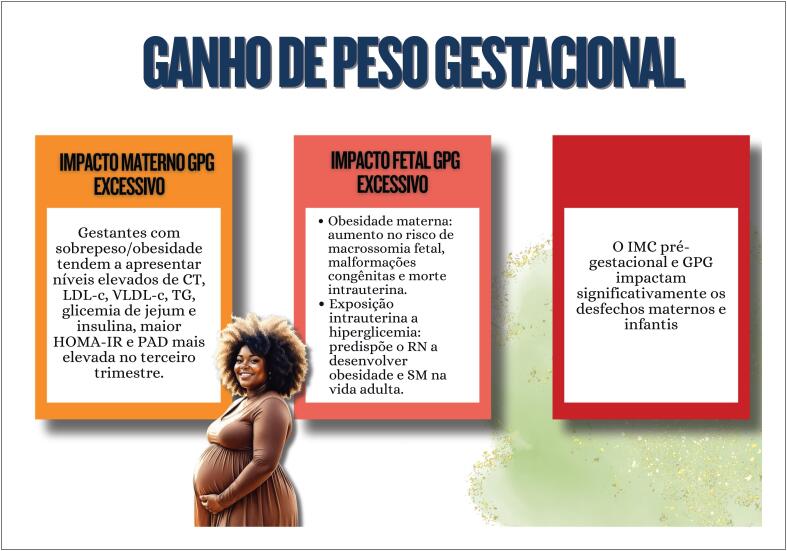
Implicações para a saúde cardiometabólica do ganho de peso na gestação. GPG: ganho de peso gestacional; CT: colesterol total, LDL-c: colesterol de lipoproteína de baixa densidade; VLDL-c: colesterol de lipoproteína de muito baixa densidade; TG: triglicerídeos; HOMA-IR: homeostasis model assessment of insulin resistance; PAD: pressão arterial diastólica; RN: recém-nascido; SM: síndrome metabólica; IMC: índice de massa corpórea.

As diretrizes recomendam valores do GPG, mas análises de estudos mais recentes sugerem que esse ganho seja personalizado considerando as três classes de obesidade^103^ ( Tabela 5.1 e Figura 5.1 ). Ver detalhes no capítulo 7.

**Tabela 5.1:** Ganho de peso na gravidez de acordo com a recomendação das diretrizes do Instituto de Medicina 2009

IMC	< 18,5	18,5–24,9	25,0–29,9	> 30
Ganho de peso (kg)	12,5–18,0	11,5–18,0	7–11,5	5–9
Ganho de peso (kg) semanal nos 2º e 3º trimestres	0,44–0,58	0,35–0,50	0,23–0,33	0,17–0,27

IMC: índice de massa corpórea.

##### 5.1.2.2. Disglicemia Gestacional

A gravidez é uma condição de RI por si só, potencialmente agravada pelo aumento da RI pré-gravidez em mulheres com obesidade. Há um aumento de 40% a 50% na RI durante a gravidez e, portanto, aumento no risco de DG.^105^

Mulheres com intolerância à glicose na gestação correm risco de desfechos gestacionais adversos, mesmo que não tenham desenvolvido DG.

A fisiopatologia subjacente primária que leva à hiperglicemia está relacionada à presença de RI, deficiência de insulina ou fisiopatologia mista. Estudos mostraram que a intolerância à glicose na gestação resistente à insulina é um subtipo de alto risco para resultados adversos na gravidez, como peso ao nascer grande para a idade gestacional, admissão na unidade de terapia intensiva neonatal, hipertensão arterial gestacional e parto cesáreo.^106^

A intolerância à glicose na gestação confere um risco aumentado de diabetes no futuro. Portanto, o reconhecimento de portadoras de intolerância à glicose na gestação pode beneficiá-las ao possibilitar um melhor rastreamento e prevenção do DM (vide dados complementares no capítulo 7).

##### 5.1.2.3. Dislipidemia Gestacional

Evidências recentes mostram que o aumento de colesterol, triglicerídeos e seus metabólitos associados à disfunção cardiometabólica parece ter consequências vasculares maternas e fetais significativas.^107^

Historicamente, a dislipidemia na gravidez tem sido considerada fisiológica e, portanto, sem relevância clínica. Esse aumento fisiológico desempenha um papel essencial durante a gravidez. No entanto, níveis elevados de lipídios em mulheres predispostas ou com formas familiares de hiperlipidemia podem acarretar risco aumentado de complicações materno-fetais. A hiperlipidemia durante a gravidez está associada a pré-eclâmpsia, parto prematuro e DG, e os filhos dessas mães apresentam propensão à formação aumentada de estrias de gordura nos vasos e maior risco de aterosclerose progressiva.^108^

##### 5.1.2.4. Comportamento Pós-parto

No puerpério, a recuperação ponderal é frequentemente incompleta e a recuperação das funções metabólicas nem sempre ocorre de forma plena. A retenção de peso pós-gestacional constitui FR para HAS, dislipidemia e SM. Além disso, estudos indicam que mulheres que apresentaram pré-eclâmpsia possuem pior perfil cardiometabólico até um ano após o parto.

A lactação, embora represente um fator de proteção metabólica por estimular o gasto energético e melhorar o controle glicêmico e lipídico, depende de fatores emocionais, sociais e de suporte adequado. A ausência de orientação especializada nesse período pode favorecer a persistência de quadros como obesidade, RI e dislipidemia, além de aumentar o risco de SM e HAS.^104^

#### 5.1.3. Transição da Menopausa

A falta do estradiol observada na menopausa determina inúmeras modificações metabólicas, hormonais, inflamatórias e da função endotelial, cuja associação favorece a rápida progressão do processo aterosclerótico em todos os territórios arteriais. Desse modo, mulheres a partir da quinta década passam a apresentar RCV mais elevado que os homens.^109 , 110^

O manuseio adequado (prevenção primária, detecção precoce e tratamento adequado) dos FR que contribuem ao longo da vida para o desenvolvimento da aterosclerose é crucial para o retardo do adoecimento cardiovascular em mulheres na peri- e pós-menopausa, bem como para a prevenção primária e secundária dos eventos que levam à mortalidade cardiovascular.^111^ Essa orientação para a prevenção cardiovascular deve ocorrer em todas as etapas de vida anteriores à menopausa, ganhando ainda maior importância a partir do climatério.^109 - 111^

##### 5.1.3.1. Idade da Menopausa

Na grande maioria das mulheres (90%), a menopausa natural ocorre em torno dos 51 anos (variando entre 45 anos e 55 anos).^110 - 112^ Em 5% das mulheres, a menopausa pode ocorrer de forma espontânea entre 40 anos e 45 anos (menopausa precoce) e em 1% pode acontecer antes dos 40 anos, caracterizando a insuficiência ovariana prematura.^110 , 112^ Mulheres submetidas a ooforectomia bilateral e a tratamentos quimio- ou radioterápicos podem evoluir para menopausa em consequência desses tratamentos, independentemente da idade em que são realizados.^109 , 110 , 112^

O RCV torna-se ainda mais acentuado em mulheres que iniciam a menopausa precocemente (de forma natural ou secundária a algum tratamento), nas que apresentam SVM de moderada a grave intensidade e nas que fazem uso de THM.^109 , 110^ Na menopausa precoce, cada ano antecipado em relação à menopausa natural aumenta em 3% o risco de DCV (DIC, AVC e mortalidade cardiovascular).^110 , 112^

Esse risco mais elevado parece associado a uma maior propensão desse grupo a apresentar perfil lipídico mais aterogênico, maior RI, HAS, maior risco de SM, bem como piora da função endotelial e dos marcadores inflamatórios, quando comparado a mulheres que apresentam menopausa natural.^110 , 112^

##### 5.1.3.2. Sintomas Vasomotores

Os SVM da menopausa, caracterizados por ondas de calor e suor geralmente noturnos, podem estar presentes em até 80% das mulheres e durar 7-9 anos. Porém, quando aparecem mais precocemente, sua duração pode ser mais longa.^109 , 110^ A intensidade dos SVM varia entre leve, moderada e grave e seu diagnóstico associa-se a prejuízo na qualidade de vida e ao aumento da utilização dos serviços de saúde.^113 - 115^

Inúmeros estudos têm demonstrado que mulheres com SVM de grave intensidade, quando comparadas a mulheres sem SVM ou com SVM leves, apresentam pior perfil cardiometabólico, maior hiperatividade simpática, pior função endotelial e maior incidência de aterosclerose subclínica.^113 , 115^

##### 5.1.3.3. Terapia Hormonal da Menopausa

Vários ensaios clínicos demonstraram que a THM com estrógenos aumenta o RCV (cardiopatia isquêmica, AVC, tromboembolismo) em mulheres pós-menopáusicas, parecendo haver atenuação desse risco quando utilizada em mulheres mais jovens (50 a 59 anos), ou seja, que se encontram no início da menopausa ou dentro de 10 anos do seu diagnóstico. O risco é descrito como atenuado também com o uso de baixas doses e outras formas de administração que não a oral, notadamente a transdérmica.^109 , 110^

As diretrizes atuais estabelecem que a THM tem indicação baseada em evidências científicas apenas para o tratamento dos SVM em mulheres que não apresentam contraindicação, não devendo ser usada naquelas com DCV estabelecida ou com RCV elevado.^78 , 109 , 110^

#### 5.1.4. Saúde Metabólica Pós-menopausa

A menopausa está associada a alterações hormonais que resultam em uma alteração no equilíbrio entre estrogênio e testosterona. Os ovários, apesar de cessarem a produção do estrogênio, ainda produzem testosterona a uma taxa menor, o que leva a seu excesso relativo. A testosterona está associada ao aumento da gordura visceral e da PA em mulheres na pós-menopausa.^116^

A presença de flutuações hormonais e alterações fisiológicas relacionadas ao envelhecimento configuram um estado metabólico comprometido, caracterizado por RI, aumento de gordura corporal total, sarcopenia e acúmulo de gordura abdominal.^117^

##### 5.1.4.1. Mudanças Metabólicas na Menopausa

A RI é definida como a resposta inadequada à insulina nos tecidos (adiposo, músculo esquelético), sistema nervoso central e fígado, sendo um dos principais fatores que levam à hiperglicemia e ao DM2, juntamente com a secreção prejudicada de insulina.

Evidências epidemiológicas sugerem proteção significativa contra a RI em mulheres na pré-menopausa, sendo essas mais sensíveis à insulina em comparação aos homens. Essa vantagem metabólica, porém, desaparece gradualmente após a menopausa ou quando a RI progride para hiperglicemia e DM. Portanto, a menopausa está associada a um maior risco de intolerância à glicose, aumento da PA e dos níveis de triglicerídeos, assim como do risco de SM, com consequente aceleração do RCV.^117 , 118^

##### 5.1.4.2. Diabetes Mellitus

As alterações descritas tornam as mulheres propensas ao desenvolvimento de DM2. Evidências sugerem que mulheres que apresentam SVM na menopausa têm maior risco de desenvolver DM2 em relação às que não apresentam tais sintomas. Em uma análise de dados do WHI, o risco de desenvolvimento de DM2 foi 18% maior em mulheres com SVM, aumentando paralelamente à gravidade desses sintomas.^119^

Mulheres que desenvolvem DM antes dos 20 anos tendem a ter menopausa precoce, enquanto a menopausa é retardada em mulheres com DM2 de início tardio.^119^

Uma publicação do estudo SWAN acompanhado por 20 anos concluiu que mulheres com múltiplos sintomas físicos, psicológicos e os da menopausa de intensidade moderada a grave tiveram início mais precoce de DM e SM.^119^

A THM foi associada à redução do risco de desenvolvimento de DM2 em mulheres anteriormente sem a doença e, nas portadoras de DM2, à melhora do controle glicêmico.^117^

##### 5.1.4.3. Dislipidemia

O estrogênio exerce um papel protetor no sistema cardiovascular, sendo sua produção ovariana realizada por meio da utilização do LDL-c como substrato. Na menopausa, o LDL-c circulante não pode ser utilizado para sintetizar estrogênio, resultando assim na redução da produção desse hormônio e no discreto aumento dos níveis séricos de LDL-c. O tamanho e a densidade das partículas de LDL-c também mudam com a menopausa. Estudos mostram que os níveis de partículas de LDL-c pequenas e densas aumentam de 10-13% em mulheres na pré-menopausa para 30-49% após a menopausa.^37^

A deficiência de estrogênio na menopausa prejudica as funções hepáticas e intestinais relacionadas ao metabolismo e transporte de lipídios, o que pode aumentar os níveis de lipídios plasmáticos e acelerar a progressão da aterosclerose.^120^

O estudo SWAN sugere que a função antiaterogênica do colesterol de lipoproteína de alta densidade (HDL-c), ou seja, a capacidade de promover o transporte reverso de colesterol, pode diminuir durante a menopausa, cursando, portanto, com níveis menos elevados de HDL-c associados a aterosclerose.^109^

### 5.2. Indicadores Antropométricos

A saúde cardiometabólica é um pilar importante do bem-estar, englobando a integridade do sistema cardiovascular e do metabolismo. Nas mulheres, esse aspecto é influenciado por mudanças hormonais, eventos reprodutivos e fatores biológicos que se manifestam ao longo do ciclo de vida, da infância à velhice.^110^

Os indicadores antropométricos são medidas corporais que fornecem informações sobre a composição e distribuição de gordura, refletindo o risco cardiometabólico. Simples e acessíveis, essas medidas são fundamentais para o rastreamento em larga escala populacional.^110 , 121^ Portanto, devem ser realizadas rotineiramente, com atenção às mudanças ao longo do ciclo de vida.

#### 5.2.1. Índice de Massa Corpórea

O IMC, calculado como peso (kg)/altura² (m), classifica o estado nutricional: < 18,5 (baixo peso); 18,5-24,9 (normal); 25-29,9 (sobrepeso); e ≥ 30 (obesidade). Um IMC elevado está associado a HAS, dislipidemia e DM. No entanto, sua limitação é não diferenciar massa magra de gordura nem avaliar a distribuição adiposa, o que reduz sua precisão em mulheres idosas ou atletas.^122 , 123^

#### 5.2.2. Circunferência da Cintura – População Brasileira

A circunferência da cintura mede a gordura visceral, um preditor mais robusto de risco cardiometabólico que o IMC. A gordura visceral está associada à RI e à inflamação crônica, fatores centrais na patogênese da DCV.^124 , 125^ Em mulheres, circunferência da cintura > 88 cm indica alto risco, conforme diretrizes da Organização Mundial da Saúde.

#### 5.2.3. Razão Cintura/Quadril

A razão cintura/quadril (RCQ) compara a circunferência da cintura à circunferência do quadril, refletindo a distribuição de gordura. Uma RCQ > 0,85 em mulheres sugere acúmulo central de gordura, associado a maior risco de SM. Estudos indicam que a RCQ é superior ao IMC na predição de eventos cardiovasculares, especialmente após a menopausa.^124 , 126^

#### 5.2.4. Percentual de Gordura Corporal

O percentual de gordura corporal é um parâmetro importante para avaliar o risco cardiometabólico, especialmente porque as mulheres tendem a apresentar maior proporção de gordura subcutânea em comparação aos homens. Alterações hormonais, como as ocorridas na puberdade, gestação e menopausa, impactam diretamente na distribuição e no acúmulo de gordura, podendo aumentar o risco de RI, dislipidemias e inflamações crônicas.^126 , 127^

#### 5.2.5. Razão Cintura/Estatura

A razão cintura/estatura (RCE) destaca-se como uma medida preditora de adiposidade central e risco cardiometabólico. Valores acima de 0,5 são fortemente associados a maior risco de HAS, DM2 e eventos cardiovasculares. Esse marcador é particularmente relevante na transição da menopausa, quando há tendência ao acúmulo de gordura abdominal.^128 , 129^

### 5.3. Biomarcadores

#### 5.3.1. Perfil Lipídico (Colesterol Total e Frações LDL e HDL; Triglicerídeos)

O LDL-c promove inflamação vascular e deposição de colesterol na íntima arterial, enquanto o HDL-c disfuncional (comum em mulheres com RI ou SM) perde capacidade antioxidante e de transporte reverso, favorecendo acúmulo de placas.^130^ Triglicerídeo elevado (> 150 mg/dL) associa-se a partículas de LDL-c pequenas e densas, além de partículas semelhantes a remanescentes, que intensificam instabilidade de placas.^131^ Como comentado anteriormente neste capítulo, a menopausa traz alterações no perfil metabólico pelo declínio estrogênico, elevando o LDL-c (∼ 10-15%) e reduzindo o HDL-c, assim como sua função protetora, agravando a dislipidemia, especialmente em mulheres com obesidade visceral ou DM.^73^ Portanto, estratégias não farmacológicas, como dieta mediterrânea e exercícios aeróbicos, melhoram a funcionalidade do HDL-c e modulam os triglicerídeos.

#### 5.3.2. Glicemia e Hemoglobina Glicada

A hiperglicemia crônica e a elevação da hemoglobina glicada (HbA1c) na mulher aumentam o RCV por mecanismos como glicação de proteínas vasculares, disfunção endotelial e ativação de vias inflamatórias, que aceleram a aterosclerose e a instabilidade de placas.^132^ A RI, comum em condições como SOP e DG prévia, induz dislipidemia aterogênica (HDL-c reduzido, triglicerídeo elevado) e estresse oxidativo, exacerbando o dano vascular.^133^

Na pós-menopausa, o declínio estrogênico reduz a sensibilidade à insulina e amplia a disfunção metabólica, correlacionando-se com maior rigidez arterial e inflamação sistêmica.^134^ Conforme demonstrado nos estudos SUSTAIN e EMPA-REG OUTCOME,^135 , 136^ as diretrizes recomendam para a maioria das mulheres com DM2: HbA1c <7%; e fármacos com benefício cardiovascular comprovado para prevenção secundária, como os agonistas do GLP-1 (ex.: semaglutida) e os inibidores do cotransportador de sódio-glicose tipo 2 (SGLT2, ex.: empagliflozina), que reduzem eventos cardiovasculares e hospitalizações por IC. A avaliação de aterosclerose subclínica (ex.: escore de cálcio coronariano) e o manejo agressivo de comorbidades (HAS, dislipidemia) são essenciais. Estratégias não farmacológicas, como dieta mediterrânea e exercícios aeróbicos, modulam a inflamação e melhoram a sensibilidade insulínica.

#### 5.3.3. Proteína C Reativa

A elevação da PCR-us na mulher está associada ao aumento do RCV devido à inflamação vascular crônica, que promove disfunção endotelial, instabilidade de placas ateroscleróticas e ativação trombótica.^137^ Fatores hormonais modulam essa relação, como na menopausa, em que, com o declínio estrogênico, há elevação da PCR-us (∼ 15-25%), que se correlaciona com maior rigidez arterial e resposta inflamatória exacerbada.^73^

Comorbidades, como doenças autoimunes (ex.: lúpus eritematoso sistêmico), e a THM oral podem aumentar a PCR-us.^138^ O uso de estatinas (ex.: rosuvastatina) em pacientes com PCR-us elevada reduziu eventos cardiovasculares independentemente do LDL-c, conforme evidenciado no estudo JUPITER.^137^ Estratégias adjuvantes incluem dieta e exercício aeróbico, que modulam a inflamação, em especial em mulheres pós-menopáusicas.^139^ Portanto, a interpretação da PCR-us deve integrar o contexto hormonal, comorbidades e estilo de vida, direcionando o tratamento para mitigar o RCV.

#### 5.3.4. Fibrinogênio

O fibrinogênio é uma glicoproteína plasmática solúvel, sintetizada pelo fígado, envolvida na agregação plaquetária, lesão endotelial e viscosidade plasmática e desempenha um papel central na formação de trombos. É também uma proteína de fase aguda da inflamação induzida principalmente pela IL-6.^140 , 141^

As mulheres apresentam níveis circulantes mais elevados de fibrinogênio independentemente de idade e níveis mais elevados de fibrinogênio funcional, conforme determinado por estimativas tromboelastográficas da contribuição do fibrinogênio para a resistência do coágulo.^141^

As diferenças funcionais e dos níveis circulantes de fibrinogênio entre homens e mulheres definem um comportamento denominado "dimorfismo sexual". Essas diferenças estão associadas aos hormônios sexuais, sendo o estradiol um importante mediador mecanicista na indução da coagulação.^141 - 143^

Dados epidemiológicos demonstraram o importante papel preditivo do fibrinogênio na DIC^140^ e um estudo publicado em 2025 com 5.690 participantes mostrou uma relação entre altos níveis de fibrinogênio e mortalidade por toda as causas, sugerindo que o fibrinogênio seja um potencial biomarcador para risco de mortalidade.^143 , 144^

#### 5.3.5. Homocisteína

Níveis elevados de homocisteína aceleram o desenvolvimento da placa aterosclerótica nas artérias por meio de mecanismos como aumento do estresse oxidativo, oxidação de LDL-c, depleção de ON, disfunção endotelial, processos inflamatórios, modificações epigenéticas e regulação de microRNA.^145^

Modificações dietéticas e de estilo de vida, atividade física regular, cessação do tabagismo e redução do consumo de álcool são essenciais para o controle da hiperhomocisteinemia.^145^

#### 5.3.6. Adipocinas

Adipocinas são peptídeos secretados pelos adipócitos, sendo leptina e adiponectina as mais conhecidas e estudadas. Seus níveis fisiológicos normais são essenciais para manter a função cardiovascular adequada.

A leptina promove a saciedade e regula o gasto energético. Situações de hiperleptinemia estão presentes na obesidade e DM2, refletindo um estado de resistência à leptina associado a processos aterogênicos, disfunção endotelial, inflamação crônica de baixo grau e disfunção vascular. Existem claras diferenças entre os sexos nos níveis circulantes de leptina, com as mulheres apresentando níveis 3 a 4 vezes maiores em relação aos homens.^146 , 147^

A adiponectina exerce efeitos anti-inflamatórios, antiaterogênicos e insulinotrópicos. Sua ação inclui a melhora da sensibilidade à insulina, a redução do estresse oxidativo e a modulação da resposta inflamatória endotelial. Níveis séricos de adiponectina têm sido inversamente associados ao risco de DCV, sendo considerado um biomarcador de proteção cardiovascular. As mulheres têm níveis significativamente mais altos de adiponectina circulante total em comparação com os homens em populações saudáveis. As diferenças sexuais nos níveis de adiponectina não são bem caracterizadas, mas as diferenças na distribuição regional de gordura (subcutânea vs. visceral) entre homens e mulheres podem contribuir para as diferenças sexuais.^146 , 147^

**Figura 5.2:**
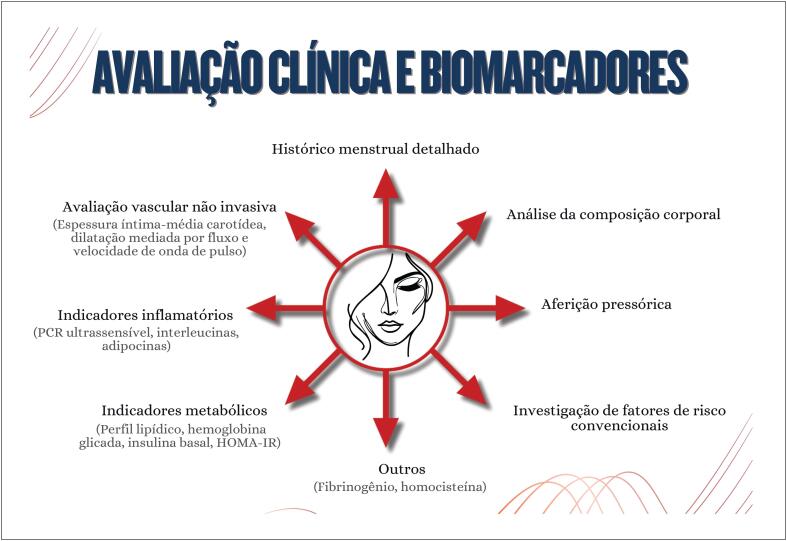
Utilização dos biomarcadores para avaliação clínica. HOMA-IR: homeostasis model assessment of insulin resistance; PCR: proteína C reativa.

A utilização dos biomarcadores para avaliação clínica pode ser resumida na Figura 5.2.

## 6. Cardiometabolismo no Período Infanto-juvenil

A idade de início da puberdade tem implicações clínicas e epidemiológicas significativas, pois variações no *timing* do evento estão associadas a diversos desfechos metabólicos e cardiovasculares. A menarca, definida como a primeira menstruação, sinaliza a maturação do eixo hipotálamo-hipófise-gonadal, marcando o início da capacidade reprodutiva feminina. Estudos apontam uma tendência secular de antecipação da menarca, com redução de cerca de três meses por década nos últimos 50 anos, reflexo de melhorias nutricionais e mudanças no estilo de vida.^148^ Atualmente, a idade média da menarca situa-se entre 12 anos e 13 anos em países desenvolvidos, enquanto em regiões de baixa renda esse evento ocorre mais tardiamente.^149^ Compreender os determinantes dessa variabilidade e seus impactos metabólicos é de fundamental importância para a saúde cardiovascular.^150^

### 6.1. Definições e Critérios Diagnósticos

Considera-se **puberdade precoce** quando o início do desenvolvimento dos caracteres sexuais ocorre antes dos 8 anos de idade nas meninas. A **menarca precoce** é definida como o início da menstruação antes dos 9,5 anos de idade. A menarca ocorre em média dois anos depois do aparecimento das mamas sendo que a menarca precoce apresenta maior prevalência em meninas negras e hispânicas. Contudo, o consenso brasileiro enfatiza a avaliação clínica individual, levando em conta o contexto de cada paciente.^151^ Por outro lado, a **menarca tardia** ocorre quando o primeiro sangramento menstrual é observado após os 15 anos de idade ou na ausência de menarca até três anos após o desenvolvimento sexual secundário completo. Suas causas incluem alterações hipotalâmicas, hipofisárias e gonadais, bem como déficits energéticos significativos.^152^

#### 6.1.1. Fisiopatologia

A puberdade depende da ativação do eixo hipotálamo-hipófise-gonadal, regulado pela liberação pulsátil do hormônio liberador de gonadotrofinas (GnRH). Esse processo estimula a produção de LH e hormônio folículo-estimulante (FSH) pela hipófise, que, por sua vez, ativa os ovários para a secreção de estrogênios.

Na **puberdade precoce** , a ativação ocorre de forma antecipada, geralmente associada à alta sensibilidade hipotalâmica às adipocinas, em particular à leptina. Esse hormônio, produzido pelo tecido adiposo, sinaliza ao hipotálamo a existência de reservas energéticas adequadas para a reprodução. Em meninas obesas, níveis elevados de leptina podem exacerbar esse gatilho, acelerando o início da puberdade. Além disso, a exposição precoce a disruptores endócrinos, como bisfenol A e ftalatos, pode interferir nos sinais hormonais naturais e precipitar a ativação do eixo reprodutivo.

Já na **puberdade tardia** , a ativação do eixo é frequentemente retardada por déficits energéticos crônicos comuns em atletas ou em pacientes com distúrbios alimentares. O baixo estoque energético reduz a secreção de leptina e outras adipocinas, prejudicando a sinalização para o hipotálamo. Em alguns casos, há resistência aos sinais metabólicos de insulina e leptina ou alterações na sinalização da kisspeptina, um neuropeptídeo hipotalâmico essencial para a secreção de GnRH.^151^

Esses mecanismos ilustram como o estado nutricional e a composição corporal influenciam diretamente o momento da menarca e suas repercussões metabólicas. Por exemplo, a obesidade pode induzir disfunções metabólicas que aceleram a maturação sexual, enquanto déficits calóricos intensos, observados nas atletas ou meninas com distúrbios alimentares como anorexia nervosa, retardam esse processo.

#### 6.1.2. Menarca Precoce, Menarca Tardia e Risco Cardiometabólico

**Figura 6.1:**
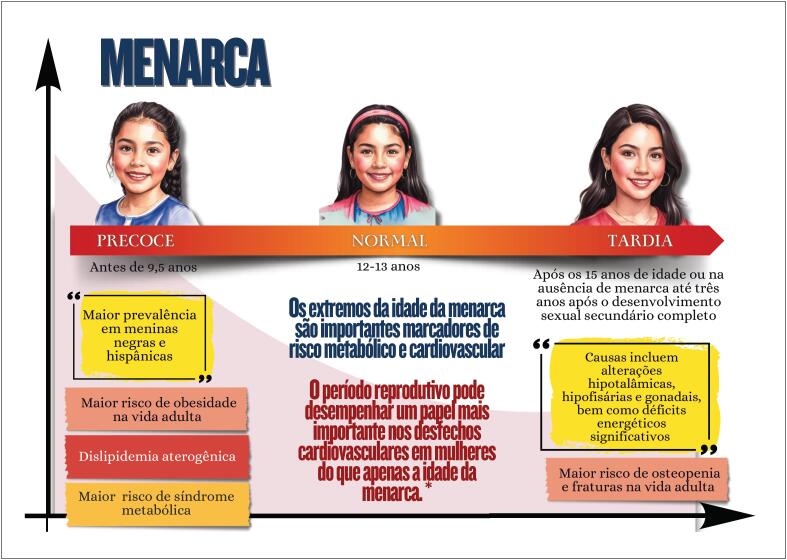
Associação do risco cardiovascular com idade da menarca. Os extremos de idade da menarca associam-se a um risco futuro de disfunção cardiometabólica e doença cardiovascular, devendo ser pensados como potencializadores de risco cardiovascular.

A menarca precoce está fortemente associada a desfechos metabólicos adversos. Um dos principais impactos é o aumento do IMC e da adiposidade central, com risco de obesidade na vida adulta cerca de 30-60% superior em comparação a mulheres com menarca em idade média^150^ ( Figura 6.1 ).

Fonte: Dastmalchi e Gulati.^154^

Outro desdobramento relevante é a RI, frequentemente identificada nesse perfil, sendo caracterizada por níveis elevados de insulina de jejum e alterações precoces no metabolismo da glicose, muitas vezes independentes do IMC.

Mudanças desfavoráveis no perfil lipídico também são comuns, incluindo elevação de triglicerídeos e LDL-c, bem como redução do HDL-c. Esses fatores configuram um quadro de dislipidemia aterogênica, que amplifica o RCV.

Além disso, há aumento de 1,5 a 2,5 vezes no risco de SM, associada a uma combinação de obesidade central, HAS, RI e dislipidemia.^100^ Mesmo após ajustes para fatores confundidores, como nível socioeconômico, o impacto metabólico da menarca precoce mantém relevância clínica.

Na **menarca tardia** , os desfechos metabólicos são mais heterogêneos. De modo geral, observa-se menor IMC e adiposidade nas pacientes, o que reflete um perfil metabólico mais favorável. No entanto, em casos relacionados a déficits energéticos, como nos distúrbios alimentares, a desnutrição torna-se um fator crítico, influenciando negativamente o metabolismo e a saúde óssea^152^ ( Figura 6.1 ).

A sensibilidade à insulina é melhor em mulheres com menarca tardia em comparação àquelas com menarca precoce.

A saúde óssea frequentemente é comprometida na menarca tardia, com risco aumentado de osteopenia e fraturas na vida adulta. A densidade mineral óssea reduzida nesses casos ressalta a necessidade de intervenções preventivas e monitoramento contínuo.^100 , 152^

Por fim, os extremos de idade da menarca correlacionam-se a um padrão de RCV em formato de "U". Na menarca precoce, observa-se aumento da PA sistólica e diastólica, espessamento da camada íntima-média carotídea e maior predisposição à aterosclerose subclínica. Em contraste, na menarca tardia, desregulações hormonais e nutricionais elevam a probabilidade de complicações tardias.^153^

### 6.2. Irregularidades Menstruais e Risco Cardiometabólico

Os distúrbios menstruais relacionados a causas hormonais e, portanto, excluídas as causas estruturais como miomatose e pólipos, constituem manifestações comuns durante a vida reprodutiva feminina, acometendo cerca de 14-25% das mulheres em idade fértil, com picos de incidência nos extremos da vida reprodutiva, como menarca e perimenopausa.^155^ Essas alterações, classificadas como oligomenorreia, polimenorreia, amenorreia, menorragia e metrorragia, vão além das alterações ginecológicas e emergem como importantes indicadores de vulnerabilidade cardiometabólica. Investigações contemporâneas evidenciam correlações robustas entre anomalias do ciclo menstrual e o desenvolvimento de DCV, ressaltando a necessidade de uma abordagem integrativa na avaliação dessas pacientes.^156^

#### 6.2.1. Mecanismos Fisiopatológicos

A interrelação entre ciclos menstruais irregulares e riscos cardiometabólicos envolve múltiplos processos fisiopatológicos interconectados. O eixo hipotálamo-hipófise-ovariano exerce função primordial nessa associação, pois desequilíbrios hormonais impactam simultaneamente as funções reprodutiva e cardiovascular. As oscilações nos níveis de estrogênio e progesterona modulam diretamente a sensibilidade insulínica, o metabolismo de lipídios, a funcionalidade endotelial e a homeostase pressórica.^44 , 155^

A RI desponta como elemento central na fisiopatologia de condições como a SOP, promovendo tanto irregularidades menstruais quanto dislipidemia, HAS e adiposidade visceral. Estudos de acompanhamento longitudinal revelam que mulheres com RI apresentam risco substancialmente elevado para DM2 e eventos cardiovasculares ao longo da vida.^156^

#### 6.2.2. Avaliação Clínica e Biomarcadores

A avaliação de mulheres com irregularidades menstruais requer uma abordagem abrangente dos FRCV. O histórico menstrual detalhado constitui componente essencial na estratificação de risco, necessitando complementação com análise da composição corporal, aferição pressórica e investigação de FR convencionais.^153 , 157 , 158^

#### 6.2.3. Intervenções Terapêuticas

O manejo de adolescentes com irregularidades menstruais e risco cardiometabólico elevado demanda estratégias multidimensionais. As modificações no estilo de vida constituem o alicerce terapêutico, incluindo atividade física regular, padrões alimentares cardioprotetores, controle ponderal e cessação do tabagismo.^157^ As intervenções farmacológicas requerem personalização conforme o perfil individual de risco.

Apesar dos avanços significativos, persistem importantes lacunas no conhecimento sobre a relação entre irregularidades menstruais e riscos cardiometabólicos.

Na prática clínica, recomendam-se incorporar a avaliação menstrual detalhada na estratificação de RCV, implementar rastreamento cardiometabólico precoce em adolescentes com irregularidades menstruais persistentes, adotar abordagem multidisciplinar no manejo e diagnóstico precoce de condições, como SOP frequentemente diagnosticada no início da puberdade,^109 , 159 - 161^ e desenvolver programas educacionais sobre a interrelação entre saúde reprodutiva e cardiovascular.^44 , 162 , 163^ Essas alterações estão detalhadas no capítulo 7.

### 6.3. Obesidade, Transtornos Alimentares e Risco Cardiovascular: Impactos a Longo Prazo

A obesidade na infância e adolescência está associada a uma série de desfechos adversos tanto físicos quanto psicológicos. Do ponto de vista físico, observa-se um aumento significativo no risco de desenvolvimento de comorbidades crônicas, como DCV, HAS, dislipidemia, RI, DM2 e doença hepática esteatótica metabólica (DHEM).^164^ Psicologicamente, a obesidade está relacionada à deterioração da saúde emocional, incluindo maior prevalência de estresse, sintomas depressivos e baixa autoestima.^165^

#### 6.3.1. Transtornos Alimentares e Obesidade

A relação entre obesidade e transtornos alimentares é reconhecidamente bidirecional. Crianças e adolescentes com obesidade apresentam maior risco de desenvolver transtornos alimentares, em especial bulimia nervosa e transtorno da compulsão alimentar periódica. Esses transtornos, por sua vez, podem agravar as consequências físicas e psicológicas já associadas à obesidade, estabelecendo um ciclo patológico de ganho ponderal e comportamentos alimentares desordenados.^165^

A *American Academy of Pediatrics* destaca a importância da triagem precoce de comportamentos alimentares desordenados e da implementação de intervenções oportunas. Tais medidas podem promover significativa melhora nos desfechos clínicos.^164^ Atitudes alimentares disfuncionais identificadas durante a infância intermediária correlacionam-se com o surgimento de obesidade e complicações cardiometabólicas no futuro, justificando a necessidade de monitoramento precoce e longitudinal.^166^

#### 6.3.2. Fatores Hereditários e Prognóstico dos Transtornos Alimentares

Diversos fatores genéticos e hereditários influenciam o risco e o prognóstico dos transtornos alimentares na infância e adolescência.^167^ Os principais incluem:

1. *Loci* Genéticos e Herdabilidade^167^
  Estudos apontam *loci* específicos associados à anorexia nervosa (região 1p33–36) e à bulimia nervosa (10p14), com herdabilidade estimada entre 48% e 74%.
2. Genes Específicos^167^
  Mutações em genes como *ESRRA, HDAC4* , *AGRP, GHRL* e *BDNF* têm sido associadas à regulação do apetite e ao risco aumentado de desenvolvimento de transtornos alimentares.
3. Histórico Familiar^168^
  Antecedentes familiares de transtornos alimentares ou distúrbios psiquiátricos elevam significativamente o risco individual, como evidenciado por estudos com gêmeos monozigóticos.
4. Transtornos Psiquiátricos Comórbidos^167^
  Existe sobreposição genética entre os transtornos alimentares e outras condições psiquiátricas, como transtorno obsessivo-compulsivo e depressão, agravando o curso clínico.
5. Doenças Autoimunes e Autoinflamatórias
  Evidências também apontam que a presença de doenças autoimunes no histórico familiar pode contribuir para o risco de desenvolvimento de transtornos alimentares, sugerindo um possível envolvimento do sistema imunológico.

#### 6.3.3. Consequências Futuras dos Transtornos Alimentares

Transtornos alimentares que se iniciam na infância apresentam repercussões duradouras. Estudos longitudinais indicam que padrões alimentares inadequados precoces estão associados à ocorrência de transtornos diagnosticáveis na adolescência.^169^ Por exemplo, a hiperfagia infantil está ligada ao risco aumentado de compulsão alimentar e transtorno da compulsão alimentar periódica, enquanto a seletividade alimentar extrema e a subalimentação persistente elevam o risco de anorexia nervosa.^170^

As consequências estendem-se para além da adolescência. Indivíduos acometidos por transtornos alimentares na juventude apresentam risco elevado de desenvolver transtornos psiquiátricos, como depressão, transtornos de ansiedade e abuso de substâncias ilícitas na fase adulta jovem. Comportamentos autolesivos também são mais prevalentes nesse grupo. Ademais, sintomas alimentares precoces estão associados a desfechos de peso desfavoráveis, incluindo obesidade ou magreza extrema, e à persistência dos comportamentos alimentares desordenados até a idade adulta.

A atuação precoce associada a estratégias terapêuticas, como terapia cognitivo-comportamental, intervenções familiares e tratamento das comorbidades psiquiátricas, é fortemente recomendada.^171^

#### 6.3.4. Obesidade Infantil e Desfechos Cardiovasculares

Conforme destacado pela *American Heart Association* , crianças com obesidade severa com frequência apresentam múltiplos FRCV simultaneamente, como hipertensão, dislipidemia e RI, desde tenra idade.^171^

A obesidade infantil tem implicações substanciais para a saúde cardiovascular na vida adulta. Trata-se de um fator fortemente associado ao desenvolvimento de hipertensão, dislipidemia, RI, DM2 e DHEM. Esses fatores persistem e evoluem, elevando a morbimortalidade cardiovascular.^171^

A revisão de Sommer e Twig aponta aumento da incidência de DCV, como IM e AVC, entre aqueles que foram obesos na infância e adolescência.^172^ Manter a obesidade desde a infância até a idade adulta está associada a RCV significativamente maior em comparação com indivíduos que normalizaram o peso.^172^

O clássico *Bogalusa Heart Study* demonstrou que essas alterações fisiológicas estão relacionadas ao aumento da espessura médio-intimal da artéria carótida e rigidez arterial, que são marcadores de aterosclerose subclínica.^173^

Além disso, alterações estruturais cardíacas, como aumento da massa ventricular esquerda e hipertrofia miocárdica, são comuns nesses indivíduos e correlacionam-se com piores desfechos cardiovasculares futuros.

#### 6.3.5. Transtornos Alimentares e Risco Cardiovascular

Transtornos alimentares iniciados na infância, como a anorexia nervosa, também implicam em alterações cardiovasculares persistentes. Mesmo após a recuperação clínica, indivíduos podem apresentar rigidez aumentada da artéria carótida, menor distensibilidade da aorta, disfunção endotelial e hiperatividade vagal.^173^

Além disso, atitudes alimentares desadaptativas durante a infância estão associadas ao desenvolvimento de obesidade e HAS na adolescência, ambos FRCV.^171^

A *American Heart Association* destaca que crianças obesas com comportamentos alimentares desordenados tendem a apresentar aumento da massa ventricular esquerda, rigidez arterial e PA elevada, fatores que persistem até a idade adulta e contribuem para o desenvolvimento de aterosclerose e outras DCV crônicas.^171^

## 7. *Continuum* Cardiometabólico e Idade Reprodutiva

O continuum cardiometabólico refere-se a um processo progressivo de alterações metabólicas e cardiovasculares interligadas, iniciado frequentemente na infância. Nas mulheres, as fases da vida reprodutiva – menarca, gravidez, puerpério - e menopausa, influenciam diretamente esse risco. A gestação é um estado fisiológico que impõe profundas alterações cardiometabólicas, necessárias para sustentar o crescimento fetal e adaptar o organismo materno às novas demandas. Entre essas mudanças, destacam-se o aumento do débito cardíaco, expansão do volume plasmático, redução da resistência vascular sistêmica, alterações no perfil lipídico, maior resistência periférica à insulina, ativação inflamatória e remodelamento vascular. Essas adaptações podem descompensar condições clínicas prévias ou revelar vulnerabilidades cardiometabólicas latentes, com implicações tanto para a saúde materna imediata quanto para o RCV futuro.^174 - 178^

Reconhecer essa relação nas diferentes fases da vida da mulher é fundamental para desenvolver estratégias de prevenção e rastreamento ao longo da vida.

**Figura 7.1:**
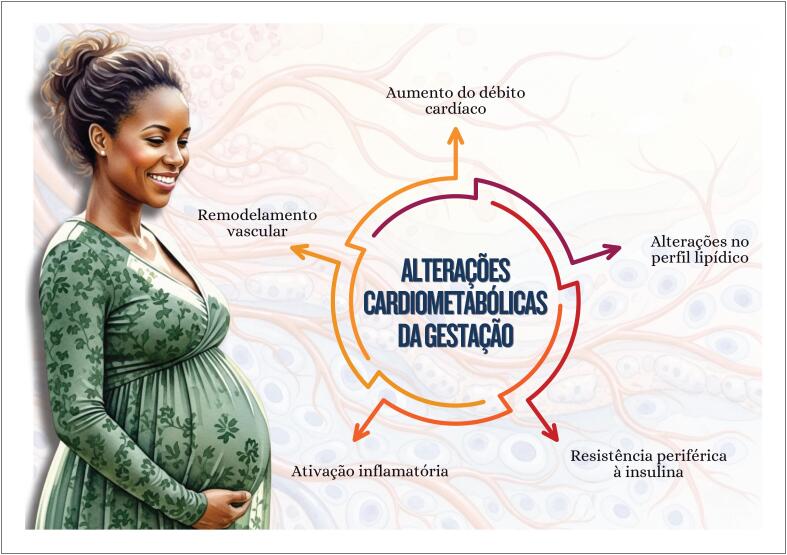
Alterações cardiometabólicas na gravidez.

A Figura 7.1 ilustra as alterações cardiometabólicas na gravidez.

### 7.1 Síndrome dos Ovários Policísticos

A SOP é a endocrinopatia reprodutiva mais comum, presente em 6–10% das mulheres. É fortemente associada a obesidade central, RI, intolerância à glicose e dislipidemia (aumento de LDL-c e triglicerídeos, redução de HDL-c), assim como a maior risco de HAS e aterosclerose precoce,^159^ esteatose hepática e distúrbios respiratórios do sono, com maior incidência de apneia obstrutiva,^5 , 97 , 98^ além de depressão.

A fisiopatologia da SOP é complexa e envolve desregulação do eixo hipotálamo-hipófise-ovariano, redução da síntese de SHBG hepática e hiperandrogenismo ovariano funcional, uma disfunção primária das células da teca do ovário, além dos elevados níveis do hormônio anti-Mülleriano, que exacerba ainda mais a disfunção ovariana.^160^

Atualmente, existem quatro fenótipos reconhecidos de SOP, cada um com diferentes implicações metabólicas e de saúde a longo prazo: 1) hiperandrogenismo + oligoanovulação + morfologia de ovário policístico; 2) hiperandrogenismo + oligoanovulação; 3) hiperandrogenismo + morfologia de ovário policístico; e 4) oligoanovulação + morfologia de ovário policístico.^161^ Os fenótipos com hiperandrogenismo têm o pior perfil metabólico.^62^

As principais alterações metabólicas relacionadas à SOP são: obesidade, presente em cerca de 50% dos casos; RI, que ocorre em 60-95% dos casos, levando à intolerância à glicose em 31-35% dos casos; e DM2 presente em 7,5-20% dos casos.

A RI desempenha papel central nas complicações metabólicas e cardiovasculares. A hiperinsulinemia resultante estimula a produção hepática de triglicerídeos e reduz os níveis de HDL-c, favorecendo o acúmulo de placas ateroscleróticas. Além disso, promove neoglicogênese, aumentando a quantidade de glicose disponível (disglicemia), reduzindo também as proteínas ligadoras dos esteroides sexuais e dos fatores de crescimento insulinoides, o que piora o quadro clínico e aumenta o processo inflamatório crônico.^63^ Isso leva a níveis mais elevados de PCR e citocinas pró-inflamatórias, que também aceleram o dano vascular e a disfunção endotelial.^64^

A dislipidemia é a anormalidade mais frequente na SOP, apresentando-se geralmente com baixos níveis de HDL-c e altas concentrações de triglicerídeos, podendo também cursar com aumento de LDL-c.^179^

O sobrepeso e a obesidade, presentes em muitas mulheres com SOP, pioram o risco uma vez que o tecido adiposo visceral libera ácidos graxos livres e adipocinas inflamatórias, exacerbando a RI e a dislipidemia. Além disso, a hiperandrogenemia na SOP está associada a um perfil lipídico aterogênico, com aumento de LDL-c oxidado e redução do HDL-c, ampliando o risco de eventos cardiovasculares precoces.^62^

Uma análise de 39 revisões sistemáticas e metanálises publicadas até 2019 demonstrou que mulheres com SOP apresentaram maior risco de desenvolver DCV.^102^ Esse risco se manteve quando avaliados AVC e DIC separadamente, mas não houve associação com IC. Observou-se que o risco de eventos cardiovasculares foi maior em mulheres jovens com SOP na idade reprodutiva comparadas a controles normais, mas nenhuma associação foi observada em mulheres na pós-menopausa com SOP.^102^ Atualmente, a maior parte da terapia é centrada na queixa principal da paciente, na redução dos sintomas de hiperandrogenismo, no restabelecimento da regularidade menstrual e na obtenção da concepção em mulheres.

A atualização das Diretrizes Internacionais Baseadas em Evidências de 2023 reintegrou o *International Evidence-Based Guideline for the Assessment and Management of Polycystic Ovary Syndrome* de 2018, abrangendo uma extensa síntese de evidências e recomendações para a SOP.^162^ As principais atualizações incluem:

- fortalecimento do reconhecimento de características mais amplas da SOP, incluindo FR metabólicos, DCV, apneia do sono, prevalência de características psicológicas e o alto risco para resultados adversos durante a gravidez;
- ênfase na carga diversificada e pouco reconhecida da doença e na necessidade de maior educação profissional de saúde;
- ênfase mantida no estilo de vida saudável, no bem-estar emocional e na melhoria da qualidade de vida;
- ênfase na terapia médica baseada em evidências.

A perda de peso deve ser priorizada precocemente.^163^

**Figura 7.2:**
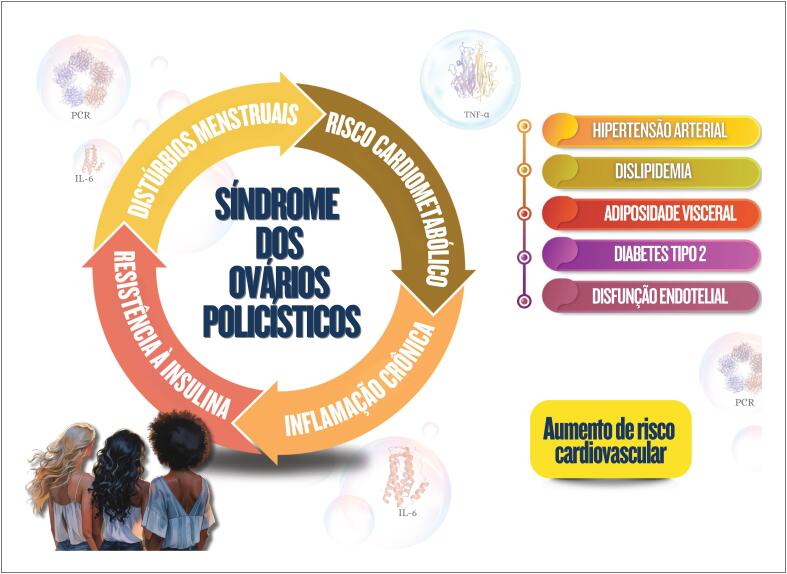
Alterações da síndrome dos ovários policísticos associadas ao aumento do risco cardiovascular.

As alterações da SOP associadas ao aumento do RCV podem ser sumarizadas na Figura 7.2.

### 7.2. Infertilidade e seu Tratamento

**Figura 7.3:**
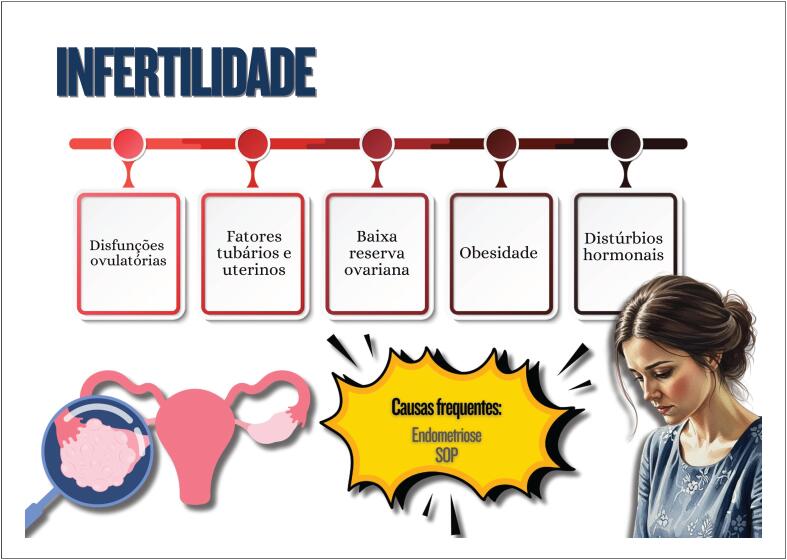
Causas frequentes de infertilidade feminina. SOP: síndrome dos ovários policísticos.

A infertilidade, definida como a ausência de gravidez clínica após 12 meses de relações regulares e desprotegidas, afeta 8-12% dos casais em idade reprodutiva.^175 , 176^ As causas femininas incluem disfunções ovulatórias, fatores tubários e uterinos, baixa reserva ovariana, obesidade e distúrbios hormonais.^177^ As causas mais frequentes são endometriose, que é uma patologia inflamatória, e SOP, que reconhecidamente causa elevação dos hormônios androgênicos e SM.^178 , 179^ Mulder *et al.* avaliaram mulheres com e sem infertilidade da mesma faixa etária e demonstraram que as inférteis são mais propensas a FR cardiometabólicos específicos, com aumento de distúrbios metabólicos, como obesidade e dislipidemia (colesterol total, LDL-c e triglicerídeos), não tendo encontrado, entretanto, alterações da glicemia de jejum, da RI e da PA^180^ ( Figura 7.3 ).

A endometriose tem uma prevalência de até 10% da população feminina e é uma causa frequente de infertilidade, que será abordada separadamente.^181 , 182^

O tratamento para infertilidade também traz repercussões no cardiometabolismo. A estimulação ovariana com clomifeno, letrozol e gonadotrofinas pode induzir síndrome de hiperestimulação ovariana com risco de eventos tromboembólicos.^183^ A fertilização *in vitro* leva a maior risco de TEV, pré-eclâmpsia e eventos cardiovasculares em longo prazo. Além disso, mulheres que engravidam após tratamento para infertilidade apresentam maior risco de DG e distúrbios hipertensivos da gestação.^182^

As intervenções no estilo de vida, como dieta balanceada, atividade física e modificações comportamentais, podem não apenas melhorar a saúde cardiovascular, mas também aumentar o sucesso no tratamento da infertilidade. A perda de peso em mulheres obesas é recomendada para melhorar a saúde reprodutiva e reduzir complicações durante a gravidez. As evidências de que a perda de peso melhora os desfechos da fertilização *in vitro* ainda são limitadas. O controle do peso, entretanto, é considerado benéfico para a fertilidade, assim como para o cardiometabolismo como um todo.

### 7.3. Modificações do Peso na Gestação

As modificações de peso durante a gestação estão intrinsecamente ligadas às adaptações fisiológicas e metabólicas que visam atender às demandas do feto. O monitoramento do GPG é parte importante da consulta pré-natal e merece atenção multidisciplinar. O IMC pré-gestacional e o GPG impactam significativamente os desfechos maternos e infantis, como descrito no capítulo 5. Tanto o excesso quanto a insuficiência de peso estão associados a complicações cardiometabólicas para a mãe e o bebê, como parto prematuro e bebês pequenos para a idade gestacional.^185^

Pesquisadores brasileiros desenvolveram curvas e recomendações de GPG específicas para a população brasileira adotadas pelo Ministério da Saúde a partir de 2022.^186^

O Quadro 7.1 mostra a orientação sobre GPG ajustado para o IMC pré-gestacional em gestação única e gemelar e a distribuição do ganho fisiológico de peso em mulheres com IMC normal.^186 , 187^

**Quadro 7.1:** Distribuição do ganho fisiológico de peso em mulheres com IMC normal (> 18 e < 25 kg/m^2^) e orientação sobre ganho de peso gestacional ajustado para o IMC de acordo com Institute of Medicine, LifeCycle Project-Maternal Obesity and Childhood Outcomes Study Group e FEBRASGO^186 , 187^

Ganho de Peso	IMC (< 18,5 kg/m^2^)	IMC (> 18,5 e < 25 kg/m^2^)	IMC (≥ 25 e < 30 kg/m^2^)	IMC ≥ 30 kg/m^2^
FEBRASGO	9,7 a 12,2 kg	8,0 a 12 kg	7 a 9 kg	5 a 7 kg
IOM:gestações únicas gestação gemelar	12,7 a 18 kg	11,5 a 16 kg 6,8 a 24,5 kg	7 a 11,5 kg 14,1 a 22,7 kg	5 a 9 kg 11,4 a 19,1 kg
Lifecycle Project-Maternal Obesity And Childhood Outcomes Study Group	14 a < 16 kg	10 a < 18	2 a < 16 kg	IMC de 30 a 34,9 kg/m^2^: 2 a < 6 kg IMC de 35 a 39,9 kg/m^2^: 0 a < 4kg IMC > 40 kg/m^2^: 0 a < 5kg
Distribuição do peso (kg)		Feto: 3,2 a 3,6 kg Gordura materna: 2,7 a 3,6 kg Volume sanguíneo: 1,4 a 1,8 kg Volume de líquido extravascular: 0,9 a 1,4 kg Líquido amniótico: 0,9 kg Mamas: 0,45 a 1,4 kg Hipertrofia uterina: 0,9 kg Placenta: 0,7 kg		

IMC: índice de massa corpórea; IOM: Institute of Medicine.

#### 7.3.1. Impacto Materno do Ganho de Peso Gestacional Excessivo

Quase 50% das mulheres iniciam a gestação com sobrepeso ou obesidade e 51% ganham peso acima do recomendado. No pós-parto, as mulheres retêm, em média, de 0,5 kg a 3 kg por gravidez. O sobrepeso e a obesidade no período pré-concepcional estão associados à redução da fertilidade e atraso na concepção. Além disso, aumentam o risco de mortalidade e complicações maternas, tais como GPG excessivo, DG, distúrbio hipertensivo da gestação, cesariana de emergência, doenças congênitas, parto prematuro, óbito fetal e risco futuro de DM e DCV. Estudos indicam que gestantes com sobrepeso ou obesidade tendem a apresentar níveis elevados de leptina, colesterol total, LDL-c, colesterol de lipoproteína de muito baixa densidade (VLDL-c), triglicerídeos, glicemia de jejum e insulina, além de maior HOMA-IR ( *homeostasis model assessment of insulin resistance* ) e PA diastólica mais elevada no terceiro trimestre.^188^

#### 7.3.2. Impacto Fetal do Ganho de Peso Gestacional Excessivo

A obesidade materna está associada a aumento no risco de macrossomia fetal, malformações congênitas e morte intrauterina. Além disso, a exposição intrauterina a um ambiente hiperglicêmico pode predispor o recém-nascido a desenvolver obesidade e SM na vida adulta, devido a alterações na programação metabólica fetal.^189^

**Figura 7.4:**
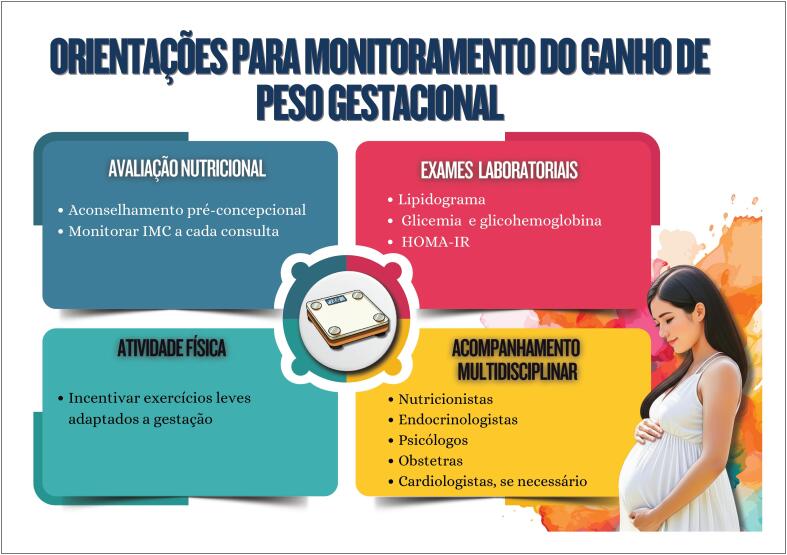
Orientações para monitoramento do ganho de peso gestacional. IMC: índice de massa corpórea; HOMA-IR: homeostasis model assessment of insulin resistance.

Em estudo com 16 milhões de nascimentos nos EUA, gestantes com IMC saudável tiveram as menores taxas de morbidade e mortalidade neonatais, enquanto aquelas com obesidade grau III apresentaram os maiores riscos desses desfechos adversos, independentemente do GPG.^190^ Na Figura 7.4 , estão descritas as orientações para monitoramento do GPG.

### 7.4. Disglicemia Gestacional

A hiperglicemia é a alteração metabólica mais comum da gestação, impulsionada pela epidemia de obesidade, maior prevalência de diabetes mellitus tipo 1 (DM1) e DM2 em mulheres em idade fértil e gravidezes tardias.^191 , 192^ Na gravidez, há redução da glicemia de jejum pela captação fetal e placentária de glicose e discreta hiperglicemia pós-prandial decorrente da ação de hormônios placentários diabetogênicos, mesmo em mulheres com metabolismo normal.^192^

A disglicemia na gestação é classificada como diabetes pré-gestacional, diagnosticada na gestação ou DG. O pré-natal oferece oportunidade de diagnóstico do DM previamente não identificado, com base nos critérios estabelecidos de glicemia, teste oral de tolerância à glicose (TOTG) ou HbA1c.^191 , 192^ A forma mais prevalente de hiperglicemia gestacional é o DG, que afeta até 25% das gestações e é definido como intolerância aos carboidratos iniciada na gestação, sem preencher critérios diagnósticos para DM fora desse período^191 , 192^ ( Quadro 7.2 ).

**Quadro 7.2:** Critérios diagnósticos para diabetes gestacional e diabetes pré-gestacional

	Normal	DG	DM
Glicemia de jejum	< 92 mg/dl	≥ 92 mg/dl e < 126 mg/dl	≥ 126 mg/dL
Glicemia ao acaso			≥ 200 mg/dL
TOTG entre 24ª e 28ª semana		Jejum: ≥ 92 e < 126 mg/dL 1° hora ≥ 180 mg/dL 2° hora ≥ 153 e < 200 mg/dL	2° hora ≥ 200 mg/dL
HbA1c	< 5,7%		≥ 6,5%

DG: diabetes gestacional; DM: diabetes mellitus; HbA1c: hemoglobina glicada; TOTG: teste oral de tolerância à glicose.

A HbA1c, embora menos sensível para diagnóstico de DG, pode identificar risco aumentado quando ≥ 5,7% no primeiro trimestre, indicando necessidade de triagem precoce.^192^ A HbA1c é um bom exame para orientar a mulher diabética que deseja engravidar e valores < 6% reduzem o risco de malformações fetais.^193^ Os principais FR para DG são idade materna avançada, sobrepeso, obesidade, história familiar de DM, RI, hipertrigliceridemia, hipertensão, GPG excessivo e DG prévio.^191^

O DG aumenta o risco de complicações obstétricas e neonatais, como aborto, pré-eclâmpsia, prematuridade, macrossomia, anomalias e morte fetal, hipoglicemia e desconforto respiratório neonatal. A adequada intervenção melhora desfechos.^191 , 192^ Seus efeitos estendem-se ao longo da vida materna, quadruplicando o risco de DM2 e duplicando o risco de eventos cardiovasculares já na primeira década pós-parto, aumento que permanece por toda vida, parecendo ser independente do desenvolvimento do DM2.^194^ Com relação ao feto, há maior risco de SM, obesidade, DM e HAS ao longo da vida.^191 - 193^

O tratamento inicial baseia-se em orientação nutricional e atividade física. Se a glicemia não for controlada em até 14 dias, deve-se iniciar terapia farmacológica, obrigatória em gestantes com DM1 ou DM2 pré-gestacional.^192 , 194 , 195^ Nesses casos, recomenda-se aspirina (100–150 mg/dia) a partir da 12ª-16ª semana para prevenção de pré-eclâmpsia.^192^ A insulina é o fármaco de escolha por sua eficácia, segurança e baixa transferência placentária.^192 , 195^

A automonitorização glicêmica é fundamental, especialmente pós-prandial, para ajuste terapêutico, prevenção de hipoglicemia e menor risco de pré-eclâmpsia.^192 , 195^ As metas glicêmicas incluem valores pré-prandiais de 65-95 mg/dL, 1ª hora pós-prandial < 140 mg/dL e 2ª hora < 120 mg/dL.^195^

A prevenção do DG deve ser iniciada antes da concepção, com controle do peso, dieta saudável e prática regular de atividade física.

### 7.5. Dislipidemia Gestacional

Durante a gravidez o organismo materno realiza adaptações para ajustar o crescimento e o desenvolvimento fetal. Assim, o aumento dos níveis de lipídios e lipoproteínas nessa fase é importante. Essas alterações são estimuladas pelo lactogênio placentário, estrogênio e progesterona e elevações dos níveis de leptina e insulina.^196^ O colesterol é essencial para o desenvolvimento embrionário e fetal, por ser um componente indispensável das membranas celulares e responsável por diversas funções de sinalização intracelular. Além disso, esse aumento do colesterol é necessário para suprir a demanda elevada por esteroides maternos e placentários, que se acumulam no corpo da mãe desde a 7ª semana, com pico no segundo e terceiro trimestres. No fim da gravidez, lipídios armazenados servem como reservatório para síntese de ácidos graxos na placenta.^196^ As adaptações dos lipídios à gestação consistem em um aumento do colesterol total e do LDL-c em cerca de 30-50% e do HDL-c em 20-40%.^197^ Os triglicerídeos se elevam mais significativamente, atingindo 2 a 4 vezes o valor pré-gestacional no terceiro trimestre, pois são, junto com a glicose, uma das principais fontes de energia fetal. O LDL-c altera não só seus níveis, mas aumenta as partículas pequenas e densas, mais aterogênicas, gerando maior impacto em pacientes com hipercolesterolemia familiar. Esse efeito desfavorável é atenuado por níveis elevados de HDL-c e de apolipoproteína A-I, que atingem seu pico durante o segundo trimestre e podem oferecer proteção contra as frações lipídicas aterogênicas.^196^

Na gestação, os níveis de Lp(a) aproximadamente dobram, sendo os mecanismos responsáveis pouco compreendidos. As hipóteses incluem influência do estrogênio na síntese e depuração, atuação como proteína de fase aguda frente a lesão endotelial e possível papel no desenvolvimento placentário.^198^

Os níveis lipídicos na gestação e a magnitude dessas alterações durante a gravidez são influenciados por diversos fatores, incluindo níveis lipídicos pré-gestacionais, IMC, idade, dieta e etnia.

A amamentação melhora o perfil lipídico, com maior redução de LDL-c e triglicerídeos do que de HDL-c.^196 , 199^

### 7.5.1. Impacto da Dislipidemia na Gravidez

A avaliação lipídica no primeiro trimestre pode prover informações de valor para resultados futuros de curto e longo prazo para a mãe e a criança, além de identificar grupos de risco específicos.^197^ A dislipidemia (elevação de apolipoproteína B, colesterol total, LDL-c e triglicerídeos), especialmente no início da gestação, está associada a desfechos adversos para a mãe e para o recém-nascido. Demonstrou-se que um perfil lipídico aterogênico aumenta o risco de lesão endotelial por meio de mecanismos de estresse oxidativo na parede arterial. Os riscos maternos incluem prematuridade, pré-eclâmpsia, HAS, DG, SM e perfis lipídicos desfavoráveis. Os riscos para os recém-nascidos incluem parto prematuro, macrossomia, desenvolvimento de lesões pré-ateroscleróticas e perfis lipídicos desfavoráveis. Mulheres com prematuridade têm duas vezes mais risco de desenvolverem DCV tardiamente. As placentas exibem alterações, como aterosclerose, infarto de vilosidades e trombose. Talvez a abordagem das anormalidades lipídicas na gravidez possa ajudar a reduzir risco de prematuridade.^200^

O perfil lipídico na gravidez também foi relacionado com desenvolvimento de SM anos depois, podendo ser usado como marcador precoce de saúde cardiovascular da mulher, como descrito no capítulo 5. Além disso, pode prever o perfil lipídico de crianças, sendo também usado como preditor de saúde de crianças em longo prazo. Monitorar essa fase pode ser uma janela de oportunidade para iniciar intervenção precoce e, possivelmente, reduzir RCV na vida futura. Lipídios aumentados têm sido associados com faixas de gorduras na aorta e rápida progressão de aterosclerose na infância.^201^

A elevação da Lp(a), uma proteína inflamatória, pode influenciar negativamente os desfechos gestacionais, aumentando o risco de complicações como pré-eclâmpsia, DM, parto prematuro e baixo peso ao nascer. Essas condições representam risco no curto prazo de morbimortalidade materna e fetal, além de estarem associadas a um aumento do RCV a longo prazo, incluindo IM, AVC e IC.^198^

Assim, a triagem lipídica no primeiro trimestre pode fornecer informações valiosas sobre desfechos de curto e longo prazo para a mãe e o recém-nascido, além de identificar grupos de risco específicos.

### 7.5.2. Impacto da Gravidez em Pacientes com Hipercolesterolemia Familiar

A hipercolesterolemia familiar é uma condição autossômica semidominante, causada por mutações em genes relacionados ao metabolismo lipídico, resultando em níveis elevados de LDL-c e risco de DIC precoce.

Mulheres com hipercolesterolemia familiar heterozigótica apresentam níveis de colesterol total e LDL-c aproximadamente duas vezes maiores que aquelas sem a condição. Em um estudo norueguês, observou-se que, embora o aumento relativo de colesterol total e LDL-c entre a 17ª e a 36ª semana gestacional fosse semelhante entre os grupos, o aumento absoluto foi significativamente maior nas mulheres com hipercolesterolemia familiar. Os triglicerídeos também se mostraram mais elevados nesse grupo embora ainda dentro da faixa de normalidade, com padrão de elevação relativo semelhante. Já os níveis de HDL-c permaneceram inalterados em ambos os grupos. Apesar de se especular sobre possíveis efeitos epigenéticos da exposição fetal a altos níveis de colesterol, os mecanismos envolvidos permanecem incertos diante de dados ainda conflitantes na literatura.^196^

Apesar da associação entre dislipidemia gestacional e desfechos adversos materno-fetais, as diretrizes da Sociedade Europeia de Cardiologia (ESC) restringem-se a uma breve menção sobre o uso de estatinas e desencorajam seu uso em mulheres em idade fértil que pretendem engravidar. O Centro de Controle e Prevenção de Doenças dos Estados Unidos (CDC – *Centers for Disease Control and Prevention* ) também não oferece recomendações específicas. A orientação é evitar medicamentos hipolipemiantes durante a gestação e a lactação, exceto em casos graves, como hipercolesterolemia familiar, onde se consideram sequestrantes de ácidos biliares (não absorvidos) ou aférese de LDL-c.^196^ A escassez de dados sobre tratamento decorre, em grande parte, da exclusão sistemática de gestantes em ensaios clínicos, o que limita o conhecimento sobre a segurança de medicamentos hipolipemiantes. Metanálises e revisões sistemáticas sobre o tema têm dados controversos e com vieses.^196^

#### 7.6. Endometriose e Risco Cardiovascular

Foi demonstrada a associação de endometriose com FR para DCV, como HAS e dislipidemia com perfil aterogênico, assim como com aumento do risco de TEV, DIC, IC e AVC.^202^ A endometriose, doença inflamatória crônica e estrogênio-dependente, é a principal causa de dor pélvica crônica em mulheres jovens e uma das principais causas de infertilidade. Está associada a um processo inflamatório crônico mediado por substâncias, como molécula de adesão intercelular, IL-1 e IL-6, TNF-α e VEGF, que induzem o aumento do estresse oxidativo.^181^ Uma revisão sistemática com 254.929 participantes revelou que a condição está associada a maior risco de DIC (HR 1,50) e doença cerebrovascular (HR 1,17).^203^

O espectro completo da patogênese e fisiopatologia da endometriose é reconhecido como uma condição multifatorial envolvendo processos hormonais, pró-inflamatórios, pró-angiogênicos, imunológicos e genéticos. Estudos genéticos identificaram variantes associadas a doenças complexas, como a DIC, ampliando o conhecimento sobre sua fisiopatologia e sugerindo novos alvos terapêuticos, especialmente no metabolismo lipídico. Além de fatores hormonais e inflamatórios, há um componente genético relevante, com desregulação do inflamossomo, promovendo proliferação celular e inflamação crônica. Esse processo inflamatório contribui para um estado pró-trombótico, favorecendo aterosclerose e sustentando a hipótese de que endometriose seja um FRCV.^182 , 204^

##### 7.6.1. Fatores de Risco

Na endometriose, ocorre uma elevação de FR bem estabelecidos de DCV, como hipertensão e dislipidemia com perfil aterogênico maior, além de aumento do risco de TEV, DIC, IC e AVC.^182^ Estudos populacionais indicam associação entre endometriose e HAS, com risco relativo (RR) de 1,14 para HAS em mulheres com endometriose e RR de 1,29 para endometriose em hipertensas, sugerindo mediação inflamatória comum. Em relação à dislipidemia, dados do *Nurses’ Health Study II* ^205^ demonstraram risco 25% maior de hipercolesterolemia entre mulheres com endometriose, além de maior prevalência da doença em mulheres com perfil lipídico aterogênico. Alterações no metabolismo de fosfolipídios e esfingolipídios desempenham papel relevante na fisiopatologia.

Embora os dados sobre tabagismo, diabetes e poluição sejam inconclusivos, uma metanálise recente apontou risco aumentado de 23% para DCV e 13% para hipertensão em mulheres com endometriose. Também foi observada associação com eventos coronarianos (RR 1,62), apesar da heterogeneidade metodológica entre os estudos. Histerectomia antes dos 50 anos, com ou sem ooforectomia, foi correlacionada a maior risco de DIC, possivelmente devido a um perfil cardiometabólico adverso. Além disso, o tratamento hormonal para endometriose pode impactar negativamente o RCV por efeitos sobre peso e metabolismo lipídico. Estilo de vida e fatores comportamentais, como sedentarismo e dieta inadequada, contribuem para a interrelação entre endometriose e DCV.^206^

**Figura 7.5:**
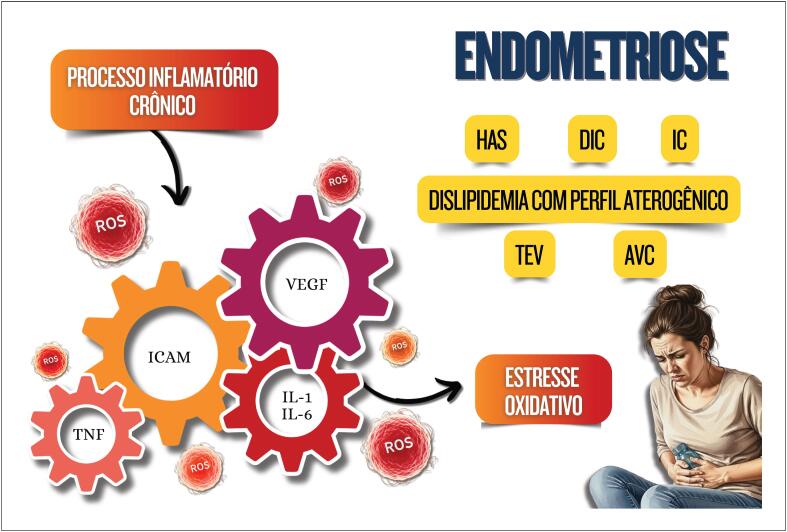
Marcadores inflamatórios e doenças cardiovasculares associadas com endometriose. HAS: hipertensão arterial sistêmica; DIC: doença isquêmica do coração; IC: insuficiência cardíaca; TEV: tromboembolismo venoso; AVC: acidente vascular cerebral; ROS: espécies reativas de oxigênio; VEGF: fator de crescimento endotelial vascular; ICAM: molécula de adesão intercelular; TNF: fator de necrose tumoral; IL-1: interleucina-1; IL-6: interleucina-6.

Torna-se premente o reconhecimento da endometriose como potencial FR para DCV para que, a partir disso, sejam abordadas estratégias de mudanças de estilo de vida e intervenção precoce, visando à prevenção e redução do RCV nessas mulheres.^182 , 206^ A Figura 7.5 descreve os marcadores inflamatórios e as DCV associadas com endometriose.

#### 7.7. Psoríase

A psoríase é tradicionalmente conhecida por causar placas inflamatórias na pele. Evidências crescentes, entretanto, mostram que se trata de doença inflamatória crônica sistêmica associada a diversas comorbidades cardiovasculares, como obesidade, SM e DCV, doenças cerebrovasculares, arritmias cardíacas, apneia do sono, dentre outras. Embora a fisiopatologia ainda não esteja totalmente esclarecida, acredita-se que a inflamação sistêmica da psoríase se relacione a estado pró-inflamatório, com participação de citocinas, como TNF-α, IL-6, leptina e outras adipocinas.

O estudo das enfermeiras americanas demonstrou, entre aquelas com psoríase, risco aumentado de desenvolver DM (RR 1,63) e HAS (RR 1,17).^207^ Recente revisão sistemática e metanálise mostrou a associação de psoríase e DIC.^208^

#### 7.8. Distúrbios Hipertensivos da Gestação e Disfunção Endotelial após a Menopausa

Os distúrbios hipertensivos da gestação afetam até 10% das gestações e representam importantes preditores de RCV ao longo da vida, sendo identificados como FR exclusivo do sexo feminino. Evidências crescentes sugerem que mulheres com histórico de distúrbios hipertensivos da gestação apresentam alterações persistentes na função endotelial mesmo décadas após a gestação, contribuindo para maior risco de DCV na pós-menopausa.^209^ Em todos os temas abordados nas outras sessões, já observamos o grande impacto na saúde cardiometabólica futura das mulheres que apresentaram distúrbios hipertensivos da gestação.

A disfunção endotelial, caracterizada por redução da biodisponibilidade de ON e aumento de marcadores inflamatórios e pró-trombóticos, é um evento precoce na aterogênese. Estudos longitudinais demonstraram que mulheres com pré-eclâmpsia prévia apresentam níveis elevados de endotelina-1, disfunção da vasodilatação dependente do endotélio e espessamento médio-intimal da carótida anos após o parto. Esse impacto parece amplificar-se após a menopausa, com piora do perfil cardiometabólico.^210 , 211^

Além disso, biomarcadores de ativação endotelial, como VCAM-1 (molécula de adesão celular vascular) e E-selectina, permanecem elevados no pós-parto tardio, sugerindo inflamação crônica de baixo grau. A interrupção precoce da exposição estrogênica, comum em mulheres com parto prematuro por pré-eclâmpsia, também pode antecipar a perda da proteção vasculogênica hormonal. A inclusão do histórico obstétrico na estratificação de RCV é fundamental para estratégias preventivas individualizadas, especialmente no climatério.^212^

Portanto, a pré-eclâmpsia deve ser considerada não apenas uma complicação gestacional isolada, mas um marcador de suscetibilidade vascular feminina ao longo da vida.^213^ A recente diretriz da ESC aponta para os fatores específicos por sexo como potenciais reclassificadores de RCV para uma categoria de risco mais elevado. Os distúrbios hipertensivos da gestação devem ser considerados na estratificação individualizada de RCV, embora poucos tenham demonstrado melhorar a predição ou a discriminação de risco além dos fatores tradicionais.^214^

O principal nessas mulheres é intensificar a abordagem dos oito pilares da prevenção cardiovascular precocemente desde o pós-parto, como o controle de PA, glicemia, dislipidemia e peso, incentivar atividade física, qualidade do sono, interrupção do tabagismo e alimentação saudável.^213 , 214^

#### 7.9. Alterações Metabólicas no Período Pós-parto

Nas últimas décadas, houve um significativo avanço nos protocolos de assistência à gravidez e ao parto. No entanto, o período pós-parto continua sendo negligenciado, apesar de sua importância crítica para a saúde materna a longo prazo. Nessa fase, as mulheres enfrentam mudanças significativas, tanto na recuperação do aparelho reprodutivo quanto nos aspectos metabólicos, endócrinos e nutricional.

Uma das mudanças mais evidentes é a variação no peso corporal. Em média, a perda de peso ocorre a uma taxa de 0,6-0,8 kg/mês nos primeiros seis meses após o parto.^215^ Entretanto, muitas mulheres retêm ou até ganham peso, o que pode levar a obesidade e aumentar o risco de complicações cardiometabólicas. A retenção de peso após o parto tem implicações importantes para a saúde cardiovascular, como ilustrado na Figura 7.4. ^216^

##### 7.9.1. Fatores de Risco Cardiometabólico no Pós-parto

Estudos recentes indicam que fatores como raça/etnia, condição socioeconômica e histórico de DG são fortes preditores de risco cardiometabólico no pós-parto.^217 , 218^ Para minimizar esses riscos algumas estratégias devem ser consideradas:

1. **Lactação** : a amamentação atua como fator protetor contra a obesidade materna, auxiliando no metabolismo lipídico por meio da lipólise hepática e da ação combinada da prolactina e da insulina;^219^
2. **Dieta** : a alimentação no pós-parto deve focar na redução calórica e na qualidade nutricional. Recomenda-se um adicional de 500 kcal/dia nos primeiros seis meses e 400 kcal nos meses subsequentes para garantir um equilíbrio metabólico adequado;^220^
3. **Atividade física** : O exercício físico melhora a aptidão cardiorrespiratória, preserva a massa magra e auxilia na perda de peso, reduzindo o risco metabólico;^221^
4. **Sono** : A privação de sono (< 5 horas/dia) tem impacto negativo no metabolismo da glicose e favorece a obesidade e a RI.^222^

##### 7.9.2. Impacto de Diabetes Gestacional e Dislipidemia no Pós-parto

A retenção excessiva de peso no pós-parto está associada a maior risco de dislipidemia e RI. Mulheres com histórico de DG têm maior probabilidade de apresentar distúrbios metabólicos no pós-parto, incluindo hiperinsulinemia e hipertrigliceridemia. Esses efeitos parecem ser influenciados pelo IMC e pela idade gestacional em que surgiram as alterações metabólicas. Estudos mostram que, mesmo anos após o parto, mulheres com DG mantêm níveis elevados de colesterol total e LDL-C, independentemente de outros FRCV tradicionais.^223^ Essas mulheres tendem a apresentar níveis elevados de triglicerídeos e LDL-c, independentemente do IMC até meses após o parto. A RI e a hiperglicemia na gestação contribuem para persistência da dislipidemia no pós-parto, impactando a saúde cardiovascular a longo prazo.^224 , 225^

##### 7.9.3. Prolactina e Metabolismo no Pós-parto

Além de sua função na lactação, a prolactina exerce um papel relevante no metabolismo materno. Embora a relação entre os níveis de prolactina e o DG ainda não esteja totalmente esclarecida, evidências sugerem que, no pós-parto, em especial durante a lactação, níveis elevados de prolactina estejam associados a menores níveis de insulina circulante, maior função das células beta e aumento da sensibilidade à insulina.^226^

O sobrepeso e a obesidade materna, o GPG excessivo e sua retenção no pós-parto são FR bem estabelecidos para complicações cardiometabólicas. Nesse contexto, o pós-parto deve ser visto como uma janela de oportunidades para intervenções preventivas, enfatizando-se uma dieta equilibrada e de qualidade, exercício físico regular, controle do peso corporal, sono adequado e amamentação. A adoção dessas estratégias pode contribuir para a preservação da saúde cardiovascular materna e a redução do risco de distúrbios cardiometabólicos ao longo da vida.

## 8. Transição Menopausal, Menopausa e Pós-Menopausa e Saúde Cardiometabólica

Os distúrbios cardiometabólicos, como o DM2 e as DCV com seus fatores associados, como obesidade, RI, hipertensão e dislipidemia, são as principais causas de mortalidade e carga de doenças em ambos os sexos.^73 , 109^ No entanto, homens e mulheres vivenciam trajetórias diferentes de risco cardiometabólico ao longo da vida influenciadas pelas flutuações hormonais. A fase reprodutiva das mulheres constitui-se em uma janela de oportunidade para prevenção e abordagem dos FRCV e dos distúrbios cardiometabólicos que serão magnificados na transição para a menopausa, durante a menopausa e no período pós-menopausa.^73 , 109^ A diminuição dos níveis endógenos de estradiol durante a transição da menopausa foi associada a um risco aumentado de problemas de saúde cardiometabólica, incluindo adiposidade abdominal, dislipidemia, DM2 e hipertensão.^73 , 109^

Em geral, o último período menstrual acontece em 90% das mulheres entre 45 anos e 55 anos de idade e, de forma paralela, o RCV aumenta na quinta década de vida, 10 anos após o que se observa no sexo masculino. A idade do início da menopausa parece não ser apenas um marcador do envelhecimento reprodutivo, com o declínio do estrogênio, mas também do aumento das complicações cardiometabólicas.^73 , 109^

**Figura 8.1:**
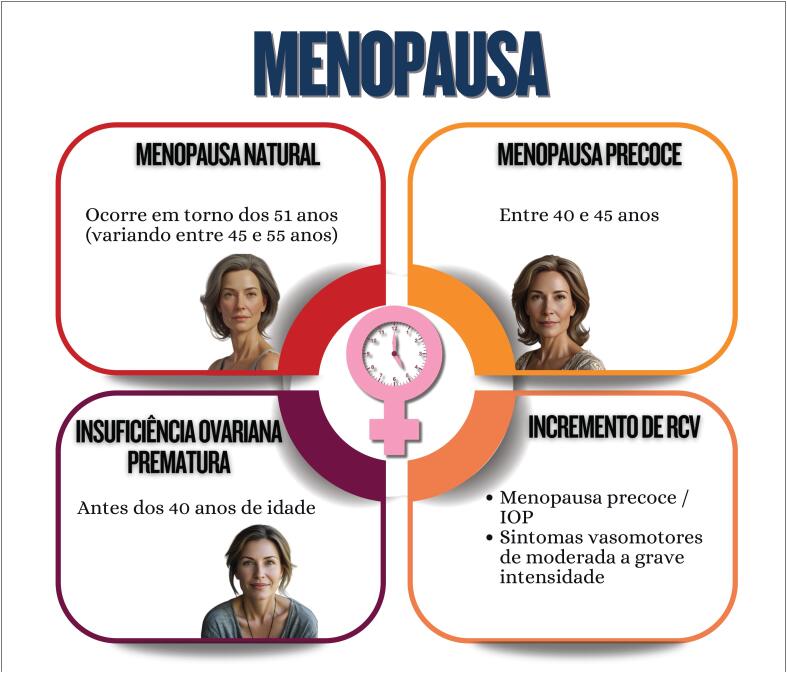
Conceituação de menopausa e aumento do risco cardiovascular. RCV: risco cardiovascular; IOP: insuficiência ovariana prematura.

A menopausa antes dos 40 anos é considerada prematura, hoje denominada insuficiência ovariana prematura, sendo sua prevalência de cerca de 1%. Quando acontece entre 40 anos e 45 anos, a menopausa é classificada como precoce, atingindo cerca de 7,3% das mulheres. As evidências demonstram que as mulheres com menopausa prematura natural ou cirúrgica têm maior chance de desenvolvimento de DCV^73 , 109^ ( Figura 8.1 ). No estudo de Honigberg *et al* ., que avaliou 144.260 mulheres, a menopausa prematura natural foi associada de forma independente a estenose aórtica, TEV, AVC isquêmico, DIC e fibrilação atrial. Já a menopausa cirúrgica foi relacionada principalmente a regurgitação mitral, tromboembolismo pulmonar, IC e DIC. A idade mais jovem na menopausa permaneceu independentemente associada ao tempo até o primeiro diagnóstico de DCV incidente (HR, 1,02/ano de idade precoce na menopausa [IC 95%, 1,01–1,03]; p < 0,001).^157^

Além disso, as mulheres com insuficiência ovariana prematura na menopausa prematura e menopausa precoce apresentam risco substancialmente maior de um evento cardiovascular não fatal, em especial de DIC e AVC antes dos 60 anos, possivelmente relacionados aos níveis baixos de estrogênios que estão vinculados à progressão da aterosclerose subclínica. Um estudo que reuniu dados individuais de 301.438 mulheres em cinco países relatou um risco 30% maior de DCV em mulheres com menopausa precoce do que em mulheres que entraram na menopausa aos 50–51 anos.^227 , 228^

A literatura descreve associação estatisticamente significativa da idade precoce na menopausa natural com o risco de mortalidade por causas gerais e cardiovasculares, enquanto a idade mais avançada gera expectativa de vida mais longa, maior densidade mineral óssea e menor risco de fraturas.^229 , 230^

### 8.1. Gravidade dos Sintomas e suas Implicações na Saúde Cardiovascular

A gravidade das manifestações clínicas na transição da menopausa também está relacionada à saúde cardiovascular das mulheres. São caracterizadas principalmente por SVM, suores noturnos, queixas neurológicas como distúrbios de sono, humor, cognição e memória, bem como alterações das funções geniturinárias e sexuais. Prejudicam a qualidade de vida, produtividade, saúde física e mental das mulheres.^73 , 109^

Os SVM constituem as principais queixas, acometendo até 80% das mulheres durante a transição da menopausa, com pico em torno do período menstrual final.^231^ Foram associados à elevação da PA e ao aumento do risco de DCV, como AVC e DIC. Os principais fatores causais envolvidos incluem desregulação autonômica com exacerbação do sistema nervoso simpático, interrupção do sono, disfunção endotelial e maior aterosclerose subclínica.^232 , 233^ As evidências sugerem associação de início precoce, duração e gravidade dos SVM com o risco de DCV.^232^ Os distúrbios do sono, por sua vez, são relatados por 40% a 60% das mulheres na menopausa, interferindo negativamente na qualidade de vida, bem como na saúde mental e física.^234^

A identificação de insuficiência ovariana prematura e menopausa precoce ou prematura, bem como da gravidade dos sintomas, é fundamental para reconhecer o aumento do RCV em mulheres de meia-idade. Essa abordagem permite avaliar a janela terapêutica para a reposição hormonal e intensificar modificações no estilo de vida, otimizando o controle de outros fatores importantes para a redução de eventos cardiovasculares, como PA, colesterol, glicemia, peso corporal, qualidade do sono, estresse mental, aptidão cardiorrespiratória e cessação do tabagismo.

### 8.2. Fatores de Risco Cardiovascular e Menopausa

A hipertensão é um dos principais FRCV em mulheres e tem associação mais forte que em homens com o risco de DCV.^235^ Globalmente, estima-se que a taxa de controle da PA seja de 23% em mulheres com HAS.^236^ Com o envelhecimento e após a menopausa, a prevalência de HAS aumenta e a taxa de controle da PA diminui.^235^ Fatores como estilo de vida e dieta desfavoráveis, obesidade e envelhecimento contribuem para o aumento da PA, assim como características relacionadas à menarca, reprodução e menopausa.

Concomitante com a menopausa, observa-se uma diminuição nos níveis de estrogênio e de andrógenos circulantes. Ocorre desregulação do sistema renina-angiotensina-aldosterona, ativação simpática, disfunção endotelial, inflamação e maior sensibilidade ao sódio.^237 , 238^ Isso leva a um rápido e acentuado aumento da PA,^239^ alta prevalência de HAS na pós-menopausa, com consequente aumento do risco de eventos cardiovasculares, como IAM, ICFEp, AVC, déficit cognitivo e doença arterial periférica, em geral com PA sistólica 10 mmHg menor que em homens.^240^ Mais ainda, as mulheres com HAS cursam mais frequentemente com DRC, hipertrofia ventricular esquerda e disfunção de microcirculação coronariana.^235^

Na abordagem da HAS, o monitoramento da PA fora do consultório é importante devido à alta incidência de hipertensão do avental branco e hipertensão mascarada. Entre as medidas não medicamentosas, destaca-se a restrição de sal. O exercício físico reduz a PA e a rigidez arterial.^241^ Para o tratamento medicamentoso, não há recomendações específicas de classes de anti-hipertensivos para mulheres na menopausa. Eventos adversos podem ser mais frequentes em mulheres, provavelmente relacionados às propriedades farmacocinéticas e farmacodinâmicas dos anti-hipertensivos.^235 , 238^

Em relação aos lipídios, estudos mostraram que, em mulheres na pós-menopausa, os níveis de colesterol total e LDL-c são maiores e as taxas de tratamento e controle da dislipidemia mais baixas do que em homens da mesma idade.^242 , 243^ Os níveis de HDL-c são menores.^231^ Estudos indicam que a função antiaterogênica do HDL-c pode estar comprometida e, nesse caso, níveis elevados de HDL-c podem se associar a aterosclerose.^244^ Além disso, a concentração de Lp(a) tende a aumentar durante a menopausa e níveis elevados de Lp(a) são mais comuns em mulheres do que em homens após os 50 anos, agravando o risco de DCV.^235 , 243^

Essas particularidades no comportamento da PA e nos distúrbios lipídicos sugerem que as diretrizes existentes para o manejo dessas condições podem ser inadequadas para atender às necessidades específicas das mulheres. Portanto, intervenções direcionadas, incluindo medidas não medicamentosas e medicamentosas, são essenciais para melhorar o cuidado cardiovascular em mulheres na menopausa. Vale lembrar que as mulheres são sub-representadas em estudos clínicos, o que determina escassez de informações científicas robustas.^233 , 235 , 238 , 244^

Na menopausa, o declínio da função ovariana resulta em um impacto significativo não apenas sobre a PA, mas também sobre outros FR cardiometabólicos, incluindo ganho de peso, adiposidade central, perfil lipídico aterogênico e aumento da glicemia associado à RI, um conjunto que constitui ameaça significativa à saúde cardiovascular da mulher.^235 , 238 , 243^

Fatores, como raça/etnia, fatores reprodutivos, composição corporal, estilo de vida, genética e saúde cardiovascular pré-menopausa, podem afetar a menopausa natural e estão associados ao risco cardiometabólico. Em um estudo de coorte incluindo 3.639 mulheres holandesas menopausadas naturalmente, aquelas que vivenciaram menopausa natural precoce apresentaram um risco 2,4 vezes maior de DM2 em comparação àquelas com início tardio da menopausa, viveram menos e passaram menos anos sem DM2 do que mulheres que vivenciaram menopausa normal ou tardia.^245^

Por outro lado, um estudo envolvendo 177.131 mulheres de quatro países diferentes constatou que aquelas com um evento cardiovascular antes dos 35 anos, em comparação com mulheres sem DCV, apresentaram risco duas vezes maior de menopausa precoce, levantando a hipótese de que as associações podem ser bidirecionais. Desse modo, um pior perfil de saúde cardiovascular na pré-menopausa pode influenciar o início da menopausa natural.^227^

A menopausa é acompanhada por diversas adaptações metabólicas, relacionadas à RI, ao aumento da massa gorda corporal total e ao acúmulo de gordura abdominal central, predispondo as mulheres ao desenvolvimento de DM2. A SM tem alta prevalência em mulheres menopausadas, indicando a perda da proteção estrogênica na saúde metabólica e cardiovascular. Além disso, menopausa precoce foi relacionada ao aumento do risco de DM2.^73 , 109^

Adicionalmente, a alta exposição estrogênica em mulheres na pré-menopausa, como em caso de gravidez, foi associada a alterações metabólicas adversas (hiperglicemia ou aumento da PA) que poderiam impactar o risco de desenvolver DM2 e HAS mais tarde na vida.^246^

Fatores ambientais e relacionados ao estilo de vida, incluindo dieta, consumo de álcool, atividade física, sobrepeso e obesidade, tabagismo, exposição a tóxicos ambientais, fatores sociodemográficos, psicológicos e sociocognitivos, entre outros, estão associados tanto ao risco de menopausa natural precoce quanto ao risco de doença cardiometabólica. Fatores ambientais e relacionados ao estilo de vida podem desencadear mecanismos subjacentes à doença cardiometabólica associada à menopausa, incluindo sua influência na metilação do DNA e na expressão gênica, indução de inflamação de baixo grau e estresse oxidativo, interferindo possivelmente na sinalização relacionada aos hormônios na menopausa.^247^

### 8.3. Terapia de Reposição Hormonal da Menopausa, Implantes Hormonais e Impacto Cardiometabólico

A THM é utilizada como a intervenção mais efetiva para aliviar os sintomas da menopausa oferecendo benefícios significativos, ainda que não possa ser empregada para a prevenção primária ou secundária da doença cardiometabólica. Com a administração de estrogênio e, em alguns casos, progesterona, a THM busca aliviar os sintomas e melhorar a qualidade de vida. A THM pode ser administrada de diversas formas, incluindo comprimidos, adesivos, géis ou spray. Fatores como início e momento da terapia, duração, dose e via de administração são determinantes de seus benefícios e malefícios. A escolha do tipo de THM depende de vários fatores, como riscos associados, indicações e contraindicações.^73 , 109^

O início da THM durante os primeiros dez anos da menopausa acarreta mais segurança em relação ao risco de distúrbios cardiometabólicos, resultando em um menor risco absoluto de TEV, DCV e AVC nos primeiros anos da menopausa, devendo ser administrada pelo menor prazo e com a menor dose, tendo a via transdérmica menos efeitos metabólicos. As preparações orais de THM em mulheres com risco tromboembólico basal podem aumentar o risco de TEV e AVC de forma dose-dependente; por outro lado, o estradiol transdérmico (isoladamente ou com progesterona micronizada) é considerado mais seguro. A via vaginal pode ser uma abordagem complementar, indicada apenas para tratamento dos sintomas geniturinários.^73 , 109^

Ensaios clínicos randomizados e controlados sugeriram que a THM poderia reduzir a RI e a incidência de DM2, melhorar o metabolismo da glicose, aumentar a supressão da produção hepática de glicose, tendo as preparações orais maior potencial para melhorar o metabolismo da glicose e diminuir o risco de desenvolver DM2.^155 , 248^ O 17β-estradiol oral geralmente é preferido em mulheres com DM2, pois essa via apresenta efeitos mais benéficos no metabolismo da glicose. Estrogênios orais também são sugeridos em mulheres na perimenopausa ou recentemente menopausadas com baixo risco de DCV. Progesterona micronizada ou didrogesterona por via oral e noretisterona oral ou transdérmica são os progestagênios mais usados em mulheres na pós-menopausa com DM2 e útero intacto. A administração de THM em mulheres na pós-menopausa com DM2 pode ser segura e eficaz, desde que o regime terapêutico tenha sido adequadamente selecionado. No entanto, o uso da THM não é recomendado para a prevenção primária de DM2 ou distúrbios cardiometabólicos.^73 , 109 , 117^

A terapia de reposição de testosterona não está indicada para a melhora de saúde cardiometabólica ou musculoesquelética, SVM ou alterações de humor. Não estão disponíveis estudos robustos suficientes sobre o impacto dos andrógenos na saúde cardiometabólica. Também existem poucos estudos clínicos sobre implantes hormonais para uso em THM, sendo, em sua maioria, sobre implantes de testosterona. Esses estudos apresentam casuística pequena e/ou metodologia com baixo grau de evidência (estudos retrospectivos ou observacionais), o que não permite conhecer os efeitos cardiometabólicos desses implantes, nem o risco de câncer de mama e de endométrio, não sendo, portanto, recomendados para uso na THM.^73 , 109 , 117^

### 8.4. Estratificação do Risco Cardiometabólico na Menopausa

Durante a menopausa e no período pós-menopausa, há uma queda abrupta nos níveis de estrogênio, hormônio fundamental para a proteção cardiovascular feminina. Entre outras alterações, essa deficiência hormonal promove: aumento de LDL-c, colesterol total, apolipoproteína B e triglicerídeos; disfuncionalidade do HDL-c, com redução de sua capacidade de promover efluxo de colesterol, levando a perda de parte de sua proteção cardiovascular; intolerância à glicose e aumento do risco de DM2; acúmulo de gordura visceral e depósitos ectópicos no fígado e coração, exacerbando a inflamação subclínica; e aumento simultâneo de obesidade abdominal, hipertensão, dislipidemia e hiperglicemia.^2 , 249^

Nos Estados Unidos, entre 2013 e 2017, observou-se um aumento de 7% nas taxas de DCV entre mulheres de meia-idade, atribuído principalmente ao crescimento da obesidade e da prevalência de FR cardiometabólico. Notavelmente, mulheres com histórico de DM2, HAS e tabagismo apresentam risco relativo significativamente maior para DCV em comparação a homens com os mesmos FRCV. Em especial, o DM2 aumenta o RCV em 3 a 7 vezes nas mulheres, contra 2 a 3 vezes nos homens, possivelmente devido a um IMC mais elevado, maior inflamação sistêmica, controle glicêmico mais deficiente e maior carga de FR no momento do diagnóstico feminino.^2 , 250^

A perda estrogênica também afeta a função endotelial e a integridade vascular com disfunção endotelial que promove a redução da dilatação mediada por fluxo, rigidez arterial aumentada refletida pelo aumento da velocidade da onda de pulso, espessamento da íntima-média carotídea, que é um marcador de aterosclerose subclínica, aumento do escore de cálcio coronariano (CAC) e presença de calcificação arterial mamária. O CAC obtido por tomografia sem contraste é atualmente reconhecido como um marcador robusto da carga aterosclerótica e importante preditor de risco de DIC, reclassificando o RCV aferido pelos FR tradicionais. Estudos demonstram que, embora mulheres tenham menos calcificação coronariana do que homens da mesma idade, a presença ou o aumento do CAC associa-se a risco relativo maior em mulheres. Além disso, mesmo em mulheres consideradas de "baixo risco" pelo escore de Framingham, um CAC detectável (>0) aumenta em até cinco vezes o RCV. A calcificação arterial mamária detectada incidentalmente em mamografias também emerge como marcador independente de risco para DCV, com risco aumentado de 2,4 vezes. Sua detecção justifica, em muitos casos, a intensificação do rastreio de FR e a avaliação complementar com CAC.^208 , 209^

Além disso, os biomarcadores têm papel relevante para a avaliação do risco cardiometabólico nas mulheres no período de transição da menopausa, na menopausa e na pós-menopausa. Dentre os biomarcadores destacamos: PCR-us que é um preditor independente de RCV (geralmente acima de 3 mg/L ou 0,3 mg/dL), mesmo em mulheres com níveis lipídicos normais, integrando o Reynolds Risk Score; níveis séricos de triglicerídeos (≥ 175 mg/dL), Lp(a) (≥ 50 mg/dL ou ≥ 125 nmol/L) e apolipoproteína B elevados (≥ 130 mg/dL), que são fatores agravantes; e as troponinas de alta sensibilidade e peptídeos natriuréticos, que possuem valor prognóstico para eventos cardiovasculares futuros, embora ainda não recomendados rotineiramente pelas diretrizes da ACC/AHA para rastreio populacional de pacientes assintomáticos.^2 , 117 , 249^

Antes de iniciar a THM, é essencial considerar o RCV total da paciente. A avaliação inicial inclui: perfil lipídico completo (colesterol total, LDL-c, HDL-c e triglicerídeos), glicemia de jejum, HbA1c e mamografia.^226^ Na ausência de escores específicos na perimenopausa e pós-menopausa, utilizam-se os escores tradicionais, podendo ser refinados pela identificação de FR potencializadores e marcadores de aterosclerose subclínica. Pacientes com DM2, DRC, hipercolesterolemia familiar ou HAS grave são consideradas automaticamente de alto ou muito alto risco.^209 , 250^ Essa avaliação inicial através do escore de RCV é importante para definir se a THM poderá ser prescrita e a melhor via de administração, visto que, nos casos de RCV moderado, deve-se optar preferencialmente pela via transdérmica.^226^

Fatores como idade do início da menopausa, estresse, ansiedade, depressão e qualidade do sono precisam ser considerados para a reclassificação do RCV, embora sua mensuração possa ser comprometida pelos sintomas próprios da menopausa.^109 , 226^

Adicionalmente, o sedentarismo na pós-menopausa leva a pior condicionamento físico e menor controle cardiometabólico, além de maior incidência de fraturas e de mortalidade. O tabagismo aumenta o risco de menopausa precoce e a probabilidade de DCV, AVC, osteoporose, DM2 e mortalidade por todas as causas.^109 , 226^

Deve-se buscar a estratificação do RCV, seguida de aconselhamento dietético e de estilo de vida. O manejo dos FR cardiometabólico na menopausa deve sempre ser individualizado, com foco em hipertensão, DM2 e dislipidemia. A THM, quando prescrita para o controle dos sintomas da menopausa ou para a prevenção da osteoporose, também pode ter um efeito benéfico, ainda que indireto, sobre os FR cardiometabólico.^109 , 226^

## 9. Distúrbios Cardiometabólicos nas Mulheres

A incidência de DM2 e obesidade vem aumentando consideravelmente nas últimas décadas. Projeções indicam que haverá mais de 600 milhões de pessoas com DM2 em 2045.^251^ Obesidade e DM2 são associados a diversos outros distúrbios cardiometabólicos. Embora ambos os sexos sejam afetados por obesidade e DM2, alguns estudos indicam diferenças entre homens e mulheres quanto a sua prevalência, diagnóstico, tratamento e complicações. As evidências disponíveis sobre essas diferenças não implicam em recomendações terapêuticas e diagnósticas específicas por sexo. No entanto, sua adequada compreensão é importante no manuseio clínico das mulheres.^252 , 253^

### 9.1. Obesidade e Síndrome Metabólica

**Figura 9.1:**
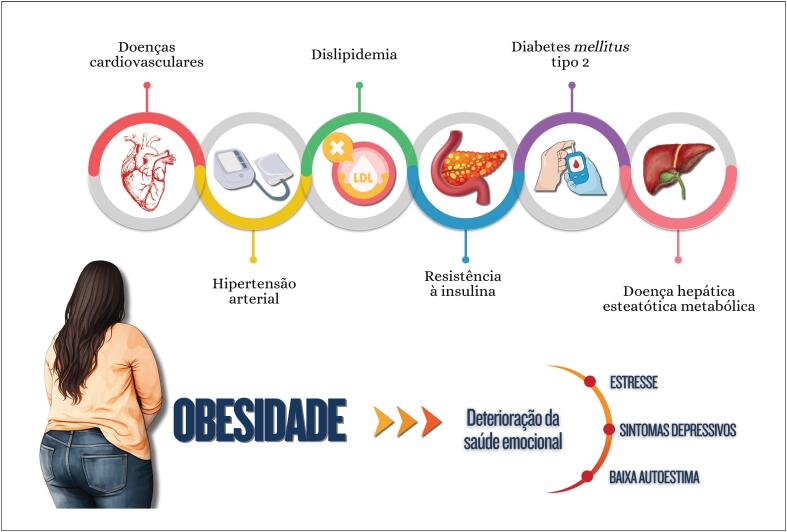
Obesidade e suas consequências cardiometabólicas na mulher.

A obesidade é causa direta ou contribui para o desenvolvimento de diversas condições clínicas, como DM2, DHEM, apneia do sono, doenças articulares, vários tipos de câncer, HAS e IC, além de aumentar o risco de mortalidade cardiovascular.^254^ O aumento de mortalidade cardiovascular está associado a obesidade de forma independente, mesmo nos indivíduos sem outras alterações metabólicas.^255^ O peso corporal é mantido por um balanço entre a energia ingerida e a gasta pelo indivíduo^256 , 257^ e, quando o balanço resulta em excesso de energia, essa é armazenada sob forma de gordura em células adiposas, usualmente no tecido subcutâneo. Existem fatores que limitam o depósito fisiológico de gordura e essa começa a se acumular em outros tecidos, como fígado, pâncreas, rins, músculos e tecido epicárdico, de forma ectópica.^258^ A obesidade tem uma forte carga genética^259 , 260^ e influência dos hábitos de vida; sua prevalência, porém, vem aumentando mundialmente em homens e mulheres.^261^ No Brasil, dados do VIGITEL de 2023 apontam uma prevalência de 24,8% em mulheres e 23,8% em homens^262^ e, se considerados indivíduos com sobrepeso, 59,6% das mulheres e 63,4% dos homens apresentam essa condição ( Figura 9.1 ).

Mulheres usualmente têm maior percentual de gordura corporal e tendência de acúmulo da gordura no tecido subcutâneo dos membros inferiores, enquanto homens apresentam maiores quantidades de gordura visceral.^263^ Essas diferenças na distribuição de gordura corporal são associadas aos hormônios sexuais e variam ao longo da vida reprodutiva da mulher. Assim, mulheres após a menopausa apresentam redistribuição de gordura corporal, com aumento de gordura visceral.^264^ O RCV acompanha essas diferenças hormonais e de composição corporal, ou seja, a proteção cardiovascular classicamente apresentada pelas mulheres durante a menacme desaparece na menopausa.^265 , 266^ Além disso, o ganho de peso nas gestações e sua manutenção no pós-parto são um importante FR para a presença de obesidade.^267^ A SOP está associada à presença de obesidade, de aumento da RI, assim como dos outros componentes da SM.

A definição de obesidade pela Organização Mundial da Saúde leva em consideração o IMC, independentemente do gênero, sendo ainda subdividida em grau 1 (IMC: 30,0–34,99 kg/m²), grau 2 (IMC: 35,0–39,99 kg/m²) e grau 3 (IMC acima de 40,0 kg/m²). Recentemente uma nova classificação de diagnóstico e estadiamento da obesidade foi proposta,^268^ dividindo-a em pré-clínica e clínica e considerando o IMC um método inadequado para seu diagnóstico de forma isolada. Entretanto, essa não foi totalmente aceita pelas sociedades científicas. No nosso entendimento, o IMC é adequado para o diagnóstico de obesidade em indivíduos com IMC acima de 30 kgm/m². Naqueles com IMC mais baixo (abaixo de 30 ou até mesmo abaixo de 25 kg/m²), porém, pode ocorrer excesso de adiposidade e suas consequências metabólicas. Nesses casos é mais importante o uso de outras ferramentas para a identificação do excesso de gordura como as medidas simples da cintura e as razões cintura/quadril e cintura/estatura, como descrito no capítulo 5. As medidas de cintura variam entre homens e mulheres, sendo 88 cm o ponto de corte de cintura para mulheres no Brasil. O ponto de corte da RCQ é 0,85 para mulheres e o da RCE é 0,5, sendo esse último similar para homens e mulheres.^269^ Outra forma mais precisa da medida de adiposidade é a avaliação da composição corporal por bioimpedância ou DEXA ( *dual-energy X-ray absorptiometry* – absorciometria de raios X de dupla energia), mas são necessários estudos que comprovem que seu uso sistematizado altere a conduta e traga benefícios mensuráveis no manejo dos pacientes com obesidade. As medidas antropométricas abordadas no capítulo 5 são importantes para estabelecer o diagnóstico do excesso de adiposidade corporal e estudar a gravidade da doença, além de serem parâmetros de resposta ao tratamento instituído.

### 9.2. Diabetes *Mellitus* Tipo 2

Existem diferenças entre os sexos no que se refere ao diagnóstico e à epidemiologia do DM2, que podem refletir fatores biológicos, sociais e comportamentais. Em geral, homens são diagnosticados mais precocemente e com menores valores de IMC, enquanto mulheres tendem a desenvolver a doença mais tardiamente, com frequência após a menopausa, quando a proteção hormonal dos estrogênios diminui.^270 - 277^ Além disso, há evidências de que as mulheres podem permanecer mais tempo com hiperglicemia subdiagnosticada devido a padrões distintos de sintomas e menor sensibilidade de alguns critérios diagnósticos, como a glicemia de jejum.^278^

A prevalência de DM2 foi estimada em 8,8% da população mundial em 2017, sendo ligeiramente maior em homens do que em mulheres (9,1% *versus* 8,4%).^251^ Apesar dessa diferença, as mulheres com DM2 apresentam maior risco de complicações cardiovasculares, como doença cerebrovascular, e mortalidade precoce, o que pode estar relacionado a desigualdades no acesso a diagnóstico e tratamento.^279^ Fatores socioeconômicos e culturais também influenciam essas disparidades, afetando o rastreamento e a busca por atendimento em mulheres de diferentes regiões. Assim, compreender as diferenças entre os sexos no diagnóstico e na prevalência do DM2 é essencial para implementar estratégias de prevenção e cuidado mais equitativas e eficazes.

Diferenças na fisiopatologia do DM2 envolvendo fatores hormonais, genéticos e metabólicos influenciam a susceptibilidade à doença e sua progressão. Em geral, mulheres apresentam maior RI, especialmente no tecido muscular esquelético, enquanto homens tendem a ter maior deposição de gordura visceral, o que está fortemente associado à disfunção metabólica.^276 , 280^ Após a menopausa, a queda dos níveis de estrogênio em mulheres contribui para o aumento da adiposidade central e piora da sensibilidade à insulina, alterando o perfil inflamatório e lipídico.^281^ Nos homens, a redução da testosterona também se associa à RI, embora com mecanismos distintos, envolvendo menor massa muscular e alterações no metabolismo hepático da glicose.^282^ Além disso, diferenças na expressão de genes relacionados ao transporte de glicose, inflamação e metabolismo energético sugerem uma base biológica para essas disparidades.^280^

Revisões sistemáticas com metanálise demonstraram heterogeneidade entre os sexos na contribuição de cada FR para desfechos cardiovasculares. Enquanto PA, colesterol e IMC parecem contribuir de forma equivalente para o risco de doença coronária e cerebrovascular,^278 , 283 , 284^ o DM2 confere maior risco às mulheres.^285^ O risco de doença coronária é geralmente mais baixo em mulheres, mas, no contexto do DM2, essas diferenças desaparecem.^285^ O DM2 confere às mulheres risco relativo de 44% de doença coronária e 27% de doença cerebrovascular, mas elas apresentam risco absoluto semelhante aos homens com DM2.^286^ Uma maior carga de comorbidades, incluindo o agrupamento de FR, além de questões hormonais e comportamentais, pode contribuir para essa diferença.^287 , 288^

Estudos indicam que as mulheres frequentemente apresentam menor adesão ao tratamento medicamentoso e ao autocuidado, o que pode estar relacionado a barreiras psicossociais, menor apoio social e maior prevalência de depressão.^276 , 289^ Há evidências de que mulheres são menos propensas a atingir metas de controle glicêmico, PA e lipídios, mesmo com tratamento semelhante ao dos homens.^290^ Além disso, diferenças farmacocinéticas e farmacodinâmicas afetam a resposta aos medicamentos antidiabéticos

Devemos ressaltar os aspectos psicossociais e comportamentais do DM2 no sexo feminino. A capacidade cognitiva deve ser monitorada ao longo da vida de todos os pacientes com DM2, sendo o sexo feminino um dos FR para disfunções cognitivas. Depressão também se apresenta com maior frequência em indivíduos com DM2, com predominância no sexo feminino.^291 - 293^ Mulheres com DM2 apresentam maior incidência de disfunção sexual, cuja ocorrência é influenciada tanto por fatores orgânicos (como neuropatia autonômica) como comportamentais.

**Figura 9.2:**
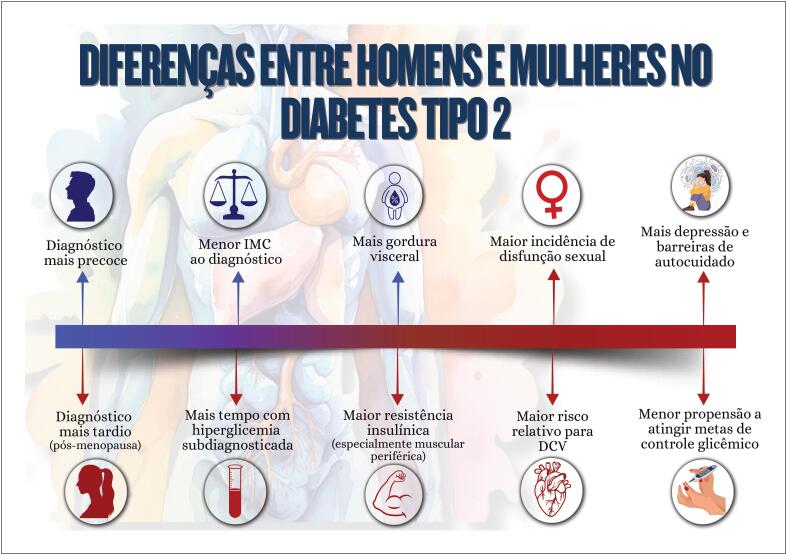
Diferenças no DM2 entre os sexos: diagnóstico mais precoce nos homens e maior risco cardiovascular e psicossocial nas mulheres. IMC: índice de massa corpórea; DCV: doença cardiovascular.

A figura 9.2 sumariza algumas diferenças entre os sexos em relação ao DM2.

### 9.3. Doença Hepática Esteatótica Metabólica

A DHEM é a hepatopatia mais prevalente mundialmente,^294 - 296^ sendo caracterizada pelo acúmulo excessivo de gordura nos hepatócitos. Compreende um espectro de manifestações hepáticas associadas a distúrbios metabólicos e cardiovasculares, como obesidade e/ou distribuição desfavorável de gordura, RI, HAS, dislipidemia e DM2.^297^ A DHEM é reconhecida como a manifestação hepática da SM, estando fortemente associada a DIC, que constitui a principal causa de mortalidade na população com DHEM.^297^

A história natural da DHEM compreende estágios como a esteatose (quando há apenas excesso de gordura no fígado, ultrapassando 5% do parênquima hepático, com mínima inflamação) e a esteato-hepatite (quando há inflamação lobular e balonização de hepatócitos, com ou sem fibrose).^298^ Independentemente do sexo, pessoas com esteato-hepatite metabólica (EHM) podem evoluir com diferentes graus de fibrose, progredir para cirrose e apresentar complicações como hipertensão portal ou carcinoma hepatocelular.^295 , 298^

Estima-se que a DHEM acometa pelo menos 30% da população ocidental.^299^ Em pessoas com excesso de peso e obesidade, a prevalência global da DHEM é de 50,7%^300^ e, naquelas com DM2, a prevalência estimada é de 65,3%.^299^ Embora a obesidade seja mais comum nas mulheres, a DHEM é mais prevalente nos homens, sendo que o risco dessa condição nas mulheres aumenta após a menopausa.^301^ Estima-se que, na população geral, as mulheres tenham um risco 19% menor de esteatose hepática em comparação aos homens; as taxas de esteato-hepatite são semelhantes entre os sexos e as mulheres têm 37% mais risco de fibrose avançada.^302^ Esse maior risco de progressão ocorre especialmente nas mulheres acima de 50 anos, destacando-se a possível participação dos hormônios sexuais na etiopatogenia da DHEM.^302^

Menopausa, história de menarca precoce^303^ e SOP estão associadas ao aumento da suscetibilidade feminina à DHEM.^304^ As mulheres apresentam uma taxa de mortalidade por cirrose mais alta do que os homens, sendo que a DHEM constitui a principal causa de transplante de fígado em mulheres sem carcinoma hepatocelular.^305^

Uma das justificativas para as diferenças na prevalência da DHEM ao longo das fases da vida da mulher consiste na influência dos hormônios sexuais femininos, particularmente o estrogênio, no metabolismo hepático e no padrão de distribuição da gordura corporal.^301^ A ativação do receptor de estrogênio alfa (ERα) no fígado promove redução da síntese, captação e armazenamento de triglicerídeos, ao mesmo tempo que favorece o catabolismo e a exportação de lipídios, efeitos que, em conjunto, protegem as mulheres não menopausadas contra a DHEM.^306^ Além disso, o padrão geoide de distribuição de gordura, caracterizado por maior concentração de gordura gluteofemoral e menor deposição de gordura visceral, constitui a distribuição típica das mulheres no menacme^301^ e está associado a menor risco de DHEM e SM.^295 , 301^ Por outro lado, após a menopausa, o declínio do estrogênio promove redistribuição de gordura, favorecendo a centralização, que tem como consequência um padrão mais androide,^301^ sabidamente associado a DHEM e SM.^295 , 301^

O diagnóstico de DHEM consiste na presença de esteatose hepática associada a pelo menos um critério para SM, na ausência de causas secundárias de esteatose.^295 , 298^ A esteatose pode ser inferida por métodos de imagem tradicionais, como ultrassonografia, tomografia computadorizada e ressonância magnética, que também são capazes de evidenciar sinais de cirrose e hipertensão portal. Entretanto, ressalta-se que esses métodos não são capazes de identificar EHM e fibrose em estágios iniciais.^297^ Por outro lado, a biópsia hepática diferencia com precisão os pacientes com esteatose daqueles com EHM, mas, por se tratar de método invasivo, é reservada sobretudo para situações em que há dúvidas sobre a etiologia da doença hepática.^295 , 297 , 298^

O diagnóstico na DHEM é baseado na identificação de fibrose. Para tal, algumas ferramentas são úteis, como os escores clínico-laboratoriais de risco para fibrose avançada e as elastografias. Dentre esses escores, destaca-se o *Fibrosis-4* (FIB-4), calculado a partir da idade e dos níveis séricos de plaquetas e aminotransferases, sendo que a interpretação do resultado independe do sexo do indivíduo.^295 , 298^ Quando aplicamos o ponto de corte de 1,3, a partir do qual são identificados os pacientes com maior risco de fibrose avançada, esse teste tem alta sensibilidade e excelente valor preditivo negativo.^298 , 307 , 308^ Isso significa, por exemplo, que uma mulher com FIB-4 ≤ 1,3 tem probabilidade muito baixa de apresentar fibrose avançada.

As elastografias (ultrassônicas e por ressonância) são métodos capazes de estimar a rigidez do fígado e, consequentemente, a presença e a quantificação da fibrose, incluindo seus estágios iniciais. A elastografia transitória pelo método de Fibroscan^®^ é a mais validada na literatura e recomendada por diretrizes nacionais^295 , 298 , 307 , 308^ e internacionais.^309^ Recomenda-se que a DHEM seja rastreada ativamente nas pessoas sob maior risco de acúmulo de gordura no fígado e de progressão para formas mais graves da doença hepática, visando identificar aquelas com fibrose significativa. Fazem parte dos grupos de risco as mulheres na pós-menopausa, as pessoas com alterações da homeostase glicêmica (pré-diabetes ou DM2), excesso de peso, SM ou história familiar positiva para cirrose e carcinoma hepatocelular. Uma forma racional de realizar o rastreamento populacional é calcular o FIB-4 nas pessoas sob risco e, naquelas com escore maior do que 1,3, realizar a elastografia.^295 , 298 , 309^

Cabe ressaltar que a avaliação isolada dos níveis séricos de aminotransferases tem baixa acurácia para identificar pacientes com EHM e fibrose, mas a elevação dessas enzimas indica a necessidade de rastreio de outras doenças hepáticas, com destaque para as hepatites virais crônicas e a hepatopatia alcoólica (considera-se excessivo o consumo alcoólico acima de 20 g/dia para as mulheres e acima de 30 g/dia para os homens).^295 , 298 , 310 - 321^

### 9.4. Doença Renal Crônica

A DRC, com destaque para a doença renal diabética (DRD), é um problema de saúde pública mundial que afeta milhões de pessoas. Neste tópico, abordaremos resumidamente as diferenças potenciais entre os gêneros, sendo as mulheres uma população de especial interesse devido às suas características biológicas e sociais.

A DRC é definida como um dano renal ou redução da função renal por um período superior a três meses, que pode progredir para a necessidade de terapia renal substitutiva. A evolução da DRC é frequentemente insidiosa. Estima-se que 425 milhões de pessoas sejam diabéticas, sendo que cerca de 30% daquelas com DM1 e 40% daquelas com DM2 desenvolverão DRC em diferentes estágios.^322^ O DM é a principal causa mundial de terapia renal substitutiva, sendo a HAS a segunda,^323^ e sua incidência vem aumentando.^324^ Dados norte-americanos mostram maior prevalência de DRD em mulheres (14,8% x 12,6% em homens).^325^ Apesar disso, parece que os homens têm maior risco para agudização de DRC e progressão para estágios avançados da doença comparados às mulheres.^326 - 328^ Esses dados são heterogêneos na literatura e podem estar relacionados a alterações hormonais, como na menopausa, à duração e idade de início do DM2 e a critérios utilizados para o diagnóstico.^329^ Estudos mostram maior velocidade de perda da taxa de filtração glomerular estimada em mulheres mais idosas e menopausadas, assim como inconsistência dos resultados relacionados à THM e às alterações na função renal.^330^

Vários estudos mostraram que os homens têm maior probabilidade para desenvolver DRD albuminúrica comparados às mulheres, nas quais a forma mais comum é a não albuminúrica. Em uma grande coorte da Itália, 29,8% dos homens apresentavam albuminúria moderada a grave em comparação com 18,3% das mulheres.^331^

Apesar da descrição de diferenças entre homens e mulheres quanto à hemodinâmica renal no DM e nos mecanismos associados, como por exemplo uma maior ocorrência de vasoconstricção da arteríola eferente do glomérulo no sexo feminino, sua significância clínica permanece incerta.^332 - 337^

Os dados de literatura atuais não permitem a utilização de critérios diferentes entre homens e mulheres para triagem, seguimento e tratamento da DRD. Assim, devemos utilizar as seguintes recomendações para ambos os sexos:^323^

1. Sugere-se que o primeiro rastreamento da DRD seja feito com amostra de urina aleatória para determinação da razão albumina/creatinina e estimativa da taxa de filtração glomerular determinada pela creatinina sérica, a partir da equação CKD-EPI de 2021 ou a equação de Schwartz para crianças. No DM2, o rastreamento deve ser iniciado no momento do diagnóstico. No DM1, deve começar a partir da puberdade ou dos 10 anos de idade, em pacientes com pelo menos 5 anos de diagnóstico, sendo repetido anualmente. Todo teste anormal da razão albumina/creatinina deve ser confirmado em, pelo menos, duas de três amostras repetidas no período de três a seis meses por causa da variabilidade diária.
2. Em indivíduos com DM1 ou DM2, sugere-se buscar a meta de HbA1c de 6,5-7% quando a taxa de filtração glomerular estimada for maior que 60 mL/min/1,73 m² e a razão albumina/creatinina maior que 30mg/g, para reduzir a progressão da albuminúria e da DRD a longo prazo. Deve-se manter a meta de HbA1c de 7-7,9% em indivíduos com DM1 ou DM2 quando a taxa de filtração glomerular estimada for menor que 45 mL/min/1,73 m² ou o paciente estiver em diálise, para evitar excesso de mortalidade.

Concluindo, considerando as diferenças descritas entre homens e mulheres, bem como as interações observadas com a presença de diabetes com ou sem DRD, é provável que os hormônios sexuais contribuam para as diferenças entre os sexos na fisiopatologia do início e da progressão da DRD.

## 10. Estratégias para Abordar os Distúrbios Cardiometabólicos nas Mulheres

### 10.1. Medidas Não Farmacológicas

A crescente prevalência de distúrbios cardiometabólicos em mulheres representa um dos maiores desafios da saúde pública, dado o forte vínculo entre obesidade e DCV. Globalmente, obesidade é responsável por cerca de 4,7 milhões de mortes anuais.^338 , 339^ O acúmulo de gordura abdominal contribui para alterações metabólicas, inflamação crônica e RI, favorecendo aterosclerose, IM e AVC. No Brasil, as DCV somam aproximadamente 28% dos óbitos femininos, conforme dados do Ministério da Saúde e da Organização Mundial de Saúde.^340 , 341^

No Brasil, 24,8% das mulheres apresentam obesidade e 38,7% apresentam sobrepeso, totalizando mais de 63% das mulheres com peso acima do recomendado.^262^ O estudo ELSA-Brasil revelou taxas elevadas de obesidade abdominal entre mulheres pretas (62%) e pardas (59,5%), com prevalência geral de sobrepeso de 61,8%.^342 , 343^ Esses dados demonstram a urgência em investimentos e intervenções que ajudem a reverter esses processos patológicos.

### 10.2. Intervenções Nutricionais

A reeducação nutricional é central na prevenção dos distúrbios cardiometabólicos, principalmente quando personalizada conforme as condições e os hábitos das pacientes. Protocolos que reduzem carboidratos simples e gorduras saturadas, ao mesmo tempo que aumentam o consumo de fibras, têm mostrado eficácia na melhora da glicemia, dislipidemia e RI. A dieta com padrão mediterrâneo é amplamente reconhecida por seus efeitos protetores.^344^

No Brasil, essa abordagem pode ser adaptada com alimentos locais como azeite de oliva, castanhas-do-pará, frutas, como mamão e abacate, peixes de água doce e leguminosas, como feijão e grão-de-bico. O guia alimentar para a população brasileira reforça o consumo de alimentos *in natura* e o respeito às culturas alimentares regionais.^345^ A adesão a planos nutricionais supervisionados por profissionais especializados resulta não apenas em perda de peso, mas também em melhorias sustentáveis nos parâmetros metabólicos, contribuindo para prevenção de DCV.^346^ A personalização da abordagem permite que as preferências culturais e regionais sejam respeitadas, pois são fatores relevantes no Brasil, dada a grande variação de hábitos alimentares no país.^109^

### 10.3. Atividade Física

A prática regular de atividade física desempenha papel complementar essencial no tratamento dos distúrbios cardiometabólicos, principalmente durante a transição menopausal e a menopausa. A combinação de exercícios aeróbicos com treinamento resistido promove melhorias significativas na composição corporal, na sensibilidade à insulina e no controle glicêmico.^347^ Adicionalmente, contribui para a redução da PA, melhora do perfil lipídico e redução da inflamação sistêmica.^348^

A associação de planos nutricionais individualizados à prática regular de atividade física potencializa os efeitos benéficos, favorecendo a função cardiovascular e diminuindo a morbimortalidade.^139^ Ademais, também está associada a menor incidência de HAS, dislipidemia e DM2, além de reduzir o risco de desenvolvimento de depressão e demência e melhorar a densidade mineral óssea e a qualidade do sono.^139 , 349^

A recomendação atual é de pelo menos 150 minutos semanais de atividade aeróbica de intensidade moderada ou 75 minutos de atividade vigorosa, associados a exercícios de resistência muscular em pelo menos dois dias por semana.^139 , 350^ A prescrição deve ser individualizada, considerando o condicionamento físico, limitações funcionais e o contexto de vida da mulher, garantindo adesão e efeitos sustentáveis a longo prazo.^109 , 209^ Dados recentes indicam que, quando mulheres praticam a mesma quantidade de atividade física que os homens, os benefícios em termos de redução da mortalidade total e cardiovascular são mais pronunciados nelas do que em homens.^351^

### 10.4. Intervenções Psicossociais

O estresse psicossocial, incluindo fatores como solidão, perdas significativas e transtornos mentais, contribui diretamente para o RCV, prejudicando a adesão ao tratamento e favorecendo comportamentos de risco como tabagismo e sedentarismo.^352^ Mulheres que participam de programas integrados de suporte psicológico apresentam melhora significativa no estilo de vida, resultando em melhores desfechos clínicos, como redução de peso e controle glicêmico e do perfil lipídico.^353^ Estratégias, como a terapia cognitivo-comportamental, são especialmente eficazes nesse contexto, com estudos indicando sua capacidade de reduzir sintomas de ansiedade e depressão, melhorar a qualidade de vida relacionada à saúde cardiovascular e aumentar a adesão ao tratamento.^353^

A terapia cognitivo-comportamental está associada a menor taxa de readmissões hospitalares e melhora da autopercepção de saúde em mulheres com DCV.^354^ Ademais, a terapia cognitivo-comportamental aplicada ao gerenciamento de estresse foi associada a mudanças sustentáveis no comportamento, maior frequência de atividade física, alimentação saudável e abandono do tabagismo.^354^

### 10.5. Tabagismo e Consumo de Álcool

O tabagismo é importante fator inflamatório crônico, sobretudo acentuado em mulheres na pós-menopausa.^355^ O cigarro eletrônico, em particular, representa nova ameaça, especialmente entre jovens e gestantes, que muitas vezes consideram esses dispositivos menos prejudiciais.^356^ Seu uso pode levar a níveis de nicotina até seis vezes superiores aos dos cigarros convencionais, aumentando o risco de dependência, estresse oxidativo, disfunção endotelial e inflamação vascular.^356^ Da mesma forma, o consumo de álcool afeta significativamente a saúde feminina e está associado à maior prevalência de SM, dislipidemias e hiperinsulinemia.^357^

A redução estrogênica da menopausa aumenta a suscetibilidade inflamatória e aterosclerótica, disfunção endotelial, rigidez arterial e alterações lipídicas, contribuindo para o estado pró-aterogênico e pró-coagulante.^358^ A associação do fator inflamatório do tabagismo na fase da pós-menopausa acelera o processo aterosclerótico.

### 10.6. Condições Clínicas Específicas

A SOP e a endometriose estão frequentemente associadas a desequilíbrios hormonais que exacerbam a RI, aumentam o risco do desenvolvimento de DM2 e promovem inflamação subclínica.^355^ Na SOP, a desregulação hormonal afeta o metabolismo das glicoproteínas e intensifica o armazenamento de gordura, contribuindo para um perfil lipídico desfavorável e elevando o risco de DCV.^359^ Na endometriose, a inflamação crônica associada à presença ectópica do tecido endometrial agrava os sintomas locais e impacta negativamente o ambiente sistêmico, afetando os mecanismos de regulação metabólica e vascular.^360^

Concluindo, as intervenções não farmacológicas têm demonstrado clara eficácia no manejo dos FRCV.^139^ A combinação de atividade física regular com intervenções nutricionais para controle do peso, além de suporte psicossocial, resulta em melhorias expressivas nos parâmetros vasculares e metabólicos.^361^

A atuação multidisciplinar tem se mostrado eficaz, permitindo a personalização e a adaptação das estratégias às necessidades individuais, gerando mudanças sustentáveis no estilo de vida e reduzindo a morbimortalidade por distúrbios cardiometabólicos.^361^

### 10.7. Estratégias Farmacológicas

#### 10.7.1. Tratamento da Hipertensão Arterial Sistêmica

A hipertensão constitui um dos FR mais prevalentes para DCV em mulheres, incide em todas as fases da vida e tem aumento progressivo com o avançar da idade.^362^ A escolha e a condução das estratégias farmacológicas devem considerar as fases do ciclo reprodutivo, incluindo perimenopausa e pós-menopausa. Determinadas condições associadas à HAS secundária apresentam maior prevalência entre mulheres, como a doença renovascular decorrente de displasia fibromuscular, a síndrome de Cushing de origem endógena, além dos distúrbios tireoidianos e paratireoidianos.^363^

- Inibidores da enzima conversora de angiotensina e bloqueadores do receptor da angiotensina: Apresentam eficácia comprovada na redução dos níveis pressóricos. Oferecerem benefícios adicionais, como nefroproteção e regressão da hipertrofia ventricular esquerda. São recomendados como primeira linha, especialmente em mulheres com diabetes, DRC na presença de albuminúria, insuficiência cardíaca com fração de ejeção reduzida, ou DIC, além de poderem ser utilizados com segurança em pacientes sem comorbidades.^214 , 364^ O uso de inibidores da enzima conversora de angiotensina (IECA) e bloqueadores dos receptores da angiotensina II (BRA) em mulheres em idade fértil exige cautela devido ao risco teratogênico, sendo fundamental excluir a possibilidade de gestação antes do início do uso e garantir a contracepção durante a terapia.^214^
- Bloqueadores de canais de cálcio: Amplamente recomendados no manejo da HAS em mulheres, são uma opção terapêutica viável durante os períodos de perimenopausa e pós-menopausa em pacientes sem comorbidades significativas.^214^ É importante destacar que os bloqueadores de canais de cálcio podem estar associados à piora dos SVM, como ondas de calor e sudorese noturna em mulheres na menopausa. Essas medicações podem intensificar tais sintomas, com impacto negativo na qualidade de vida.^214^
- Diuréticos tiazídicos: eficazes no controle da HAS em mulheres com sobrepeso ou obesidade, especialmente quando há edema associado.^214^ Entre os principais representantes, a indapamida destaca-se por seu efeito vasodilatador adicional e ação prolongada, proporcionando controle pressórico sustentado e menor impacto metabólico em comparação à hidroclorotiazida.^364^ Essa, embora amplamente utilizada e acessível, tem eficácia menos duradoura e maior risco de distúrbios eletrolíticos e metabólicos.^364^ A escolha deve ser individualizada, considerando-se o perfil clínico e os objetivos terapêuticos.
- Espironolactona: antagonista da aldosterona, é eficaz na **HAS resistente** , quando há maior ativação do sistema renina-angiotensina-aldosterona.^214^ Também é indicada em casos de hiperaldosteronismo primário e na SOP, devido à sua ação antiandrogênica.^214 , 364^ Apresenta perfil metabólico favorável, com baixo impacto sobre glicemia e lipídios, relevante em mulheres com RCV aumentado.^214^ É necessário monitorar a função renal e os níveis de potássio, sobretudo em pacientes idosas ou com DRC, devido ao risco de hipercalemia.^364^

Os distúrbios hipertensivos da gestação foram discutidos no capítulo 7, bem como sua abordagem terapêutica. Na Figura 10 , salientamos as implicações desses distúrbios ao longo do ciclo de vida das mulheres.

**Figura 10.1:**
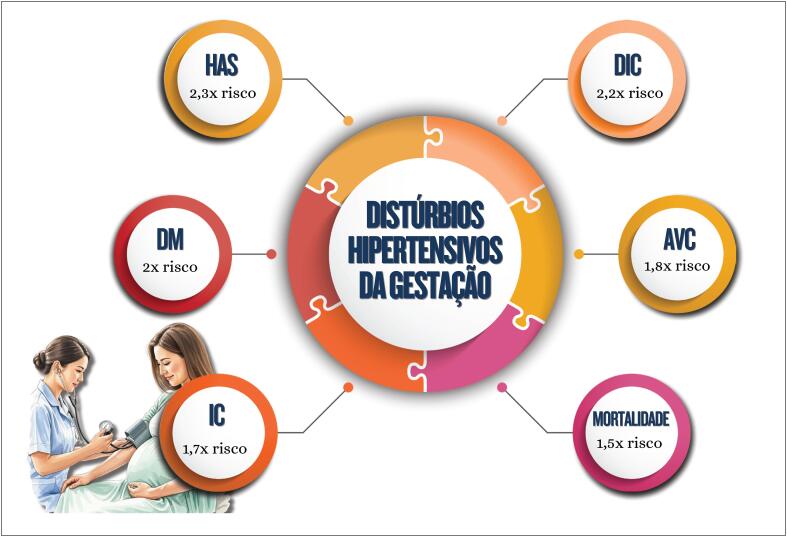
Implicações dos distúrbios hipertensivos da gestação ao longo do ciclo de vida das mulheres. Fonte: Davis et al.^365^ HAS: hipertensão arterial sistêmica; DIC: doença isquêmica do coração; AVC: acidente vascular cerebral; IC: insuficiência cardíaca; DM: diabetes mellitus.

#### 10.7.2. Manejo das Dislipidemias

A dislipidemia é particularmente marcante na transição da menopausa. A queda dos níveis de estrogênio está relacionada a alterações metabólicas relevantes como RI, redistribuição de gordura corporal com predomínio de acúmulo abdominal e alterações no perfil lipídico.^366^ A menopausa associa-se a elevações significativas de colesterol total, LDL-c, apolipoproteína B, triglicerídeos e Lp(a), além da possível redução do efeito protetor antiaterogênico do HDL-c.^109^ Valores de LDL-c ≥ 130 mg/dL são considerados elevados, enquanto níveis de HDL-c < 50 mg/dL representam FR adicional para DCV, especialmente quando associados a outros componentes da SM.^109^ Esse aumento do RCV deve ser considerado na abordagem terapêutica individualizada, devendo o tratamento da dislipidemia ser baseado não apenas nos valores de LDL-c, HDL-c e triglicerídeos, mas também no perfil de RCV global da paciente.^109 , 212^

#### 10.7.3. Hipolipemiantes Orais

As estatinas são a terapia de primeira linha na dislipidemia, eficazes na redução de eventos cardiovasculares e regressão da aterosclerose em mulheres de alto risco, com benefícios comparáveis aos observados em homens.^367^ Devem ser indicadas tanto na prevenção primária quanto secundária, com metas de LDL-c definidas conforme o RCV: < 100 mg/dL para risco intermediário, < 70 mg/dL para alto risco e < 50 mg/dL para risco muito alto.^367 , 368^ Em mulheres em idade fértil, o uso deve ser individualizado em casos de alto risco, sendo contraindicado durante a gestação e lactação.^369^

A ezetimiba, quando associada às estatinas, potencializa a redução do LDL-c e contribui na regressão da aterosclerose e melhora da função endotelial, mantendo segurança e eficácia semelhantes entre os sexos.^367^ É particularmente útil em mulheres com intolerância a doses elevadas de estatinas ou com resposta insuficiente à monoterapia.

Apesar de níveis de HDL-c acima de 50 mg/dL serem desejáveis em mulheres, a prioridade terapêutica permanece na redução do LDL-c.^367^ A hipertrigliceridemia, frequente especialmente na menopausa, deve ser tratada quando os triglicerídeos ultrapassam 200 mg/dL e obrigatoriamente acima de 500 mg/dL.^367^ O fenofibrato é a opção preferencial entre os fibratos, por seu perfil de segurança e benefícios metabólicos adicionais em mulheres.^109^

Inibidores da PCSK9, como alirocumabe, evolocumabe e inclisirana, são indicados para pacientes com LDL-c elevado que não atingem metas com estatinas ou são intolerantes a elas, incluindo casos de hipercolesterolemia familiar.^368^ A inclisirana, um silenciador de RNA, destaca-se pela posologia semestral após a dose de indução, com boa adesão e eficácia sustentada. Essas medicações não devem ser usadas durante a gestação ou lactação e apresentam eficácia semelhante em homens e mulheres na redução de eventos cardiovasculares.^368^

**Figura 10.2:**
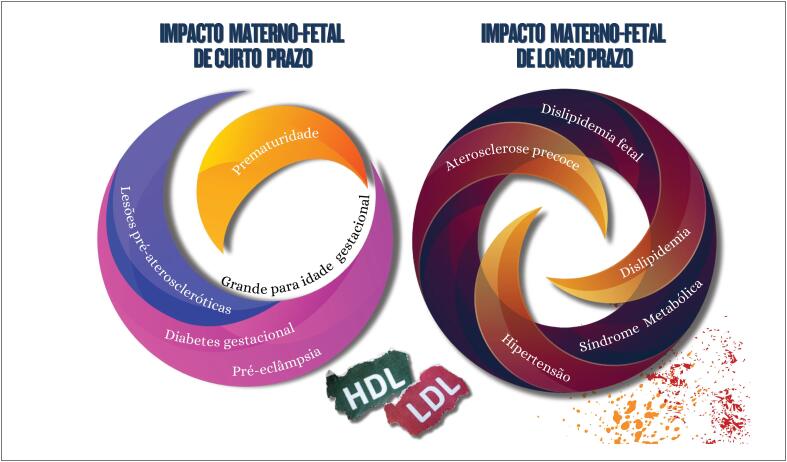
Impactos materno e fetal associados a perfis lipídicos aterogênicos na gestação.

**Figura 10.3:**
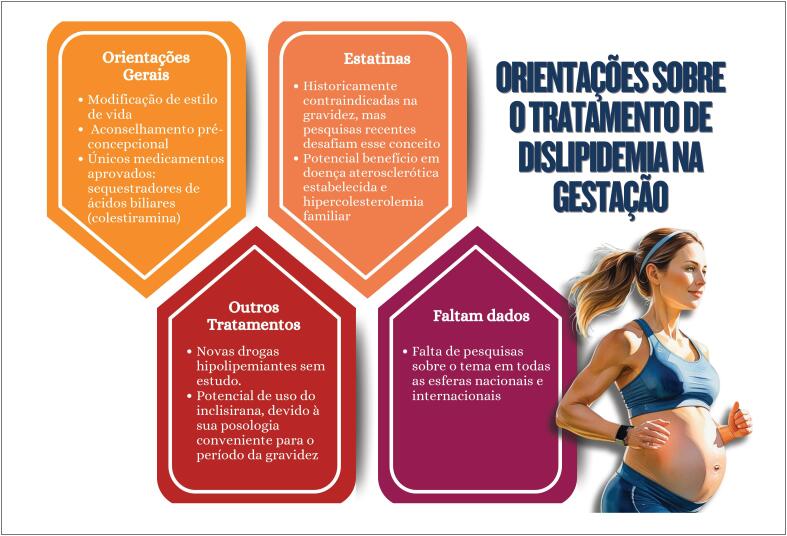
Recomendações para a abordagem das dislipidemias na gestação.

Os impactos materno e fetal associados a perfis lipídicos aterogênicos na gestação foram descritos no capítulo 7 e estão resumidos na Figura 10.2. As recomendações para a abordagem das dislipidemias na gestação são demonstradas na Figura 10.3.

#### 10.7.4. Controle do Diabetes *Mellitus*

Por mais que as evidências disponíveis não sejam suficientes para recomendações terapêuticas específicas para o sexo feminino, devem-se levar em conta fatores hormonais, comportamentais e sociais específicos de cada sexo no manejo do DM2 e de suas complicações. Além disso, é fundamental promover a conscientização sobre essas diferenças entre profissionais de saúde e pacientes a fim de garantir um atendimento mais equitativo e eficaz.

Portanto, o manejo do DM2 em mulheres exige abordagem abrangente, contemplando a heterogeneidade clínica e metabólica. O objetivo central é atingir metas específicas que reduzam a morbimortalidade cardiovascular e metabólica. Entre as mais relevantes estão: HbA1c abaixo de 7%, além do controle do peso corporal e da circunferência abdominal, fatores intimamente ligados a RI e RCV.^369^

Mulheres apresentam progressão distinta do pré-diabetes para DM2, frequentemente associada a maiores índices de obesidade e risco aumentado de complicações metabólicas. Diante disso, o manejo deve contemplar não apenas o controle glicêmico, mas também estratégias específicas para reduzir a RI e prevenir comorbidades associadas.^109 , 209^

Em relação aos fármacos usados no tratamento do DM2, algumas considerações merecem ser abordadas: mulheres podem apresentar maior risco de hipoglicemia com sulfonilureias e resposta distinta às glitazonas, relacionada à função renal e composição corporal.^182^ Por outro lado, os homens podem apresentar maior resposta à perda de peso induzida por inibidores do SGLT2, em parte devido à diferença na distribuição de gordura corporal. Além disso, os inibidores do SGLT2 estão associados a maior taxa de infecções fúngicas genitais, especialmente em mulheres.^291^ Quanto ao tratamento com análogos de GLP-1, especial cuidado deve ser tomado em mulheres em uso de contracepção oral. A Associação Americana de Diabetes recomenda que mulheres em uso dessa classe de medicamento devem optar por um método anticoncepcional não oral ou adicionar um método de barreira durante as primeira quatro semanas do uso de tirzepatida (análogo de GLP-1), devido aos seus efeitos sobre esvaziamento gástrico, tendo potenciais consequências sobre a farmacocinética do anticoncepcional oral.^292^ Outras considerações merecem destaque, conforme descrito abaixo, porém não implicam em recomendações terapêuticas específicas por sexo.

- Metformina: permanece como a primeira escolha no tratamento do DM2, especialmente em casos assintomáticos, na SOP, em casos sem evidências de DCV ou renal estabelecida e naquelas com baixo RCV global.^369^
- Inibidores do SGLT2: demonstram benefícios importantes na redução da glicemia, mas também oferecem proteção cardiovascular e renal em mulheres com RCV elevado, ICFEp e insuficiência cardíaca com fração de ejeção reduzida, e DRC associada a albuminúria.^369^
- Pioglitazona: uma tiazolidinediona particularmente útil em mulheres com RI, como na SOP, e pré-diabetes ou DM2 com perfil metabólico aterogênico. Além de melhorar a sensibilidade à insulina, apresenta benefícios sobre o perfil lipídico e a inflamação vascular. No entanto, seu uso deve ser criterioso, considerando riscos como ganho de peso, perda de massa óssea e retenção hídrica, especialmente em mulheres com alto risco de IC.^369^

#### 10.7.5. Manejo da Obesidade

O manejo da obesidade é desafiador, complexo e idealmente deve ser realizado por equipe multiprofissional. O tratamento deve considerar minimizar o estigma relacionado à doença, sendo esse aspecto merecedor de especial atenção em mulheres devido à sua maior vulnerabilidade.^214^ É importante estabelecer com a paciente alvos terapêuticos factíveis e associados a benefícios clínicos, podendo-se utilizar estratégias de abordagem para perda de peso, como as usadas no combate ao tabagismo (5 As, do inglês *ask, assess, advice, agree, assist).* ^271^ A Associação Brasileira para o Estudo da Obesidade e Síndrome Metabólica (ABESO) propôs uma classificação da obesidade com base no peso corporal ao longo da vida e metas terapêuticas derivadas dessa avaliação, tendo assim como alvo a proporção de perda de peso em relação ao IMC máximo da vida, não sendo recomendado o alvo pouco realístico de normalização do IMC para < 25 kg/m^2^.^252^ Os alvos estão apresentados na Tabela 10.1.

**Tabela 10.1:** Classificação proposta de obesidade "reduzida" e "controlada" com base no IMC máximo

IMC Máximo	Inalterado *	Reduzido *	Controlado *
30–40 kg/m²	< 5%	5–9,9%	> 10%
40–50 kg/m²	< 10%	10–14,9%	> 15%

* Reduções percentuais no peso corporal a partir do IMC máximo alcançado.^252^

Como visto acima, as medidas não farmacológicas, como dieta com restrição calórica, prática de exercícios físicos e programas que incluem abordagem psicossocial, são fundamentais no manejo das doenças metabólicas, notadamente da obesidade. A restrição calórica é mais relevante para a redução do peso, mas a prática de exercícios físicos é fundamental para a manutenção da perda de peso e prevenção da perda de massa muscular que usualmente acompanha o emagrecimento.^271^ No entanto, os benefícios das medidas não farmacológicas tendem a diminuir com o tempo,^272^ não sendo encontrados benefícios em desfechos cardiovasculares em longo prazo com essa intervenção, tanto em homens quanto em mulheres. Uma análise de subgrupo do estudo *Look Ahead* com pacientes que apresentaram redução de pelo menos 10% do peso no primeiro ano mostrou redução do desfecho primário (eventos cardiovasculares maiores e internação por angina) em 20%.^273^

Existem diversos tratamentos farmacológicos para obesidade com efeitos variáveis sobre peso corporal, porém apenas tratamentos mais recentes se associaram à redução de desfechos. O estudo SELECT foi o primeiro a demonstrar benefícios cardiovasculares nesse cenário.^274^ O uso do agonista do GLP-1 semaglutida, por via subcutânea na dose semanal de 2,4 mg em pacientes com sobrepeso e DCV estabelecida por aproximadamente 3 anos, reduziu o peso corporal em 8,51% e resultou em diminuição do risco de eventos cardiovasculares maiores em 28%. A análise de subgrupo das mulheres incluídas (n = 4.872, 28% da amostra) mostrou redução de HR 0,84 (IC 95%: 0,66–1,07), enquanto os homens apresentaram 0,79 (IC 95%: 0,70–0,90), sem relato se houve resultado estatisticamente significativo entre os grupos na análise. As diferenças numéricas encontradas provavelmente são relacionadas ao menor poder da análise do subgrupo das mulheres pelo reduzido tamanho amostral. Vários outros medicamentos antiobesidade mais potentes, apresentando redução de peso corporal de até 25%, estão sendo estudados.^275^ No entanto, os resultados dos estudos de fase 3 com desfechos cardiovasculares ainda não estão disponíveis.

#### 10.7.6. Manejo da Doença Hepática Esteatótica Metabólica

O tratamento da DHEM consiste em modificação do estilo de vida com foco na redução de pelo menos 5% do peso corporal, uma vez que a perda de peso é a medida mais eficaz na melhora histológica da DHEM.^295 , 298^ O consumo de bebidas alcoólicas deve ser limitado, bem como a ingestão da frutose utilizada no preparo de alimentos ultraprocessados e bebidas açucaradas. Além da modificação do estilo de vida, mulheres com formas mais avançadas da DHEM podem se beneficiar de terapias farmacológicas que atuam sobre a EHM e/ou fibrose hepática. São exemplos desses medicamentos: resmetirom, semaglutida, pioglitazona, tirzepatida, vitamina E e inibidores do SGLT2.

O resmetirom, um agonista seletivo do receptor β do hormônio tireoidiano (THR- β), foi o primeiro medicamento a obter a aprovação de uma agência regulatória para o tratamento da EHM não cirrótica, com fibrose moderada a avançada (compatível com os estágios F2 e F3). No MAESTRO-NASH,^310^ estudo de fase 3 que incluiu 966 participantes com EHM tratados por 52 semanas (322 no grupo de 80 mg de resmetirom, 323 no grupo de 100 mg de resmetirom e 321 no grupo placebo), a medicação foi associada à melhora da fibrose em comparação com placebo. Não houve análise de subgrupos referentes ao sexo. A semaglutida, um agonista do receptor do GLP-1, também promoveu melhora de resultados histológicos em uma população com EHM e fibrose F2-F3. Na análise interina do ESSENCE,^311^ estudo de fase 3 incluindo 800 participantes tratados por 70 semanas (57,1% eram mulheres e 55,9% com DM2) houve melhora significativa da EHM sem piora da fibrose e redução significativa da fibrose sem piora da EHM (desfechos primários) com a semaglutida na dose semanal de 2,4 mg *versus* placebo.

A pioglitazona, um agonista seletivo do receptor gama ativado por proliferador de peroxissoma (PPARγ), é recomendada como tratamento para EHM e/ou fibrose em pessoas com DM2,^295 , 298 , 308^ uma vez que a maioria dos estudos que comparam a pioglitazona com placebo demonstra benefícios na inflamação e nas alterações histológicas.^312^ Não houve, porém, subanálise referente ao sexo na maioria deles. Importante salientar que a medicação pode piorar sintomas de IC, em função de retenção hídrica e, especificamente nas mulheres, a pioglitazona está associada ao aumento de risco de fraturas ósseas e ganho de peso.^312 , 313^

A tirzepatida, um co-agonista dos receptores do polipeptídeo insulinotrópico dependente de glicose (GIP) e do GLP-1 já aprovado em alguns países como agente antidiabético e antiobesidade, também foi testada no tratamento das pessoas com EHM e fibrose F2-F3. No SYNERGY-NASH,^314^ um estudo de fase 2b controlado por placebo, a medicação promoveu resolução da EHM sem piora da fibrose em 61% dos indivíduos avaliados após 52 semanas de tratamento (57% dos pacientes eram mulheres, porém não foi realizada subanálise nesse grupo).

No estudo PIVENS,^315^ conduzido em pessoas com EHM e sem DM2, o uso da vitamina E (800 UI/dia) por dois anos promoveu melhora da atividade da doença hepática, estimada na histologia pelo índice NAS ( *Non-Alcoholic Steatohepatitis Activity Score* ), sem aumento na fibrose em comparação ao placebo (43% vs. 19%; p < 0,001). Por fim, embora estudos de desfechos histológicos relacionados à DHEM com os inibidores do SGLT2 sejam escassos, há evidências de redução de enzimas hepáticas, de gordura hepática e de rigidez hepática aferida por elastografia com esses agentes na população com DM2.^316 - 318^ Assim, algumas diretrizes sobre o manejo da DHEM preconizam que o tratamento com inibidores do SGLT2 deve ser considerado em pessoas com DM2 que apresentam EHM e/ou fibrose, sem diferenças entre homens e mulheres em relação aos desfechos.^295 , 298 , 308^

Nos casos de insucesso da combinação de modificação do estilo de vida e farmacoterapia, pessoas com DHEM associada a fibrose e obesidade a partir de classe II devem ser consideradas para cirurgia bariátrica.^319^ Os benefícios dessa cirurgia para a DHEM são consistentes em diversos estudos avaliando técnicas operatórias distintas e, embora a redução da EHM já seja evidente ao final do primeiro ano pós-operatório, benefícios significativos na fibrose demandam mais tempo, como demonstrado em estudos com tempos de seguimento de 5 a 6 anos.^320 , 321^

#### 10.7.7. Manejo da Doença Renal Crônica

Os dados atuais da literatura não permitem que utilizemos critérios diferentes para triagem, diagnóstico e tratamento da DRC, em especial a DRD, e, portanto, orientamos que os critérios da Sociedade Brasileira de Diabetes sejam utilizados. Mais pesquisas são necessárias para identificar diferenças fisiológicas clinicamente relevantes entre os sexos, com o objetivo de identificar novas terapias que modifiquem os desfechos clínicos.

As diferenças na albuminúria entre homens e mulheres podem influenciar o tratamento, com terapias direcionadas para reduzir a proteinúria, incluindo bloqueio do sistema renina-angiotensina-aldosterona, inibidores do SGLT2 e antagonistas dos receptores de mineralocorticoides.^331^

Os grandes ensaios clínicos randomizados utilizando os inibidores do SGLT2 mostraram que homens e mulheres parecem ter benefícios iguais, embora apenas 28,5% a 36,9% dos participantes fossem mulheres nesses estudos. Uma análise agrupada dos ensaios EMPA-REG OUTCOME, CANVAS, DECLARE TIMI-58 e CREDENCE mostrou que não houve diferença na redução de eventos cardiovasculares maiores ou de eventos adversos dos inibidores do SGLT2, incluindo amputação, fratura e infecção do trato urinário, entre homens e mulheres, porém as mulheres são mais propensas a infecção genital.^370^

Apesar do benefício igual dos inibidores do SGLT2 independentemente do sexo, mulheres são menos propensas a receber prescrição de um inibidor do SGLT2, mesmo com diagnósticos de DRD, insuficiência cardíaca com fração de ejeção reduzida ou DCV aterosclerótica, privando assim muitas mulheres dos benefícios cardiovasculares e renais.

A Tabela 10.2 apresenta um resumo relacionada às classes de tratamento e diferenças em relação ao sexo.

**Tabela 10.2:** Diferenças baseadas no sexo em terapias para doença renal diabética

Terapia	Diferenças Baseadas no Sexo
Inibidores do SGLT2	Nenhuma diferença no benefício cardiovascular ou renal. Risco aumentado de cetoacidose diabética em mulheres com DM1 em uso off-label, talvez relacionado a maior cetogênese em mulheres. Risco aumentado de infecções micóticas genitais em mulheres.
Agonistas do receptor GLP-1 e inibidores da dipeptidil peptidase-4	Nenhuma diferença entre os sexos. Diferença teórica de sexo na resposta vascular ao óxido nítrico.
Antagonistas do receptor de mineralocorticoide	Nenhuma diferença entre os sexos em ensaios clínicos randomizados.
Antagonistas do receptor de endotelina	Diferenças baseadas no sexo na expressão do receptor de endotelina. Nenhuma diferença em estudos clínicos randomizados.
Metformina	Maiores efeitos adversos gastrointestinais em mulheres, com potencial melhora em menores doses.
Bloqueio do RAAS	Em mulheres, o estrogênio diminui a renina, ECA, Ang II e aumenta angiotensinogênio e Ang1-7. Contraindicado na gestação.
Terapia hormonal na menopausa	Estradiol, progesterona e terapia hormonal combinada reduzem a albuminúria em mulheres na pós-menopausa. Dados controversos.

Adaptado de Sridhar et al.^338^ Ang1-7: angiotensina 1-7; Ang II: angiotensina II; DM1: diabetes mellitus tipo 1; ECA: enzima conversora de angiotensina; GLP-1: peptídeo semelhante ao glucagon 1; RAAS: sistema renina-angiotensina-aldosterona; SGLT2:, cotransportador de sódio-glicose-2.

#### 10.7.8. Papel dos Análogos de GLP-1 no Tratamento Cardiometabólico em Mulheres

Os análogos de GLP-1 promovem secreção de insulina de forma glicose-dependente, reduzem o apetite e retardam o esvaziamento gástrico. Contribuem para controle glicêmico eficaz e para melhora de marcadores de RCV.^369^

#### 10.7.9. Especificidades em Mulheres

Fatores hormonais femininos e a distribuição de gordura corporal contribuem para a expressão mais agressiva da síndrome cardiometabólica. Estudos indicam que os análogos de GLP-1 exercem efeitos particularmente benéficos, com maior impacto na redução de peso e na melhora de parâmetros inflamatórios e lipídicos quando em comparação com os homens, o que pode ser parcialmente explicado por respostas hormonais diferenciadas e maior sensibilidade aos efeitos anorexígenos desses fármacos.^371 , 372^

#### 10.7.10. Semaglutida

Agonista do receptor de GLP-1, tem eficácia consolidada no controle glicêmico e na redução de peso. Em pacientes com sobrepeso ou obesidade, demonstrou reduções significativas no peso corporal e na HbA1c, mesmo na ausência de DM2.^371^ Em pacientes com DM2, reduziu em 26% o risco de eventos cardiovasculares maiores.^135^ Nos indivíduos com DCV aterosclerótica prévia e IMC ≥ 27, sem histórico de DM2, reduziu em 20% o risco combinado de eventos cardiovasculares maiores e a mortalidade por todas as causas.^274^ Também foram demonstrados efeitos renais significativos em pacientes com DM2 e DRC. Houve redução de 24% no risco de eventos renais graves e morte por causas renais ou cardiovasculares no grupo tratado com semaglutida apresentando menor declínio anual da taxa de filtração glomerular estimada.^373^ Evidências recentes indicam que seu uso por via oral em pacientes com diabetes também contribui para a redução de eventos cardiovasculares, sendo útil em contextos clínicos onde o uso injetável é menos viável.^374^

#### 10.7.11. Tirzepatida

Agonista duplo dos receptores de GIP e GLP-1, representa um avanço no manejo da doença cardiometabólica. Seu uso demonstrou reduções significativas nos níveis de HbA1c (até 2,4%) e no peso corporal (até 20%), além de melhorias no perfil lipídico e redução de marcadores inflamatórios.^375 , 376^ Essas características posicionam este agonista duplo GIP/GLP1 como alternativa promissora, especialmente em mulheres com obesidade central e RI.

#### 10.7.12. Perspectivas Futuras

Um recente estudo avaliando a eficácia do uso semanal de 2,4 mg de semaglutida em relação a 5-15 mg de tirzepatida para tratamento da obesidade demonstrou superioridade da tirzepatida tanto na perda ponderal quanto no controle metabólico, com efeitos cardiovasculares e perfil de segurança comparáveis. Ainda assim, o subgrupo de mulheres tratadas com semaglutida obteve maior perda proporcional de peso em relação aos homens e melhora de FR cardiometabólicos após as 72 semanas de tratamento. De qualquer forma, o planejamento e a decisão terapêutica final deverão ser sempre individuais e feitos com base nas características fisiológicas, hormonais e metabólicas específicas de cada paciente.^376^

### 10.8. Considerações Farmacológicas Específicas

#### 10.8.1. Interações Medicamentosas

Mulheres com condições crônicas como osteoporose, depressão e ansiedade estão frequentemente expostas a polifarmácia, ampliando o risco de interações medicamentosas relevantes, devido a menor depuração renal e maior proporção de gordura corporal. Entre as interações de maior impacto, destacam-se os inibidores seletivos da recaptação da serotonina, que podem inibir o CYP3A4 e interferir no metabolismo de estatinas lipofílicas, elevando o risco de miopatia.^367^

#### 10.8.2. Importância do Controle de Peso

Intervenções que promovam a redução de peso corporal possuem impacto direto na melhora do perfil lipídico, controle pressórico e sensibilidade à insulina. Dentre essas intervenções, destaca-se a classe dos agonistas GLP-1 que têm demonstrado efeitos consistentes na redução ponderal, com benefícios adicionais na prevenção de eventos cardiovasculares maiores. O manejo da obesidade deve ser compreendido como componente fundamental da estratégia terapêutica dos distúrbios cardiometabólicos.^117 , 274 , 373 - 376^

#### 10.8.3. Abordagem Individualizada

A heterogeneidade clínica entre mulheres exige que o manejo das condições cardiometabólicas seja pautado por abordagens personalizadas, que considerem não apenas parâmetros fisiológicos, como idade e presença de comorbidades, mas também fatores psicossociais, histórico reprodutivo e preferências individuais. A individualização terapêutica, portanto, representa um pilar essencial para o cuidado centrado na mulher, promovendo intervenções mais eficazes e seguras ao longo de sua trajetória de saúde.^377 , 378^

### 10.9. Tratamento Cirúrgico

#### 10.9.1. Cirurgia Bariátrica

A cirurgia bariátrica é uma estratégia consagrada em todo o mundo, com mortalidade perioperatória variando entre 0,03% e 0,2%.^379^ No Brasil, cerca de 70% dos pacientes submetidos ao procedimento são mulheres.^380^ Seus benefícios englobam melhora significativa da doença metabólica e de comorbidades associadas, como DM2, apneia obstrutiva do sono, HAS, dislipidemia, síndrome de Pickwick, DHEM e doença do refluxo gastroesofágico.^381^ A indicação segue diretrizes que preconizam cirurgia em pacientes com IMC ≥ 35 kg/m² e comorbidades associadas, ou IMC ≥ 40 kg/m² isolado, independentemente da presença de outras doenças.^382^ A cirurgia bariátrica esteve associada a menor incidência de eventos cardiovasculares maiores em pacientes com DCV e obesidade em uma coorte de 2.638 pacientes seguida por 4,6 anos, tendo seu benefício principal no grupo com IC e DIC.^383^

Após o procedimento, as mulheres apresentam perda ponderal discretamente menor que os homens. A redução de peso pode levar a aumento abrupto da SHBG, queda dos níveis de testosterona e elevação do FSH, melhorando a disfunção ovulatória e a irregularidade menstrual e favorecendo a concepção espontânea em idade fértil.^384^

São contraindicações à cirurgia bariátrica: doença psiquiátrica grave sem controle; demências moderadas a graves; dependência de álcool ou drogas ilícitas; DIC grave ou outras cardiopatias graves; e hipertensão portal com varizes esofágicas. Em pacientes com IMC > 50 kg/m², o risco cirúrgico é elevado devido à maior incidência de comorbidades e à complexidade anatômica, refletindo-se em tempo operatório prolongado, maior morbidade perioperatória e internações mais longas em alguns estudos.^382^

**Figura 10.4:**
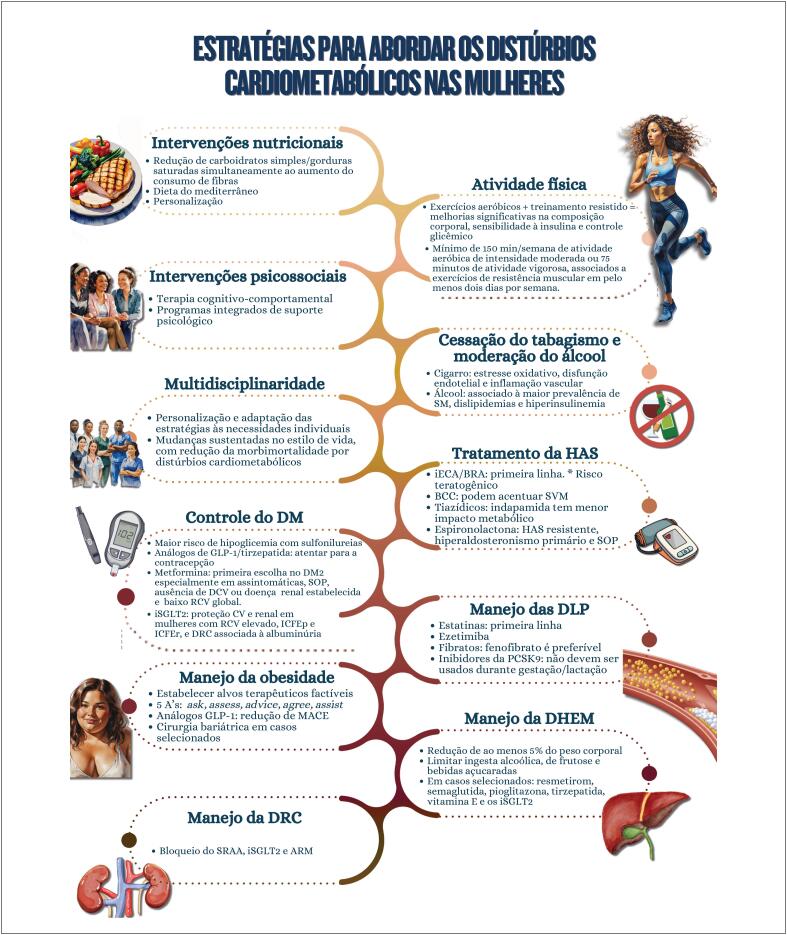
Estratégias para abordar os distúrbios cardiometabólicos nas mulheres. ARM: antagonista do receptor de mineralocorticoide; BCC: bloqueador dos canais de cálcio; BRA: bloqueador de receptor de angiotensina; CV: cardiovascular; DCV: doença cardiovascular; DM: diabetes mellitus; DM2: diabetes mellitus tipo 2; DHEM: doença hepática esteatótica metabólica; DLP: dislipidemia; DRC: doença renal crônica; SM: síndrome metabólica; GLP-1: peptídeo semelhante ao glucagon 1; HAS: hipertensão arterial sistêmica; ICFEp: insuficiência cardíaca com fração de ejeção preservada; ICFEr: insuficiência cardíaca com fração de ejeção reduzida; iECA: inibidor da enzima conversora de angiotensina; iSGLT2: inibidor do cotransportador de sódio-glicose tipo 2; MACE: eventos cardiovasculares maiores; RCV: risco cardiovascular; SOP: síndrome do ovário policístico; SRAA: sistema renina-angiotensina-aldosterona; SVM: sintomas vasomotores.

A Figura 10.4 resume as estratégias de abordagem dos distúrbios cardiometabólicos nas mulheres, ressaltando a importância da abordagem multidisciplinar centrada na mulher, envolvendo a promoção de hábitos saudáveis, rastreamento individualizado de FR e seu controle, manejo clínico integrado e inclusão de aspectos psicossociais na avaliação cardiometabólica.

## 11. Recomendações para o Manejo dos Distúrbios Cardiometabólicos nas Mulheres

Para a elaboração das recomendações listadas no final deste capítulo, procedemos a uma revisão sistemática (Anexo 1) com dez perguntas PICO. Nessa revisão sistemática, foram incluídos revisões sistemáticas, metanálises, estudos multicêntricos controlados e randomizados, além de diretrizes. Utilizamos as seguintes bases de dados: PubMed/MEDLINE, Embase, Cochrane Library, LILACS e BVS. Empregamos a metodologia GRADE ( *Grading of Recommendations Assessment, Development and Evaluation* ), que é um sistema utilizado para avaliar a certeza da evidência e a força das recomendações em saúde nas diretrizes clínicas. O GRADE classifica a evidência em níveis (alta, moderada, baixa ou muito baixa) e, com base nessa classificação, determina a direção (CONTRA ou a FAVOR) e a força de uma recomendação (FORTE ou FRACA), conforme descrito a seguir.

- Alta: Há alta confiança de que o efeito estimado esteja próximo do efeito verdadeiro.
- Moderada: Há moderada confiança no efeito estimado. É provável que estudos futuros possam impactar a confiança na estimativa.
- Baixa: A confiança no efeito estimado é limitada.
- Muito baixa: Há grande incerteza na estimativa do efeito.

**Figura 11.1:**
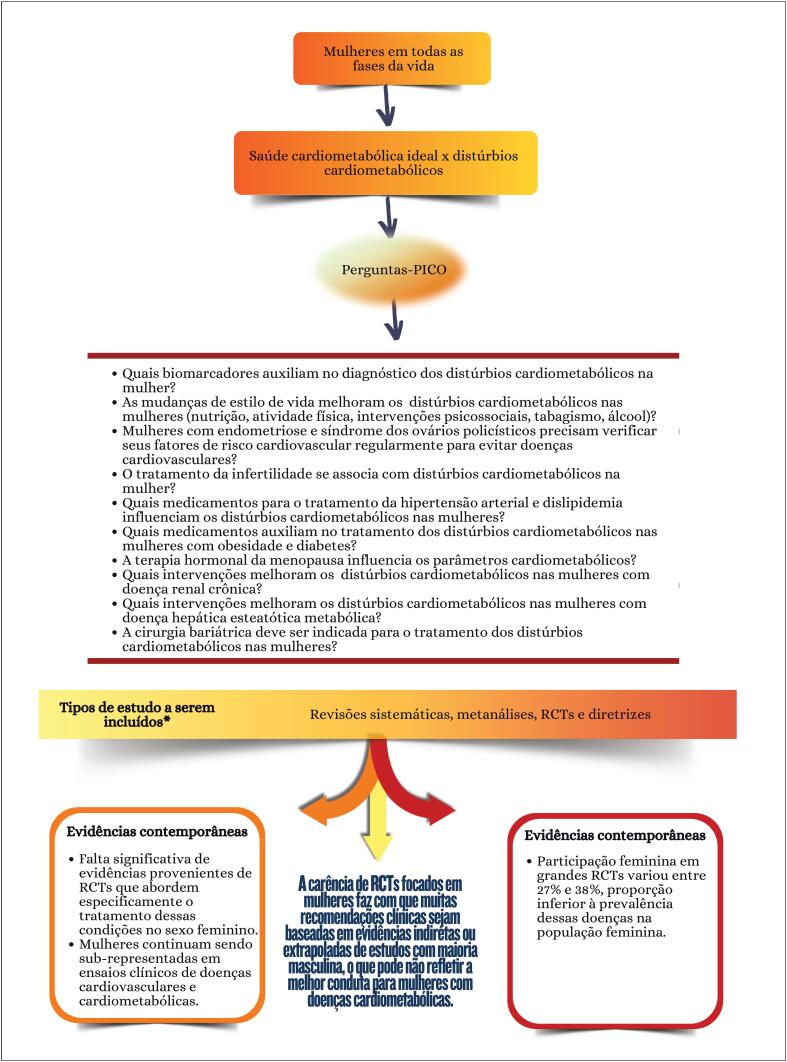
Estrutura da revisão sistemática que norteou este posicionamento. RCTs: estudos multicêntricos controlados e randomizados.

A Figura 11.1 resume a estrutura da revisão sistemática que norteou este posicionamento.

Apesar do crescente reconhecimento da importância dos distúrbios cardiometabólicos em mulheres, há uma falta significativa de evidências provenientes de estudos multicêntricos controlados e randomizados que abordem especificamente o tratamento dessas condições no sexo feminino. Essa limitação compromete a elaboração de recomendações clínicas baseadas em evidências robustas e adaptadas às particularidades das mulheres. Cabe ressaltar que as mulheres continuam sendo sub-representadas em ensaios clínicos de doenças cardiovasculares e cardiometabólicas. Em análises relativamente recentes, a participação feminina em grandes estudos multicêntricos controlados e randomizados variou entre 27% e 38% dos participantes, proporção inferior à prevalência dessas doenças na população feminina.^385 , 386^ Apenas cerca de um terço dos estudos publica análises de resultados específicas para mulheres, dificultando a avaliação da eficácia e segurança dos tratamentos nesse grupo. Adicionalmente, a carência de estudos multicêntricos controlados e randomizados focados em mulheres faz com que muitas recomendações clínicas sejam baseadas em evidências indiretas ou extrapoladas de estudos com maioria masculina, o que pode não refletir a melhor conduta para mulheres com distúrbios cardiometabólicos.^387^

Existe uma forte correlação entre distúrbios cardiometabólicos e condições inflamatórias nas mulheres ao longo de seu ciclo de vida, como SOP, DG ou pré-eclâmpsia, que predispõem ao aumento do risco de IAM, AVC e DIC, além de repercussão sobre o binômio materno-fetal posteriormente, na infância e na juventude do concepto. Estudo recente com 12.480 pares mãe-filho demonstrou que os FR cardiometabólico maternos foram significativamente associados ao aumento de 4,88% e 1,90% nas pressões sistólica e diastólica dos filhos, respectivamente. Uma combinação de distúrbios hipertensivos da gravidez com obesidade pré-gestacional ou DG foi significativamente associada a uma PA mais alta entre 2 e 18 anos de idade, ratificando a importância da abordagem precoce das condições cardiometabólicas na infância, na adolescência e no período reprodutivo das mulheres.^388^

As diferenças sexuais observadas em relação aos distúrbios cardiometabólicos e as doenças inflamatórias sugerem que os hormônios sexuais regulem as vias inflamatórias nas mulheres. Existem alterações na sinalização inflamatória em mulheres que podem sustentar diferenças na hipertensão, aterosclerose, obesidade, DRC e DHEM. O conhecimento dos mecanismos específicos que impulsionam as condições inflamatórias crônicas que afetam as mulheres, como SOP, DG e hipertensão pós-menopausa, auxiliará na abordagem multidisciplinar necessária à diminuição da carga das DCV nas mulheres.^30^

Várias intervenções apresentam efeito combinado para as doenças crônicas, especialmente as cardiometabólicas. Estudo recente testou as associações entre o padrão de atividade física e a incidência de 678 condições em 89.573 participantes (62 ± 8 anos de idade; 56% mulheres) do estudo de coorte prospectivo UK Biobank, que usaram um acelerômetro por uma semana entre junho de 2013 e dezembro de 2015. Foi observado que tanto a atividade física concentrada em 1 ou 2 dias quanto os padrões de atividade mais regulares estavam associados a um risco similarmente menor de mais de 200 doenças, em particular com menor risco de condições **cardiometabólicas** .^389^

Apresentamos a seguir as recomendações onde existem evidências relativamente robustas para os distúrbios cardiometabólicos nas mulheres.

## RECOMENDAÇÕES ATUAIS PARA OS DISTÚRBIOS CARDIOMETABÓLICOS NAS MULHERES

DIETA^390 - 398^

**Table:** 

Recomendações – A FAVOR	Força de Recomendação	Certeza da Evidência
Recomendam-se intervenções abrangentes no estilo de vida para mulheres com sobrepeso, obesidade, síndrome metabólica, hipertensão, dislipidemia, doença hepática esteatótica metabólica e DM2 empregando dietas mediterrânea, DASH, de restrição energética intermitente, com alto teor de proteína e restrição calórica com objetivo de reduzir em média 5–10% do peso corporal.	FORTE	ALTA

DM2: diabetes mellitus tipo 2; DASH: Dietary Approaches to Stop Hypertension.

ATIVIDADE FÍSICA^399 - 408^

**Table:** 

Recomendações – A FAVOR	Força de Recomendação	Certeza da Evidência
Recomenda-se para mulheres com sobrepeso e obesidade, síndrome metabólica, DM2, hipertensão, dislipidemia e no período de perimenopausa a prática de atividade física regular com meta inicial de 150 minutos por semana de exercícios aeróbicos e treinamento de força duas a três vezes por semana. Deve-se aumentar para 300 minutos por semana para perda de peso ≥ 5%, dado que a redução da gordura visceral apresenta uma relação com melhora nos desfechos cardiometabólicos no curto e médio prazo.	FORTE	MODERADA

DM2: diabetes mellitus tipo 2.

CONSUMO DE ÁLCOOL E TABACO^356 , 409 - 417^

**Table:** 

Recomendações – A FAVOR	Força de Recomendação	Certeza da Evidência
O consumo de álcool aumenta o risco de anemia e diabetes gestacional, enquanto o uso de tabaco duplica a chance de baixo peso ao nascer e aumenta as chances de infertilidade e menopausa precoce. O consumo de álcool por mulheres, mesmo em quantidades moderadas, e o tabagismo podem elevar a pressão arterial, aumentar os níveis séricos de LDL-c e triglicerídeos, diminuir o HDL-c sérico e aumentar o risco de AVC, insuficiência cardíaca, resistência à insulina e DM2. Recomenda-se que mulheres não fumem e consumam < 140 g de álcool por semana fora do período de gravidez.	FORTE	ALTA

AVC: acidente vascular cerebral; DM2: diabetes mellitus tipo 2.

OBESIDADE^274 , 418 - 425^

**Table:** 

Recomendações – A FAVOR	Força de Recomendação	Certeza da Evidência
Recomenda-se mudança de estilo de vida associada ao uso dos agonistas do GLP-1 e dos inibidores do SGLT2 para tratamento de obesidade e DM2 em mulheres, com efeitos benéficos na redução do peso e em parâmetros metabólicos.	FORTE	ALTA

DM2: diabetes mellitus tipo 2.

## RECOMENDAÇÕES ATUAIS PARA OS DISTÚRBIOS CARDIOMETABÓLICOS NAS MULHERES

DIABETES^420 , 426 - 432^

**Table:** 

Recomendações – A FAVOR	Força de Recomendação	Certeza da Evidência
Recomenda-se intervenção no estilo de vida associada ao uso de análogos do GLP1 e dos inibidores do SGLT2 para tratamento do DM2 em mulheres, com efeitos benéficos na redução do peso e em parâmetros metabólicos, como redução da hemoglobina glicada. Metformina não deve ser utilizada como agente de primeira linha para o manejo do diabetes na gravidez e, quando utilizada para tratar a síndrome dos ovários policísticos e induzir ovulação, deve ser descontinuada até o final do primeiro trimestre, pois atravessa a barreira placentária.	FORTE	ALTA

DM2: diabetes mellitus tipo 2.

DISLIPIDEMIA^366^

**Table:** 

Recomendações – A FAVOR	Força de Recomendação	Certeza da Evidência
Em mulheres com muito alto risco cardiovascular em prevenção secundária, além das mudanças de estilo de vida, devem-se empregar estatinas de alta potência isoladas ou combinadas com ezetimiba, com o objetivo de reduzir LDL-c ≥ 50% com alvo de LDL-c < 50 mg/dL e NÃO-HDL-c < 80 mg/dL, independentemente do valor basal de LDL-c.	FORTE	ALTA
Em mulheres de alto risco cardiovascular em prevenção secundária, além das mudanças de estilo de vida, devem-se empregar estatinas de alta potência isoladas ou combinadas com ezetimiba, com o objetivo de reduzir a LDL-c ≥ 50% com alvo de LDL-c < 70 mg/dL e NÃO-HDL-c < 100 mg/dL, independentemente do valor basal de LDL-c.	FORTE	ALTA

HIPERTENSÃO ARTERIAL^209 , 211 , 214^

**Table:** 

Recomendações – A FAVOR	Força de Recomendação	Certeza da Evidência
Recomenda-se o controle do peso corporal para alcançar valores saudáveis de IMC (cerca de 20–25 kg/m^2^) e circunferência da cintura (<80 cm em mulheres), visando reduzir a PA e o risco cardiovascular. Recomenda-se a prática de exercícios de baixa a moderada intensidade em todas as gestantes sem contraindicações para reduzir o risco de hipertensão gestacional e pré-eclâmpsia.	FORTE	ALTA
Em mulheres com hipertensão crônica ou gestacional, recomenda-se iniciar o tratamento medicamentoso para aquelas com PA sistólica confirmada ≥ 140 mmHg ou PA diastólica ≥ 90 mmHg.	FORTE	ALTA

SOP: síndrome dos ovários policísticos; DM2: diabetes mellitus tipo 2.

DOENÇA HEPÁTICA ESTEATÓTICA METABÓLICA^309 , 311 , 314 , 302 , 414 , 433 , 434^

**Table:** 

Recomendações – A FAVOR	Força de Recomendação	Certeza da Evidência
Em mulheres com DHEM, recomenda-se a modificação do estilo de vida (perda de peso, mudanças na dieta, exercícios físicos, cessação do uso de tabaco e álcool), manejo otimizado das comorbidades – incluindo o uso de terapias a base de incretinas (ex: semaglutida, tirzepatida) para DM2 ou obesidade (se indicado) – e cirurgia bariátrica na presença de obesidade severa	FORTE	MODERADA

DM2: diabetes mellitus tipo 2; DHEM: doença hepática esteatótica metabólica.

## RECOMENDAÇÕES ATUAIS PARA OS DISTÚRBIOS CARDIOMETABÓLICOS NAS MULHERES

DOENÇA RENAL CRÔNICA^435 - 441^

**Table:** 

Recomendações – A FAVOR	Força de Recomendação	Certeza da Evidência
A gravidez é uma das principais causas de lesão renal aguda em mulheres em idade fértil e junto com pré-eclâmpsia pode levar a DRC subsequente. A DRC tem efeito negativo na gravidez, mesmo em estágios muito iniciais, e os riscos aumentam com a progressão da DRC e com a concomitância de DM2. Recomenda-se monitoramento dos marcadores de função renal durante a gravidez e nos anos subsequentes.	FORTE	MODERADA

DM2: diabetes mellitus tipo 2; DRC: doença renal crônica.

TERAPIA CIRÚRGICA* - CIRURGIA BARIÁTRICA^442 - 448^

* ( *bypass* gástrico em Y de Roux, gastrectomia vertical, banda gástrica ajustável laparoscópica, revestimento de bypass duodenal-jejunal/desvio biliopancreático)

**Table:** 

Recomendações – A FAVOR	Força de Recomendação	Certeza da Evidência
A cirurgia bariátrica é recomendada para mulheres com IMC ≥ 35 kg/m² e histórico de diabetes, DHEM ou alto risco de eventos cardiovasculares, assim como para aquelas com IMC ≥ 40 kg/m², independentemente de comorbidades, para a melhoria dos parâmetros cardiometabólicos (perfil lipídico e glicêmico) e marcadores inflamatórios.	FORTE	ALTA

DHEM: doença hepática esteatótica metabólica; IMC: índice de massa corpórea.

SÍNDROME DOS OVÁRIOS POLICÍSTICOS^43 , 65 , 97 , 141 , 156 , 159 , 172 , 265 , 304 , 444^

**Table:** 

Recomendações – A FAVOR	Força de Recomendação	Certeza da Evidência
A SOP está associada a anovulação, hiperandrogenismo e resistência à insulina, aumentando o risco de doenças cardiovasculares e DM2. Mulheres com SOP devem realizar uma avaliação do perfil lipídico completo e do estado glicêmico no momento do diagnóstico. O tratamento inclui, além de mudanças no estilo de vida, agentes sensibilizadores de insulina, como metformina ou mioinositol, e, para casos de hirsutismo e ciclos irregulares, contraceptivos orais combinados. Medicamentos antiobesidade, como liraglutida, semaglutida e agonistas do GLP-1 e orlistate, também podem ser indicados no controle do peso.	FORTE	MODERADA

SOP: síndrome dos ovários policísticos; DM2: diabetes mellitus tipo 2.

ENDOMETRIOSE^203 - 206^

**Table:** 

Recomendações – A FAVOR	Força de Recomendação	Certeza da Evidência
A endometriose está associada a um processo inflamatório crônico com aumento do estresse oxidativo e elevação de fatores de risco cardiovascular, maior risco de hipertensão, alterações lipídicas, doença arterial coronária, insuficiência cardíaca e AVC. Tratamento com terapias hormonais e estimulação ovariana para fertilização *in vitro* também podem elevar o risco de tromboembolismo. Intervenções baseadas no estilo de vida podem auxiliar na redução do risco cardiovascular.	FRACA	FRACA

AVC: acidente vascular cerebral.

## SUPLEMENTO

**1:** Quais biomarcadores auxiliam no diagnóstico das anormalidades cardiometabólicas na mulher?

Base	Estratégia
MEDLINE/PubMed	("Biomarkers"[Mesh] OR "biological markers"[tiab] OR biomarkers[tiab] OR "molecular markers"[tiab] OR "diagnostic markers"[tiab] OR "metabolic markers"[tiab] OR "cardiometabolic biomarkers"[tiab]) AND ("Metabolic Syndrome"[Mesh] OR "Cardiovascular Diseases"[Mesh] OR "Glucose Intolerance"[Mesh] OR "Hypertension"[Mesh] OR "Dyslipidemias"[Mesh] OR "Insulin Resistance"[Mesh] OR "Type 2 Diabetes Mellitus"[Mesh] OR "cardiometabolic risk"[tiab] OR "cardiometabolic abnormalities"[tiab] OR "metabolic syndrome"[tiab] OR "insulin resistance"[tiab] OR "glucose intolerance"[tiab] OR dyslipidemia*[tiab] OR hypertension[tiab] OR "type 2 diabetes"[tiab] OR "cardiovascular disease"[tiab]) AND ("Women"[Mesh] OR "Female"[Mesh] OR women[tiab] OR woman[tiab] OR female*[tiab]) Filters applied: in the last 5 years, Randomized Controlled Trial, Systematic Review.
Embase	('biological marker'/exp OR 'biomarker'/exp OR 'biological marker':ti, ab OR biomarkers:ti, ab OR 'molecular marker':ti, ab OR 'diagnostic marker':ti, ab OR 'metabolic marker':ti, ab OR 'cardiometabolic biomarker':ti, ab) AND ('cardiometabolic disorder'/exp OR 'metabolic syndrome'/exp OR 'cardiovascular disease'/exp OR 'glucose intolerance'/exp OR 'hypertension'/exp OR 'dyslipidemia'/exp OR 'insulin resistance'/exp OR 'type 2 diabetes mellitus'/exp OR 'cardiometabolic risk':ti, ab OR 'cardiometabolic abnormalit*':ti, ab OR 'metabolic syndrome':ti, ab OR 'insulin resistance':ti, ab OR 'glucose intolerance':ti, ab OR dyslipidemi*:ti, ab OR hypertension:ti, ab OR 'type 2 diabetes':ti, ab OR 'cardiovascular disease':ti, ab) AND ('female'/exp OR women:ti, ab OR woman:ti, ab OR female*:ti, ab)
Cochrane	([mh "Biomarkers"] OR "biological markers":ti, ab OR biomarkers:ti, ab OR "molecular markers":ti, ab OR "diagnostic markers":ti, ab OR "metabolic markers":ti, ab OR "cardiometabolic biomarkers":ti, ab) AND ([mh "Metabolic Syndrome"] OR [mh "Cardiovascular Diseases"] OR [mh "Glucose Intolerance"] OR [mh "Hypertension"] OR [mh "Dyslipidemias"] OR [mh "Insulin Resistance"] OR [mh "Type 2 Diabetes Mellitus"] OR "cardiometabolic risk":ti, ab OR "cardiometabolic abnormalities":ti, ab OR "metabolic syndrome":ti, ab OR "insulin resistance":ti, ab OR "glucose intolerance":ti, ab OR dyslipidemia*:ti, ab OR hypertension:ti, ab OR "type 2 diabetes":ti, ab OR "cardiovascular disease":ti, ab) AND ([mh "Women"] OR [mh "Female"] OR women:ti, ab OR woman:ti, ab OR female*:ti, ab)
BVS	(TW: "Biomarcadores" OR "Biomarkers" OR "Biomarcadores" OR "Marcadores Biológicos" OR "Biological Markers" OR "Marcadores Biológicos" OR "Marcadores moleculares" OR "Molecular Markers" OR "Marcadores diagnósticos" OR "Diagnostic Markers" OR "Marcadores metabólicos" OR "Metabolic Markers" OR "Marcadores cardiometabólicos" OR "Cardiometabolic Biomarkers") AND (TW: "Anormalidades Cardiometabólicas" OR "Cardiometabolic Abnormalities" OR "Anormalidades Cardiometabólicas" OR "Riesgo Cardiometabólico" OR "Cardiometabolic Risk" OR "Risco Cardiometabólico" OR "Síndrome Metabólica" OR "Metabolic Syndrome" OR "Síndrome Metabólica" OR "Resistência à Insulina" OR "Insulin Resistance" OR "Resistencia a la Insulina" OR "Intolerância à Glicose" OR "Glucose Intolerance" OR "Intolerancia a la Glucosa" OR "Dislipidemia" OR "Dyslipidemia" OR "Dislipidemia" OR "Hipertensão" OR "Hypertension" OR "Hipertensión" OR "Diabetes tipo 2" OR "Type 2 Diabetes" OR "Diabetes tipo 2" OR "Doenças Cardiovasculares" OR "Cardiovascular Diseases" OR "Enfermedades Cardiovasculares") AND (TW: "Mulheres" OR "Women" OR "Mujeres" OR "Feminino" OR "Female" OR "Femenino")

## SUPLEMENTO

**2:** A Terapia Hormonal da Menopausa influencia os parâmetros cardiometabólicos?

Base	Estratégia
**MEDLINE/PubMed**	("Hormone Replacement Therapy"[Mesh] OR "Estrogen Replacement Therapy"[Mesh] OR "menopausal hormone therapy"[tiab] OR "hormone replacement therapy"[tiab] OR HRT[tiab] OR "estrogen therapy"[tiab] OR "estradiol therapy"[tiab] OR "postmenopausal hormone therapy"[tiab]) AND ("Metabolic Syndrome"[Mesh] OR "Cardiovascular Diseases"[Mesh] OR "Blood Pressure"[Mesh] OR "Insulin Resistance"[Mesh] OR "Lipid Metabolism"[Mesh] OR "Cholesterol"[Mesh] OR "Triglycerides"[Mesh] OR "Glucose Metabolism Disorders"[Mesh] OR "cardiometabolic parameters"[tiab] OR "cardiometabolic profile"[tiab] OR "insulin sensitivity"[tiab] OR "lipid profile"[tiab] OR "blood pressure"[tiab] OR "glucose levels"[tiab] OR "cholesterol levels"[tiab] OR triglycerides[tiab]) Filters applied: in the last 5 years, Randomized Controlled Trial, Systematic Review.
Embase	('hormone replacement therapy'/exp OR 'estrogen replacement therapy'/exp OR 'menopausal hormone therapy':ti, ab OR 'hormone replacement therapy':ti, ab OR HRT:ti, ab OR 'estrogen therapy':ti, ab OR 'estradiol therapy':ti, ab OR 'postmenopausal hormone therapy':ti, ab) AND ('metabolic syndrome'/exp OR 'cardiovascular disease'/exp OR 'blood pressure'/exp OR 'insulin resistance'/exp OR 'lipid metabolism'/exp OR 'cholesterol'/exp OR 'triglyceride'/exp OR 'glucose metabolism disorder'/exp OR 'cardiometabolic parameter*':ti, ab OR 'cardiometabolic profile':ti, ab OR 'insulin sensitivity':ti, ab OR 'lipid profile':ti, ab OR 'blood pressure':ti, ab OR 'glucose level*':ti, ab OR 'cholesterol level*':ti, ab OR triglyceride*:ti, ab)
Cochrane	([mh "Hormone Replacement Therapy"] OR [mh "Estrogen Replacement Therapy"] OR "menopausal hormone therapy":ti, ab OR "hormone replacement therapy":ti, ab OR HRT:ti, ab OR "estrogen therapy":ti, ab OR "estradiol therapy":ti, ab OR "postmenopausal hormone therapy":ti, ab) AND ([mh "Metabolic Syndrome"] OR [mh "Cardiovascular Diseases"] OR [mh "Blood Pressure"] OR [mh "Insulin Resistance"] OR [mh "Lipid Metabolism"] OR [mh "Cholesterol"] OR [mh "Triglycerides"] OR [mh "Glucose Metabolism Disorders"] OR "cardiometabolic parameters":ti, ab OR "cardiometabolic profile":ti, ab OR "insulin sensitivity":ti, ab OR "lipid profile":ti, ab OR "blood pressure":ti, ab OR "glucose levels":ti, ab OR "cholesterol levels":ti, ab OR triglycerides:ti, ab)
BVS	("Terapia Hormonal da Menopausa" OR "Menopausal Hormone Therapy" OR "Terapia Hormonal de la Menopausia" OR "Terapia de Reposição Hormonal" OR "Hormone Replacement Therapy" OR "Terapia de Reemplazo Hormonal" OR "Terapia Estrogênica" OR "Estrogen Therapy" OR "Terapia con Estrógenos" OR "Terapia com Estradiol" OR "Estradiol Therapy" OR "Terapia con Estradiol" OR HRT) AND ("Parâmetros Cardiometabólicos" OR "Cardiometabolic Parameters" OR "Parámetros Cardiometabólicos" OR "Perfil Cardiometabólico" OR "Cardiometabolic Profile" OR "Perfil Cardiometabólico" OR "Síndrome Metabólica" OR "Metabolic Syndrome" OR "Síndrome Metabólica" OR "Sensibilidade à Insulina" OR "Insulin Sensitivity" OR "Sensibilidad a la Insulina" OR "Perfil Lipídico" OR "Lipid Profile" OR "Perfil Lipídico" OR "Pressão Arterial" OR "Blood Pressure" OR "Presión Arterial" OR "Níveis de Glicose" OR "Glucose Levels" OR "Niveles de Glucosa" OR "Colesterol" OR "Cholesterol" OR "Colesterol" OR "Triglicerídeos" OR "Triglycerides" OR "Triglicéridos" OR "Doenças Cardiovasculares" OR "Cardiovascular Diseases" OR "Enfermedades Cardiovasculares")

## SUPLEMENTO

**3:** As mudanças de estilo de vida melhoram as anormalidades cardiometabólicas nas mulheres?

Base	Estratégia
MEDLINE/PubMed	("Life Style"[Mesh] OR "Lifestyle"[tiab] OR "lifestyle change"[tiab] OR "behavior change"[tiab] OR "lifestyle intervention"[tiab] OR "behavioral intervention"[tiab]) AND ("Metabolic Syndrome"[Mesh] OR "Cardiovascular Diseases"[Mesh] OR "Insulin Resistance"[Mesh] OR "Glucose Intolerance"[Mesh] OR "Dyslipidemias"[Mesh] OR "Obesity"[Mesh] OR "Hypertension"[Mesh] OR "Type 2 Diabetes Mellitus"[Mesh] OR "Physical Activity"[Mesh] OR "Exercise"[Mesh] OR "Motor Activity"[Mesh] OR "Diet"[Mesh] OR "Nutrition Therapy"[Mesh] OR "Healthy Diet"[tiab] OR "Physical Activity"[tiab] OR exercise[tiab] OR "psychosocial intervention"[tiab] OR "smoking cessation"[tiab] OR smoking[tiab] OR tobacco[tiab] OR alcohol[tiab] OR "alcohol consumption"[tiab] OR "cardiometabolic abnormalities"[tiab] OR "cardiometabolic risk"[tiab]) AND ("Women"[Mesh] OR "Female"[Mesh] OR women[tiab] OR woman[tiab] OR female*[tiab]) Filters applied: in the last 5 years, Randomized Controlled Trial, Systematic Review.
Embase	('life style'/exp OR 'lifestyle change':ti, ab OR 'behavior change':ti, ab OR 'lifestyle intervention':ti, ab OR 'behavioral intervention':ti, ab) AND ('metabolic syndrome'/exp OR 'cardiovascular disease'/exp OR 'insulin resistance'/exp OR 'glucose intolerance'/exp OR 'dyslipidemia'/exp OR 'obesity'/exp OR 'hypertension'/exp OR 'type 2 diabetes mellitus'/exp OR 'physical activity'/exp OR 'exercise'/exp OR 'diet'/exp OR 'nutrition therapy'/exp OR 'healthy diet':ti, ab OR 'psychosocial intervention':ti, ab OR 'smoking cessation':ti, ab OR smoking:ti, ab OR tobacco:ti, ab OR alcohol:ti, ab OR 'alcohol consumption':ti, ab OR 'cardiometabolic risk':ti, ab OR 'cardiometabolic abnormalit*':ti, ab) AND ('female'/exp OR women:ti, ab OR woman:ti, ab OR female*:ti, ab)
Cochrane	("lifestyle" OR "lifestyle change" OR "lifestyle intervention" OR "behavior change" OR "behavioral intervention") AND ("cardiometabolic abnormalities" OR "cardiometabolic risk" OR "metabolic syndrome" OR "insulin resistance" OR "glucose intolerance" OR "dyslipidemia" OR "obesity" OR "hypertension" OR "type 2 diabetes" OR "cardiovascular disease" OR "physical activity" OR "exercise" OR "diet" OR "nutrition" OR "healthy diet" OR "psychosocial intervention" OR "smoking" OR "smoking cessation" OR "tobacco" OR "alcohol" OR "alcohol consumption") AND (women OR woman OR female OR females)
BVS	(TW: "Estilo de Vida" OR "Lifestyle" OR "Estilo de Vida" OR "Mudança de Estilo de Vida" OR "Lifestyle Change" OR "Cambio de Estilo de Vida" OR "Intervenção de Estilo de Vida" OR "Lifestyle Intervention" OR "Intervención de Estilo de Vida" OR "Mudança Comportamental" OR "Behavior Change" OR "Cambio de Comportamiento" OR "Intervenção Comportamental" OR "Behavioral Intervention" OR "Intervención Conductual") AND (TW: "Anormalidades Cardiometabólicas" OR "Cardiometabolic Abnormalities" OR "Anormalidades Cardiometabólicas" OR "Risco Cardiometabólico" OR "Cardiometabolic Risk" OR "Riesgo Cardiometabólico" OR "Síndrome Metabólica" OR "Metabolic Syndrome" OR "Síndrome Metabólica" OR "Resistência à Insulina" OR "Insulin Resistance" OR "Resistencia a la Insulina" OR "Intolerância à Glicose" OR "Glucose Intolerance" OR "Intolerancia a la Glucosa" OR "Dislipidemia" OR "Dyslipidemia" OR "Dislipidemia" OR "Obesidade" OR "Obesity" OR "Obesidad" OR "Hipertensão" OR "Hypertension" OR "Hipertensión" OR "Diabetes Tipo 2" OR "Type 2 Diabetes" OR "Diabetes Tipo 2" OR "Doenças Cardiovasculares" OR "Cardiovascular Diseases" OR "Enfermedades Cardiovasculares" OR "Atividade Física" OR "Physical Activity" OR "Actividad Física" OR "Exercício" OR "Exercise" OR "Ejercicio" OR "Dieta" OR "Diet" OR "Dieta" OR "Nutrição" OR "Nutrition" OR "Nutrición" OR "Dieta Saudável" OR "Healthy Diet" OR "Dieta Saludable" OR "Intervenção Psicossocial" OR "Psychosocial Intervention" OR "Intervención Psicosocial" OR "Tabagismo" OR "Smoking" OR "Tabaquismo" OR "Cessação do Tabagismo" OR "Smoking Cessation" OR "Cese del Tabaquismo" OR "Álcool" OR "Alcohol" OR "Alcohol") AND (TW: "Mulheres" OR "Women" OR "Mujeres" OR "Feminino" OR "Female" OR "Femenino")

## SUPLEMENTO

**4:** Quais medicamentos auxiliam no tratamento das anormalidades cardiometabólicas nas mulheres com obesidade e diabetes?

Base	Estratégia
MEDLINE/PubMed	("Drug Therapy"[Mesh] OR "Pharmaceutical Preparations"[Mesh] OR "Hypoglycemic Agents"[Mesh] OR "Antihyperglycemic Agents"[Mesh] OR "Antihypertensive Agents"[Mesh] OR "Lipid Regulating Agents"[Mesh] OR medication*[tiab] OR drug*[tiab] OR pharmacotherapy[tiab] OR "glucose-lowering agents"[tiab] OR "antidiabetic drugs"[tiab] OR "insulin sensitizers"[tiab] OR "weight loss drugs"[tiab]) AND (("Metabolic Syndrome"[Mesh] OR "Cardiovascular Diseases"[Mesh] OR "Insulin Resistance"[Mesh] OR "Glucose Intolerance"[Mesh] OR "Hypertension"[Mesh] OR "Dyslipidemias"[Mesh] OR "cardiometabolic risk"[tiab] OR "cardiometabolic abnormalities"[tiab] OR "insulin resistance"[tiab] OR "metabolic syndrome"[tiab] OR "blood pressure"[tiab] OR "glucose intolerance"[tiab] OR cholesterol[tiab] OR "lipid profile"[tiab]) AND ("Women"[Mesh] OR "Female"[Mesh] OR women[tiab] OR woman[tiab] OR female*[tiab])) AND ("Obesity"[Mesh] OR "Obesity, Morbid"[Mesh] OR "Diabetes Mellitus, Type 2"[Mesh] OR obesity[tiab] OR overweight[tiab] OR "morbid obesity"[tiab] OR "type 2 diabetes"[tiab] OR T2DM[tiab]) Filters applied: in the last 5 years, Randomized Controlled Trial, Systematic Review.
Embase	('drug therapy'/exp OR 'pharmaceutical preparation'/exp OR 'hypoglycemic agent'/exp OR 'antihyperglycemic agent'/exp OR 'antihypertensive agent'/exp OR 'lipid regulating agent'/exp OR medication*:ti, ab OR drug*:ti, ab OR pharmacotherapy:ti, ab OR 'glucose lowering agent':ti, ab OR 'antidiabetic drug':ti, ab OR 'insulin sensitizer':ti, ab OR 'weight loss drug':ti, ab) AND (('metabolic syndrome'/exp OR 'cardiovascular disease'/exp OR 'insulin resistance'/exp OR 'glucose intolerance'/exp OR 'hypertension'/exp OR 'dyslipidemia'/exp OR 'cardiometabolic risk':ti, ab OR 'cardiometabolic abnormalit*':ti, ab OR 'blood pressure':ti, ab OR cholesterol:ti, ab OR 'lipid profile':ti, ab) AND ('female'/exp OR women:ti, ab OR woman:ti, ab OR female*:ti, ab)) AND ('obesity'/exp OR 'morbid obesity'/exp OR 'type 2 diabetes mellitus'/exp OR obesity:ti, ab OR overweight:ti, ab OR 'morbid obesity':ti, ab OR 'type 2 diabetes':ti, ab OR T2DM:ti, ab)
Cochrane	([mh "Drug Therapy"] OR [mh "Pharmaceutical Preparations"] OR [mh "Hypoglycemic Agents"] OR [mh "Antihyperglycemic Agents"] OR [mh "Antihypertensive Agents"] OR [mh "Lipid Regulating Agents"] OR medication*:ti, ab OR drug*:ti, ab OR pharmacotherapy:ti, ab OR "glucose-lowering agents":ti, ab OR "antidiabetic drugs":ti, ab OR "insulin sensitizers":ti, ab OR "weight loss drugs":ti, ab) AND (([mh "Metabolic Syndrome"] OR [mh "Cardiovascular Diseases"] OR [mh "Insulin Resistance"] OR [mh "Glucose Intolerance"] OR [mh "Hypertension"] OR [mh "Dyslipidemias"] OR "cardiometabolic risk":ti, ab OR "cardiometabolic abnormalities":ti, ab OR "insulin resistance":ti, ab OR "metabolic syndrome":ti, ab OR "blood pressure":ti, ab OR "glucose intolerance":ti, ab OR cholesterol:ti, ab OR "lipid profile":ti, ab) AND ([mh "Women"] OR [mh "Female"] OR women:ti, ab OR woman:ti, ab OR female*:ti, ab)) AND ([mh "Obesity"] OR [mh "Obesity, Morbid"] OR [mh "Diabetes Mellitus, Type 2"] OR obesity:ti, ab OR overweight:ti, ab OR "morbid obesity":ti, ab OR "type 2 diabetes":ti, ab OR T2DM:ti, ab)
BVS	(TW: "Medicamentos" OR "Drugs" OR "Medicamentos" OR "Terapia Medicamentosa" OR "Drug Therapy" OR "Tratamiento Farmacológico" OR "Preparações Farmacêuticas" OR "Pharmaceutical Preparations" OR "Preparaciones Farmacéuticas" OR "Agentes Hipoglicemiantes" OR "Hypoglycemic Agents" OR "Agentes Hipoglucemiantes" OR "Agentes Antihipertensivos" OR "Antihypertensive Agents" OR "Agentes Antihipertensivos" OR "Agentes Reguladores de Lipídios" OR "Lipid Regulating Agents" OR "Agentes Reguladores de Lípidos" OR "Sensibilizadores de Insulina" OR "Insulin Sensitizers" OR "Sensibilizadores de Insulina" OR "Medicamentos para Emagrecimento" OR "Weight Loss Drugs" OR "Medicamentos para Adelgazar") AND (TW: "Anormalidades Cardiometabólicas" OR "Cardiometabolic Abnormalities" OR "Anormalidades Cardiometabólicas" OR "Risco Cardiometabólico" OR "Cardiometabolic Risk" OR "Riesgo Cardiometabólico" OR "Síndrome Metabólica" OR "Metabolic Syndrome" OR "Síndrome Metabólica" OR "Resistência à Insulina" OR "Insulin Resistance" OR "Resistencia a la Insulina" OR "Dislipidemia" OR "Dyslipidemia" OR "Dislipidemia" OR "Hipertensão" OR "Hypertension" OR "Hipertensión" OR "Diabetes Tipo 2" OR "Type 2 Diabetes" OR "Diabetes Tipo 2" OR "Obesidade" OR "Obesity" OR "Obesidad" OR "Sobrepeso" OR "Overweight" OR "Sobrepeso" OR "Doenças Cardiovasculares" OR "Cardiovascular Diseases" OR "Enfermedades Cardiovasculares") AND (TW: "Mulheres" OR "Women" OR "Mujeres" OR "Feminino" OR "Female" OR "Femenino")

## SUPLEMENTO

**5:** O tratamento da infertilidade se associa com anormalidades cardiometabólicas na mulher?

Base	Estratégia
MEDLINE/PubMed	("Infertility, Female"[Mesh] OR "Infertility"[Mesh] OR infertility[tiab] OR "fertility treatment*"[tiab] OR "Assisted Reproductive Techniques"[Mesh] OR "assisted reproductive technolog*"[tiab] OR ART[tiab] OR "In Vitro Fertilization"[Mesh] OR "in vitro fertilization"[tiab] OR IVF[tiab] OR "Intracytoplasmic Sperm Injection"[Mesh] OR ICSI[tiab] OR "Ovulation Induction"[Mesh] OR "ovulation induction"[tiab] OR clomiphene[tiab] OR letrozole[tiab] OR gonadotropin*[tiab]) AND ("Cardiometabolic Diseases"[Mesh] OR "Metabolic Syndrome"[Mesh] OR "Insulin Resistance"[Mesh] OR "Dyslipidemias"[Mesh] OR "Hypertension"[Mesh] OR "Obesity"[Mesh] OR "Diabetes Mellitus, Type 2"[Mesh] OR cardiometabolic[tiab] OR "cardio-metabolic"[tiab] OR "metabolic syndrome"[tiab] OR "insulin resistance"[tiab] OR dyslipidemia[tiab] OR hyperlipidemia[tiab] OR hypertension[tiab] OR "blood pressure"[tiab] OR obesity[tiab] OR overweight[tiab] OR BMI[tiab] OR "body mass index"[tiab] OR "type 2 diabetes"[tiab] OR diabetes[tiab]) AND ("Women"[Mesh] OR "Female"[Mesh] OR women[tiab] OR woman[tiab] OR female*[tiab]) Filters applied: in the last 5 years, Randomized Controlled Trial, Systematic Review.
Embase	('female infertility treatment'/exp OR 'infertility'/exp OR 'assisted reproductive technology'/exp OR 'in vitro fertilization'/exp OR 'intracytoplasmic sperm injection'/exp OR 'ovulation induction'/exp OR infertility:ti, ab OR 'fertility treatment*':ti, ab OR 'assisted reproductive technolog*':ti, ab OR ART:ti, ab OR 'in vitro fertilization':ti, ab OR IVF:ti, ab OR 'intracytoplasmic sperm injection':ti, ab OR ICSI:ti, ab OR 'ovulation induction':ti, ab OR clomiphene:ti, ab OR letrozole:ti, ab OR gonadotropin*:ti, ab) AND ('cardiometabolic disorder'/exp OR 'metabolic syndrome'/exp OR 'insulin resistance'/exp OR 'dyslipidemia'/exp OR 'hypertension'/exp OR 'obesity'/exp OR 'type 2 diabetes mellitus'/exp OR cardiometabolic:ti, ab OR 'cardio metabolic':ti, ab OR dyslipidemia*:ti, ab OR hyperlipidemia:ti, ab OR hypertension:ti, ab OR 'blood pressure':ti, ab OR BMI:ti, ab OR obesity:ti, ab OR overweight:ti, ab OR diabetes:ti, ab OR 'type 2 diabetes':ti, ab) AND ('female'/exp OR women:ti, ab OR woman:ti, ab OR female*:ti, ab)
Cochrane	([mh "Infertility, Female"] OR [mh "Infertility"] OR infertility:ti, ab OR "fertility treatment*":ti, ab OR [mh "Assisted Reproductive Techniques"] OR "assisted reproductive technolog*":ti, ab OR ART:ti, ab OR [mh "In Vitro Fertilization"] OR "in vitro fertilization":ti, ab OR IVF:ti, ab OR [mh "Intracytoplasmic Sperm Injection"] OR ICSI:ti, ab OR [mh "Ovulation Induction"] OR "ovulation induction":ti, ab OR clomiphene:ti, ab OR letrozole:ti, ab OR gonadotropin*:ti, ab) AND ([mh "Cardiometabolic Diseases"] OR [mh "Metabolic Syndrome"] OR [mh "Insulin Resistance"] OR [mh "Dyslipidemias"] OR [mh "Hypertension"] OR [mh "Obesity"] OR [mh "Diabetes Mellitus, Type 2"] OR cardiometabolic:ti, ab OR "cardio-metabolic":ti, ab OR "metabolic syndrome":ti, ab OR "insulin resistance":ti, ab OR dyslipidemia:ti, ab OR hyperlipidemia:ti, ab OR hypertension:ti, ab OR "blood pressure":ti, ab OR obesity:ti, ab OR overweight:ti, ab OR BMI:ti, ab OR "body mass index":ti, ab OR "type 2 diabetes":ti, ab OR diabetes:ti, ab) AND ([mh "Women"] OR [mh "Female"] OR women:ti, ab OR woman:ti, ab OR female*:ti, ab)
BVS	("Infertilidade" OR "Infertility" OR "Infertilidad" OR "Tratamento da Infertilidade" OR "Infertility Treatment" OR "Tratamiento de la Infertilidad" OR "Tecnologias de Reprodução Assistida" OR "Assisted Reproductive Technologies" OR "Tecnologías de Reproducción Asistida" OR "Fertilização in vitro" OR "In Vitro Fertilization" OR "Fertilización in vitro" OR "Injeção Intracitoplasmática de Espermatozoides" OR "Intracytoplasmic Sperm Injection" OR "Inyección intracitoplasmática de espermatozoides" OR "Indução da Ovulação" OR "Ovulation Induction" OR "Inducción de la Ovulación" OR Clomifeno OR Clomiphene OR Clomifeno OR Letrozol OR Letrozole OR Letrozol OR Gonadotrofinas OR Gonadotropins OR Gonadotropinas) AND ("Anormalidades Cardiometabólicas" OR "Cardiometabolic Abnormalities" OR "Anormalidades Cardiometabólicas" OR "Risco Cardiometabólico" OR "Cardiometabolic Risk" OR "Riesgo Cardiometabólico" OR "Síndrome Metabólica" OR "Metabolic Syndrome" OR "Síndrome Metabólica" OR "Resistência à Insulina" OR "Insulin Resistance" OR "Resistencia a la Insulina" OR "Dislipidemia" OR "Dyslipidemia" OR "Dislipidemia" OR "Hipertensão" OR "Hypertension" OR "Hipertensión" OR "Obesidade" OR "Obesity" OR "Obesidad" OR "Sobrepeso" OR "Overweight" OR "Sobrepeso" OR "Diabetes Tipo 2" OR "Type 2 Diabetes" OR "Diabetes Tipo 2" OR "Doenças Cardiovasculares" OR "Cardiovascular Diseases" OR "Enfermedades Cardiovasculares") AND ("Mulheres" OR "Women" OR "Mujeres" OR "Feminino" OR "Female" OR "Femenino")

## SUPLEMENTO

**6:** Quais os medicamentos para o tratamento da hipertensão arterial e da dislipidemia influenciam as alterações cardiometabólicas nas mulheres?

Base	Estratégia
MEDLINE/PubMed	("Drug Therapy"[Mesh] OR "Drugs"[Mesh] OR "Antihypertensive Agents"[Mesh] OR "Hypolipidemic Agents"[Mesh] OR medication*[tiab] OR drug*[tiab] OR "pharmacologic treatment"[tiab] OR pharmacotherapy[tiab]) AND ("Hypertension"[Mesh] OR "Antihypertensive Agents"[Mesh] OR hypertension[tiab] OR "high blood pressure"[tiab] OR "blood pressure control"[tiab] OR "Dyslipidemias"[Mesh] OR "Hyperlipidemias"[Mesh] OR dyslipidemia[tiab] OR hyperlipidemia[tiab] OR "lipid-lowering"[tiab] OR statins[tiab] OR "ACE inhibitors"[tiab] OR ARBs[tiab] OR "beta blockers"[tiab] OR diuretics[tiab] OR "calcium channel blockers"[tiab]) AND ("Cardiometabolic Diseases"[Mesh] OR "Metabolic Syndrome"[Mesh] OR "Insulin Resistance"[Mesh] OR "Diabetes Mellitus, Type 2"[Mesh] OR "Obesity"[Mesh] OR cardiometabolic[tiab] OR "metabolic syndrome"[tiab] OR "insulin resistance"[tiab] OR "glucose intolerance"[tiab] OR hyperglycemia[tiab] OR "type 2 diabetes"[tiab] OR obesity[tiab] OR overweight[tiab] OR "cardiovascular risk"[tiab]) AND ("Women"[Mesh] OR "Female"[Mesh] OR women[tiab] OR woman[tiab] OR female*[tiab]) Filters applied: in the last 5 years, Randomized Controlled Trial, Systematic Review.
Embase	('drug therapy'/exp OR 'drug'/exp OR 'antihypertensive agent'/exp OR 'hypolipidemic agent'/exp OR medication*:ti, ab OR drug*:ti, ab OR 'pharmacologic treatment':ti, ab OR pharmacotherapy:ti, ab) AND ('hypertension'/exp OR 'antihypertensive agent'/exp OR hypertension:ti, ab OR 'high blood pressure':ti, ab OR 'blood pressure control':ti, ab OR 'dyslipidemia'/exp OR 'hyperlipidemia'/exp OR dyslipidemia:ti, ab OR hyperlipidemia:ti, ab OR 'lipid lowering':ti, ab OR statins:ti, ab OR 'ACE inhibitors':ti, ab OR ARBs:ti, ab OR 'beta blockers':ti, ab OR diuretics:ti, ab OR 'calcium channel blockers':ti, ab) AND ('cardiometabolic disorder'/exp OR 'metabolic syndrome'/exp OR 'insulin resistance'/exp OR 'type 2 diabetes mellitus'/exp OR 'obesity'/exp OR cardiometabolic:ti, ab OR 'metabolic syndrome':ti, ab OR 'insulin resistance':ti, ab OR 'glucose intolerance':ti, ab OR hyperglycemia:ti, ab OR 'type 2 diabetes':ti, ab OR obesity:ti, ab OR overweight:ti, ab OR 'cardiovascular risk':ti, ab) AND ('female'/exp OR women:ti, ab OR woman:ti, ab OR female*:ti, ab)
Cochrane	([mh "Drug Therapy"] OR [mh "Drugs"] OR [mh "Antihypertensive Agents"] OR [mh "Hypolipidemic Agents"] OR medication*:ti, ab OR drug*:ti, ab OR "pharmacologic treatment":ti, ab OR pharmacotherapy:ti, ab) AND ([mh "Hypertension"] OR [mh "Antihypertensive Agents"] OR hypertension:ti, ab OR "high blood pressure":ti, ab OR "blood pressure control":ti, ab OR [mh "Dyslipidemias"] OR [mh "Hyperlipidemias"] OR dyslipidemia:ti, ab OR hyperlipidemia:ti, ab OR "lipid-lowering":ti, ab OR statins:ti, ab OR "ACE inhibitors":ti, ab OR ARBs:ti, ab OR "beta blockers":ti, ab OR diuretics:ti, ab OR "calcium channel blockers":ti, ab) AND ([mh "Cardiometabolic Diseases"] OR [mh "Metabolic Syndrome"] OR [mh "Insulin Resistance"] OR [mh "Diabetes Mellitus, Type 2"] OR [mh "Obesity"] OR cardiometabolic:ti, ab OR "metabolic syndrome":ti, ab OR "insulin resistance":ti, ab OR "glucose intolerance":ti, ab OR hyperglycemia:ti, ab OR "type 2 diabetes":ti, ab OR obesity:ti, ab OR overweight:ti, ab OR "cardiovascular risk":ti, ab) AND ([mh "Women"] OR [mh "Female"] OR women:ti, ab OR woman:ti, ab OR female*:ti, ab)
BVS	(TW: "Medicamentos" OR "Drugs" OR "Medicamentos" OR "Terapia Medicamentosa" OR "Drug Therapy" OR "Tratamiento Farmacológico" OR "Agentes Anti-hipertensivos" OR "Antihypertensive Agents" OR "Agentes Antihipertensivos" OR "Agentes Hipolipemiantes" OR "Hypolipidemic Agents" OR "Agentes Hipolipemiantes" OR "Tratamento Farmacológico" OR "Pharmacologic Treatment" OR "Tratamiento Farmacológico") AND TW: ("Hipertensão" OR "Hypertension" OR "Hipertensión" OR "Pressão Alta" OR "High Blood Pressure" OR "Presión Alta" OR "Controle da Pressão Arterial" OR "Blood Pressure Control" OR "Control de la Presión Arterial" OR "Dislipidemia" OR "Dyslipidemia" OR "Dislipidemia" OR "Hiperlipidemia" OR "Hyperlipidemia" OR "Hiperlipidemia" OR "Redução de Lipídios" OR "Lipid-Lowering" OR "Reducción de Lípidos" OR Estatinas OR Statins OR Estatinas OR "Inibidores da ECA" OR "ACE Inhibitors" OR "Inhibidores de la ECA" OR BRA OR ARBs OR BRA OR "Betabloqueadores" OR "Beta Blockers" OR "Betabloqueadores" OR Diuréticos OR Diuretics OR Diuréticos OR "Bloqueadores dos Canais de Cálcio" OR "Calcium Channel Blockers" OR "Bloqueadores de los Canales de Calcio") AND (TW: "Anormalidades Cardiometabólicas" OR "Cardiometabolic Disorders" OR "Trastornos Cardiometabólicos" OR "Síndrome Metabólica" OR "Metabolic Syndrome" OR "Síndrome Metabólica" OR "Resistência à Insulina" OR "Insulin Resistance" OR "Resistencia a la Insulina" OR "Diabetes Tipo 2" OR "Type 2 Diabetes" OR "Diabetes Tipo 2" OR "Obesidade" OR "Obesity" OR "Obesidad" OR "Sobrepeso" OR "Overweight" OR "Sobrepeso" OR "Risco Cardiovascular" OR "Cardiovascular Risk" OR "Riesgo Cardiovascular") AND (TW: "Mulheres" OR "Women" OR "Mujeres" OR "Feminino" OR "Female")

## SUPLEMENTO

**7:** A cirurgia bariátrica deve ser indicada para o tratamento das alterações cardiometabólicas nas mulheres?

Base	Estratégia
MEDLINE/PubMed	("Bariatric Surgery"[Mesh] OR "Gastric Bypass"[Mesh] OR "Gastrectomy"[Mesh] OR "bariatric surgery"[tiab] OR "gastric bypass"[tiab] OR "sleeve gastrectomy"[tiab] OR "Roux-en-Y"[tiab] OR "metabolic surgery"[tiab] OR "weight loss surgery"[tiab]) AND ("Cardiometabolic Diseases"[Mesh] OR "Metabolic Syndrome"[Mesh] OR "Insulin Resistance"[Mesh] OR "Diabetes Mellitus, Type 2"[Mesh] OR "Obesity"[Mesh] OR "Dyslipidemias"[Mesh] OR "Hypertension"[Mesh] OR cardiometabolic[tiab] OR "metabolic syndrome"[tiab] OR "insulin resistance"[tiab] OR "type 2 diabetes"[tiab] OR hyperglycemia[tiab] OR dyslipidemia[tiab] OR hyperlipidemia[tiab] OR hypertension[tiab] OR "blood pressure"[tiab] OR "cardiovascular risk"[tiab]) AND ("Women"[Mesh] OR "Female"[Mesh] OR women[tiab] OR woman[tiab] OR female*[tiab]) Filters applied: in the last 5 years, Randomized Controlled Trial, Systematic Review.
Embase	('bariatric surgery'/exp OR 'gastric bypass'/exp OR 'gastrectomy'/exp OR 'bariatric surgery':ti, ab OR 'gastric bypass':ti, ab OR 'sleeve gastrectomy':ti, ab OR 'roux-en-y':ti, ab OR 'metabolic surgery':ti, ab OR 'weight loss surgery':ti, ab) AND ('cardiometabolic disorder'/exp OR 'metabolic syndrome'/exp OR 'insulin resistance'/exp OR 'type 2 diabetes mellitus'/exp OR 'obesity'/exp OR 'dyslipidemia'/exp OR 'hypertension'/exp OR cardiometabolic:ti, ab OR 'metabolic syndrome':ti, ab OR 'insulin resistance':ti, ab OR 'type 2 diabetes':ti, ab OR hyperglycemia:ti, ab OR dyslipidemia:ti, ab OR hyperlipidemia:ti, ab OR hypertension:ti, ab OR 'blood pressure':ti, ab OR 'cardiovascular risk':ti, ab) AND ('female'/exp OR women:ti, ab OR woman:ti, ab OR female*:ti, ab)
Cochrane	([mh "Bariatric Surgery"] OR [mh "Gastric Bypass"] OR [mh "Gastrectomy"] OR "bariatric surgery":ti, ab OR "gastric bypass":ti, ab OR "sleeve gastrectomy":ti, ab OR "Roux-en-Y":ti, ab OR "metabolic surgery":ti, ab OR "weight loss surgery":ti, ab) AND ([mh "Cardiometabolic Diseases"] OR [mh "Metabolic Syndrome"] OR [mh "Insulin Resistance"] OR [mh "Diabetes Mellitus, Type 2"] OR [mh "Obesity"] OR [mh "Dyslipidemias"] OR [mh "Hypertension"] OR cardiometabolic:ti, ab OR "metabolic syndrome":ti, ab OR "insulin resistance":ti, ab OR "type 2 diabetes":ti, ab OR hyperglycemia:ti, ab OR dyslipidemia:ti, ab OR hyperlipidemia:ti, ab OR hypertension:ti, ab OR "blood pressure":ti, ab OR "cardiovascular risk":ti, ab) AND ([mh "Women"] OR [mh "Female"] OR women:ti, ab OR woman:ti, ab OR female*:ti, ab)
BVS	(TW: "Cirurgia Bariátrica" OR "Bariatric Surgery" OR "Cirugía Bariátrica" OR "Bypass Gástrico" OR "Gastric Bypass" OR "Bypass Gástrico" OR "Gastrectomia Vertical" OR "Sleeve Gastrectomy" OR "Gastrectomía Vertical" OR "Roux-en-Y" OR "Roux-en-Y" OR "Cirurgia Metabólica" OR "Metabolic Surgery" OR "Cirugía Metabólica" OR "Cirurgia para Perda de Peso" OR "Weight Loss Surgery" OR "Cirugía para Pérdida de Peso") AND (TW: "Anormalidades Cardiometabólicas" OR "Cardiometabolic Abnormalities" OR "Anormalidades Cardiometabólicas" OR "Risco Cardiometabólico" OR "Cardiometabolic Risk" OR "Riesgo Cardiometabólico" OR "Síndrome Metabólica" OR "Metabolic Syndrome" OR "Síndrome Metabólica" OR "Resistência à Insulina" OR "Insulin Resistance" OR "Resistencia a la Insulina" OR "Diabetes Tipo 2" OR "Type 2 Diabetes" OR "Diabetes Tipo 2" OR "Obesidade" OR "Obesity" OR "Obesidad" OR "Dislipidemia" OR "Dyslipidemia" OR "Dislipidemia" OR "Hipertensão" OR "Hypertension" OR "Hipertensión" OR "Risco Cardiovascular" OR "Cardiovascular Risk" OR "Riesgo Cardiovascular") AND (TW: "Mulheres" OR "Women" OR "Mujeres" OR "Feminino" OR "Female")

## SUPLEMENTO

**8:** Quais intervenções melhoram as alterações cardiometabólicas nas mulheres com doença renal crônica?

Base	Estratégia
MEDLINE/PubMed	("Life Style"[Mesh] OR "Exercise"[Mesh] OR "Diet Therapy"[Mesh] OR "Drug Therapy"[Mesh] OR intervention*[tiab] OR treatment*[tiab] OR therapy[tiab] OR management[tiab] OR lifestyle[tiab] OR diet[tiab] OR nutrition[tiab] OR "physical activity"[tiab] OR exercise[tiab] OR medication*[tiab] OR pharmacologic*[tiab]) AND ("Cardiometabolic Diseases"[Mesh] OR "Metabolic Syndrome"[Mesh] OR "Insulin Resistance"[Mesh] OR "Glucose Intolerance"[Mesh] OR "Dyslipidemias"[Mesh] OR "Hypertension"[Mesh] OR "Type 2 Diabetes Mellitus"[Mesh] OR "Obesity"[Mesh] OR cardiometabolic[tiab] OR "metabolic syndrome"[tiab] OR "insulin resistance"[tiab] OR "glucose intolerance"[tiab] OR dyslipidemia[tiab] OR hyperglycemia[tiab] OR "type 2 diabetes"[tiab] OR hypertension[tiab] OR "cardiovascular risk"[tiab]) AND ("Renal Insufficiency, Chronic"[Mesh] OR "Kidney Diseases"[Mesh] OR "chronic kidney disease"[tiab] OR CKD[tiab] OR "chronic renal failure"[tiab] OR "end-stage renal disease"[tiab] OR ESRD[tiab] OR "kidney dysfunction"[tiab]) AND ("Women"[Mesh] OR "Female"[Mesh] OR women[tiab] OR woman[tiab] OR female*[tiab]) Filters applied: in the last 5 years, Randomized Controlled Trial, Systematic Review.
Embase	('life style'/exp OR 'exercise'/exp OR 'diet therapy'/exp OR 'drug therapy'/exp OR intervention*:ti, ab OR treatment*:ti, ab OR therapy:ti, ab OR management:ti, ab OR lifestyle:ti, ab OR diet:ti, ab OR nutrition:ti, ab OR 'physical activity':ti, ab OR exercise:ti, ab OR medication*:ti, ab OR pharmacologic*:ti, ab) AND ('cardiometabolic disorder'/exp OR 'metabolic syndrome'/exp OR 'insulin resistance'/exp OR 'dyslipidemia'/exp OR 'hypertension'/exp OR 'type 2 diabetes mellitus'/exp OR 'obesity'/exp OR cardiometabolic:ti, ab OR 'metabolic syndrome':ti, ab OR 'insulin resistance':ti, ab OR hyperglycemia:ti, ab OR 'cardiovascular risk':ti, ab) AND ('chronic kidney disease'/exp OR 'renal insufficiency, chronic'/exp OR 'chronic kidney disease':ti, ab OR CKD:ti, ab OR 'chronic renal failure':ti, ab OR 'end stage renal disease':ti, ab OR ESRD:ti, ab OR 'kidney dysfunction':ti, ab OR 'kidney disease':ti, ab) AND ('female'/exp OR women:ti, ab OR woman:ti, ab OR female*:ti, ab)
Cochrane	([mh "Life Style"] OR [mh "Exercise"] OR [mh "Diet Therapy"] OR [mh "Drug Therapy"] OR intervention*:ti, ab OR treatment*:ti, ab OR therapy:ti, ab OR management:ti, ab OR lifestyle:ti, ab OR diet:ti, ab OR nutrition:ti, ab OR "physical activity":ti, ab OR exercise:ti, ab OR medication*:ti, ab OR pharmacologic*:ti, ab) AND ([mh "Cardiometabolic Diseases"] OR [mh "Metabolic Syndrome"] OR [mh "Insulin Resistance"] OR [mh "Glucose Intolerance"] OR [mh "Dyslipidemias"] OR [mh "Hypertension"] OR [mh "Type 2 Diabetes Mellitus"] OR [mh "Obesity"] OR cardiometabolic:ti, ab OR "metabolic syndrome":ti, ab OR "insulin resistance":ti, ab OR "glucose intolerance":ti, ab OR dyslipidemia:ti, ab OR hyperglycemia:ti, ab OR "type 2 diabetes":ti, ab OR hypertension:ti, ab OR "cardiovascular risk":ti, ab) AND ([mh "Renal Insufficiency, Chronic"] OR [mh "Kidney Diseases"] OR "chronic kidney disease":ti, ab OR CKD:ti, ab OR "chronic renal failure":ti, ab OR "end-stage renal disease":ti, ab OR ESRD:ti, ab OR "kidney dysfunction":ti, ab) AND ([mh "Women"] OR [mh "Female"] OR women:ti, ab OR woman:ti, ab OR female*:ti, ab)
BVS	(TW: "Intervenções" OR "Interventions" OR "Intervenciones" OR "Tratamento" OR "Treatment" OR "Tratamiento" OR "Terapia" OR "Therapy" OR "Terapia" OR "Manejo" OR "Management" OR "Manejo" OR "Estilo de Vida" OR "Lifestyle" OR "Estilo de Vida" OR "Exercício" OR "Exercise" OR "Ejercicio" OR "Atividade Física" OR "Physical Activity" OR "Actividad Física" OR "Dieta" OR "Diet" OR "Dieta" OR "Nutrição" OR "Nutrition" OR "Nutrición" OR "Terapia Nutricional" OR "Nutrition Therapy" OR "Terapia Nutricional" OR "Medicamentos" OR "Medication" OR "Medicamentos" OR "Tratamento Farmacológico" OR "Pharmacologic Treatment" OR "Tratamiento Farmacológico") AND (TW: "Doença Renal Crônica" OR "Chronic Kidney Disease" OR "Enfermedad Renal Crónica" OR "Insuficiência Renal Crônica" OR "Chronic Renal Failure" OR "Insuficiencia Renal Crónica" OR "Doença Renal Terminal" OR "End-Stage Renal Disease" OR "Enfermedad Renal Terminal" OR CKD OR ERC OR "Disfunção Renal" OR "Kidney Dysfunction" OR "Disfunción Renal" OR "Doença Renal" OR "Kidney Disease" OR "Enfermedad Renal") AND (TW: "Anormalidades Cardiometabólicas" OR "Cardiometabolic Disorders" OR "Trastornos Cardiometabólicos") AND ("Mulheres" OR "Women" OR "Mujeres")

## SUPLEMENTO

**9:** Quais intervenções melhoram as alterações cardiometabólicas nas mulheres com Doença Hepática Esteatótica Metabólica (MASLD)?

Base	Estratégia
MEDLINE/PubMed	("Life Style"[Mesh] OR "Exercise"[Mesh] OR "Diet Therapy"[Mesh] OR "Drug Therapy"[Mesh] OR intervention*[tiab] OR treatment*[tiab] OR therapy[tiab] OR management[tiab] OR lifestyle[tiab] OR diet[tiab] OR nutrition[tiab] OR "physical activity"[tiab] OR exercise[tiab] OR medication*[tiab] OR pharmacologic*[tiab]) AND ("Cardiometabolic Diseases"[Mesh] OR "Metabolic Syndrome"[Mesh] OR "Insulin Resistance"[Mesh] OR "Glucose Intolerance"[Mesh] OR "Dyslipidemias"[Mesh] OR "Hypertension"[Mesh] OR "Type 2 Diabetes Mellitus"[Mesh] OR cardiometabolic[tiab] OR "metabolic syndrome"[tiab] OR "insulin resistance"[tiab] OR "glucose intolerance"[tiab] OR dyslipidemia[tiab] OR hyperglycemia[tiab] OR "type 2 diabetes"[tiab] OR hypertension[tiab] OR "cardiovascular risk"[tiab]) AND ("Non-alcoholic Fatty Liver Disease"[Mesh] OR "Fatty Liver"[Mesh] OR MASLD[tiab] OR "metabolic dysfunction-associated steatotic liver disease"[tiab] OR NAFLD[tiab] OR "nonalcoholic fatty liver disease"[tiab] OR "fatty liver"[tiab] OR "hepatic steatosis"[tiab] OR steatosis[tiab] OR steatohepatitis[tiab] OR NASH[tiab]) AND ("Women"[Mesh] OR "Female"[Mesh] OR women[tiab] OR woman[tiab] OR female*[tiab]) Filters applied: in the last 5 years, Randomized Controlled Trial, Systematic Review.
Embase	('life style'/exp OR 'exercise'/exp OR 'diet therapy'/exp OR 'drug therapy'/exp OR intervention*:ti, ab OR treatment*:ti, ab OR therapy:ti, ab OR management:ti, ab OR lifestyle:ti, ab OR diet:ti, ab OR nutrition:ti, ab OR 'physical activity':ti, ab OR exercise:ti, ab OR medication*:ti, ab OR pharmacologic*:ti, ab) AND ('cardiometabolic disorder'/exp OR 'metabolic syndrome'/exp OR 'insulin resistance'/exp OR 'dyslipidemia'/exp OR 'hypertension'/exp OR 'type 2 diabetes mellitus'/exp OR cardiometabolic:ti, ab OR 'metabolic syndrome':ti, ab OR 'insulin resistance':ti, ab OR hyperglycemia:ti, ab OR 'cardiovascular risk':ti, ab) AND ('nonalcoholic fatty liver'/exp OR 'fatty liver'/exp OR MASLD:ti, ab OR 'metabolic dysfunction associated steatotic liver disease':ti, ab OR NAFLD:ti, ab OR 'nonalcoholic fatty liver disease':ti, ab OR 'fatty liver':ti, ab OR 'hepatic steatosis':ti, ab OR steatosis:ti, ab OR steatohepatitis:ti, ab OR NASH:ti, ab) AND ('female'/exp OR women:ti, ab OR woman:ti, ab OR female*:ti, ab)
Cochrane	([mh "Life Style"] OR [mh "Exercise"] OR [mh "Diet Therapy"] OR [mh "Drug Therapy"] OR intervention*:ti, ab OR treatment*:ti, ab OR therapy:ti, ab OR management:ti, ab OR lifestyle:ti, ab OR diet:ti, ab OR nutrition:ti, ab OR "physical activity":ti, ab OR exercise:ti, ab OR medication*:ti, ab OR pharmacologic*:ti, ab) AND ([mh "Cardiometabolic Diseases"] OR [mh "Metabolic Syndrome"] OR [mh "Insulin Resistance"] OR [mh "Glucose Intolerance"] OR [mh "Dyslipidemias"] OR [mh "Hypertension"] OR [mh "Type 2 Diabetes Mellitus"] OR cardiometabolic:ti, ab OR "metabolic syndrome":ti, ab OR "insulin resistance":ti, ab OR "glucose intolerance":ti, ab OR dyslipidemia:ti, ab OR hyperglycemia:ti, ab OR "type 2 diabetes":ti, ab OR hypertension:ti, ab OR "cardiovascular risk":ti, ab) AND ([mh "Non-alcoholic Fatty Liver Disease"] OR [mh "Fatty Liver"] OR MASLD:ti, ab OR "metabolic dysfunction-associated steatotic liver disease":ti, ab OR NAFLD:ti, ab OR "nonalcoholic fatty liver disease":ti, ab OR "fatty liver":ti, ab OR "hepatic steatosis":ti, ab OR steatosis:ti, ab OR steatohepatitis:ti, ab OR NASH:ti, ab) AND ([mh "Women"] OR [mh "Female"] OR women:ti, ab OR woman:ti, ab OR female*:ti, ab)
BVS	(TW: "Intervenções" OR "Interventions" OR "Intervenciones" OR "Tratamento" OR "Treatment" OR "Tratamiento" OR "Terapia" OR "Therapy" OR "Terapia" OR "Manejo" OR "Management" OR "Manejo" OR "Estilo de Vida" OR "Lifestyle" OR "Estilo de Vida" OR "Exercício" OR "Exercise" OR "Ejercicio" OR "Atividade Física" OR "Physical Activity" OR "Actividad Física" OR "Dieta" OR "Diet" OR "Dieta" OR "Nutrição" OR "Nutrition" OR "Nutrición" OR "Terapia Nutricional" OR "Nutrition Therapy" OR "Terapia Nutricional" OR "Medicamentos" OR "Medication" OR "Medicamentos" OR "Tratamento Farmacológico" OR "Pharmacologic Treatment" OR "Tratamiento Farmacológico") AND (TW: "Doença Hepática Esteatótica Metabólica" OR "Metabolic Dysfunction-Associated Steatotic Liver Disease" OR "Enfermedad Hepática Esteatótica Metabólica" OR MASLD OR "Doença Hepática Gordurosa Não Alcoólica" OR "Nonalcoholic Fatty Liver Disease" OR "Enfermedad Hepática Grasa No Alcohólica" OR NAFLD OR "Esteatose Hepática" OR "Fatty Liver" OR "Esteatosis Hepática" OR "Esteato-hepatite" OR "Steatohepatitis" OR "Esteatohepatitis" OR NASH) AND (TW: "Anormalidades Cardiometabólicas" OR "Cardiometabolic Disorders" OR "Trastornos Cardiometabólicos") AND ("Mulheres" OR "Women" OR "Mujeres")

## SUPLEMENTO

**10:** Mulheres com endometriose e SOP precisam verificar fatores de risco cardiovasculares regularmente para evitar DCV?

Base	Estratégia
MEDLINE/PubMed	("Endometriosis"[Mesh] OR endometriosis[tiab]) OR ("Polycystic Ovary Syndrome"[Mesh] OR "Stein-Leventhal Syndrome"[Mesh] OR "ovarian dysfunction"[tiab] OR PCOS[tiab] OR "polycystic ovary syndrome"[tiab]) AND ("Risk Factors"[Mesh] OR "Cardiovascular Risk"[tiab] OR "Metabolic Syndrome"[Mesh] OR "Insulin Resistance"[Mesh] OR "Obesity"[Mesh] OR "Hypertension"[Mesh] OR "Dyslipidemias"[Mesh] OR "risk factor*"[tiab] OR "metabolic risk"[tiab] OR "insulin resistance"[tiab] OR dyslipidemia[tiab] OR obesity[tiab] OR overweight[tiab] OR "blood pressure"[tiab] OR hypertension[tiab] OR inflammation[tiab]) AND ("Cardiovascular Diseases"[Mesh] OR "cardiovascular disease*"[tiab] OR CVD[tiab] OR "heart disease*"[tiab] OR "cardiac disease*"[tiab] OR atherosclerosis[tiab] OR "coronary artery disease"[tiab] OR "myocardial infarction"[tiab] OR stroke[tiab] OR "ischemic heart disease"[tiab]) Filters applied: in the last 5 years, Randomized Controlled Trial, Systematic Review.
Embase	('endometriosis'/exp OR endometriosis:ti, ab) OR ('polycystic ovary syndrome'/exp OR 'stein leventhal syndrome'/exp OR PCOS:ti, ab OR 'polycystic ovary syndrome':ti, ab OR 'ovarian dysfunction':ti, ab) AND ('risk factor'/exp OR 'cardiovascular risk':ti, ab OR 'metabolic syndrome'/exp OR 'insulin resistance'/exp OR 'obesity'/exp OR 'hypertension'/exp OR 'dyslipidemia'/exp OR 'blood pressure':ti, ab OR inflammation:ti, ab) AND ('cardiovascular disease'/exp OR 'heart disease':ti, ab OR 'cardiac disease':ti, ab OR atherosclerosis:ti, ab OR 'coronary artery disease':ti, ab OR 'myocardial infarction':ti, ab OR stroke:ti, ab OR 'ischemic heart disease':ti, ab OR CVD:ti, ab)
Cochrane	([mh "Endometriosis"] OR endometriosis:ti, ab) OR ([mh "Polycystic Ovary Syndrome"] OR [mh "Stein-Leventhal Syndrome"] OR "ovarian dysfunction":ti, ab OR PCOS:ti, ab OR "polycystic ovary syndrome":ti, ab) AND ([mh "Risk Factors"] OR "cardiovascular risk":ti, ab OR [mh "Metabolic Syndrome"] OR [mh "Insulin Resistance"] OR [mh "Obesity"] OR [mh "Hypertension"] OR [mh "Dyslipidemias"] OR "risk factor*":ti, ab OR "metabolic risk":ti, ab OR "insulin resistance":ti, ab OR dyslipidemia:ti, ab OR obesity:ti, ab OR overweight:ti, ab OR "blood pressure":ti, ab OR hypertension:ti, ab OR inflammation:ti, ab) AND ([mh "Cardiovascular Diseases"] OR "cardiovascular disease*":ti, ab OR CVD:ti, ab OR "heart disease*":ti, ab OR "cardiac disease*":ti, ab OR atherosclerosis:ti, ab OR "coronary artery disease":ti, ab OR "myocardial infarction":ti, ab OR stroke:ti, ab OR "ischemic heart disease":ti, ab)
BVS	(TW: "Endometriose" OR "Endometriosis" OR "Endometriosis") OR ("Síndrome dos Ovários Policísticos" OR "Polycystic Ovary Syndrome" OR "Síndrome de Ovario Poliquístico" OR SOP OR PCOS OR "Síndrome de Stein-Leventhal" OR "Stein-Leventhal Syndrome" OR "Síndrome de Stein-Leventhal" OR "Disfunção Ovariana" OR "Ovarian Dysfunction" OR "Disfunción Ovárica") AND (TW: "Fatores de Risco" OR "Risk Factors" OR "Factores de Riesgo" OR "Risco Cardiovascular" OR "Cardiovascular Risk" OR "Riesgo Cardiovascular" OR "Síndrome Metabólica" OR "Metabolic Syndrome" OR "Síndrome Metabólica" OR "Resistência à Insulina" OR "Insulin Resistance" OR "Resistencia a la Insulina" OR "Dislipidemia" OR "Dyslipidemia" OR "Dislipidemia" OR "Obesidade" OR "Obesity" OR "Obesidad" OR "Sobrepeso" OR "Overweight" OR "Sobrepeso" OR "Pressão Arterial" OR "Blood Pressure" OR "Presión Arterial" OR "Hipertensão" OR "Hypertension" OR "Hipertensión" OR "Intolerância à Glicose" OR "Glucose Intolerance" OR "Intolerancia a la Glucosa" OR "Hiperglicemia" OR "Hyperglycemia" OR "Hiperglucemia" OR "Inflamação" OR "Inflammation" OR "Inflamación") AND (TW: "Doenças Cardiovasculares" OR "Cardiovascular Diseases" OR "Enfermedades Cardiovasculares" OR "Doença Isquêmica do Coração" OR "Ischemic Heart Disease" OR "Enfermedad Isquémica del Corazón" OR "Doença Arterial Coronariana" OR "Coronary Artery Disease" OR "Enfermedad de las Arterias Coronarias" OR "Aterosclerose" OR "Atherosclerosis" OR "Aterosclerosis" OR "Infarto do Miocárdio" OR "Myocardial Infarction" OR "Infarto de Miocardio" OR "Acidente Vascular Cerebral" OR "Stroke" OR "Accidente Cerebrovascular")

## Lista de Abreviaturas e Siglas

–AVC: acidente vascular cerebral
–BRA: bloqueadores dos receptores da angiotensina II
–CAC: escore de cálcio coronariano
–CBG: *cortisol-binding-globulin*
–CHIP: hematopoiese clonal de potencial indeterminado
–COC: contraceptivos orais combinados
–DALYs: *Disability-Adjusted Life Years* - Anos de Vida Ajustados por Incapacidade
–DCM/SBC: Departamento de Cardiologia da Mulher da Sociedade Brasileira de Cardiologia
–DCV: doenças cardiovasculares
–DG: diabetes gestacional
–DHEM: doença hepática esteatótica metabólica
–DIC: doença isquêmica do coração
–DIU-LNG: sistema intrauterino liberador de levonorgestrel
–DM: diabetes *mellitus*
–DM1: diabetes *mellitus* tipo 1
–DM2: diabetes *mellitus* tipo 2
–DRC: doença renal crônica
–DRD: doença renal diabética
–EHM: esteato-hepatite metabólica
–eNOS: enzima óxido nítrico sintase endotelial
–ESC: Sociedade Europeia de Cardiologia
–FAI: índice de atenuação da gordura perivascular
–FEBRASGO: Federação Brasileira das Associações de Ginecologia e Obstetrícia
–FIB-4: *Fibrosis-4*
–FR: fatores de risco
–FRCV: fatores de risco cardiovascular
–FSH: hormônio folículo-estimulante
–GIP: polipeptídeo insulinotrópico dependente de glicose
–GLP-1: peptídeo semelhante ao glucagon 1 ( *glucagon-like peptide-1* )
–GnRH: hormônio liberador de gonadotrofinas
–GPG: ganho de peso gestacional
–HAS: hipertensão arterial sistêmica
–HbA1c: hemoglobina glicada
–HDL-c: colesterol de lipoproteína de alta densidade
–HOMA-IR: *homeostasis model assessment of insulin resistance*
–IAM: infarto agudo do miocárdio
–IC: insuficiência cardíaca
–ICFEp: insuficiência cardíaca com fração de ejeção preservada
–IECA: inibidores da enzima conversora de angiotensina
–IL: interleucina
–IM: infarto do miocárdio
–IMC: índice de massa corpórea
–INOCA: isquemia com artérias coronárias normais
–IP-10: proteína 10 induzível por interferon gama
–LDL-c: colesterol de lipoproteína de baixa densidade
–LH: hormônio luteinizante
–Lp(a): lipoproteína a
–MCP-1: proteína quimiotática de monócitos 1
–MINOCA: infarto do miocárdio com artérias coronárias normais
–NF-κB: fator nuclear kappa B
–ON: óxido nítrico
–PA: pressão arterial
–PCR: proteína C reativa
–PCR-us: PCR ultrassensível
–RCE: razão cintura-estatura
–RCQ: razão cintura-quadril
–RCV: risco cardiovascular
–RI: resistência à insulina
–ROS: espécies reativas de oxigênio
–RR: risco relativo
–SBEM: Sociedade Brasileira de Endocrinologia e Metabologia
–sEng: endoglina solúvel
–sFlt-1: fator solúvel tipo tirosinaquinase-1
–SGLT2: cotransportador de sódio-glicose tipo 2
–SHBG: *sexual-hormone-binding-globulin*
–SM: síndrome metabólica
–SOP: síndrome dos ovários policísticos
–SVM: sintomas vasomotores
–TEV: tromboembolismo venoso
–THM: terapia de reposição hormonal da menopausa
–TNF-α: fator de necrose tumoral alfa
–TOTG: teste oral de tolerância à glicose
–VCAM-1: molécula de adesão celular vascular
–VEGF: fator de crescimento endotelial vascular
–VLDL-c: colesterol de lipoproteína de muito baixa densidade
–WHI: *Women's Health Initiative*
