# Supplementary material for: Posicionamento sobre a Saúde Cardiometabólica ao Longo do Ciclo de Vida da Mulher – 2025
Source: Arq Bras Cardiol. 2025 Sep 17;122(9):e20250615. [Article in Portuguese] doi: 10.36660/abc.20250615 (PMC12674840; doi:10.36660/abc.20250615)
Supplement: Translation

# Translation: Position Statement on Cardiometabolic Health Across the Woman's Life Course – 2025

**DOI:** 10.36660/abc.20250615i

**Authors:** Gláucia Maria Moraes de Oliveira, Maria Cristina Costa de Almeida, Cynthia Melissa Valério, Fernando Giuffrida, Larissa Neto Espíndola, Maria Cristina de Oliveira Izar, Celi Marques-Santos, Claudia Maria Vilas Freire, Carlos Japhet da Matta Albuquerque, Antonio Carlos Pallandri Chagas, Dalton Bertolim Précoma, Evandro Tinoco Mesquita, José Francisco Kerr Saraiva, Maria Elizabeth Navegantes Caetano Costa, Viviana de Mello Guzzo Lemke, Alexandre Jorge Gomes de Lucena, Andréa Araujo Brandão, Antonio Aurelio de Paiva Fagundes, Ariane Vieira Scarlatelli Macedo, Carisi Anne Polanczyk, Cristiane Bauermann Leitão, Daniel Souto Silveira, Elaine dos Reis Coutinho, Eliana Aguiar Petri Nahas, Elizabeth Regina Giunco Alexandre, Erika Maria Gonçalves Campana, Erika Olivier Vilela Bragança, Fernanda Marciano Consolim Colombo, Imara Correia de Queiroz Barbosa, Ivan Romero Rivera, Jaime Kulak, João Eduardo Nunes Salles, João Roberto de Sá, José Maria Soares, Larissa de Almeida Dourado, Lidia Zytynski Moura, Lucelia Batista Neves Cunha Magalhães, Luciano de Melo Pompei, Luiz Guilherme Passaglia, Marcelo Heitor Vieira Assad, Marcio Alexandre Hipólito Rodrigues, Maria Alayde Mendonça Rivera, Maria Antonieta Albanez Albuquerque de Medeiros Lopes, Maria Sanali Moura de Oliveira Paiva, Marildes Luiza de Castro, Milena dos Santos Barros Campos, Olga Ferreira de Souza, Orlando Otávio de Medeiros, Rafaela Andrade Penalva Freitas, Regina Coeli Marques de Carvalho, Sheyla Cristina Tonheiro Ferro da Silva, Thais de Carvalho Vieira Rodrigues, Walkiria Samuel Avila, Wellington Santana da Silva, Willyan Issamu Nazima, Lucia Helena Simões da Costa-Paiva, Maria Celeste Osorio Wender

**Affiliations:** 1 Rio de Janeiro RJ Brazil Universidade Federal do Rio de Janeiro (UFRJ),x Rio de Janeiro, RJ – Brazil; 2 Belo Horizonte MG Brazil Hospital João XXIII, Fundação Hospitalar do Estado de Minas Gerais, Belo Horizonte, MG – Brazil; 3 Belo Horizonte MG Brazil Centro Universitário de Belo Horizonte (UNIBH), Belo Horizonte, MG – Brazil; 4 Rio de Janeiro RJ Brazil Instituto Estadual de Diabetes e Endocrinologia Luiz Capriglione (IEDE-RJ), Rio de Janeiro, RJ – Brazil; 5 Salvador BA Brazil Universidade do Estado da Bahia, Salvador, BA – Brazil; 6 Salvador BA Brazil Hospital Santa Izabel, Salvador, BA – Brazil; 7 Salvador BA Brazil Hospital Municipal de Salvador, Salvador, BA – Brazil; 8 São Paulo SP Brazil Universidade Federal de São Paulo (UNIFESP), São Paulo, SP – Brazil; 9 Aracaju SE Brazil Universidade Tiradentes, Aracaju, SE – Brazil; 10 Aracaju SE Brazil Hospital São Lucas, Rede D’Or São Luiz, Aracaju, SE – Brazil; 11 Belo Horizonte MG Brazil Universidade Federal de Minas Gerais (UFMG), Belo Horizonte, MG –Brazil; 12 Recife PE Brazil Hospital Santa Joana Recife – Rede Américas, Recife PE – Brazil; 13 Recife PE Brazil Hospital Barão de Lucena – SUS/PE, Recife PE – Brazil; 14 Santo André SP Brazil Centro Universitário Faculdade de Medicina do ABC, Santo André, SP – Brazil; 15 São Paulo SP Brazil Instituto do Coração (Incor) do Hospital das Clínicas da Faculdade de Medicina da Universidade de São Paulo (HCFMUSP), São Paulo, SP – Brazil; 16 Curitiba PR Brazil Sociedade Hospitalar Angelina Caron, Curitiba, PR – Brazil; 17 Rio de Janeiro RJ Brazil Universidade Federal Fluminense (UFF), Rio de Janeiro, RJ – Brazil; 18 Campinas SP Brazil Pontifícia Universidade Católica de Campinas, Campinas, SP – Brazil; 19 Belém PA Brazil Centro Universitário do Estado Pará (CESUPA), Belém PA – Brazil; 20 Curitiba PR Brazil Cardiocare-Clínica Cardiológica Ltda, Curitiba, PR – Brazil; 21 Recife PE Brazil Hospital Agamenon Magalhães, Recife, PE – Brazil; 22 Rio de Janeiro RJ Brazil Universidade do Estado do Rio de Janeiro (UERJ), Rio de Janeiro, RJ – Brazil; 23 Brasília DF Brazil Instituto D’Or de Pesquisa e Ensino (IDOR), Brasília, DF – Brazil; 24 Brasília DF Brazil Universidade de Brasília (UNB), Brasília, DF – Brazil; 25 Brasília DF Brazil Hospital DFStar, Brasília, DF – Brazil; 26 São Paulo SP Brazil Santa Casa de Misericórdia de São Paulo, São Paulo, SP – Brazil; 27 Porto Alegre RS Brazil Hospital de Clínicas da Universidade Federal do Rio Grande do Sul (UFRS), Porto Alegre, RS – Brazil; 28 Porto Alegre RS Brazil Universidade Federal do Rio Grande do Sul, Porto Alegre, RS – Brazil; 29 Porto Alegre RS Brazil Instituto de Medicina Vascular – Hospital Mãe de Deus, Porto Alegre, RS – Brazil; 30 Campinas SP Brazil Hospital PUC Campinas, Campinas, SP – Brazil; 31 Botucatu SP Brazil Faculdade de Medicina de Botucatu, Universidade Estadual Paulista Julio Mesquita Filho (UNESP), Botucatu, SP – Brazil; 32 São Paulo SP Brazil Hospital do Coração (HCor), São Paulo, SP – Brazil; 33 São José dos Campos SP Brazil RitmoCheck, São José dos Campos, SP – Brazil; 34 Campina Grande PB Brazil Universidade Federal de Campina Grande, Campina Grande, PB – Brazil; 35 Maceió AL Brazil Universidade Federal de Alagoas, Maceió, AL – Brazil; 36 Curitiba PR Brazil Universidade Federal do Paraná, Curitiba, PR – Brazil; 37 São Paulo SP Brazil Faculdade de Ciências Médicas da Santa Casa de São Paulo, São Paulo, SP – Brazil; 38 São Paulo SP Brazil Faculdade de Medicina da Universidade de São Paulo, Hospital das Clínicas, São Paulo, SP – Brazil; 39 Curitiba PR Brazil Pontifícia Universidade Católica do Paraná, Curitiba, PR – Brazil; 40 Salvador BA Brazil Faculdade de Medicina da Unesulbahia, Salvador, BA – Brazil; 41 Belo Horizonte MG Brazil Hospital das Clínicas da Universidade Federal de Minas Gerais (UFMG), Belo Horizonte, MG – Brazil; 42 Rio de Janeiro RJ Brazil Instituto Nacional de Cardiologia, Rio de Janeiro, RJ – Brazil; 43 Belo Horizonte MG Brazil Universidade Federal de Minas Gerais (UFMG), Belo Horizonte, MG – Brazil; 44 Recife PE Brazil Real Hospital Português, Recife, PE – Brazil; 45 Recife PE Brazil Hospital São Marcos, Recife, PE – Brazil; 46 Natal RN Brazil Cardiointerve, Natal, RN – Brazil; 47 Aracaju SE Brazil Hospital Universitário da Universidade Federal de Sergipe, Aracaju, SE – Brazil; 48 Brasília DF Brazil Ministério da Saúde, Brasília, DF – Brazil; 49 São Paulo SP Brazil Instituto Dante Pazzanese de Cardiologia, São Paulo, SP – Brazil; 50 Fortaleza CE Brazil Hospital Geral de Fortaleza, Fortaleza, CE – Brazil; 51 Fortaleza CE Brazil Secretaria da Saúde do Ceará, Fortaleza, CE – Brazil; 52 Aracaju SE Brazil CEMISE Oncoclínicas, Aracaju, SE – Brazil; 53 Aracaju SE Brazil Prefeitura Municipal de Aracaju, Aracaju, SE – Brazil; 54 Aracaju SE Brazil Universidade Federal de Sergipe (UFS), Aracaju, SE – Brazil; 55 São Luís MA Brazil Universidade Federal do Maranhão (UFMA), São Luís, MA – Brazil; 56 Londrina PR Brazil Hospital Evangélico de Londrina, Londrina, PR – Brazil; 57 Campinas SP Brazil Universidade Estadual de Campinas (UNICAMP), Campinas, SP – Brazil

**Table:** 

Position Statement on Cardiometabolic Health Across the Woman's Life Course – 2025	
The report below lists declarations of interest as reported to the SBC by the experts during the period of the development of these statement, 2024/2025.	
Expert	Type of relationship with industry
Alexandre Jorge Gomes de Lucena	Nothing to be declared
Antonio Aurelio de Paiva Fagundes Junior	Financial declaration A - Economically relevant payments of any kind made to (i) you, (ii) your spouse/partner or any other person living with you, (iii) any legal person in which any of these is either a direct or indirect controlling owner, business partner, shareholder or participant; any payments received for lectures, lessons, training instruction, compensation, fees paid for participation in advisory boards, investigative boards or other committees, etc. from the brazilian or international pharmaceutical, orthosis, prosthesis, equipment and implants industry: AstraZeneca: Lokelma; Boehringer-Ingelheim: Metalyse; Novartis: Sybrava; Abbot: Ritmonorm; Mundipharma: Rezzayo; Libbs: Plenance. B - Research funding under your direct/personal responsibility (directed to the department or institution) from the brazilian or international pharmaceutical, orthosis, prosthesis, equipment and implants industry: Novartis: renal failure; Eli lilly: Lipoprotein A.
Antonio Carlos Palandri Chagas	Financial declaration A - Economically relevant payments of any kind made to (i) you, (ii) your spouse/partner or any other person living with you, (iii) any legal person in which any of these is either a direct or indirect controlling owner, business partner, shareholder or participant; any payments received for lectures, lessons, training instruction, compensation, fees paid for participation in advisory boards, investigative boards or other committees, etc. from the brazilian or international pharmaceutical, orthosis, prosthesis, equipment and implants industry: Vita Institute.
Ariane Vieira Scarlatelli Macedo	Financial declaration A - Economically relevant payments of any kind made to (i) you, (ii) your spouse/partner or any other person living with you, (iii) any legal person in which any of these is either a direct or indirect controlling owner, business partner, shareholder or participant; any payments received for lectures, lessons, training instruction, compensation, fees paid for participation in advisory boards, investigative boards or other committees, etc. from the brazilian or international pharmaceutical, orthosis, prosthesis, equipment and implants industry: Bayer: anticoagulation and heart failure; Pfizer: anticoagulation and amyloidosis; Jannsen: leukemia. Other relationships Funding of continuing medical education activities, including travel, accommodation and registration in conferences and courses, from the brazilian or international pharmaceutical, orthosis, prosthesis, equipment and implants industry: - Bayer: heart failure.
Carisi Anne Polanczyk	Nothing to be declared
Carlos Japhet da Matta Albuquerque	Nothing to be declared
Celi Marques Santos	Nothing to be declared
Claudia Maria Vilas Freire	Nothing to be declared
Cristiane Bauermann Leitão	Financial declaration A - Economically relevant payments of any kind made to (i) you, (ii) your spouse/partner or any other person living with you, (iii) any legal person in which any of these is either a direct or indirect controlling owner, business partner, shareholder or participant; any payments received for lectures, lessons, training instruction, compensation, fees paid for participation in advisory boards, investigative boards or other committees, etc. from the brazilian or international pharmaceutical, orthosis, prosthesis, equipment and implants industry: Novo Nordisk. Other relationships Funding of continuing medical education activities, including travel, accommodation and registration in conferences and courses, from the brazilian or international pharmaceutical, orthosis, prosthesis, equipment and implants industry: - Lilly.
Cynthia Melissa Valerio	Financial declaration A - Economically relevant payments of any kind made to (i) you, (ii) your spouse/partner or any other person living with you, (iii) any legal person in which any of these is either a direct or indirect controlling owner, business partner, shareholder or participant; any payments received for lectures, lessons, training instruction, compensation, fees paid for participation in advisory boards, investigative boards or other committees, etc. from the brazilian or international pharmaceutical, orthosis, prosthesis, equipment and implants industry: Novo Nordisk: Diabetes e Obesidade; Boehringer Ingelheim: Diabetes; Eli Lilly: Diabetes e Obesidade; AstraZeneca: Diabetes; EMS: Diabetes e Obesidade. B - Research funding under your direct/personal responsibility (directed to the department or institution) from the brazilian or international pharmaceutical, orthosis, prosthesis, equipment and implants industry: Chiesi: metreleptina. Other relationships Funding of continuing medical education activities, including travel, accommodation and registration in conferences and courses, from the brazilian or international pharmaceutical, orthosis, prosthesis, equipment and implants industry: - Chiesi: metreleptina.
Daniel Souto Silveira	Nothing to be declared
Dalton Bertolim Precoma	Financial declaration B - Research funding under your direct/personal responsibility (directed to the department or institution) from the brazilian or international pharmaceutical, orthosis, prosthesis, equipment and implants industry: Janssen: anticoagulação; Astrazeneca: dislipidemia e inibidores da aldosterona; Novonordisk: insuficiência cardíaca; Arrowhead: dislipidemia; Vertrix: antiagregação plaquetária. Other relationships Funding of continuing medical education activities, including travel, accommodation and registration in conferences and courses, from the brazilian or international pharmaceutical, orthosis, prosthesis, equipment and implants industry: - Daiichi Sankyo: anticoagulation; GSK: vaccines; Astrazeneca: cardiometabolism.
Elaine dos Reis Coutinho	Financial declaration A - Economically relevant payments of any kind made to (i) you, (ii) your spouse/partner or any other person living with you, (iii) any legal person in which any of these is either a direct or indirect controlling owner, business partner, shareholder or participant; any payments received for lectures, lessons, training instruction, compensation, fees paid for participation in advisory boards, investigative boards or other committees, etc. from the brazilian or international pharmaceutical, orthosis, prosthesis, equipment and implants industry: Novartis: Sybrava; Biolab: Livalo e Repatha; Daichii sankyo: Nustendi. Other relationships Funding of continuing medical education activities, including travel, accommodation and registration in conferences and courses, from the brazilian or international pharmaceutical, orthosis, prosthesis, equipment and implants industry: - Novartis, Daichii, Biolab.
Eliana Aguiar Petri Nahas	Financial declaration A - Economically relevant payments of any kind made to (i) you, (ii) your spouse/partner or any other person living with you, (iii) any legal person in which any of these is either a direct or indirect controlling owner, business partner, shareholder or participant; any payments received for lectures, lessons, training instruction, compensation, fees paid for participation in advisory boards, investigative boards or other committees, etc. from the brazilian or international pharmaceutical, orthosis, prosthesis, equipment and implants industry: Libbs: Yumi, Iziz, Libiam, Natifa; Theramex: Estreva Gel, linha Systen; Exeltis: Gynpro; Gedeon: Lenzetto; Besins: Oestrogel; Astellas: Veoza.
Elizabeth Regina Giunco Alexandre	Financial declaration A - Economically relevant payments of any kind made to (i) you, (ii) your spouse/partner or any other person living with you, (iii) any legal person in which any of these is either a direct or indirect controlling owner, business partner, shareholder or participant; any payments received for lectures, lessons, training instruction, compensation, fees paid for participation in advisory boards, investigative boards or other committees, etc. from the brazilian or international pharmaceutical, orthosis, prosthesis, equipment and implants industry: Servier: Vastarel MR; Lilly: Mounjaro; Libbs: Ebatz e Stanglitz, NovoNordisk: Ozempic; Astra Zeneca: Breztri; Boehringer-Ingelhein: Glyxambi; Mantecorpp: Nesina/Addera. Other relationships Funding of continuing medical education activities, including travel, accommodation and registration in conferences and courses, from the brazilian or international pharmaceutical, orthosis, prosthesis, equipment and implants industry: - Lilly.
Erika Maria Gonçalves Campana	Financial declaration A - Economically relevant payments of any kind made to (i) you, (ii) your spouse/partner or any other person living with you, (iii) any legal person in which any of these is either a direct or indirect controlling owner, business partner, shareholder or participant; any payments received for lectures, lessons, training instruction, compensation, fees paid for participation in advisory boards, investigative boards or other committees, etc. from the brazilian or international pharmaceutical, orthosis, prosthesis, equipment and implants industry: Servier, Brace Pharma, Biolab, Momenta. Other relationships Funding of continuing medical education activities, including travel, accommodation and registration in conferences and courses, from the brazilian or international pharmaceutical, orthosis, prosthesis, equipment and implants industry: - Servier, Biolab: hypertension.
Érika Olivier Vilela Bragança	Financial declaration A - Economically relevant payments of any kind made to (i) you, (ii) your spouse/partner or any other person living with you, (iii) any legal person in which any of these is either a direct or indirect controlling owner, business partner, shareholder or participant; any payments received for lectures, lessons, training instruction, compensation, fees paid for participation in advisory boards, investigative boards or other committees, etc. from the brazilian or international pharmaceutical, orthosis, prosthesis, equipment and implants industry: Biolab: Dozoito. Other relationships Funding of continuing medical education activities, including travel, accommodation and registration in conferences and courses, from the brazilian or international pharmaceutical, orthosis, prosthesis, equipment and implants industry: - Merck.
Evandro Tinoco Mesquita	Financial declaration A - Economically relevant payments of any kind made to (i) you, (ii) your spouse/partner or any other person living with you, (iii) any legal person in which any of these is either a direct or indirect controlling owner, business partner, shareholder or participant; any payments received for lectures, lessons, training instruction, compensation, fees paid for participation in advisory boards, investigative boards or other committees, etc. from the brazilian or international pharmaceutical, orthosis, prosthesis, equipment and implants industry: Ache: Astra educational material and classes. Other relationships Funding of continuing medical education activities, including travel, accommodation and registration in conferences and courses, from the brazilian or international pharmaceutical, orthosis, prosthesis, equipment and implants industry: - Pfizer: amyloidosis.
Fernanda Marciano Consolim Colombo	Financial declaration A - Economically relevant payments of any kind made to (i) you, (ii) your spouse/partner or any other person living with you, (iii) any legal person in which any of these is either a direct or indirect controlling owner, business partner, shareholder or participant; any payments received for lectures, lessons, training instruction, compensation, fees paid for participation in advisory boards, investigative boards or other committees, etc. from the brazilian or international pharmaceutical, orthosis, prosthesis, equipment and implants industry: Daiichi Sankyo; Merck; Servier; AstraZeneca. Other relationships Funding of continuing medical education activities, including travel, accommodation and registration in conferences and courses, from the brazilian or international pharmaceutical, orthosis, prosthesis, equipment and implants industry: - Daiichi Sankyo; Servier.
Fernando M. A. Giuffrida	Nothing to be declared
Gláucia Maria Moraes de Oliveira	Nothing to be declared
Imara Correia de Queiroz Barbosa	Financial declaration A - Economically relevant payments of any kind made to (i) you, (ii) your spouse/partner or any other person living with you, (iii) any legal person in which any of these is either a direct or indirect controlling owner, business partner, shareholder or participant; any payments received for lectures, lessons, training instruction, compensation, fees paid for participation in advisory boards, investigative boards or other committees, etc. from the brazilian or international pharmaceutical, orthosis, prosthesis, equipment and implants industry: AstraZeneca: heart failure (Forxiga, Selozok); Servier: hypertension (Triplixam).
Ivan Romero Rivera	Nothing to be declared
Jaime Kulak Junior	Financial declaration A - Economically relevant payments of any kind made to (i) you, (ii) your spouse/partner or any other person living with you, (iii) any legal person in which any of these is either a direct or indirect controlling owner, business partner, shareholder or participant; any payments received for lectures, lessons, training instruction, compensation, fees paid for participation in advisory boards, investigative boards or other committees, etc. from the brazilian or international pharmaceutical, orthosis, prosthesis, equipment and implants industry: Bayer: Mirena; Besins: Vagifem; Biolab: Qlaira; Theramex: Systen, Estreva; Merck: Glifage XR; Astellas: Fezolinetanto. Other relationships Funding of continuing medical education activities, including travel, accommodation and registration in conferences and courses, from the brazilian or international pharmaceutical, orthosis, prosthesis, equipment and implants industry: Astellas: Congress of the International Menopause Society; Besins: Congress of FIGO and Congress of the International Menopause Society.
João Eduardo Nunes Salles	Nothing to be declared
João Roberto de Sá	Nothing to be declared
José Francisco Kerr Saraiva	Financial declaration C - Personal research funding paid by the brazilian or international pharmaceutical, orthosis, prosthesis, equipment and implants industry: Bayer: finerinone; Novo Nordisk: semaglutide; AstraZeneca: Zirconium cyclosilicate, dapagliflozin; Amgen: evolocumab; Boehringer Ingelheimer: empagliflozin; Lilly: tirzepatide, atorvastatin viatris; Daichii Sankyo: bempedoic acid/Edoxaban; Mantecorp: rosuvastatin. Other relationships Funding of continuing medical education activities, including travel, accommodation and registration in conferences and courses, from the brazilian or international pharmaceutical, orthosis, prosthesis, equipment and implants industry: - Bayer: finerinone; Novo Nordisk: Semaglutide; AstraZeneca: Zirconium cyclosilicate, dapagliflozin; Amgen: evolocumab; Boehringer Ingelheimer: empagliflozin; Lilly: tirzepatide, atorvastatin viatris; Daichii Sankyo: bempedoic acid/edoxaban.
José Maria Soares Júnior	Financial declaration A - Economically relevant payments of any kind made to (i) you, (ii) your spouse/partner or any other person living with you, (iii) any legal person in which any of these is either a direct or indirect controlling owner, business partner, shareholder or participant; any payments received for lectures, lessons, training instruction, compensation, fees paid for participation in advisory boards, investigative boards or other committees, etc. from the brazilian or international pharmaceutical, orthosis, prosthesis, equipment and implants industry: Pfizer: Abrysvo; Libbs: Zaila.
Larissa de Almeida Dourado	Other relationships Funding of continuing medical education activities, including travel, accommodation and registration in conferences and courses, from the brazilian or international pharmaceutical, orthosis, prosthesis, equipment and implants industry: - EMS: Xakilis; Novartis: Sybrava.
Larissa Neto Espíndola Macedo	Financial declaration A - Economically relevant payments of any kind made to (i) you, (ii) your spouse/partner or any other person living with you, (iii) any legal person in which any of these is either a direct or indirect controlling owner, business partner, shareholder or participant; any payments received for lectures, lessons, training instruction, compensation, fees paid for participation in advisory boards, investigative boards or other committees, etc. from the brazilian or international pharmaceutical, orthosis, prosthesis, equipment and implants industry: Servier: Trimetazidine.
Lidia Zytynski Moura	Financial declaration A - Economically relevant payments of any kind made to (i) you, (ii) your spouse/partner or any other person living with you, (iii) any legal person in which any of these is either a direct or indirect controlling owner, business partner, shareholder or participant; any payments received for lectures, lessons, training instruction, compensation, fees paid for participation in advisory boards, investigative boards or other committees, etc. from the brazilian or international pharmaceutical, orthosis, prosthesis, equipment and implants industry: Bayer, Merck, Novartis, Novo Nordisk, Lilly, Viatris. Other relationships Funding of continuing medical education activities, including travel, accommodation and registration in conferences and courses, from the brazilian or international pharmaceutical, orthosis, prosthesis, equipment and implants industry: - Novo Nordisk, Astra.
Lucelia Batista Neves Cunha Magalhães	Nothing to be declared
Lucia Helena Simões da Costa Paiva	Financial declaration A - Economically relevant payments of any kind made to (i) you, (ii) your spouse/partner or any other person living with you, (iii) any legal person in which any of these is either a direct or indirect controlling owner, business partner, shareholder or participant; any payments received for lectures, lessons, training instruction, compensation, fees paid for participation in advisory boards, investigative boards or other committees, etc. from the brazilian or international pharmaceutical, orthosis, prosthesis, equipment and implants industry: BAYER: diu mirena; Astellas Fezolinetanto; Theramex Systen e Estreva Gel, Besisn Vagifem. Other relationships Funding of continuing medical education activities, including travel, accommodation and registration in conferences and courses, from the brazilian or international pharmaceutical, orthosis, prosthesis, equipment and implants industry: - Astellas; International Menopaus Society Congress 2024.
Luciano de Melo Pompei	Financial declaration A - Economically relevant payments of any kind made to (i) you, (ii) your spouse/partner or any other person living with you, (iii) any legal person in which any of these is either a direct or indirect controlling owner, business partner, shareholder or participant; any payments received for lectures, lessons, training instruction, compensation, fees paid for participation in advisory boards, investigative boards or other committees, etc. from the brazilian or international pharmaceutical, orthosis, prosthesis, equipment and implants industry: Abbott, Aché, Astellas, Bayer, Besins, Biolab, Mantecorp, Libbs, Theramex. Other relationships Funding of continuing medical education activities, including travel, accommodation and registration in conferences and courses, from the brazilian or international pharmaceutical, orthosis, prosthesis, equipment and implants industry: - Besins.
Luiz Guilherme Passaglia	Financial declaration A - Economically relevant payments of any kind made to (i) you, (ii) your spouse/partner or any other person living with you, (iii) any legal person in which any of these is either a direct or indirect controlling owner, business partner, shareholder or participant; any payments received for lectures, lessons, training instruction, compensation, fees paid for participation in advisory boards, investigative boards or other committees, etc. from the brazilian or international pharmaceutical, orthosis, prosthesis, equipment and implants industry: DASA Medical Advisory Board in Minas Gerais. Other relationships Participation in government-related regulatory authorities or advocacy authorities in cardiology: - Member of the Cardiology Committee of CRM MG and member of the Municipal Cardiology Commission of the Municipal Health Department of BH.
Marcelo Heitor Vieira Assad	Financial declaration A - Economically relevant payments of any kind made to (i) you, (ii) your spouse/partner or any other person living with you, (iii) any legal person in which any of these is either a direct or indirect controlling owner, business partner, shareholder or participant; any payments received for lectures, lessons, training instruction, compensation, fees paid for participation in advisory boards, investigative boards or other committees, etc. from the brazilian or international pharmaceutical, orthosis, prosthesis, equipment and implants industry: AstraZeneca: Forxiga; BAYER: Firialta; Biolab: Repatha; Boerhringer Ingelheim: Glyxambi; Daiichy Sankyo: Benicar, Nustendi; EMS: Bramicar; GSK: Shingrix; Libbs: Stanglit; Lilly: Mounjaro; Novo Nordisk: Wegovy and Wegov: Rybelusus and Ozempic; Novartis: Sybrava; Pfizer: Prevenar 20; Viatris: Lipitor, Inspra. B - Financiamento de pesquisas sob sua responsabilidade direta/pessoal (direcionado ao departamento ou instituição) provenientes da indústria farmacêutica, de órteses, próteses, equipamentos e implantes, brasileiras ou estrangeiras: - AMGEN: Olpasirana. Other relationships Funding of continuing medical education activities, including travel, accommodation and registration in conferences and courses, from the brazilian or international pharmaceutical, orthosis, prosthesis, equipment and implants industry: - Bayer: Firialta; Daiichi Sankyo: Benicar; Novo Nordisk: Wegovy.
Marcio Alexandre Hipólito Rodrigues	Financial declaration B - Research funding under your direct/personal responsibility (directed to the department or institution) from the brazilian or international pharmaceutical, orthosis, prosthesis, equipment and implants industry: Besins, Theramex.
Maria Alayde Mendonça Rivera	Nothing to be declared
Maria Antonieta Albanez Albuquerque de Medeiros Lopes	Financial declaration A - Economically relevant payments of any kind made to (i) you, (ii) your spouse/partner or any other person living with you, (iii) any legal person in which any of these is either a direct or indirect controlling owner, business partner, shareholder or participant; any payments received for lectures, lessons, training instruction, compensation, fees paid for participation in advisory boards, investigative boards or other committees, etc. from the brazilian or international pharmaceutical, orthosis, prosthesis, equipment and implants industry: Boston, Medtronic, Daiichi Sankyo. Other relationships Funding of continuing medical education activities, including travel, accommodation and registration in conferences and courses, from the brazilian or international pharmaceutical, orthosis, prosthesis, equipment and implants industry: - Boston.
Maria Celeste Osorio Wender	Nothing to be declared
Maria Cristina Costa de Almeida	Nothing to be declared
Maria Cristina de Oliveira Izar	Declaração financeira A - Pagamento de qualquer espécie e desde que economicamente apreciáveis, feitos a (i) você, (ii) ao seu cônjuge/ companheiro ou a qualquer outro membro que resida com você, (iii) a qualquer pessoa jurídica em que qualquer destes seja controlador, sócio, acionista ou participante, de forma direta ou indireta, recebimento por palestras, aulas, atuação como proctor de treinamentos, remunerações, honorários pagos por participações em conselhos consultivos, de investigadores, ou outros comitês, etc. Provenientes da indústria farmacêutica, de órteses, próteses, equipamentos e implantes, brasileiras ou estrangeiras: - AstraZeneca: Forxiga; BAYER: Firialta; Biolab: Repatha; Boerhringer Ingelheim: Glyxambi; Daiichy Sankyo: Benicar, Nustendi; EMS: Bramicar; GSK: Shingrix; Libbs: Stanglit; Lilly: Mounjaro; Novo Nordisk: Wegovy e Wegov: Rybelusus e Ozempic; Novartis: Sybrava; Pfizer: Prevenar 20; Viatris: Lipitor, Inspra. B - Financiamento de pesquisas sob sua responsabilidade direta/pessoal (direcionado ao departamento ou instituição) provenientes da indústria farmacêutica, de órteses, próteses, equipamentos e implantes, brasileiras ou estrangeiras: - AMGEN: Olpasirana. Outros relacionamentos Financiamento de atividades de educação médica continuada, incluindo viagens, hospedagens e inscrições para congressos e cursos, provenientes da indústria farmacêutica, de órteses, próteses, equipamentos e implantes, brasileiras ou estrangeiras: - Bayer: Firialta; Daiichi Sankyo: Benicar; Novo Nordisk: Wegovy.
Maria Elizabeth Navegantes Caetano Costa	Financial declaration A - Economically relevant payments of any kind made to (i) you, (ii) your spouse/partner or any other person living with you, (iii) any legal person in which any of these is either a direct or indirect controlling owner, business partner, shareholder or participant; any payments received for lectures, lessons, training instruction, compensation, fees paid for participation in advisory boards, investigative boards or other committees, etc. from the brazilian or international pharmaceutical, orthosis, prosthesis, equipment and implants industry: Libbs: Plenance Enze; Servier: Vastarel. Other relationships Funding of continuing medical education activities, including travel, accommodation and registration in conferences and courses, from the brazilian or international pharmaceutical, orthosis, prosthesis, equipment and implants industry: - Libbs; Servier: Participation in conference.
Maria Sanali Moura de Oliveira Paiva	Other relationships Any economically relevant equity interest in companies in the healthcare or education industry or in any companies competing with or supplying to SBC: - Cardiology.
Marildes Luiza de Castro	Financial declaration A - Economically relevant payments of any kind made to (i) you, (ii) your spouse/partner or any other person living with you, (iii) any legal person in which any of these is either a direct or indirect controlling owner, business partner, shareholder or participant; any payments received for lectures, lessons, training instruction, compensation, fees paid for participation in advisory boards, investigative boards or other committees, etc. from the brazilian or international pharmaceutical, orthosis, prosthesis, equipment and implants industry: Novartis: Sacubitril/Valsartana; Pfizer: Patisiran; Merck: Vericiquat; Amgen.
Milena dos Santos Barros Campos	Nothing to be declared
Olga Ferreira de Souza	Nothing to be declared
Orlando Otávio de Medeiros	Nothing to be declared
Rafaela Andrade Penalva Freitas	Financial declaration A - Economically relevant payments of any kind made to (i) you, (ii) your spouse/partner or any other person living with you, (iii) any legal person in which any of these is either a direct or indirect controlling owner, business partner, shareholder or participant; any payments received for lectures, lessons, training instruction, compensation, fees paid for participation in advisory boards, investigative boards or other committees, etc. from the brazilian or international pharmaceutical, orthosis, prosthesis, equipment and implants industry: AstraZeneca: diabetes; NovoNordisk: diabetes, obesity; Daiichi Sankyo Brasil: dyslipidemia; Servier: chronic coronary disease; Libbs: diabetes; Mantecorp: diabetes. Other relationships Funding of continuing medical education activities, including travel, accommodation and registration in conferences and courses, from the brazilian or international pharmaceutical, orthosis, prosthesis, equipment and implants industry: - AstraZeneca: diabetes; Novartis: dyslipidemia; Novo Nordisk: diabetes, obesity.
Regina Coeli Marques de Carvalho	Nothing to be declared
Sheyla Cristina Tonheiro Ferro da Silva	Other relationships Funding of continuing medical education activities, including travel, accommodation and registration in conferences and courses, from the brazilian or international pharmaceutical, orthosis, prosthesis, equipment and implants industry: - Astra Zeneca: FORXIGA, lokelma; Novartis: entresto, Sybrava; Novo Nordisk: rybelsus, wygovy; Lilly: Jardiance, Mounjaro.
Thais de Carvalho Vieira Rodrigues	Nothing to be declared
Viviana de Mello Guzzo Lemke	Nothing to be declared
Walkiria Samuel Avila	Nothing to be declared
Wellington Santana da Silva Júnior	Financial declaration A - Economically relevant payments of any kind made to (i) you, (ii) your spouse/partner or any other person living with you, (iii) any legal person in which any of these is either a direct or indirect controlling owner, business partner, shareholder or participant; any payments received for lectures, lessons, training instruction, compensation, fees paid for participation in advisory boards, investigative boards or other committees, etc. from the brazilian or international pharmaceutical, orthosis, prosthesis, equipment and implants industry: Abbott, AstraZeneca, Brace Pharma, Libbs, Lilly, Novartis, Novo Nordisk. Other relationships Funding of continuing medical education activities, including travel, accommodation and registration in conferences and courses, from the brazilian or international pharmaceutical, orthosis, prosthesis, equipment and implants industry: - AstraZeneca, Novo Nordisk.
Willyan Issamu Nazima	Nothing to be declared

## Table of contents

**1. Introduction** 13
**2. Highlights** 18
**3. Inflammation and Implications for the Cardiovascular System** 23
**3.1. Inflammation and Atherosclerosis** 23
**3.2. Inflammation and Heart Failure in Women** 25
**3.3. Hormonal Aspects in Inflammation** 25
**3.4. Inflammation in Cardio-Oncology** 25
**3.5. Inflammation in Pregnancy and Preeclampsia** 25
**4. Implications of Sex Steroids for Cardiometabolic Health** 27
**4.1. Origin and Metabolism of Sex Steroids** 27
**4.2. Chemical Classification of Estrogens** 28
**4.2.1. Natural Estrogens** 28
**4.2.2. Synthetic Estrogens** 28
**4.3. Chemical Classification of Progestagens** 28
**4.3.1. Natural Progesterone** 28
**4.3.2. Progestagens** 28
**4.4. Common Indications of Therapies with Estrogens, Progestagens, and Testosterone in Women** 29
**4.4.1. Polycystic Ovary Syndrome** 29
**4.4.2. Hormonal contraception** 29
**4.4.3. Menopausal Hormone Therapy: Regimens, Doses, and Routes, Focused on Cardiovascular Health** 30
**4.5. Risks: Metabolic Effects, Thromboembolism, and Breast Cancer** 32
**4.5.1. Metabolic Effects** 32
**4.5.2. Thromboembolism** 32
**4.5.3. Breast Cancer** 32
**5. Indicators of Cardiometabolic Health** 33
  5.1. Assessment of Reproductive Risk Factors with Implications for Cardiometabolic Health 33
**5.1.1. Puberty and Pregestational Period** 33
***5.1.1.1. Age at Menarche*** 33
***5.1.1.2. Menstrual Cycle Characteristics*** 33
**5.1.2. Pregnancy and Postpartum Period** 33
***5.1.2.1. Weight changes in pregnancy*** 33
***5.1.2.2. Gestational dysglycemia*** 33
***5.1.2.3. Gestational Dyslipidemia*** 33
***5.1.2.4. Postpartum Behavior*** 34
**5.1.3. Menopause Transition** 34
***5.1.3.1. Age at menopause*** 35
***5.1.3.2. Vasomotor Symptoms*** 35
***5.1.3.3. Menopausal Hormone Therapy*** 35
**5.1.4. Postmenopausal Metabolic Health** 35
***5.1.4.1. Menopausal metabolic changes*** 35
***5.1.4.2. Diabetes mellitus*** 35
***5.1.4.3. Dyslipidemia*** 35
**5.2. Anthropometric Indicators** 36
**5.2.1. Body Mass Index** 36
**5.2.2. Waist circumference – Brazilian population** 36
**5.2.3. Waist-To-Hip Ratio** 36
**5.2.4. Body Fat Percentage** 36
**5.2.5. Waist-To-Height Ratio** 36
**5.3. Biomarkers** 36
**5.3.1. Lipid Profile (Total Cholesterol, LDL-c, HDL, Triglycerides)** 36
**5.3.2. Glycemia and Glycated Hemoglobin** 36
**5.3.3. C-reactive Protein** 37
**5.3.4. Fibrinogen** 37
**5.3.5. Homocysteine** 37
**5.3.6. Adipokines** 37
**6. Cardiometabolism in Childhood and Adolescence** 37
**6.1. Definitions and Diagnostic Criteria** 37
**6.1.1. Pathophysiology** 37
**6.1.2. Early Menarche, Late Menarche, and Cardiometabolic Risk** 37
**6.2. Menstrual irregularities and cardiometabolic risk** 39
**6.2.1. Pathophysiological Mechanisms** 39
**6.2.2. Clinical Assessment and Biomarkers** 40
**6.2.3. Therapeutic Interventions** 40
**6.3. Obesity, Eating Disorders, and Cardiovascular Risk: Long-Term Impacts** 40
**6.3.1. Eating Disorders and Obesity** 40
**6.3.2. Hereditary Factors and Prognosis of Eating Disorders** 40
**6.3.3. Future Consequences of Eating Disorders** 40
**6.3.4. Childhood Obesity and Cardiovascular Outcomes** 41
**6.3.5. Eating Disorders and Cardiovascular Risk** 41
**7. Cardiometabolic Continuum and Reproductive Age** 41
**7.1. Polycystic Ovary Syndrome** 41
**7.2. Infertility and its Treatment** 42
**7.3. Weight changes in pregnancy** 43
**7.3.1. Maternal Impact of Excessive Gestational Weight Gain** 44
**7.3.2. Fetal Impact of Excessive Gestational Weight Gain** 44
**7.4. Gestational Dysglycemia** 44
**7.5. Gestational dyslipidemia** 45
**7.5.1. Impact of Dyslipidemia in Pregnancy** 46
**7.5.2. Impact of Pregnancy on Patients with Familial Hypercholesterolemia** 46
**7.6. Endometriosis and Cardiovascular Risk** 46
**7.6.1. Risk factors** 46
**7.7. Psoriasis** 46
**7.8. Hypertensive Disorders of Pregnancy and Endothelial Dysfunction after Menopause** 49
**7.9. Metabolic Changes in Postpartum** 49
**7.9.1. Cardiometabolic Risk Factors in Postpartum** 49
**7.9.2. Impact of gestational diabetes and dyslipidemia in postpartum** 49
**7.9.3. Prolactin and Metabolism in Postpartum** 49
**8. Cardiometabolic Health in Menopause Transition, Menopause and Postmenopause** 50
**8.1. Severity of Symptoms and their Implications for Cardiovascular Health** 50
**8.2. Cardiovascular Risk Factors and Menopause** 50
**8.3. Menopausal Hormone Therapy, Hormone Implants, and Cardiometabolic Impact** 52
**8.4. Stratification of Cardiometabolic Risk in Menopause** 52
**9. Cardiometabolic Disorders in Women** 53
**9.1. Obesity and Metabolic Syndrome** 53
**9.2. Type 2 Diabetes Mellitus** 54
**9.3. Metabolic Dysfunction-Associated Steatotic Liver Disease** 55
**9.4. Chronic Kidney Disease** 56
**10. Strategies for Addressing Cardiometabolic Disorders in Women** 57
**10.1. Nonpharmacological Measures** 57
**10.2. Nutritional Interventions** 57
**10.3. Physical Activity** 57
**10.4. Psychosocial Interventions** 58
**10.5. Tobacco Use and Alcohol Consumption** 58
**10.6. Specific Clinical Conditions** 58
**10.7. Pharmacological Strategies** 58
**10.7.1. Systemic Arterial Hypertension Treatment** 58
**10.7.2. Management of Dyslipidemias** 59
**10.7.3. Oral Lipid-Lowering Drugs** 59
**10.7.4. Diabetes Mellitus Control** 59
**10.7.5. Management of Obesity** 60
**10.7.6. Management of Metabolic Dysfunction-Associated Steatotic Liver Disease** 62
**10.7.7. Management of Chronic Kidney Disease** 62
**10.7.8. Role of GLP-1 Analogues in Women’s Cardiometabolic Treatment** 63
**10.7.9. Specificities in Women** 63
**10.7.10. Semaglutide** 63
**10.7.11. Tirzepatide** 63
**10.7.12. Future Perspectives** 64
**10.8. Specific pharmacological considerations** 64
**10.8.1. Drug Interactions** 64
**10.8.2. Importance of Weight Control** 64
**10.8.3. Individualized Approach** 64
**10.9. Surgical Treatment** 64
**10.9.1. Bariatric Surgery** 64
**11. Recommendations for the Management of Cardiometabolic Disorders in Women** 64
Acknowledgments 67
Supplement 71
References 82

**Figure:** Female life cycle and cardiometabolic continuum: risk factors and strategies. Spiral representing a woman's life cycle (phases) and its association with the cardiometabolic continuum. Reproductive events are written in blue, marking each phase, such as age of menarche (early or late), POS, GD, etc. Major cardiometabolic risk factors, such as insulin resistance, dyslipidemia, central obesity, and chronic inflammation, are represented around the spiral, where major intervention points throughout life, such as early screening, pharmacological therapies, lifestyle changes, and multidisciplinary approach, stand out, aimed at primary and secondary prevention of women's cardiovascular diseases. GD: gestational diabetes; LSC: lifestyle changes; POF: premature ovarian failure; POS: polycystic ovary syndrome; VMS: vasomotor symptoms.

## 1. Introduction

Cardiometabolic health can be characterized by ideal levels of serum glucose and lipids and of blood pressure (BP) in association with low adiposity and cardiovascular risk (CVR). Usually, cardiometabolic health is the absence of metabolic dysfunction characteristic of some diseases, such as cardiovascular diseases (CVD), type 2 diabetes *mellitus* (T2DM), and metabolic syndrome (MS). Poor metabolic health is responsible for a substantial population burden of disability, diseases, as well as cardiovascular, neoplastic, and all-cause deaths. Different characteristics related to reproduction have been increasingly associated with metabolic diseases based on life course epidemiology, which postulates that biological, behavioral, and social factors during sensitive stages of life, mediated by hormonal fluctuations, act independently, cumulatively and interactively to influence the posterior risk for health and disease^1 , 2^( Figure 1.1 ).

**Figure 1.1:** Spectrum of cardiometabolic health. BMI: body mass index; WHR: waist to hip ratio; WHtR: waist to height ratio.

The prevalence of cardiometabolic disorders is increasing worldwide among women and men and has been associated with higher rates of obesity and its risk factors (RFs), such as hypertension and T2DM. In addition, there is increasing evidence suggesting that sexual hormones, and sex- and gender-specific molecular mechanisms can influence the metabolism of glucose and lipids, thus impacting on cardiometabolic RFs. Moreover, there is an emerging predominance of common types of cardiometabolic disorders, such as heart failure (HF), atrial fibrillation, and ischemic heart disease (IHD), that differ between females and males. Significant sex-specific variations have been reported in risk profiles, with an especial emphasis on women, whose risk scores are inadequate to predict both atherothrombotic diseases and their related outcomes, such as cardiovascular death, myocardial infarction (MI), stroke, HF, ventricular and supraventricular arrhythmias, as well as need for revascularization and hospitalization.^2^

The ranking of CVD deaths and DALYs (disability-adjusted life years, a measure that represents the burden of disease on a population) rates per 100,000 inhabitants, attributed to RFs in women and men in Brazil in 2021, shows that metabolic RFs represent the first five RFs for women's death and the first four RFs for women's DALYs. It is worth noting that the increase in systolic BP, low-density lipoprotein cholesterol (LDL-c), body mass, and serum glucose, in that order, is frequently associated with CVD death and burden in the Brazilian female population. In addition, kidney dysfunction is an important metabolic RF for women and men. It is worth noting the increase in body mass and serum glucose in the past 21 years, representing a significant increase of obesity and diabetes *mellitus* (DM) in women and men.^3^( Figures 1.2 and 1.3 )

**Figure 1.2:** Ranking of mortality rates per 100,000 inhabitants due to cardiovascular disease attributed to risk factors, in women and men, in Brazil, in 2021.^3^

**Figure 1.3:** Ranking of DALYs rates per 100,000 inhabitants due to cardiovascular disease attributed to risk factors, in women and men, in Brazil, in 2021.^3^

Recent evidence suggests that female reproductive characteristics can be related to RFs that contribute to later metabolic dysfunction that culminates in CVD in menopause. These reproductive characteristics are as follows: age of menarche, menstrual irregularity of endocrine origin, development of polycystic ovary syndrome (POS), excessive weight gain in pregnancy, gestational dysglycemia and dyslipidemia, hypertensive disorders of pregnancy, severity and timing of menopausal symptoms, and effect of hypoestrogenism on the cardiovascular system. Those RFs can be markers of future dysfunction or be explained by shared underlying etiologies that promote disease in the long run. Identifying potentially modifiable characteristics has a significant influence in strategies to implement a healthy lifestyle, as well as drug and surgical therapies that can relieve metabolic burden in the long run.^4 - 6^

The cardiometabolic *continuum* , sequence of cardiovascular events resulting from gene-environmental interactions, influences of unhealthy lifestyles, and metabolic diseases, such as DM and systemic arterial hypertension (SAH), occurs predominantly over the course of a woman's life ( Figure 1.4 ). In a recent study, the cardiometabolic *continuum* was analyzed to assess differences between sexes and populations in two distinct cohorts: the UK Biobank (17,700 participants) and the Brazilian Longitudinal Study of Adult Health (in Portuguese, ELSA-Brasil - *Estudo Longitudinal de Saúde do Adulto* ) with 7,162 participants. The authors have studied the cardiometabolic *continuum* using machine learning and identified five patterns. They reported female disadvantage regarding the time of appearance of the cardiometabolic *continuum* . In the UK Biobank cohort, when SAH was the first disease of the cardiometabolic *continuum* and diagnosed in isolation, it occurred faster in women. In the ELSA-Brasil cohort, not only DM was more frequently the first disease in the female cardiometabolic *continuum* but also diagnosed faster when followed by SAH. In addition, women had a greater incidence of isolated SAH and DM, and a smaller percentage of them was classified as healthy. The authors have emphasized the unequal access to proper treatment and diagnosis of the female cardiometabolic *continuum* and stressed the need for sex-differentiated health policies in Brazil to reduce inequities.^7^

**Figure 1.4:** Cardiometabolic continuum in the different phases of a woman's life.

The cardiovascular system, kidneys and liver share RFs, such as dyslipidemia, hypertension, tobacco use, DM, and central/truncal obesity. Shared metabolic and functional disorders result in damage to those organs via overlapping pathophysiological pathways, to which hormonal influences, derived from the different cycles of a woman's life, are added. The increase in metabolic RFs over the years supports the need to improve the identification and treatment of women's cardiometabolic disorders, mainly because they are underdiagnosed and undertreated. This is compounded by the women's small participation in clinical trials that guide the currently available therapeutic strategies, which include lifestyle changes, drug therapy, and surgery. Moreover, the inflammatory nature, notably higher in women with cardiometabolic disorders, as well as the new pharmacological and surgical therapeutic options that provide management based on mechanisms that can reach multiple pathophysiological pathways influenced by hormones, require further studies in the female sex. Specific social programs to encourage healthy diets and physical activity, as well as better access to public health policies aimed at women with cardiometabolic disorders, are required.^4 , 8^

This *Position Statement on Cardiometabolic Health Over the Course of a Woman's Life* is a joint effort of the Women's Cardiology Department of the Brazilian Society of Cardiology (DCM/SBC), Brazilian Federation of the Societies of Gynecology and Obstetrics (FEBRASGO), and Brazilian Society of Endocrinology and Metabolism Study (SBEM) to close the knowledge gap on women's cardiometabolic disorders.

In what follows, we present the highlights of this position statement according to their respective chapters.

## 2. Highlights

### Chapter 3

- In women, menopause and pregnancy complications (mainly preeclampsia/eclampsia) activate inflammation and accelerate coronary atherosclerosis, causing vascular stiffness, endothelial dysfunction, and microvascular ischemia;
- Biological variations between women and men result from differences in gene expression of sex chromosomes modulated by hormonal and environmental influences, which result in cardiovascular conditions associated with autonomic regulation and vascular and cardiac remodeling;
- Women with menstrual irregularities of endocrine origin frequently exhibit a pro-inflammatory status with elevation of inflammatory markers that intermediate atherosclerotic genesis;
- The increased prevalence of risk factors in women, such as obesity, diabetes, and hypertension, associated with the effects of menopause, seems to explain the higher prevalence of heart failure with preserved ejection fraction;
- The association of chronic inflammation and adiposity in heart failure with preserved ejection fraction seems to be related to pericardial and epicardial adipokines, leading to microcirculatory inflammation, cardiac fibrosis, and physiological diastolic filling changes;
- Postmenopausal hypoestrogenism promotes metabolic changes, such as increased central adiposity, worse glucose metabolism, and increased levels of total cholesterol, LDL-c, triglycerides, apolipoproteins, and lipoprotein a.

### Chapter 4

- Sex steroid hormones bind to hormonal receptors in several tissues and have multiple biological effects;
- The different sex steroid hormones are frequently indicated to treat conditions, such as polycystic ovary syndrome, as well as for contraception and menopausal hormone therapy, which can have implications for cardiometabolic risk;
- Combined oral contraceptives are not recommended for women with a history of tobacco use over the age of 35 years, cardiovascular diseases, venous thromboembolism, diabetes with vascular complication, migraine with neurological signs, severe liver disease, liver tumors, breast cancer, and systemic lupus erythematosus;
- The cardiovascular effects of menopausal hormone therapy are influenced by hormone type and dose used, administration route, and timing of therapy initiation in relation to menopause onset ("window of opportunity"). More benefits and fewer adverse effects are believed to occur when therapy is initiated within 10 years from menopause onset;
- The factors determining the type and dose of menopausal hormone therapy are as follows: patient's preference, uterus presence/absence, need for contraception, intensity of symptoms, and associated comorbidities.

### Chapter 5

- Menstrual cycle assessment can be used as additional data in the investigation of women's cardiometabolic health status;
- Age of menarche (early or late), menstrual cycle irregularities, and polycystic ovary syndrome are associated with a higher future risk of cardiovascular diseases;
- In pregnancy, abnormal weight gain and changes in lipid profile and glycemia can be associated with adverse pregnancy and postpartum maternal and infant outcomes;
- Hyperlipidemia during pregnancy is associated with preeclampsia, preterm delivery, and gestational diabetes, and children are likely to develop fatty streaks and experience increased risk for progressive atherosclerosis;
- Pregnancy is a condition of insulin resistance per se (40-50% increase), which can be aggravated in women with pre-pregnancy obesity, who are also at higher risk of developing gestational diabetes;
- Women with glucose intolerance during pregnancy are at risk for adverse gestational outcomes, even when they do not develop diabetes;
- In early menopause and in the presence of moderate to severe vasomotor symptoms, there is a higher likelihood of a more atherogenic lipid profile, insulin resistance, higher elevation of blood pressure, higher risk of metabolic syndrome, and worsening of endothelial function and inflammatory markers;
- Anthropometric indicators, such as biomarkers (lipid and glycemic profiles, C-reactive protein, fibrinogen, homocysteine and adipokine), provide information on body fat composition and distribution, reflecting the cardiometabolic risk.

### Chapter 6

- Early or late menarche and menstrual irregularities of endocrine origin are important markers of metabolic and cardiovascular risk, whose early identification guides preventive measures, aimed at reducing metabolic risk and burden of cardiovascular diseases of the female population;
- Obesity and eating disorders in childhood and adolescence involve genetic, metabolic, behavioral, and environmental aspects and significantly affect cardiovascular and mental health throughout adult life;
- Early identification and continuous monitoring of obesity, in addition to implementation of therapeutic strategies since childhood are essential to interrupt the vicious cycle of obesity, eating disorders and their complications, minimizing the adverse outcomes in the long run.

### Chapter 7

- Women with polycystic ovary syndrome can have insulin resistance and dyslipidemia, which contribute to the development of cardiovascular diseases because of the excess of androgens, weight gain with visceral fat accumulation, and chronic inflammatory process;
- Endometriosis is associated with chronic inflammatory process with increased oxidative stress and elevation of cardiovascular risk factors, as well as an increased risk for venous thromboembolism, ischemic heart disease, heart failure, and stroke;
- Treatment for infertility can have effects on cardiometabolism, such as ovarian hyperstimulation syndrome, with an increased risk for thromboembolic events, preeclampsia, gestational diabetes, and hypertensive disorders of pregnancy, increasing cardiovascular events in the long run;
- Psoriasis is a systemic chronic inflammatory disease associated with obesity, metabolic syndrome, cardio- and cerebrovascular diseases, cardiac arrhythmias, sleep apnea, and others;
- Preeclampsia is considered a risk marker of cardiovascular diseases throughout life, which seems to increase after menopause, with worsening of the cardiometabolic profile;
- Postpartum excessive weight retention is associated with a higher risk of dyslipidemia and insulin resistance;
- Women with history of gestational diabetes are more likely to experience metabolic disorders in the postpartum period and throughout life, independently of other traditional cardiovascular risk factors.

### Chapter 8

- The decrease in endogenous estradiol levels during menopause transition is associated with an increased risk of cardiometabolic disorders, such as abdominal adiposity, dyslipidemia, type 2 diabetes *mellitus* , and systemic arterial hypertension, which are related to renin-angiotensin-aldosterone system dysfunction, sympathetic activation, endothelial dysfunction, inflammation, and higher sodium sensitivity;
- Women with premature ovarian failure and those with early menopause are at a higher risk of non-fatal cardiovascular events before the age of 60 years;
- Menopausal hormone therapy is used to relieve menopause symptoms and not indicated for primary or secondary prevention of cardiometabolic disorders;
- Testosterone replacement therapy is not indicated to improve cardiometabolic or musculoskeletal health, vasomotor symptoms, or mood changes;
- Hormone implants for menopause, mainly of testosterone, are not recommended because their cardiometabolic effects and risks for breast and endometrial cancers are not known.

### Chapter 9

- Weight gain during pregnancy and its maintenance in the postpartum and polycystic ovary syndrome are risk factors for obesity and for the increase in insulin resistance and in other metabolic syndrome components;
- Women with type 2 diabetes *mellitus* are at higher risk for cardiovascular complications as compared to men;
- Menopause, history of early menarche, and polycystic ovary syndrome are associated with an increase in women's susceptibility to metabolic dysfunction-associated steatotic liver disease, whose mortality rate due to cirrhosis is higher than that of men. It is the major cause of liver transplantation in women without hepatocellular carcinoma;
- Regarding chronic kidney disease, women more often require dialysis and show faster glomerular filtration rate loss as compared to men, especially the elderly and postmenopausal ones.

### Chapter 10

- The increasing prevalence of cardiometabolic disorders in women represents one of the major challenges in public health because of the strong relation between obesity and cardiovascular diseases;
- Nutritional education is crucial for preventing cardiometabolic disorders, and the association of nutritional plans with regular physical activity practice enhances beneficial effects, favoring cardiovascular function and decreasing morbidity and mortality among women;
- Women who participate in integrated psychological support programs significantly improve their lifestyle and clinical outcomes, such as weight reduction, as well as glycemic and lipid profile control;
- Tobacco use is a major chronic inflammatory factor, which is particularly compounded by menopause;
- Multidisciplinary approach has shown to be effective in generating sustained lifestyle changes, reducing morbidity and mortality from cardiometabolic disorders;
- Hypertension is the most prevalent risk factor for cardiovascular disease in all phases of a woman's life and its occurrence increases progressively with age. The choice and conduction of pharmacological treatment strategies should consider women's reproductive cycle phases, including peri- and postmenopause. The use of angiotensin-converting-enzyme inhibitors and angiotensin receptor blockers in women of reproductive age requires caution because of teratogenic risk;
- Menopause is associated with significant elevations in total cholesterol, LDL-c, apolipoprotein B, triglycerides, and lipoprotein a, in addition to a possible reduction in the antiatherogenic protective effect of HDL-c. Although HDL-c levels above 50 mg/dL are desired in women, LDL-c reduction remains the therapeutic priority;
- Women have a different progression of prediabetes to type 2 diabetes *mellitus* , frequently associated with higher obesity indices and increased risk of metabolic complications. Women can have higher risk of hypoglycemia with sulfonylureas and different response to glitazones, related to kidney function and body composition. Regarding treatment with GLP-1 analogues, women on oral contraception require especial attention;
- There are several pharmacological treatments for obesity with different effects on body weight, but only more recent treatments are associated with a reduction in cardiovascular and metabolic outcomes;
- The treatment of metabolic dysfunction-associated steatotic liver disease consists in lifestyle changes with focus on reducing at least 5% of body weight, because weight loss is the most effective measure to improve that disease's histological findings;
- Pregnancy is one of the major causes of acute kidney injury in women of reproductive age and, along with preeclampsia, can lead to chronic kidney disease. Chronic kidney disease has a negative effect on pregnancy, even at the very beginning, and the risks increase with the progression of pregnancy and the concomitant presence of type 2 diabetes *mellitus* ;
- To improve cardiometabolic parameters (lipid and glycemic profiles) and inflammatory markers, bariatric surgery is recommended for women with body mass index ≥ 35 kg/m² and history of diabetes, metabolic dysfunction-associated steatotic liver disease, or at high risk for cardiovascular events, as well as for those with body mass index ≥ 40 kg/m², independently of comorbidities.

### Chapter 11

### Chapter 11

### Chapter 11

## 3. Inflammation and Implications for the Cardiovascular System

Inflammation is a process of defense activated by infectious agents, autoimmune and inflammatory diseases, influenced by traditional RFs, such as DM, hypertension, dyslipidemia, obesity, and tobacco use. In association with these metabolic cardiorenal factors, there are physiological changes of ageing, called immunosenescence or immune-aging.

In women, menopause and pregnancy, in particular preeclampsia/eclampsia, activate inflammation and accelerate coronary atherosclerosis, promoting vascular stiffness, endothelial dysfunction, and microvascular ischemia. Some relevant examples of the role played by inflammation in women's CVDs are as follows: heart failure with preserved ejection fraction (HFpEF) with its inflammatory phenotype; and ischemia/myocardial infarction with no obstructive coronary arteries (INOCA/MINOCA).

### 3.1. Inflammation and Atherosclerosis

Cardiovascular diseases, particularly IHD, are the major cause of death in women, mainly between the ages of 45 years and 75 years.^9 , 10^Although sex-related differences in CVR have been well established, they are not completely understood.^11^Thus, better knowledge of the mechanisms that contribute to worsen women's risk profiles is mandatory to reduce morbidity and mortality from those diseases.

Several factors contribute to differences between sexes regarding the diagnosis and treatment of CVDs. It is a consensus that women are under-represented in large clinical trials, as evidenced in the VIGO Study, which has assessed 2349 women and 1152 men with acute myocardial infarction (AMI) under the age of 55 years, in the presence of three or more RFs. That study showed that women are less likely to receive guidance on cardiovascular prevention.^12 , 13^This scenario can jeopardize the ability to accurately assess the efficacy and safety of different therapies in women, thus hindering specific strategies to prevent and treat CVDs.^13^

In addition to traditional RFs, several emerging conditions should be considered in female CVR, such as depression, domestic violence, socioeconomic and cultural profiles, obstetric and gynecologic history, gestational hypertension, gestational diabetes (GD), preterm delivery, premature menopause, POS, breast cancer, etc.^10 , 14^

In IHD, those factors reflect significant differences between sexes regarding pathophysiology, clinical presentation, and outcomes. INOCA is more frequent in women, specifically between the ages of 45 years and 65 years,^15^and so as MINOCA.^16^Although epicardial coronary vasospasm is more common among men, up to 70% of the cases of coronary microcirculation dysfunction occur in women.^17^Spontaneous coronary dissection, although rare, is a cause of acute coronary syndrome in women under the age of 50 years.^18^

The atherosclerotic process has been known for decades and was initially associated with inflammation by Rudolph Virchow, who showed that inflammation is a core element in the formation and progression of atherosclerotic plaque in coronary syndromes, for both chronic obstruction and plaque rupture.^19^

Atherosclerosis begins early with the accumulation of modified lipoproteins on vascular endothelium and worsens with age. In women, in addition to the metabolic effects of menopause, ageing contributes to endothelial dysfunction, mainly by increasing reactive oxygen species (ROS) production associated with senescence. Cytokines and inflammatory cells have pro-atherogenic effects by disrupting the endothelial barrier, reducing vasodilation, inducing the expression of adhesion molecules and chemokines, and facilitating the recruitment of leukocytes to atherosclerotic lesions.^19 - 21^

Biological variations between women and men result from differences in gene expression of sex chromosomes, modulated by hormonal and environmental influences, that result in cardiovascular conditions associated with autonomic regulation, as well as vascular and cardiac remodeling.^18^

In addition, epigenetic factors play a relevant role, especially in women, whose expression is widely modulated by menopause. These changes can influence gene transcription and translation via mechanisms, such as DNA methylation, histone modification, and regulation of non-coding RNAs. A related phenomenon, clonal hematopoiesis of indeterminate potential (CHIP), refers to the presence of somatic mutations in blood cells with no progression to hematological malignancies.^22 , 23^These mutations increase with age, are detected in approximately 10% of individuals older than 70 years, and are associated with the concept of inflamm-aging, a term proposed by Franceschi *et al* . to explain the inflammatory process increase by immunosenescence.^22 , 23^

Thus, biological differences and underlying female-sex-specific pathophysiology of CVD have not been completely elucidated, and further research is required for the development of more effective preventive and therapeutic strategies.

### 3.2. Inflammation and Heart Failure in Women

Local or systemic inflammatory process participates in HF pathogenesis, with cardiac remodeling and fibrosis, in addition to macro- and microcirculation abnormalities. Low-intensity chronic inflammation is associated with different RFs, such as obesity, insulin resistance (IR), T2DM, dyslipidemia, chronic kidney disease (CKD), and ageing, and leads to a reduction in nitric oxide (NO) production, changing the ventricular diastolic physiology, promoting left atrial myopathy.^24 , 25^The increased prevalence of those RFs in women, associated with the effects of menopause, seem to explain the higher prevalence of HFpEF. The association of chronic inflammation and adiposity in HFpEF results from evidence pointing to the role of the release of substances produced by the visceral adipose tissue (adipokines) particularly located around the pericardium and epicardium, leading to microcirculation inflammation, cardiac fibrosis, and physiological diastolic filling changes.^26 , 27^

In women, menopause induces cardiac function changes, resulting from the decrease in estrogen production and consequent reduction in NO production. This hormonal deficiency causes changes in the coronary circulation diastolic function and physiology. Clinically, HFpEF can show different phenotypes, two of which are common in women: the cardiometabolic phenotype, in which obesity and DM are associated with diastolic dysfunction; and atrial fibrillation and myopathy, present in elderly women.^28 , 29^In addition, conditions such as obesity, DM, and hypertensive disorders of pregnancy increase the risk of HFpEF in women, probably because of associated inflammatory mechanisms. Despite the higher prevalence of those conditions in women, they are not included in most clinical trials, which leads to poor knowledge about treatment efficacy in the female sex.^30^

Biological markers of inflammation, such as ultrasensitive C-reactive protein (us-CRP) over 2mg/L, have been proven useful to characterize individuals with HFpEF of cardiometabolic phenotype, usually associated with worse prognosis.^31^

Preeclampsia has been recognized as a condition that leads to changes in immune and systemic inflammation activation, with consequent endothelial and cardiac dysfunction, and it can manifest over the following decades and increase the risk for HF.^32 , 33^

In women, autoimmune diseases, mainly systemic lupus erythematosus, can cause inflammation of the myocardium, pericardium, and valvular endocardium, as well as of the coronary macro- and microcirculation. Thus, pathophysiological abnormalities and clinical aspects of the disease must be understood so that preventive measures can be established and cardiovascular complications be early detected.^34^

The important role of HF has been evidenced in women. Studies, such as the PURSUIT-HFpEF and the analysis from TOPCAT study, have shown that women with HFpEF have more significant diastolic dysfunction and worse clinical outcomes as compared to men. Understanding the sex-specific mechanisms is crucial to advance the management of HFpEF.^30^

The *post-hoc* analysis from TOPCAT study has shown significant differences in the baseline characteristics between women and men with HFpEF.^35^Women (55.5% of the cohort) had fewer cardiovascular comorbidities (previous infarction: 18.7% vs. 30.9% in men, p < 0.001), worse cardiac function (NYHA class III/IV: 38.5% vs. 31.5%, p = 0.003), and higher ejection fraction (63.3% vs. 58.9%, p = 0.001). Women were older, had higher prevalence of obesity, atrial fibrillation, and SAH, while men more often had coronary artery disease and history of tobacco use. Despite these differences, both groups had similar HF symptoms and comparable use of cardioprotective drugs.^35^The results showed that women had lower cardiovascular mortality (8.9% vs. 14.8%, p < 0.001) and fewer hospitalizations due to HF (13.4% vs. 18.2%, p = 0.008). After adjustment, the female sex was a protective factor against cardiovascular mortality (HR: 0.53; 95%CI: 0.40–0.73). However, women had worse health-related quality of life and higher functional limitation. Regarding treatment with spironolactone, there was a reduction in all-cause mortality in women (HR: 0.68; 95%CI: 0.48–0.96), but not in men ( *P* for the interaction = 0.190), suggesting possible sex-specific benefit.

The PURSUIT-HFpEF study, a multicenter prospective registry from East Asia, has investigated sex differences in diastolic dysfunction and clinical outcomes in patients with HFpEF. Women represented 55.2% of the cohort (481 out of 871 patients).^36^The study showed that women had a higher prevalence of diastolic dysfunction (52.8% vs. 32.0% in men, p < 0.001), independently of comorbidities, such as SAH and T2DM. Anemia and obesity were factors associated with diastolic dysfunction only in women. Despite the worse diastolic function, women had a similar rate of combined events (death/hospitalization due to HF) in a non-adjusted analysis. However, after multivariable adjustment, female sex was independently associated with a higher risk of clinical events (HR: 1.54; 95%CI: 1.14–2.07), mainly for hospitalizations due to HF.^30 , 36^

Both studies emphasize that, from the metabolic viewpoint, HFpEF is a different condition in women, with:

1. Higher diastolic dysfunction associated with factors, such as obesity and anemia; thus, screening is important.
2. Better global survival despite worse symptomatology, possibly because of fewer ischemic comorbidities.
3. Different response to therapies, such as spironolactone, which can be more effective in women.

### 3.3. Hormonal Aspects in Inflammation

Women's cardiovascular health is influenced by a complex interaction of hormonal and inflammatory factors that manifest in a particular way in different phases of life, such as puberty, pregnancy, and menopause. Hormonal fluctuations, especially estrogen and progesterone levels, play an important role in maintaining vascular integrity and cardiac function. Therefore, understanding how such changes relate to the increase in CVD risk is crucial.

In postmenopausal women, hypoestrogenism promotes important metabolic changes, such as increase in central adiposity, worsening of glucose metabolism, and elevation in the levels of total cholesterol, LDL-c, triglycerides, apolipoproteins, and lipoprotein a (Lp(a)).^37 , 38^In addition, endothelial dysfunction occurs, with consequent increase in IHD risk.

Estrogen acts by interacting with different receptors, such as ERα, Erβ, and GPER, activating genomic and non-genomic signaling pathways.^39^This results in inflammatory response modulation, reducing the production of pro-inflammatory cytokines, such as interleukin 6 (IL-6),^40^and increasing the expression of anti-inflammatory cytokines.^41^A key mechanism involves inhibition of nuclear factor kappa B (NF-κB) activity, reducing the transcription of inflammatory genes.^41^In addition, estrogen increases the production of NO in endothelial cells, contributing to vasodilation, and inhibits the expression of adhesion molecules, reducing leukocyte infiltration in the vascular wall.^42^

Endothelial function is benefited by estrogen that induces the expression of the enzyme endothelial nitric oxide synthase (eNOS), increasing NO production and promoting vasodilation. In addition, estrogen stimulates the regeneration of endothelial cells and inhibits the proliferation of vascular smooth muscle cells, preventing atherosclerosis progression.^42^

Chronic inflammatory processes represent another crucial underlying mechanism. Women with irregular cycles frequently have a pro-inflammatory status characterized by elevation in markers, such as C-reactive protein (CRP), IL-6, and tumor necrosis factor alpha (TNF-α). This systemic inflammation acts as an intermediate between menstrual dysfunction and atherogenesis.^18^

### 3.4. Inflammation in Cardio-Oncology

In addition to traditional RFs and MS ( Figure 3.1 ), women with cancer have an increased CVR because of the cardiotoxic effects of oncological therapies, such as those with anthracycline and trastuzumab. These therapies can lead to complications, such as HF, AMI, and stroke, even years after the end of oncological treatment.

**Figure 3.1:** Complications of metabolic syndrome.

Systemic chronic inflammation plays a significant role in the intersection of cancer and CVD. The tumoral inflammatory microenvironment and the side effects from oncological treatment contribute synergically to endothelial dysfunction, vascular remodeling, and atherosclerosis progression, especially in women. For example, thoracic radiotherapy has been associated with coronary inflammatory changes detected by biomarkers and the perivascular fat attenuation index (FAI), reflecting persistent vascular inflammatory activity. In addition, the prolonged use of aromatase inhibitors in breast cancer survivors has been associated with endothelial function worsening and higher risk of cardiovascular events.

In women undergoing oncological treatment, a multidisciplinary approach is essential, including baseline assessment of cardiac risk prior to the beginning of cancer therapy, which should be optimized to minimize cardiotoxicity. Interventions in lifestyle and maintenance of cardiovascular surveillance in the long run are also required. In addition, it is important to address racial and ethnic health disparities. Black and Hispanic women have a higher risk of cardiotoxicity and worse cardiovascular outcomes as compared to White women because of socioeconomic factors and inequalities in access to health care.^43 , 44^

### 3.5 Inflammation in Pregnancy and Preeclampsia

Preeclampsia is a multisystemic disease of pregnancy, whose pathophysiology has not been completely understood. Reduced placental perfusion, resulting from deficient trophoblastic invasion of maternal uterine wall, associated with the secretion of inflammatory cytokines and angiogenic factors, is believed to play a role in the disease. These factors contribute to endothelial dysfunction, vascular inflammation, and poor maternal perfusion. Endothelial dysfunction has been indicated as a major phenomenon responsible for preeclampsia and gestational hypertension.

Placental ischemia is related to the increased production of circulating antiangiogenic factors, such as soluble Fms-like tyrosine kinase-1 (sFlt-1) and soluble endoglin (sEng), that cause generalized endothelial dysfunction, NO pathway impairment, oxidative stress, excessive inflammation, imbalance in angiogenic factors, and loss of endogenous protective regulators.^45^Pregnant women with RFs associated with chronic inflammation (autoimmune diseases, obesity, pregestational hypertension, DM, and dyslipidemia) are more likely to develop preeclampsia. Inflammatory mediators might have local autocrine or paracrine effects, in addition to amplifying the effects of antiangiogenic factors.^46^

Pregnancy, even in normal conditions, is a state of oxidative stress because of increased maternal metabolism and placental activity. However, in preeclampsia, compensatory mechanisms fail, leading to the increased production of pathogenic factors and subsequent vascular dysfunction.^47^Observational and experimental studies have shown the association between inflammation and endothelial dysfunction. An important characteristic of systemic inflammation in preeclampsia is the predominance of Th1-type immunity and no Th2 biased immune response. However, normal pregnancy is characterized by a change to Th2-type immunity. In addition, the circulating levels of pro-inflammatory cytokines, such as IL-6, TNF-α, and chemokines IL-8, IP-10 (interferon-gamma-induced protein 10) and MCP-1 (monocyte chemoattractant protein 1) are elevated in preeclampsia.^45 , 46^

McCarthy *et al* . have investigated the role of mitochondrial dysfunction as a facilitator of oxidative stress, inflammation, apoptosis, and metabolic changes — all of which are crucial pathogenic intermediates in preeclampsia.^47^

Obesity increases the risk of preeclampsia. White adipose tissue secrets pro-inflammatory mediators that contribute to the chronic inflammatory state and metabolic complications of obesity. In addition, visceral adiposity is associated with metabolic RFs and complications, such as GD and preeclampsia.^48^The sFlt-1, the soluble form of the vascular endothelial growth factor (VEGF) receptor, is known to be secreted by adipocytes in non-pregnant women. Huda *et al* .^48^have shown that dysregulation of inflammatory pathways occurs predominantly in the visceral adipose tissue, with activation of macrophages and increased TNF-α and IL-6 expressions in that tissue, but not in the subcutaneous fat, emphasizing that, in preeclampsia, dysregulation of inflammatory pathways occurs predominantly in the visceral adipose tissue.

Metanalysis conducted by Guan *et al* .^49^has assessed the relation between pro- and anti-inflammatory biomarkers and their dynamic changes throughout preeclampsia progression. Women with preeclampsia had significantly higher levels of CRP, IL-4, IL-6, IL-8, IL-10, and TNF-α. The levels of pro-inflammatory cytokines were higher than those of the anti-inflammatory ones. Women with gestational age over 34 weeks had elevated levels of IL-6 and TNF-α. Higher systolic BP was associated with higher levels of IL-8, IL-10, and CRP. Such findings suggest that inflammatory imbalance is an independent RF for preeclampsia, and failure in anti-inflammatory autoregulation leads to disease progression.

Finally, recent studies have shown that neuroinflammation with participation of the autonomic nervous system can be present in the induction and evolution of inflammatory reactions associated with preeclampsia. In addition, the reduced autonomic regulation in patients with preeclampsia can be involved in the late fetal neurological maturation, with cognitive deficits and mental disorders. These findings show a link between maternal inflammation in preeclampsia and its impacts on fetal health and neurodevelopment.^50^

The factors associated with inflammation in women are summarized in Figure 3.2.

**Figure 3.2:** Factors associated with inflammation in women.

## 4. Implications of Sex Steroids for Cardiometabolic Health

### 4.1. Origin and Metabolism of Sex Steroids

The major sex steroids acting in the female body are estrogens, progesterone, and testosterone. These hormones are produced in the ovaries, adrenal cortex, and through peripheral conversion. Sex steroids have in common a cyclopentanophenanthrene ring derived from cholesterol. They are divided into three major groups according to the number of their carbon atoms as follows: the 21-carbon series includes corticosteroids and progestagens, being based on pregnane nucleus; the 19-carbon series includes all androgens, being based on androstane nucleus; and estrogens are 18-carbon steroids based on estrane nucleus.^51^

From cholesterol, which is obtained directly from blood stream, steroidogenesis originates natural sex steroids.^51^In mitochondria, an enzymatic hydroxylation system and a desmolase continue the process, forming the first steroid, pregnenolone. From that phase, steroidogenesis can progress via delta 5 pathway, which predominates in the follicle, to the synthesis of estrogens, or via delta 4 pathway, which predominates in the corpus luteum, to the synthesis of progesterone. Pregnenolone and progesterone are converted into androgens, androstenedione, and testosterone, in theca cells. Androstenedione, by the action of 17β-dehydrogenases, can be converted into testosterone and, through aromatization in granulosa cells, results in the formation of estrone and estradiol.^52^

Once synthesized, in the plasma those hormones are transported bound to SHBG (sexual-hormone-binding-globulin), CBG (cortisol-binding-globulin), or albumin, while a small fraction remains free. Then, sex steroids bind to hormone receptors present in several tissues, exerting their different biological effects.^53^These steroids are metabolized in the liver and excreted through the urine or bile.^51^

In addition, sex steroids can be extracted from plants in the pharmaceutical industry that manufactures products for clinical use. Their molecular structure is identical to that of hormones naturally produced by the female body (isomolecular), being, thus, called bioidentical hormones, whose effects are equal to those of endogenous hormones.

Synthetic sex steroids are produced in laboratories through a process involving several chemical steps and pharmaceutical engineering, originating molecules with different characteristics and biological potencies.^51 - 53^

### 4.2. Chemical Classification of Estrogens

#### 4.2.1. Natural Estrogens

The natural estrogens present in bloodstream are estradiol, estrone, estriol, and estetrol. Estradiol is the major and most potent estrogen secreted by the human ovary, originating mostly from androstenedione. Estrone is secreted by the ovaries in significant daily amounts. Estriol is the peripheral metabolite of estrone and estradiol, rather than secreted by the ovary. Estriol is formed through general metabolic "detoxification", which means the conversion of biologically active material into less active forms. Estetrol is produced only during intrauterine life by the fetal liver.^51 - 53^

#### 4.2.2. Synthetic Estrogens

Synthetic estrogens are classified as steroidal and non-steroidal, and their major examples are as follows:

- Steroidal - ethinyl estradiol, mestranol, estradiol valerate
- Non-steroidal - diethylstilbestrol, hexestrol, dienestrol Of those, estradiol valerate and ethinyl estradiol are currently the most clinically important.

Estradiol valerate is an estradiol ester, a prodrug that is rapidly metabolized to 17β-estradiol and valeric acid. The esterification of estradiol is aimed at improving its absorption and bioavailability after oral administration. After absorption, the esters are cleaved, releasing endogenous estradiol or 17β-estradiol. Thus, estradiol esters are considered bioidentical forms of estradiol.^54 , 55^Their use in isolation or combination with progestagens is mainly related to menopausal hormone therapy (MHT) and contraception.

Ethinyl estradiol, an agonist of estrogen receptors, is more resistant to metabolism as compared to estradiol and has better bioavailability when used orally. These differences favor ethinyl estradiol use in combined contraceptive pills, although they also increase the risk of thromboembolism and other rare adverse effects.^55^( Figure 4.1 ).

**Figure 4.1:** Chemical classification of estrogens.^55^

### 4.3. Chemical Classification of Progestagens

#### 4.3.1. Natural Progesterone

Progesterone, originally secreted by the ovaries, acts on the hormone-dependent target organs, especially endometrium and breast tissue. Its effects, however, go beyond, because they act on immunity, central nervous system, cardiovascular system, etc. From the physiological viewpoint, progesterone acts on tissues previously impregnated with estradiol. In addition, progesterone has a stereoisomer, dydrogesterone, available for oral use.^56^

#### 4.3.2. Progestagens

Progestagens are a large group of synthetic molecules that differ regarding their chemical structure and the molecule they are created from, which bestow them a different effect profile based on affinity with and potency in different steroidal receptors. There are three large groups: testosterone derivatives (norethindrone, levonorgestrel, norgestimate, desogestrel, etonogestrel, gestodene, dienogest), progesterone derivatives (medroxyprogesterone, cyproterone, megestrol), and spironolactone derivatives (drospirenone). ( Figure 4.2 ).

**Figure 4.2:** Classification of progestagens.

Progestagens are characterized by high affinity with progesterone receptor; however, according to their origin, they can have affinity with other receptors, leading to effects, such as androgenic/antiandrogenic, antiestrogenic/estrogenic, antimineralocorticoid, and glucocorticoid activity. This pharmacodynamic profile of progestagens should guide their selection based on their expected benefits, safety profile, and unwanted effects.^51 , 52 , 57^( Figure 4.3 ).

**Figure 4.3:** Biological effects of endogenous progesterone and other progestagens. Adapted from Kuhl et al.^55^MPA: medroxyprogesterone acetate; NETA: norethisterone acetate; LNG: levonorgestrel.

Progestagens have different progestational potencies, determined by their capacity to change the animal endometrium. Considering progesterone to have progestational potency of 1, progestagens are as follows in decreasing progestational potency order: desogestrel, levonorgestrel, nomegestrol, medroxyprogesterone, norethisterone, and drospirenone.^58^

From the pharmacokinetic viewpoint, progesterone and progestagens differ regarding their pharmaceutical forms and administration routes. For example, micronized progesterone can be administered via oral and vaginal routes. Vaginal administration offers good absorption, with lower fluctuations in plasma concentration than the oral route.^52^

In addition, progestagens differ in their binding to plasma proteins. Micronized progesterone binds to albumin and CBG, but not to SHBG. Norethindrone, desogestrel and norgestrel bind to albumin and SHBG, while medroxyprogesterone acetate binds mainly to albumin.^59^

Differences in binding to plasma proteins in addition to clearance and association or not with estrogens determine differences in the plasma half-life of progestagens. Most progestagens are administered daily.

### 4.4. Common Indications of Therapies with Estrogens, Progestagens, and Testosterone in Women

#### 4.4.1. Polycystic Ovary Syndrome

Polycystic ovary syndrome is a frequent indication of therapy with steroid hormones. It is a gynecological disorder associated with frequent endocrine disorders in women of reproductive age. In addition, metabolic disorders are present, increasing the CVR.^60^

Women with com POS can develop IR and dyslipidemia, which contribute to the development of CVD, such as atherosclerosis, SAH, and AMI.^61^These metabolic changes are boosted by the excess of androgens and weight gain, which can promote visceral fat accumulation and chronic inflammation. These factors combined create a metabolic environment that favors CVD development even in young women.^62 - 64^

The management of CVR in POS requires multidisciplinary approaches, including lifestyle changes, such as balanced diet and physical exercises, in addition to pharmacological interventions when necessary. Combined oral contraceptives (COCs) are often used in POS treatment to control menstrual changes and hirsutism, being effective to reduce the circulating levels of androgens, regulate the cycles, and protect the endometrium. However, that treatment does not improve IR and can even worsen it, depending on the type of progestagen used.^60^Insulin-sensitizing drugs, such as metformin or alternatives, such as pioglitazone^65^and myoinositol,^61 , 66^reduce IR, improving ovarian function. Statins can be indicated to control dyslipidemia, and anti-hypertensive drugs are essential for women with SAH. Treatments for obesity, such as glucagon-like peptide 1 (GLP-1) analogues (liraglutide and semaglutide), can be used.^67^Early identification of those metabolic changes and their proper treatment are crucial to reduce CVR in women with POS.

#### 4.4.2. Hormonal contraception

Sex steroids are often used for hormonal contraception. The COCs have two types of hormones (estrogen + progestagen) in different formulations.^68^The estrogen component offers cyclic endometrial stability and potentiates the suppressive effect of progestagen on the hypothalamic-pituitary-ovarian axis. The most used estrogen component is ethinyl estradiol, at current doses between 15μg and 30μg. New COCs with natural estrogens – estradiol (17β-estradiol and estradiol valerate) and more recently estetrol – appeared as alternatives with potential for lower hepatic and cardiovascular impact.^69^

In different COCs, those estrogens are combined with a progestagen. The major contraceptive effect of the progestagenic component is suppression of the luteinizing hormone (LH) secretion and of ovulation. In current formulations, several progestagens are used. They are synthetic progestagens that inhibit ovulation/pregnancy, and the most modern ones have been developed to cause fewer secondary/adverse effects. Pregnancy rates in the first year of COC use vary, being 0.3% with consistent and correct use. With typical use, it can reach 9%, showing the method's efficacy during real use^70^and emphasizing the importance of contraceptive counseling. The COCs are not recommended for women aged over 35 years with history of tobacco use, CVD, venous thromboembolism (VTE), SAH, DM with vascular complication, migraine with neurological symptoms, severe liver disease, liver tumors, breast cancer, and systemic lupus erythematosus. The contraceptive method safety has been well established by the Medical Eligibility Criteria for Contraceptive Use^71^( Figure 4.4 ).

**Figure 4.4:** Contraindications to the use of combined oral contraceptives. SLE: systemic lupus erythematosus.

Progestagen-only hormonal contraceptives contain only synthetic progestagen, such as desogestrel, norethisterone or drospirenone. Long-acting progestagen-only contraceptives can also be used as implants in the subdermal region of the forearm, and, in Brazil, the only implant approved for contraception is Implanon NXT^®^(etonogestrel), which has high contraceptive efficacy for 3 years.^72^The levonorgestrel intrauterine system (LNG-IUS) is a hormonal IUS that releases 20 µg of levonorgestrel per day with local intrauterine action and high contraceptive efficacy for 8 years. In addition, there is the IUS releasing 8 µg of levonorgestrel for 3 years. Long-acting progestagen-only contraceptives have the advantage of high efficacy for prolonged time and pregnancy rates lower than 1% per year, without depending on either correct use or user's motivation.^72^

#### 4.4.3. Menopausal Hormone Therapy: Regimens, Doses, and Routes, Focused on Cardiovascular Health

In women, hormonal fluctuations that begin in menopause transition, being followed by a gradual and irreversible reduction in estrogen production in menopause, although physiological, creates a "window of vulnerability" and marks the inflection point of cardiometabolic risk, with negative impact on vascular health, glucose and lipid metabolism, and body fat distribution.^73 , 74^Thus, MHT, especially estrogen, can interfere with that risk, as long as judiciously and individually prescribed. It should always be associated with integral health measures, with strategies for lifestyle, diet, and physical exercise practice.^75^

The MHT is the most effective treatment to relieve vasomotor symptoms (VMS) and genitourinary symptoms, as well as to prevent bone loss in peri- and postmenopausal women.^76^It should be initiated when menopausal symptoms begin to interfere with daily life. However, for women with premature ovarian failure (menopause < 40 years) or early menopause (between 40 years and 45 years), MHT is recommended to start as early as possible to reduce health risks in the long run.^77^Cardiovascular effects of MHT are strongly influenced by the type, dose, and route of administration used, as well as onset time of menopause ("window of opportunity"), with higher benefits and lower rates of adverse effects when MHT begins within 10 years of menopause onset.^77^Determining factors of MHT type and dose are: patient's preference, presence/absence of uterus, need for contraception, severity of symptoms, and comorbidities.^75^

Therapeutic regimens include estrogen-only therapy (for hysterectomized women) and combined therapy of estrogen and progestagen (women with uterus).^76^In the combined regimen, continuous use is indicated for women with postmenopausal amenorrhea for more than 12 months, while the sequential regimen is more indicated in perimenopause. MHT should be prescribed at the lowest effective dose to control symptoms.^78^The most used estrogen in MHT is 17β-estradiol, whose administration routes can be oral, transdermal (patch, gel, spray), or vaginal.^75^For proper relief of VMS and bone protection, low doses of estradiol (approximately 1 mg of oral estradiol or equivalent in other routes) are usually necessary.^75 , 76^In healthy women without cardiovascular risk factors (CVRFs), any route, including the oral route, can be prescribed for MHT.^75^However, differently from the oral route, the transdermal route avoids the ‘hepatic first-pass effect’ in estrogen metabolism, resulting in lower impact on coagulation factors, triglycerides, and CRP, which can reduce the risk for VTE and cardiovascular events. Thus, transdermal route is preferred for women with RFs, such as obesity, MS, tobacco use, hypertension, as well as risk for VTE.^79^

The use of "compounded bioidentical" hormones for MHT is not recommended because of concerns regarding quality, regulation, safety, efficacy, and lack of standardization of the products used.^75 , 76^

The only indication for testosterone supplementation in postmenopause is to treat hypoactive sexual desire disorder, after excluding other causes. Although data on the effects of androgenic therapy on cardiovascular health of postmenopausal women with hypoactive sexual desire disorder are limited, evidence indicates that, in physiological doses, transdermal testosterone does not significantly increase CVR.^80^

An individualized approach with the best evidence-based decision should be used. Maintaining MHT and not starting MHT in women ≥ 60 years seem associated with a better risk-benefit profile for cardiovascular events.^75^Transdermal route for estrogen at low doses combined with progestagens of safe profile represents a favorable option regarding CVR.^79^

### 4.5. Risks: Metabolic Effects, Thromboembolism, and Breast Cancer

#### 4.5.1. Metabolic Effects

MHT reduces visceral fat, IR, and the LDL-c to HDL-c ratio, but slightly increases triglycerides when used orally.^81 , 82^

A review has also confirmed lower levels of glycemia, insulinemia, and IR among MHT users.^83^The *Women’s Health Initiative* (WHI) study has found a lower significant risk of DM among MHT users as compared to those using placebo.^84^

There is evidence that the progestagen type used in MHT can interfere with metabolic effects. Medroxyprogesterone is known to have more glucocorticoid effects than norethisterone or progesterone.^85^Studies have suggested that progesterone and dydrogesterone interfere less with the benefits provided by estrogens,^86 , 87^while norethisterone, depending on the dose, can lead to loss of some of those benefits.^88^

#### 4.5.2. Thromboembolism

Ethinyl estradiol, a synthetic estrogen widely used in combination with progestagen in COCs, is associated with increased risk of VTE.^89^This risk is influenced by factors, such as dose of estrogen and type of progestagen present in the formulation. COCs containing levonorgestrel (second-generation progestagen) are associated with a lower risk of VTE as compared to those containing desogestrel, gestodene, drospirenone, or cyproterone (third- and fourth-generation progestagens).

When COC contains natural estrogens instead of ethinyl estradiol, the risk is slightly increased.^89^In addition, ethinyl estradiol of COCs can exacerbate the production of hepatic angiotensinogen, which increases BP via renin-angiotensin-aldosterone system.

When administered orally, they can increase the risk of venous thrombosis, odds ratio 1.58 (1.52–1.64), while the transdermal route is not associated with increased risk, odds ratio 0.93 (0.87–1.01).^90^In addition, a recent metanalysis has concluded that MHT combining conjugated equine estrogens plus progestagens increased systolic BP and the risk of hypertension, while other formulations, such as oral or transdermal estradiol plus progestagen, estradiol only, and tibolone, did not have significant effects on BP, showing that such effects can be influenced by different administration routes and formulations.^91^

#### 4.5.3. Breast Cancer

Breast cancer is a hormone-dependent neoplasm, and the proliferative effects of sex steroids in breast tissue are well known.^92^

MHT can associate with an increased risk for breast cancer. The WHI study has found that MHT with conjugated estrogens plus medroxyprogesterone acetate associated with eight more breast cancer cases per 10,000 women-year.^93^

However, effects differ depending on the progestagen used in the formulation. Observational studies have shown no increase in the risk when the association in MHT was with micronized progesterone^94^or dydrogesterone.^95^

Thus, different MHT formulations and doses also seem to have different effects on breast cancer risk.

## 5. Indicators of Cardiometabolic Health

### 5.1. Assessment of Reproductive Risk Factors with Implications for Cardiometabolic Health

This chapter addresses the relevant RFs of women's cardiometabolic profile in puberty, pregestational, gestational, and postgestational periods, as well as menopause transition, which will be detailed in chapters 7 and 8.

#### 5.1.1 Puberty and Pregestational Period

Menstrual cycle assessment has been suggested to be used as an additional vital sign in the investigation of women's general health.^96^In this scenario, age at menarche (early or late), menstrual cycle irregularities of endocrine origin, and POS have been associated with a higher risk of future CVD, especially atherosclerotic disease.^5 , 97^

##### 5.1.1.1. Age at Menarche

The CARDIA ( *Coronary Artery Risk Development in Young Adults* ) study, which enrolled 2788 women, aged 18-30 years, initiating in 1985-1986, and followed them up for 35 years (ages between 50 years and 65 years), has observed that early menarche associated later with adverse levels of glucose and lipids, and each earlier year of menarche, in relation to the mean age of 12 years, associated with greater body mass index (BMI) and visceral adiposity.^98^

Other studies have shown that early menarche is associated with elevation in glucose, insulin, BP, and body fat, as well as with higher risk of future CVD.^5^

A cohort study of 648 women stratified according to age at menarche (≤10, 11, 12, 13, 14, ≥15 years) and using the mean age of 12 years as reference, has reported that early or late menarche associated with a higher risk of future cardiovascular events, represented by all-cause death, non-fatal AMI, non-fatal stroke, or hospitalization due to HF.^99^

In a cohort of 1.2 million women, with mean age of 56±5 years, no previous heart disease, and followed up for 12 years, those with early (≤ 10 years) and late (≥ 17 years) menarche had a higher risk of IHD, stroke, and SAH.^100^

However, a metanalysis of 12 cohort studies carried out up to 2018, with 2,341,769 participants and 79,363 deaths, has shown that, for each 1-year increase in the age at menarche, there was a reduction in the relative risk for all-cause mortality, cardiovascular mortality, IHD mortality, and stroke mortality,^101^evidencing the need for further studies on the impact of age at menarche on future CVD.

##### 5.1.1.2. Menstrual Cycle Characteristics

A systematic review and metanalysis of observational studies performed up to 2022 has analyzed the association of oligomenorrhea and menstrual irregularity with CVR, observing the association of these changes with CVD, IHD, and AMI, but not with stroke, as documented in women with POS^5 , 97 , 98 , 102^(see chapter 8).

#### 5.1.2. Pregnancy and Postpartum Period

Pregnancy involves several physiological, hormonal, and metabolic transformations, fundamental to ensure proper fetal development and adaptation of the maternal body to new demands. Abnormal weight gain and changes in lipid and glycemic profiles, however, can be associated with adverse outcomes in pregnancy and postpartum for mother and infant.

##### 5.1.2.1. Weight changes in pregnancy

There is evidence that both low and excessive gestational weight gain (GWG) are associated with negative fetal and neonatal outcomes.^103^

Pregestational obesity favors the risk for gestational hypertension and GD, cesarean birth, and high birth weight, being recognized as a significant RF for spontaneous abortion, preterm delivery, metabolic disorders that complicate pregnancy, and higher rates of abnormal deliveries, stillbirths, and neonatal death. On the other hand, malnutrition can contribute to lower birth weight, placental abnormalities and complications, higher rates of surgical births, and higher fetal and neonatal mortality.^103 , 104^

Guidelines recommend GWG values, but analyses of more recent studies have suggested that this gain should be personalized considering three classes of obesity.^103^( Table 5.1 and Figure 5.1 ) (details in chapter 7).

**Table 5.1:** Weight gain in pregnancy according to the recommendation of the 2009 Institute of Medicine guidelines

BMI	< 18.5	18.5–24.9	25.0–29.9	> 30
Weight gain (kg)	12.5–18.0	11.5–8.0	7–11.5	5–9
Weight gain (kg) per week in the 2^nd^and 3^rd^trimesters	0.44–0.58	0.35–0.50	0.23–0.33	0.17–0.27

BMI: body mass index.

**Figure 5.1:** Implications of weight gain in pregnancy for cardiometabolic health. BMI: body mass index; DBP: diastolic blood pressure; GWG: gestational weight gain; HOMA-IR: homeostasis model assessment of insulin resistance; LDL-c: low-density lipoprotein cholesterol; MS: metabolic syndrome; TC: total cholesterol; TG: triglycerides; VLDL-c: very-low-density lipoprotein cholesterol.

##### 5.1.2.2. Gestational dysglycemia

Pregnancy is a condition of IR per se, potentially aggravated by increased pregestational IR in women with obesity. During pregnancy, IR is increased by 40-50%, which increases the risk for GD.^105^

Pregnant women with glucose intolerance are at risk for adverse gestational outcomes, even in the absence of GD.

The primary underlying pathophysiology leading to hyperglycemia is related to the presence of IR, insulin deficiency, or mixed pathophysiology. Studies have shown that insulin-resistant glucose intolerance in pregnancy is a subtype with high risk for adverse results in pregnancy, such as high birth weight, admission to neonatal intensive care unit, gestational arterial hypertension, and cesarean delivery.^106^

Glucose intolerance in pregnancy increases the risk for future diabetes. Thus, recognizing glucose intolerance in pregnancy enables better DM screening and prevention (more details in chapter 7).

##### 5.1.2.3. Gestational Dyslipidemia

Recent evidence shows that the increase in cholesterol, triglycerides, and metabolites associated with cardiometabolic dysfunction seems to have significant maternal and fetal vascular consequences.^107^

Historically, gestational dyslipidemia has been considered physiological and, thus, without clinical relevance. This physiological increase plays an essential role during pregnancy. However, high lipid levels in women who are predisposed to or have familial forms of hyperlipidemia can increase the risk for maternal-fetal complications. Hyperlipidemia during pregnancy is associated with preeclampsia, preterm delivery, and GD, and infants are more likely to develop vascular fatty streaks and experience increased risk for progressive atherosclerosis.^108^

##### 5.1.2.4. Postpartum Behavior

In the postpartum period, weight loss is usually incomplete and full recovery of metabolic functions is not always obtained. Postgestational weight retention is an RF for SAH, dyslipidemia, and MS. In addition, studies have shown that women with preeclampsia have a worse cardiometabolic profile up to 1 year after delivery.

Although lactation is a factor of metabolic protection because it stimulates energy expenditure and better glycemic and lipid control, it depends on adequate emotional, social, and support factors. Lack of specialized guidance for postpartum lactation can favor the persistence of obesity, IR, and dyslipidemia, in addition to increasing the risk for MS and SAH.^104^

#### 5.1.3. Menopause Transition

The lack of estradiol in menopause causes several metabolic, hormonal, inflammatory, and endothelial function changes, whose association favors the rapid progression of the atherosclerotic process in all arterial territories. Thus, from the eighth decade on, women have higher CVR than men do.^109 , 110^

Adequate management (primary prevention, early detection and proper treatment) of RFs that contribute to atherosclerosis development throughout life is crucial to delay CVD in peri- and postmenopausal women, as well as to primary and secondary prevention of events that lead to cardiovascular mortality.^111^This guidance on cardiovascular prevention should occur in all life stages prior to menopause, becoming more important from menopause on.^109 - 111^

##### 5.1.3.1. Age at menopause

For the large majority of women (90%), natural menopause occurs around the age of 51 years (age range: from 45 years to 55 years).^110 - 112^For 5% of women, menopause can occur spontaneously between the ages of 40 years and 45 years (early menopause), and, for 1%, before the age of 40 years, characterizing premature ovarian failure.^110 , 112^Women submitted to bilateral oophorectomy and chemotherapy or radiotherapy can progress to menopause as a consequence of these treatments, independently of their age at treatment time.^109 , 110 , 112^

The CVR is even higher in women who experience menopause early (naturally or secondary to some treatment), in those with moderate to severe VMS, and in those on MHT.^109 , 110^In early menopause, each year of earlier menopause onset increases by 3% the risk for CVD (IHD, stroke, and cardiovascular mortality).^110 , 112^

This higher risk seems to associate with a higher propension of this group to have a more atherogenic lipid profile, higher IR, SAH, higher risk of MS, as well as worse endothelial function and inflammatory markers, as compared to women who experience natural menopause.^110 , 112^

##### 5.1.3.2. Vasomotor Symptoms

Menopausal VMS, characterized by hot flushes and night sweats, can be present in up to 80% of women and last 7-9 years. However, when they appear earlier, they can last longer.^109 , 110^The intensity of VMS varies from mild to moderate and severe, and their diagnosis is associated with impairment of quality of life and increased use of health services.^113 - 115^

Several studies have shown that women with severe VMS, as compared to women with mild or no VMS, have a worse cardiometabolic profile, greater sympathetic hyperactivity, worse endothelial function, and higher incidence of subclinical atherosclerosis.^113 , 115^

##### 5.1.3.3. Menopausal Hormone Therapy

Several clinical trials have shown that MHT with estrogens increases CVR (IHD, stroke, thromboembolism) in postmenopausal women, and that risk seems to be attenuated when MHT is used in younger women (50-59 years), who are at the beginning of menopause or within 10 years from its diagnosis. In addition, that risk is attenuated with the use of low doses of hormones and routes of administration other than the oral one, notably the transdermal route.^109 , 110^

Current guidelines establish that MHT has scientific evidence-based indication only to treat VMS in women without contraindication and should not be used in those with established CVD or high CVR.^78 , 109 , 110^

#### 5.1.4. Postmenopausal Metabolic Health

Menopause is associated with hormonal changes that result in an imbalance between estrogen and testosterone. The ovaries, despite estrogen production cessation, produce testosterone at a lower rate, leading to its relative excess. Testosterone is associated with increased visceral fat and BP levels in postmenopausal women.^116^

The hormonal fluctuations and physiological changes related to ageing determine an impaired metabolic status, characterized by IR, increased total body fat, sarcopenia, and accumulation of abdominal fat.^117^

##### 5.1.4.1. Menopausal metabolic changes

Insulin resistance is defined as an inadequate response to insulin in tissues (adipose, skeletal muscle), central nervous system, and liver, being one of the major factors leading to hyperglycemia and T2DM, along with impaired insulin secretion.

Epidemiological evidence has suggested significant protection against IR in premenopausal women, who are more sensitive to insulin as compared to men. This metabolic advantage, however, disappears gradually after menopause or when IR progresses to hyperglycemia and DM. Thus, menopause is associated with a higher risk of glucose intolerance, and an increase in BP and triglyceride levels, as well as in the risk for MS, with consequent acceleration of CVR.^117 , 118^

##### 5.1.4.2. Diabetes mellitus

The changes described make women more likely to develop T2DM. Evidence suggests that women with menopausal VMS have higher risk of developing T2DM as compared to those who do not have such symptoms. In a data analysis from the WHI, the risk of developing T2DM was 18% higher in women with VMS, increasing in parallel with the severity of those symptoms.^119^

Women who develop DM before the age of 20 years tend to experience early menopause, while menopause is delayed in those with T2DM of late onset.^119^

A publication of the SWAN Study with a 20-year follow-up has concluded that women with multiple physical and psychological symptoms, as well as moderate to severe menopausal symptoms, had earlier onset of DM and MS.^119^

The MHT was associated with a reduction in the risk of developing T2DM in women without previous T2DM, and, in those with T2DM, MHT leads to better glycemic control.^117^

##### 5.1.4.3. Dyslipidemia

Estrogen has a protective role in the cardiovascular system, and its ovarian production uses LDL-c as a substrate. In menopause, circulating LDL-c cannot be used to synthesize estrogen, resulting in the reduction of estrogen production and a mild increase in LDL-c serum levels. In addition, the size and density of LDL-c particles change in menopause. Studies have shown that the levels of small and dense LDL-c particles increase from 10-13% in premenopausal women to 30-49% after menopause.^37^

Menopausal estrogen deficiency impairs the hepatic and intestinal functions related to lipid metabolism and transportation, which can increase plasma lipid levels and accelerate atherosclerosis progression.^120^

The SWAN study has suggested that the antiatherogenic function of high-density lipoprotein cholesterol (HDL-c), which is its ability to promote cholesterol reverse transport, can decrease during menopause, when less elevated HDL-c levels are associated with atherosclerosis.^109^

### 5.2. Anthropometric Indicators

Cardiometabolic health is a pillar of well-being, encompassing integrity of the cardiovascular system and metabolism. In women, this is influenced by hormonal changes, reproductive events, and biological factors that manifest over the course of a woman's life, from childhood to old age.^110^

Anthropometric indicators are body measures that provide information on body fat composition and distribution, reflecting cardiometabolic risk. Simple and accessible, these measures are fundamental to large-scale populational screening.^110 , 121^Therefore, they should be performed routinely, with attention to changes over the course of life.

#### 5.2.1. Body Mass Index

The BMI, calculated as weight (kg) / height² (m), classifies the nutritional status as follows: < 18.5 (low weight); 18.5-24.9 (normal); 25-29.9 (overweight); and ≥ 30 (obesity). An elevated BMI is associated with SAH, dyslipidemia, and DM. However, it has limitations, such as not differentiating slim mass from fat and not assessing adipose distribution, which reduce its accuracy in elderly or athletic women.^122 , 123^

#### 5.2.2. Waist circumference – Brazilian population

Waist circumference measures visceral fat, a more robust predictor of cardiometabolic risk than BMI. Visceral fat is associated with IR and chronic inflammation, central factors in CVD pathogenesis.^124 , 125^In women, waist circumference > 88 cm indicates high risk, according to the World Health Organization guidelines.

#### 5.2.3. Waist-To-Hip Ratio

Waist-to-hip ratio (WHR) compares waist circumference to hip circumference, reflecting fat distribution. A WHR > 0.85 in women suggests central fat accumulation, associated with higher risk of MS. Studies have indicated that WHR is superior to BMI in predicting cardiovascular events, especially after menopause.^124 , 126^

#### 5.2.4. Body Fat Percentage

Body fat percentage is an important parameter to assess cardiometabolic risk, especially because women tend to have a higher subcutaneous fat proportion as compared to men. Hormonal changes, such as those of puberty, pregnancy, and menopause, have a direct impact on body fat distribution and accumulation, and can increase the risk for IR, dyslipidemias, and chronic inflammations.^126 , 127^

#### 5.2.5. Waist-To-Height Ratio

Waist-to-height ratio (WHtR) is a predictive measure of central adiposity and cardiometabolic risk. Values greater than 0.5 are strongly associated with higher risk for SAH, T2DM, and cardiovascular events. This marker is particularly relevant in menopause transition, when there is a trend towards abdominal fat accumulation.^128 , 129^

### 5.3. Biomarkers

#### 5.3.1. Lipid Profile (Total Cholesterol, LDL-c, HDL, Triglycerides)

LDL-c promotes vascular inflammation and cholesterol build-up in the arterial intima layer, while dysfunctional HDL-c (common in women with IR or MS) loses its antioxidant ability and of reverse transport, favoring plaque accumulation.^130^High triglyceride levels (>150 mg/dL) associates with small and dense LDL-c particles, as well as with particles similar to remnants, which intensify plaque instability.^131^As previously cited in this chapter, menopause promotes metabolic profile changes due to estrogen decline, increasing LDL-c (∼10-15%) and reducing HDL-c, as well as its protective function, worsening dyslipidemia, especially in women with visceral obesity or DM.^73^Thus, nonpharmacological strategies, such as Mediterranean diet and aerobic exercises, improve HDL-c functionality and modulate triglycerides.

#### 5.3.2. Glycemia and Glycated Hemoglobin

Chronic hyperglycemia and elevated glycated hemoglobin (HbA1c) in women increase the CVR via mechanisms, such as glycation of vascular proteins, endothelial dysfunction, and activation of inflammatory pathways, which accelerate atherosclerosis and plaque instability.^132^Insulin resistance, common in conditions such as POS and previous GD, induces atherogenic dyslipidemia (reduced HDL-c, elevated triglyceride) and oxidative stress, exacerbating vascular injury.^133^

In postmenopause, estrogen decline reduces insulin sensitivity and increases metabolic dysfunction, correlating with higher arterial stiffness and systemic inflammation.^134^Based on findings from the SUSTAIN and EMPA-REG OUTCOME studies,^135 , 136^guidelines recommend for most women with T2DM: HbA1c <7%; and the use of drugs with proven cardiovascular benefit for secondary prevention, such as GLP-1 agonists (ex.: semaglutide) and sodium-glucose cotransporter type 2 (SGLT2) inhibitors (ex.: empagliflozin), which reduce cardiovascular events and hospitalizations due to HF. Assessment of subclinical atherosclerosis (ex.: coronary artery calcium score) and aggressive management of comorbidities (SAH, dyslipidemia) are essential. Nonpharmacological strategies, such as Mediterranean diet and aerobic exercises, modulate inflammation and improve insulin sensitivity.

#### 5.3.3. C-reactive Protein

Elevation of us-CRP in women is associated with increased CVR due to chronic vascular inflammation, which promotes endothelial dysfunction, atherosclerotic plaque instability, and thrombotic activation.^137^Hormonal factors modulate that relation, such as menopause, in which estrogen decline increases us-CRP (∼15-25%), which correlates with higher arterial stiffness and exacerbated inflammatory response.^73^

Comorbidities, such as autoimmune diseases (ex.: systemic lupus erythematosus), as well as oral MHT can increase us-CRP.^138^The use of statins (ex.: rosuvastatin) for patients with elevated us-CRP reduced cardiovascular events independently of LDL-c levels, as evidenced in the JUPITER study.^137^Adjuvant strategies include diet and aerobic exercise that modulate inflammation, especially in postmenopausal women.^139^Thus, us-CRP interpretation should be added to hormonal context, comorbidities, and lifestyle, guiding the treatment to mitigate CVR.

#### 5.3.4. Fibrinogen

Fibrinogen is a soluble plasma glycoprotein, synthesized in the liver and involved in platelet aggregation, endothelial injury, and plasma viscosity. It plays a central role in the formation of thrombi. In addition, it is a protein of the acute phase of inflammation, induced mainly by IL-6.^140 , 141^

Women have higher circulating levels of fibrinogen independently of age, as well as higher levels of functional fibrinogen, as determined by using thromboelastography estimates of fibrinogen contribution to clot resistance.^141^

Differences in fibrinogen function and circulating levels between women and men define a behavior called sex dimorphism. Such differences are associated with sex hormones, and estradiol is an important mechanistic mediator in coagulation induction.^141 - 143^

Epidemiological data have shown the important predictive role of fibrinogen in IHD,^140^and a study published in 2025 with 5690 participants showed a relation between high fibrinogen levels and all-cause mortality, suggesting that fibrinogen is a potential biomarker of mortality risk.^143 , 144^

#### 5.3.5. Homocysteine

Elevated homocysteine levels accelerate the development of atherosclerotic plaque in arteries via mechanisms, such as increased oxidative stress, LDL-c oxidation, NO depletion, endothelial dysfunction, inflammatory processes, epigenetic changes, and microRNA regulation.^145^

Changes in diet and lifestyle, regular physical activity, tobacco use cessation, and alcohol consumption reduction are essential to control hyperhomocysteinemia.^145^

#### 5.3.6. Adipokines

Adipokines are peptides secreted by adipocytes, of which leptin and adiponectin are the most frequently known and studied. Normal physiological levels of adipokines are essential to maintain adequate cardiovascular function.

Leptin promotes satiety and regulates energy expenditure. Hyperleptinemia is present in obesity and T2DM, reflecting a state of leptin resistance associated with atherogenic processes, endothelial dysfunction, low-grade chronic inflammation, and vascular dysfunction. There are clear sex-related differences in circulating leptin levels, which are three to four times higher in women as compared to men.^146 , 147^

Adiponectin has anti-inflammatory, antiatherogenic, and insulinotropic effects. Its action includes improvement in insulin sensitivity, oxidative stress reduction, and modulation of endothelial inflammatory response. Adiponectin serum levels have been inversely associated with CVD risk and considered a biomarker of cardiovascular protection. Women have significantly higher total adiponectin circulating levels as compared to men in healthy populations. Sex-related differences in adiponectin levels have not been well characterized, but differences in regional fat distribution (subcutaneous vs. visceral) between women and men can contribute to the sex-related differences.^146 , 147^

The use of biomarkers for clinical assessment is summarized in Figure 5.2.

**Figure 5.2:** Use of biomarkers for clinical assessment. HOMA-IR: homeostasis model assessment of insulin resistance; us-CRP: ultrasensitive C-reactive protein.

## 6. Cardiometabolism in Childhood and Adolescence

The age of puberty onset has significant clinical and epidemiological implications, because variations in its timing are associated with several metabolic and cardiovascular outcomes. Menarche, defined as the first occurrence of menstruation, indicates the hypothalamic-pituitary-gonadal axis maturation, marking the onset of female reproductive ability. Studies have shown a secular trend towards a lower age of menarche onset, with a reduction of three months per decade in the past 50 years, a reflex of nutritional improvement and lifestyle changes.^148^Currently, the mean age of menarche onset is 12-13 years in developed countries, while, in low-income regions, that event occurs later.^149^Understanding the determinants of that variability and its metabolic impacts is crucial for cardiovascular health.^150^

### 6.1. Definitions and Diagnostic Criteria

When sex characteristics develop before the age of 8 years in girls, it is considered early puberty. Early menarche is defined as the first occurrence of menstruation before the age of 9.5 years. On average, menarche occurs two years after the development of breasts, and early menarche has higher prevalence in Black and Hispanic girls. However, the Brazilian consensus emphasizes the need for individual clinical assessment, considering each patient's context.^151^Late menarche is defined as the first occurrence of menstruation after the age of 15 years or the absence of menarche up to 3 years after complete secondary sexual development. Its causes include hypothalamic, pituitary, and gonadal changes, in addition to significant energy deficits.^152^

#### 6.1.1 Pathophysiology

Puberty depends on the activation of the hypothalamic-pituitary-gonadal axis, regulated by the pulsatile release of gonadotrophin-releasing hormone (GnRH). This process stimulates the production of LH and follicle-stimulating hormone (FSH) by the pituitary, which stimulates the ovaries to secrete estrogens.

In early puberty, the activation occurs early, usually associated with high hypothalamic sensitivity to adipokines, particularly to leptin. This hormone, produced by the adipose tissue, signals to hypothalamus the existence of adequate energy reserves for reproduction. In obese girls, elevated leptin levels can exacerbate that trigger, accelerating puberty onset. In addition, the early exposure to endocrine disruptors, such as bisphenol A and phthalates, can interfere with the natural hormonal signals and precipitate the reproductive axis activation.

In late puberty, the reproductive axis activation is frequently delayed due to chronic energy deficits common in athletes or patients with eating disorders. Low energy storage reduces the secretion of leptin and other adipokines, impairing signaling to the hypothalamus. In some cases, there is resistance to the metabolic signals of insulin and leptin or changes in kisspeptin signaling, a hypothalamic neuropeptide essential for GnRH secretion.^151^

These mechanisms illustrate how nutritional status and body composition directly influence menarche timing and its metabolic repercussions. For example, obesity can induce metabolic dysfunctions that accelerate sexual maturation, while severe calorie deficits, observed in athletes or girls with eating disorders, such as anorexia nervosa, delay the process.

#### 6.1.2. Early Menarche, Late Menarche, and Cardiometabolic Risk

Early menarche is strongly associated with adverse metabolic outcomes. One of the major impacts is the increase in BMI and central adiposity, with increased risk for adulthood obesity by approximately 30-60% as compared to women with normal age of menarche.^150^( Figure 6.1 ).

**Figure 6.1:** Association of cardiovascular risk with age of menarche. The extremes of menarchal age associate with a future risk of cardiometabolic disorder and cardiovascular disease and should be considered enhancers of cardiovascular risk. Source: Dastmalchi and Gulati.^154^

Other relevant consequence is IR, frequently identified in that profile and characterized by elevated levels of fasting insulin and early changes in glucose metabolism, often regardless of BMI.

In addition, unfavorable lipid profile changes, such as elevation of triglycerides and LDL-c, and HDL-c reduction, are common. These are findings of atherogenic dyslipidemia, which increases CVR.

In addition, there is a 1.5- to 2.5-fold increase in the risk for MS, associated with the combination of central obesity, SAH, IR, and dyslipidemia.^100^Even after adjusting for confounding factors, such as socioeconomic level, the metabolic impact of early menarche remains clinically important.

In late menarche, metabolic outcomes are more heterogeneous. Usually, patients have lower BMI and less adiposity, which reflects a more favorable metabolic profile. However, in cases related to energy deficits, such as eating disorders, malnutrition becomes a critical factor, influencing negatively metabolism and bone health^152^( Figure 6.1 ).

Insulin sensitivity is better in women with late menarche as compared to those with early menarche.

In late menarche, bone health is frequently impaired, with increased risk for osteopenia and fractures in adulthood. The reduced bone mineral density in those cases emphasizes the need for preventive interventions and continuous monitoring.^100 , 152^

Finally, the extremes of menarchal age correlate with a "U" shaped pattern of CVR. In early menarche, there is an increase in systolic and diastolic BP, carotid intimal thickening, and higher predisposition to subclinical atherosclerosis. In contrast, in late menarche, hormonal and nutritional disorders elevate the likelihood of late complications.^153^

### 6.2. Menstrual irregularities and cardiometabolic risk

Excluding structural causes, such as uterine myomatosis and polyps, the menstrual disorders related to hormonal causes are common manifestations during female reproductive life, affecting 14-25% of women at childbearing age, with peak incidence in reproductive life extremes, such as menarche and perimenopause.^155^These changes, classified as oligomenorrhea, polymenorrhea, amenorrhea, menorrhagia, and metrorrhagia, go beyond gynecological changes and emerge as important indicators of cardiometabolic vulnerability. Contemporary investigations have evidenced solid correlations between menstrual cycle anomalies and the development of CVD, emphasizing the need for an integrative approach for those patients’ assessment.^156^

#### 6.2.1. Pathophysiological Mechanisms

The interrelation between irregular menstrual cycles and cardiometabolic risks involves multiple interconnected pathophysiological processes. The hypothalamic-pituitary-ovarian axis plays an essential role in that association, because hormonal imbalances impact reproductive and cardiovascular functions simultaneously. Oscillations in estrogen and progesterone levels directly modulate insulin sensitivity, lipid metabolism, endothelial function, and BP homeostasis.^44 , 155^

Insulin resistance is the central element in the pathophysiology of certain conditions, such as POS, promoting both menstrual irregularities and dyslipidemia, SAH, and visceral adiposity. Longitudinal follow-up studies have shown that women with IR have a substantially elevated risk for T2DM and cardiovascular events over the course of their life cycles.^156^

#### 6.2.2. Clinical Assessment and Biomarkers

The assessment of women with menstrual irregularities requires a comprehensive approach of CVRFs. Detailed menstrual history is an essential component of risk stratification that needs to be complemented with body composition analysis, BP measurement, and investigation of conventional RFs.^153 , 157 , 158^

#### 6.2.3. Therapeutic Interventions

The management of adolescents with menstrual irregularities at high cardiometabolic risk requires multidimensional strategies. Lifestyle changes are the basis of treatment, which includes regular physical activity, cardioprotective eating patterns, body weight control, and tobacco use cessation.^157^Pharmacological interventions require customization according to individual risk profile.

Despite significant advances, there are still important knowledge gaps of the relation between menstrual irregularities and cardiometabolic risks.

In clinical practice, the following measures are recommended: incorporation of detailed menstrual assessment into CVR stratification; early cardiometabolic screening in adolescents with persistent menstrual irregularities; adoption of a multidisciplinary approach in the management and early diagnosis of conditions, such as POS, frequently diagnosed at puberty onset;^109 , 159 - 161^and development of educational programs focused on the interrelation of reproductive and cardiovascular health.^44 , 162 , 163^These changes are detailed in chapter 7.

### 6.3. Obesity, Eating Disorders, and Cardiovascular Risk: Long-Term Impacts

Obesity in childhood and adolescence is associated with a series of adverse physical and psychological outcomes. From the physical viewpoint, there is a significant increase in the risk of developing chronic comorbidities, such as CVD, SAH, dyslipidemia, IR, T2DM, and metabolic dysfunction-associated steatotic liver disease (MASLD).^164^From the psychological viewpoint, obesity relates to deterioration of emotional health, including higher prevalence of stress, depressive symptoms, and low self-esteem.^165^

#### 6.3.1. Eating Disorders and Obesity

The relation between obesity and eating disorders is known to be bidirectional. Children and adolescents with obesity are at higher risk of developing eating disorders, especially bulimia nervosa and periodic compulsive eating disorder. These disorders can aggravate the physical and psychological consequences already associated with obesity, establishing a pathological cycle of weight gain and eating-disordered behaviors.^165^

The American Academy of Pediatrics stresses the importance of early screening for eating-disordered behaviors and implementation of timely interventions. Such measures can promote significant improvement in clinical outcomes.^164^Dysfunctional eating behaviors identified during middle childhood correlate with the appearance of obesity and future cardiometabolic complications, justifying the need for early and longitudinal monitoring.^165^

#### 6.3.2. Hereditary Factors and Prognosis of Eating Disorders

Several genetic and hereditary factors influence the risk and prognosis of eating disorders in childhood and adolescence.^167^The major ones include:

1. Genetic *loci* and heritability^167^
  Studies have shown specific *loci* associated with anorexia nervosa (1p33–36 region) and bulimia nervosa (10p14), with estimated heritability between 48% and 74%.
2. Specific genes^167^
  Mutations and genes, such as *ESRRA, HDAC4* , *AGRP, GHRL,* and *BDNF,* have been associated with appetite regulation and increased risk of developing eating disorders.
3. Family history^168^
  Family history of eating disorders or psychiatric disorders significantly increases individual risk, as evidenced in studies with monozygotic twins.
4. Comorbid psychiatric disorders^167^
  There is genetic overlapping of eating disorders and other psychiatric conditions, such as obsessive-compulsive disorder and depression, worsening the clinical course.
5. Autoimmune and autoinflammatory diseases
  There is evidence that the presence of autoimmune diseases in the family history can contribute for the risk of developing eating disorders, suggesting possible immune system involvement.

#### 6.3.3. Future Consequences of Eating Disorders

Eating disorders that begin in childhood have long-lasting repercussions. Longitudinal studies have shown that inadequate early eating patterns are associated with disorders diagnosed in adolescence.^169^For example, childhood hyperphagia is related to increased risk for eating compulsion and periodic compulsive eating disorder, while extreme food selectivity and persistent malnutrition increase the risk for anorexia nervosa.^170^

The consequences extend beyond adolescence. Individuals affected by eating disorders in their youth have an elevated risk of developing psychiatric disorders, such as depression, anxiety disorders, and abuse of illicit substances in young adulthood. In addition, self-harm behaviors are more prevalent in that group. Moreover, early eating symptoms are associated with adverse weight outcomes, such as obesity or extreme thinness, and persistence of eating-disordered behaviors up to adulthood.

Early intervention associated with therapeutic strategies, such as cognitive-behavioral therapy, family interventions, and treatment of psychiatric comorbidities, is strongly advised.^171^

#### 6.3.4. Childhood Obesity and Cardiovascular Outcomes

According to the American Heart Association, children with severe obesity frequently have multiple and simultaneous CVRFs, such as hypertension, dyslipidemia, and IR, since young age.^171^

Childhood obesity has substantial implications for cardiovascular health in adulthood. It is strongly associated with the development of hypertension, dyslipidemia, IR, T2DM, and MASLD. These factors persist and evolve, increasing cardiovascular morbidity and mortality.^171^

The systematic review by Sommer and Twig has shown increased incidence of CVD, such as MI and stroke, among individuals who were obese in childhood and adolescence.^172^Maintaining obesity from childhood to adulthood was associated with a significantly higher CVR as compared to individuals whose weight was normalized.^172^

The classical *Bogalusa Heart Study* has shown that such physiological changes are related to increased carotid intima-media thickness and arterial stiffness, which are markers of subclinical atherosclerosis.^173^

In addition, cardiac structural changes, such as increased left ventricular mass and myocardial hypertrophy, are common in those individuals and correlate with worse future cardiovascular outcomes.

#### 6.3.5. Eating Disorders and Cardiovascular Risk

Eating disorders initiating in childhood, such as anorexia nervosa, result in persistent cardiovascular changes. Even after clinical recovery, individuals can have increased carotid artery stiffness, reduced aortic distensibility, endothelial dysfunction, and vagal hyperactivity.^173^

In addition, maladaptive eating behaviors during childhood are associated with the development of obesity and SAH during adolescence, both of which are CVRFs.^171^

The American Heart Association emphasizes that obese children with eating-disordered behaviors tend to have increased left ventricular mass, arterial stiffness, and elevated BP, factors that persist up to adulthood and contribute to the development of atherosclerosis and other chronic CVDs.^171^

## 7. Cardiometabolic *Continuum* and Reproductive Age

The cardiometabolic *continuum* refers to a process of The cardiometabolic continuum refers to a progressive process of interconnected metabolic and cardiovascular changes, often beginning in childhood. In women, the phases of reproductive life — menarche, pregnancy, puerperium, and menopause — directly influence this risk. Pregnancy is a physiological state with deep cardiometabolic changes, necessary to sustain fetal growth and adapt the maternal organism to new demands. Of such changes, increased cardiac output, expansion of plasma volume, reduction in systemic vascular resistance, lipid profile changes, higher peripheral insulin resistance, inflammatory activation, and vascular remodeling stand out. These adaptations can decompensate previous clinical conditions or reveal latent cardiometabolic vulnerabilities, with implications for immediate maternal health and future CVR.^174 - 178^

Recognizing this relation in the different phases of a woman's life is essential to develop preventive strategies and screening throughout life.

Figure 7.1 illustrates cardiometabolic changes in pregnancy.

**Figure 7.1:** Cardiometabolic changes in pregnancy.

### 7.1. Polycystic Ovary Syndrome

The POS is the most common reproductive endocrinopathy, present in 6-10% of women. It is strongly associated with central obesity, IR, glucose intolerance, and dyslipidemia (increased LDL-c and triglycerides, decreased HDL-c), as well as with higher risk for SAH and early atherosclerosis,^159^hepatic steatosis, sleep respiratory disorders with higher incidence of obstructive apnea,^5 , 97 , 98^and depression.

The pathophysiology of POS is complex and involves dysregulation of the hypothalamic-pituitary-ovarian axis, reduced hepatic synthesis of SHBG, and functional ovarian hyperandrogenism, a primary dysfunction of ovarian theca cells, in addition to elevated anti-müllerian hormone levels, which exacerbates even more ovarian dysfunction.^160^

Currently, there are four recognized phenotypes of POS, each one with different long-term implications for metabolism and health: 1) hyperandrogenism + oligoanovulation + polycystic ovary morphology; 2) hyperandrogenism + oligoanovulation; 3) hyperandrogenism + polycystic ovary morphology; and 4) oligoanovulation + polycystic ovary morphology.^161^The phenotypes with hyperandrogenism have worse metabolic profile.^62^

The major metabolic changes related to POS are as follows: obesity, in approximately 50% of cases; IR, in 60-95% of cases, leading to glucose intolerance in 31-35% of cases; and T2DM, in 7.5-20% of cases.

Insulin resistance plays a central role in metabolic and cardiovascular complications. The resulting hyperinsulinemia stimulates the hepatic production of triglycerides and reduces HDL-c levels, favoring the accumulation of atherosclerotic plaques. In addition, IR promotes neoglucogenesis, increasing the amount of glucose available (dysglycemia), reducing sex-steroid-binding proteins and insulin-like growth factors, worsening the clinical findings and increasing the chronic inflammatory process.^63^This leads to higher levels of CRP and pro-inflammatory cytokines, which also accelerate vascular injury and endothelial dysfunction.^64^

Dyslipidemia is the most frequent abnormality in POS, presenting with low HDL-c levels and high concentrations of triglycerides, and increased LDL-c levels can occur.^179^

Overweight and obesity, present in many women with POS, worsen the risk because the visceral adipose tissue releases free fatty acids and inflammatory adipokines, exacerbating IR and dyslipidemia. In addition, hyperandrogenemia in POS is associated with an atherogenic lipid profile, with increased oxidized LDL-c and reduced HDL-c, increasing the risk of early cardiovascular events.^62^

Analysis of 39 systematic reviews and metanalyses published up to 2019 has shown that women with POS are at higher risk for CVD.^102^This risk remained when stroke and IHD were assessed separately, but there was no association with HF. In addition, the risk of cardiovascular events was higher in young women of reproductive age with POS as compared to normal controls, but no association was observed in postmenopausal women with POS.^102^Currently, most of the therapy is centered on the patient's major complaint, reducing hyperandrogenism symptoms, restoring menstrual regularity, and obtaining conception in women.

The up-dating of the *2023 International Evidence-based Guideline for the Assessment and Management of Polycystic Ovary Syndrome* reintegrated the *2018 International Evidence-Based Guideline for the Assessment and Management of Polycystic Ovary Syndrome* , encompassing a wide synthesis of evidence and recommendations for POS.^162^The major up-dates were as follows:

- strengthening of the recognition of broader characteristics of POS, including metabolic RFs, CVD, sleep apnea, prevalence of psychological characteristics, and high risk for adverse outcomes during pregnancy;
- emphasis on the diverse and rarely recognized burden of the disease and on the need for improving health professional education;
- maintained emphasis on a healthy lifestyle, emotional well-being, and better quality of life;
- emphasis on evidence-based medical therapy.

Weight loss should be prioritized early.^163^

The POS changes associated with increased CVR are summarized in Figure 7.2.

**Figure 7.2:** Polycystic ovary syndrome changes associated with increased cardiovascular risk.

### 7.2. Infertility and its Treatment

Infertility, defined as the absence of clinical pregnancy after 12 months of regular and unprotected sexual intercourse, affects 8-12% of reproductive-age couples.^175 , 176^Female causes include ovulatory dysfunctions, tubal and uterine factors, low ovarian reserve, obesity, and hormonal disorders.^177^The most frequent causes are endometriosis, which is an inflammatory pathology, and POS, which is known to cause increase in androgen hormones and MS.^178 , 179^Mulder *et al.* , assessing women with and without infertility in the same age group, have shown that infertile ones had a greater tendency to have specific cardiometabolic RFs, with an increase in metabolic disorders, such as obesity and dyslipidemia (total cholesterol, LDL-c, and triglycerides), but they found no change in fasting glycemia, IR, and BP.^180^( Figure 7.3 )

**Figure 7.3:** Most frequent causes of female infertility. POS: polycystic ovary syndrome.

Endometriosis has a prevalence of up to 10% of the female population, is a frequent cause of infertility, and will be addressed separately.^181 , 182^

The treatment for infertility has cardiometabolic repercussions. Ovarian stimulation with clomiphene, letrozole, and gonadotrophins can induce ovarian hyperstimulation syndrome with risk for thromboembolic events.^183^ *In vitro* fertilization leads to higher risk of VTE, preeclampsia, and cardiovascular events in the long run. In addition, women getting pregnant after infertility treatment have higher risk for GD and hypertensive disorders of pregnancy.^182^

Interventions in lifestyle, such as balanced diet, physical activity practice, and behavioral changes, can not only improve cardiovascular health, but increase infertility treatment success as well. Weight loss in obese women is recommended to improve reproductive health and reduce complications during pregnancy. There is still limited evidence that weight loss improves *in vitro* fertilization outcomes. Weight control, however, is considered beneficial to fertility and cardiometabolism.^184^

### 7.3. Weight changes in pregnancy

Weight changes during pregnancy are intrinsically related to physiological and metabolic adaptations to meet fetal demands. Monitoring GWG is an important part of prenatal consultation and deserves multidisciplinary attention. Pregestational BMI and GWG significantly impact maternal and infant outcomes, as described in chapter 5. Both excessive and insufficient weight are associated with maternal and fetal cardiometabolic complications, such as preterm delivery and small for gestational age newborns.^165^

Brazilian researchers have developed curves and recommendations of specific GWG for the Brazilian population adopted by the Ministry of Health from 2022 on.^186^

Chart 7.1 shows the recommendation for GWG adjusted to pregestational BMI in single and twin pregnancies, as well as physiological weight gain distribution in women with normal BMI.^186 , 187^

**Chart 7.1:** Distribution of physiological weight gain in women with normal BMI (>18 and <25kg/m^2^) and recommendation for gestational weight gain adjusted to BMI according to Institute of Medicine, Lifecycle Project-Maternal Obesity and Childhood Outcomes Study Group, and FEBRASGO^186 , 187^

Weight gain	BMI(< 18.5 kg/m^2^)	BMI(> 18.5 and < 25 kg/m^2^)	BMI(≥ 25 and < 30 kg/m^2^)	BMI (≥ 30 kg/m^2^)
FEBRASGO	9.7 to 12.2 kg	8.0 to 12 kg	7 to 9 kg	5 to 7 kg
IOM: Single pregnancy Twin pregnancy	12.7 to 18 kg	11.5 to 16 kg 16.8 to 24.5 kg	7 to 11,5 kg 14,1 to 22,7 kg	5 to 9 kg 11,4 to 19,1 kg
Lifecycle Project-Maternal Obesity and Childhood Outcomes Study Group	14 to < 16 kg	10 to < 18	2 to < 16 kg	BMI from 30 to 34.9 kg/m^2^: 2 to < 6 kg BMI from 35 to 39.9 kg/m^2^: 0 to < 4 kg BMI > 40 kg/m^2^: 0 to < 5kg
Weight distribution (kg)		Fetus: 3.2 to 3.6 kg Maternal fat: 2.7 to 3.6 kg Blood volume: 1.4 to 1.8 kg Extravascular fluid volume: 0.9 to 1.4 kg Amniotic fluid: 0.9 kg Breasts: 0.45 to 1.4 kg Uterine hypertrophy: 0.9 kg Placenta: 0.7 kg		

BMI: body mass index; IOM: Institute of Medicine.

#### 7.3.1. Maternal Impact of Excessive Gestational Weight Gain

Almost 50% of women initiate pregnancy with overweight or obesity, and 51% gain weight above the recommended amount. In postpartum, on average, women retain 0.5-3 kg per pregnancy. Preconception overweight and obesity are associated with reduced fertility and a delay in conception. In addition, they increase the risk of maternal mortality and complications, such as excessive GWG, GD, hypertensive disorders of pregnancy, emergency cesarean delivery, congenital diseases, preterm delivery, fetal death, and future risk of DM and CVD. Studies have shown that pregnant women with overweight or obesity tend to have elevated levels of leptin, total cholesterol, LDL-c, very-low-density lipoprotein cholesterol (VLDL-c), triglycerides, fasting glycemia, and insulin, in addition to higher HOMA-IR (homeostasis model assessment of insulin resistance) and diastolic BP in the third trimester.^188^

#### 7.3.2. Fetal Impact of Excessive Gestational Weight Gain

Maternal obesity is associated with increased risk of fetal macrosomia, congenital malformations, and intrauterine death. In addition, intrauterine exposure to a hyperglycemic environment can predispose the newborn to develop obesity and MS in adulthood, because of changes in fetal metabolic programming.^189^

In a study with 16 million births in the USA, pregnant women with healthy BMI had lower infant morbidity and mortality rates, while those with class 3 obesity had higher risks of those adverse outcomes, independently of GWG.^190^ Figure 7.4 shows the recommendations for GWG monitoring.

**Figure 7.4:** Recommendations for gestational weight gain monitoring. BMI: body mass index; HOMA-IR: homeostasis model assessment of insulin resistance.

### 7.4. Gestational Dysglycemia

Hyperglycemia is the most common metabolic change in pregnancy, driven by the obesity epidemic, higher prevalence of type 1 diabetes mellitus (T1DM) and T2DM in women of reproductive age, and late pregnancies.^191 , 192^In pregnancy, there is a reduction in fasting glycemia because of fetal and placental glucose uptake, as well as mild postprandial hyperglycemia due to diabetogenic placental hormones, even in women with normal metabolism.^192^

Dysglycemia in pregnancy is classified as pregestational diabetes, diagnosed in pregnancy or GD. Prenatal consultations offer the opportunity to diagnose previously unidentified DM, based on established criteria of glycemia, oral glucose tolerance test (OGTT), or HbA1c.^191 , 192^The most prevalent form of gestational hyperglycemia is GD, which affects up to 25% of the pregnancies and is defined as intolerance to carbohydrates initiated in pregnancy, without meeting the diagnostic criteria for DM outside this period.^191 , 192^( Chart 7.2 ).

**Chart 7.2:** Diagnostic criteria for gestational diabetes and pregestational diabetes

	Normal	GD	DM
Fasting glycemia	< 92 mg/dl	≥ 92 mg/dl and < 126 mg/dl	≥ 126 mg/dL
Random glycemia			≥ 200 mg/dL
OGTT between the 24th and the 28th weeks		Fasting: ≥ 92 and < 126 mg/dL 1^st^hour ≥ 180 mg/dL 2^nd^hour ≥ 153 and < 200 mg/dL	2^nd^hour ≥ 200 mg/dL
HbA1c	< 5.7%		≥ 6.5%

DM: diabetes mellitus; GD: gestational diabetes; HbA1c: glycated hemoglobin; OGTT: oral glucose tolerance test

Although less sensitive to diagnose GD, HbA1c can identify increased risk when ≥ 5.7% in the first trimester, indicating the need for early screening.^192^HbA1c is a reliable test to guide women with diabetes who want to get pregnant, and measures < 6% reduce the risk of fetal malformations.^193^The major RFs for GD are advanced maternal age, overweight, obesity, family history of DM, IR, hypertriglyceridemia, hypertension, excessive GWG, and previous GD.^191^

Gestational diabetes increases the risk of obstetric and neonatal complications, such as abortion, preeclampsia, prematurity, macrosomia, fetal anomalies and death, hypoglycemia, and neonatal respiratory distress. Adequate intervention improves outcomes.^191 , 192^Its effects last throughout maternal life, increasing by four-fold the risk of T2DM and by two-fold the risk of cardiovascular events already in the first postpartum decade, and that increase persists throughout life and seems to be independent of T2DM development.^194^Regarding the fetus, there is higher risk of MS, obesity, DM, and SAH over the course of life.^191 - 193^

The initial treatment is based on nutritional guidance and physical activity practice. If glycemia is not controlled in up to 14 days, pharmacological therapy should be initiated, and that is mandatory for pregnant women with pregestational T1DM or T2DM.^202 , 205^In such cases, aspirin (100–150 mg/day) is recommended from the 12th-16th week on to prevent preeclampsia.^192^Insulin is the drug of choice due to its efficacy, safety, and low placental transfer.^192 , 195^

Glycemic self-monitoring, especially in the post-prandial period, is fundamental for therapeutic adjustment, hypoglycemia prevention, and lower risk of preeclampsia.^192 , 195^Glycemic goals include pre-prandial values of 65-95 mg/dL, 1st post-prandial hour < 140 mg/dL, and 2^nd^post-prandial hour < 120 mg/dL.^195^

Prevention of GD should be initiated before conception, with weight control, healthy diet, and regular practice of physical activity.

### 7.5. Gestational dyslipidemia

During pregnancy, the maternal organism undergoes adaptations to adjust fetal growth and development. Thus, the increase in lipid and lipoprotein levels in this phase is important. These changes are stimulated by placental lactogen hormone, estrogen, and progesterone, as well as by elevations in leptin and insulin levels.^196^Cholesterol is crucial for embryonic and fetal development, because it is an essential component of cell membranes and responsible for several intracellular signaling functions. In addition, this cholesterol increase is necessary to meet the elevated demand for maternal and placental steroids, which accumulate in the maternal body since the 7th week, peaking in the second and third trimesters. By the end of pregnancy, stored lipids serve as a reservoir for the synthesis of fatty acids in the placenta.^196^The adaptations of lipids to pregnancy consist in an increase in total cholesterol and LDL-c by approximately 30-50% and in HDL-c, by 20-40%.^197^Triglycerides increase more significantly, reaching 2 to 4 times their pregestational levels in the third trimester, because, along with glucose, they are one of the major sources of fetal energy. LDL-c changes not only its levels, but increases small and dense, more atherogenic, particles, generating higher impact on patients with familial hypercholesterolemia. This unfavorable effect is attenuated by elevated levels of HDL-c and apolipoprotein A-I, which peak during the second trimester and can offer protection against atherogenic lipid fractions.^196^

In pregnancy, the levels of Lp(a) almost double and the mechanisms responsible for that are not clearly understood. The hypotheses for that increase include estrogen influence in Lp(a) synthesis and clearance, Lp(a) action as acute phase protein in endothelial injury, and Lp(a) possible role in placental development.^198^

Lipid levels in pregnancy and the magnitude of their changes during pregnancy are influenced by several factors, such as pregestational lipid levels, BMI, age, diet, and ethnicity.

Breast-feeding improves the lipid profile, with a higher reduction in LDL-c and triglycerides than in HDL-c.^196 , 199^

#### 7.5.1. Impact of Dyslipidemia in Pregnancy

Lipid assessment in the first trimester can provide valuable information regarding short- and long-term results for the mother and newborn, in addition to identifying specific risk groups.^197^Dyslipidemia (elevation of apolipoprotein B, total cholesterol, LDL-c, and triglycerides), especially at the beginning of pregnancy, is associated with adverse outcomes for the mother and newborn. It has been shown that an atherogenic lipid profile increases the risk of endothelial injury via oxidative stress mechanisms in arterial wall. Maternal risks include preterm delivery, preeclampsia, SAH, GD, MS, and unfavorable lipid profiles. The risks for newborns include premature birth, macrosomia, development of pre-atherosclerotic lesions, and unfavorable lipid profiles. Women with preterm deliveries have a two-fold increased risk of developing CVD later. The placenta shows changes, such as atherosclerosis, infarction of villi, and thrombosis. Addressing lipid abnormalities in pregnancy might help reduce the risk of prematurity.^200^

In addition, the lipid profile in pregnancy has been related to the development of MS years later and can be used as an early marker of a woman's cardiovascular health, as described in chapter 5. Moreover, it can predict the lipid profile of children and be used as a predictor of children's health in the long run. Monitoring this phase can be a window of opportunity to initiate an early intervention and possibly reduce future CVR. Increased lipid levels have been associated with fatty streaks in the aorta and rapid progression of atherosclerosis in childhood.^201^

Elevation in Lp(a), an inflammatory protein, can negatively influence gestational outcomes, increasing the risk of complications, such as preeclampsia, DM, preterm delivery, and low birth weight. These conditions represent short-term risk of maternal and fetal morbidity and mortality, and are associated with increased CVR in the long run, such as MI, stroke, and HF.^198^

Therefore, lipid screening in the first trimester can provide valuable information about short- and long-term outcomes of the mother and newborn, in addition to identifying specific risk groups.

#### 7.5.2. Impact of Pregnancy on Patients with Familial Hypercholesterolemia

Familial hypercholesterolemia is an autosomal semidominant condition, caused by mutations in genes related to lipid metabolism, resulting in elevated LDL-c levels and risk of early IHD.

Women with heterozygous familial hypercholesterolemia have total cholesterol and LDL-c levels approximately twice higher than those without the condition. A Norwegian study has reported that, although the relative increase in total cholesterol and LDL-c between the 17th and 36th gestational weeks was similar in the groups, the absolute increase was significantly higher in women with familial hypercholesterolemia. Triglycerides were also more elevated in that group although still within the normal range and with a similar relative elevation pattern. HDL-c levels, however, remained unchanged in both groups. Despite speculation about possible epigenetic effects of fetal exposure to high cholesterol levels, the mechanisms involved remain uncertain because of conflicting literature data.^196^

Despite the association of gestational dyslipidemia and adverse maternal-fetal outcomes, the European Society of Cardiology (ESC) guidelines only briefly mention the use of statins and discourage it in women of reproductive age who want to get pregnant. In addition, the United States Centers for Disease Control and Prevention (CDC) offers no specific guidance. They recommend that lipid-lowering drugs should be avoided during pregnancy and lactation, except in severe cases, such as familial hypercholesterolemia, for which bile acid sequestrants (non-absorbed) or LDL-c apheresis are considered.^196^The scarcity of data about the treatment is mostly due to the systematic exclusion of pregnant women from clinical trials, which limits the knowledge on the safety of lipid-lowering drugs. Metanalyses and systematic reviews about the subject have provided controversial and biased data.^196^

### 7.6. Endometriosis and Cardiovascular Risk

The association of endometriosis with RFs for CVDs, such as SAH and dyslipidemia with atherogenic profile, has been shown, as well as the increased risk for VTE, IHD, HF, and stroke.^202^Endometriosis, an estrogen-dependent chronic inflammatory disease, is the major cause of chronic pelvic pain in young women and one of the major causes of infertility. It is associated with a chronic inflammatory process mediated by substances, such as intercellular adhesion molecule, IL-1 and IL-6, TNF-α, and VEGF, which induce an increase in oxidative stress.^181^A systematic review with 254,929 participants has revealed that the condition is associated with higher risk of IHD (HR 1.50) and cerebrovascular disease (HR 1.17).^203^

The complete spectrum of pathogenesis and pathophysiology of endometriosis is recognized as a multifactorial condition involving hormonal, pro-inflammatory, pro-angiogenic, immunological, and genetic processes. Genetic studies have identified variants associated with complex diseases, such as IHD, enlarging the knowledge of its pathophysiology and suggesting new therapeutic targets, especially in lipid metabolism. In addition to hormonal and inflammatory factors, there is a relevant genetic component, with dysregulation of inflammasome, promoting cell proliferation and chronic inflammation. This inflammatory process contributes to a pro-thrombotic state, favoring atherosclerosis and supporting the hypothesis that endometriosis is a CVRF.^182 , 204^

#### 7.6.1. Risk factors

In endometriosis, there is an elevation in well-known RFs for CVD, such as hypertension and dyslipidemia with major atherogenic profile, in addition to increased risk for VTE, IHD, HF, and stroke.^182^Population studies have shown an association between endometriosis and SAH, with relative risk (RR) of 1.14 for SAH in women with endometriosis and RR of 1.29 for endometriosis in women with SAH, suggesting common inflammatory mediation. Regarding dyslipidemia, data from the *Nurses’ Health Study II* ^205^have shown a 25%-higher risk of hypercholesterolemia in women with endometriosis, in addition to higher prevalence of endometriosis in women with an atherogenic lipid profile. Changes in the metabolism of phospholipids and sphingolipids play a significant role in endometriosis pathophysiology.

Although data on tobacco use, diabetes, and pollution are inconclusive, a recent metanalysis has shown increased risk of 23% for CVD and of 13% for hypertension in women with endometriosis. In addition, association with coronary events (RR 1.62) has been observed, despite the methodological heterogeneity of the studies. Hysterectomy before the age of 50 years, with or without oophorectomy, has been correlated to higher risk of IHD, possibly because of an adverse cardiometabolic profile. In addition, hormonal treatment for endometriosis can negatively impact CVR because of effects on weight and lipid metabolism. Lifestyle and behavioral factors, such as sedentary lifestyle and inadequate diet, contribute to the interrelation between endometriosis and CVD.^206^

Recognizing endometriosis as a potential RF for CVD is crucial, and so are the implementation of strategies for lifestyle changes and early intervention to prevent and minimize CVR in such women.^182 , 206^ Figure 7.5 depicts inflammatory markers and CVDs associated with endometriosis.

**Figure 7.5:** Inflammatory markers and cardiovascular diseases associated with endometriosis. HF: heart failure; ICAM: intercellular adhesion molecule; IHD: ischemic heart disease; IL-1: interleukin-1; IL-6: interleukin-6; ROS: reactive oxygen species; SAH: systemic arterial hypertension; TNF: tumor necrosis factor; VEGF: vascular endothelial growth factor; VTE: venous thromboembolism.

### 7.7. Psoriasis

Psoriasis is traditionally known to cause inflammatory plaques on the skin. However, there is increasing evidence that it is a systemic chronic inflammatory disease associated with cardiovascular comorbidities, such as obesity, MS, and CVD, cerebrovascular diseases, cardiac arrhythmias, sleep apnea, etc. Although its pathophysiology has not been totally clarified, psoriasis’ systemic inflammation is believed to relate to a pro-inflammatory state with the participation of cytokines, such as TNF-α, IL-6, leptin, and other adipokines.

The study of US female nurses has shown an increased risk of developing DM (RR 1.63) and SAH (RR 1.17) among female nurses with psoriasis.^207^Recent systematic review and metanalysis have shown the association of psoriasis with IHD.^208^

### 7.8. Hypertensive Disorders of Pregnancy and Endothelial Dysfunction after Menopause

Hypertensive disorders of pregnancy affect up to 10% of pregnancies and represent important predictors of CVR over the course of life, being identified as exclusive RFs of the female sex. Increasing evidence has suggested that women with history of hypertensive disorders of pregnancy have persistent changes in endothelial function even decades after pregnancy, which contributes to higher risk of CVD in postmenopause.^219^Other sessions of this position statement have already addressed the large impact on future cardiometabolic health of women with hypertensive disorders of pregnancy.

Endothelial dysfunction, characterized by a reduction in NO bioavailability and increased inflammatory and pro-thrombotic markers, is an early event in atherogenesis. Longitudinal studies have shown that women with previous preeclampsia had elevated endothelin-1 levels, endothelium-dependent vasodilation dysfunction, and carotid intimal thickening years after delivery. This impact seems to increase after menopause, with worsening of the cardiometabolic profile.^210 , 211^

In addition, biomarkers of endothelial activation, such as VCAM-1 (vascular cell adhesion molecule) and E-selectin, remain elevated in late postpartum, suggesting low-grade chronic inflammation. Early interruption of estrogen exposure, common in women with preterm delivery due to preeclampsia, may also lead to an earlier loss of hormonally-mediated vasculogenic protection. Inclusion of obstetric history in CVR stratification is essential to the elaboration of individualized preventive strategies, especially in postmenopause.^212^

Thus, preeclampsia should not be considered only an isolated gestational complication, but rather a marker of female vascular susceptibility over the course of life.^213^The recent ESC guideline indicates that sex-specific factors are potential reclassifiers of CVR to a higher risk category. Hypertensive disorders of pregnancy should be considered in the individualized CVR stratification, although only a few disorders have shown to improve risk prediction or discrimination beyond traditional factors.^214^

For such women, it is essential to intensify, as early as from postpartum on, the approach using the eight pillars of cardiovascular prevention, such as control of BP, glycemia, dyslipidemia, and weight, physical activity, sleep quality, tobacco use cessation and healthy eating.^213 , 214^

### 7.9. Metabolic Changes in Postpartum

In recent decades, there has been a significant advance in protocols for pregnancy and delivery care. However, the postpartum period continues to be neglected, despite its critical importance to long-term maternal health. In that period, women face significant changes in their reproductive system recovery, as well as in metabolic, endocrine, and nutritional aspects.

One of the most evident changes is body weight variation. On average, weight loss occurs at a rate of 0.6-0.8 kg/month in the first six months after childbirth.^215^However, some women retain or even gain weight, which can lead to obesity and increase the risk of cardiometabolic complications. Weight retention after childbirth has significant implications for cardiovascular health, as shown in Figure 7.4. ^216^

#### 7.9.1. Cardiometabolic Risk Factors in Postpartum

Recent studies have shown that factors such as race/ethnicity, socioeconomic level, and GD history, are strong predictors of cardiometabolic risk in postpartum.^217 , 218^To minimize these risks, some strategies should be considered:

1. **Lactation** : breastfeeding is a protective factor against maternal obesity, improving lipid metabolism via hepatic lipolysis and combined action of prolactin and insulin;^219^
2. **Diet** : postpartum diet should focus on calorie reduction and nutritional quality. Addition of 500 kcal/day is recommended in the first six months, and 400 kcal/day in subsequent months to ensure adequate metabolic balance;^220^
3. **Physical activity** : physical exercise improves cardiorespiratory fitness, preserves lean mass, and helps lose weight, reducing metabolic risk;^221^
4. **Sleep** : sleep deprivation (<5 hours/day) has a negative impact on glucose metabolism and favors obesity and IR.^222^

#### 7.9.2. Impact of gestational diabetes and dyslipidemia in postpartum

Excessive weight retention in postpartum is associated with a higher risk of dyslipidemia and IR. Women with history of GD are more likely to have metabolic disorders in postpartum, such as hyperinsulinemia and hypertriglyceridemia. These effects seem to be influenced by BMI and the gestational age at which the metabolic changes appeared. Studies have shown that, even years after childbirth, women with GD maintain elevated total cholesterol and LDL-C levels, independently of other traditional CVRFs.^223^These women tend to have elevated levels of triglycerides and LDL-c, independently of BMI, up to months after giving birth. Hyperglycemia and IR in pregnancy contribute to dyslipidemia persistence in postpartum, impacting cardiovascular health in the long run.^224 , 225^

#### 7.9.3. Prolactin and Metabolism in Postpartum

In addition to its function in lactation, prolactin plays a relevant role in maternal metabolism. Although the relation of prolactin levels and GD has not been totally clarified, evidence suggests that, in postpartum, especially during lactation, elevated prolactin levels are associated with lower circulating insulin levels, higher function of beta cells, and increased insulin sensitivity.^226^

Maternal overweight and obesity, excessive GWG, and weight retention in postpartum are well established RFs for cardiometabolic complications. Therefore, the postpartum period should be seen as a window of opportunity for preventive interventions, with special attention to balanced and quality diet, regular physical exercise, body weight control, adequate sleep, and breastfeeding. The adoption of such strategies can contribute to preserve maternal cardiovascular health and reduce the risk for cardiometabolic disorders throughout life.

## 8. Cardiometabolic Health in Menopause Transition, Menopause and Postmenopause

Cardiometabolic disorders, such as T2DM and CVDs with their associated RFs, such as obesity, IR, hypertension, and dyslipidemia, are the major causes of mortality and burden of diseases in both sexes.^73 , 109^Women and men, however, experience different trajectories of cardiometabolic risk throughout life influenced by hormonal fluctuations. Women's reproductive phase is a window of opportunity for prevention and approach of CVRFs and cardiometabolic disorders that are magnified in menopause transition, during menopause, and in postmenopause.^73 , 109^Reduction in endogenous estradiol levels during menopause transition has been associated with an increased risk of cardiometabolic health issues, such as abdominal adiposity, dyslipidemia, T2DM, and hypertension.^73 , 109^

Usually, the last menstrual cycle occurs between 45 years and 55 years of age in 90% of women, and, in parallel, the CVR increases in the fifth decade of life, 10 years later than in the male sex. The age of menopause onset seems to be a marker of not only reproductive ageing with the decline in estrogen levels, but also of an increase in cardiometabolic complications.^73 , 109^

Menopause before age 40 years is considered premature, currently named premature ovarian failure, and has a prevalence of 1%. When menopause occurs between 40 years and 45 years, it is classified as early menopause and affects 7.3% of women. Evidence has shown that women with natural or surgical premature menopause are more likely to develop CVD.^73 , 109^( Figure 8.1 ) In the study by Honigberg *et al* ., assessing 144,260 women, natural premature menopause was independently associated with aortic stenosis, VTE, ischemic stroke, IHD, and atrial fibrillation. Surgical menopause, however, has been related mainly to mitral regurgitation, pulmonary thromboembolism, HF, and IHD. Menopause at a younger age remained independently associated with time until the first diagnosis of incident CVD (HR, 1.02/year of earlier age of menopause onset [95%CI, 1.01-1.03]; *p* < 0.001).^157^

**Figure 8.1:** Concept of menopause and increased cardiovascular risk. CVR: cardiovascular risk; POF: premature ovarian failure.

In addition, women with premature and early menopause have a substantially higher risk of a non-fatal cardiovascular event, especially IHD and stroke, before the age of 60 years, possibly associated with low estrogens levels that are related to subclinical atherosclerosis progression. A study with 301,438 women in five countries has reported a 30%-higher risk of CVD in women with early menopause as compared to those experiencing menopause at the age of 50-51 years.^227 , 228^

The literature has reported a statistically significant association of early age of natural menopause onset with risk of mortality due to general and cardiovascular causes, while more advanced age of menopausal onset results in longer life expectancy, higher bone mineral density, and lower risk of fractures.^229 , 230^

### 8.1. Severity of Symptoms and their Implications for Cardiovascular Health

The severity of the clinical manifestations of menopause transition are also related to women's cardiovascular health. They are mainly characterized by VMS, night sweats, neurological complaints, such as sleep, mood, cognition, and memory disorders, in addition to changes in genitourinary and sexual functions. They impair women's quality of life, productivity, and physical and mental health.^73 , 109^

Vasomotor symptoms are the major complaints, affecting 80% of women during menopause transition, peaking around the final menstrual cycle.^231^They have been associated with BP elevation and increased risk of CVD, such as stroke and IHD. The major causing factors include autonomic dysregulation with exacerbation of the sympathetic nervous system, sleep interruption, endothelial dysfunction, and more severe subclinical atherosclerosis.^232 , 233^Evidence has suggested the association of early VMS onset, duration, and severity with risk for CVD.^232^Sleep disorders are reported by 40% to 60% of menopausal women and interfere negatively with their quality of life, as well as mental and physical health.^234^

Identifying premature ovarian failure and early or premature menopause, as well as severity of symptoms, is fundamental to recognize the increased CVR in middle-aged women. This approach enables assessing the therapeutic window for hormonal replacement and intensification of lifestyle changes, optimizing the control of other important factors to reduce cardiovascular events, such as BP, cholesterol, glycemia, body weight, quality of sleep, mental stress, cardiorespiratory fitness, and tobacco use cessation.

### 8.2. Cardiovascular Risk Factors and Menopause

Hypertension is one of the major CVRFs in women, in whom the association with CVD risk is higher than in men.^235^Globally, the rate of BP control is estimated to be 23% in women with SAH.^236^With ageing and after menopause, SAH prevalence increases and the BP control rate decreases.^235^Some factors, such as unfavorable lifestyle and diet, obesity, and ageing, as well as characteristics related to menarche, reproduction, and menopause contribute to increase BP.

Concomitantly with menopause, there is a decrease in the circulating levels of estrogen and androgens. In addition, there are renin-angiotensin-aldosterone system dysregulation, sympathetic activation, endothelial dysfunction, inflammation, and higher sodium sensitivity.^237 , 328^This leads to a rapid and accentuated increase in BP^239^and high SAH prevalence in postmenopause, with consequent elevation in the risk of cardiovascular events, such as AMI, HFpEF, stroke, cognitive deficit, and peripheral artery disease, usually with systolic BP 10 mm Hg lower than in men.^240^Moreover, women with SAH more frequently have CKD, left ventricular hypertrophy, and coronary microcirculation dysfunction.^235^

In managing SAH, BP monitoring outside the medical office is important because of the high incidence of white coat hypertension and masked hypertension. Of nonpharmacological measures, salt restriction stands out. Physical exercise reduces BP and arterial stiffness.^241^Regarding pharmacological treatment, there is no specific recommendation regarding classes of antihypertensive drugs for menopausal women. Adverse events can be more frequent in women, probably related to pharmacokinetic and pharmacodynamic properties of antihypertensive drugs.^235 , 238^

Regarding lipids, studies have shown that, in postmenopausal women, total cholesterol and LDL-c levels are higher, and dyslipidemia treatment and control rates are lower than those in men of the same age.^242 , 243^HDL-c levels are lower.^231^Studies have shown that the antiatherogenic function of HDL-c can be impaired and, in such case, elevated HDL-c levels can be associated with atherosclerosis.^244^In addition, Lp(a) concentration tends to increase during menopause, and elevated Lp(a) levels are more common in women than in men after the age of 50 years, increasing the risk of CVD.^235 , 243^

These particularities in BP behavior and in lipid disorders suggest that current guidelines for the management of such conditions might be inadequate to meet women's specific needs. Thus, targeted interventions, such as non-medicamentous and medicamentous measures, are essential to improve menopausal women's cardiovascular care. It is worth noting that women are under-represented in clinical trials, which determines scarcity of solid scientific information on women.^233 , 235 , 238 , 244^

In menopause, the decline in ovarian function results in a significant impact on not only BP, but also other cardiometabolic RFs, such as weight gain, central adiposity, atherogenic lipid profile, and increased glycemia associated with IR, a significantly threatening set of factors to women's cardiovascular health.^235 , 238 , 243^

Factors, such as race/ethnicity, reproductive factors, body composition, lifestyle, genetics, and premenopausal cardiovascular health, can affect natural menopause and are associated with cardiometabolic risk. In a cohort study including 3639 Dutch women with natural menopause, those who had experienced early natural menopause had a 2.4-higher risk of T2DM as compared to those with late menopause onset, and lived less and fewer years without T2DM as compared to women who had experienced normal or late menopause.^245^

However, another study involving 177,131 women from four different countries has shown that those with a cardiovascular event before the age of 35 years, as compared to women without CVD, had a twice higher risk of early menopause, raising the hypothesis that the associations can be bidirectional. Thus, a worse premenopausal cardiovascular health profile can influence natural menopause onset.^227^

Menopause is accompanied by several metabolic adaptations related to IR, increased total body fat mass, and accumulation of central abdominal fat, predisposing women to develop T2DM. Metabolic syndrome has high prevalence in menopausal women, indicating loss of estrogen protection in metabolic and cardiovascular health. In addition, early menopause has been related to increased risk of T2DM.^73 , 109^

Moreover, high estrogen exposure in premenopausal women, such as pregnancy, has been associated with adverse metabolic changes (hyperglycemia or increased BP) that could impact the risk of developing T2DM and SAH later in life.^246^

Environmental factors and those related to lifestyle, such as diet, alcohol consumption, physical activity, overweight and obesity, tobacco use, and exposure to environmental toxins, as well as sociodemographic, psychological, and social cognitive factors, are associated with the risk of both early natural menopause and cardiometabolic disease. Environmental factors and those related to lifestyle can trigger underlying mechanisms of cardiometabolic disease associated with menopause, including its influence on DNA methylation and gene expression, induction of low-grade inflammation and oxidative stress, probably interfering with hormone-related signaling in menopause.^247^

### 8.3. Menopausal Hormone Therapy, Hormone Implants, and Cardiometabolic Impact

Menopausal hormone therapy is used as the most effective intervention to relieve menopausal symptoms. It offers significant benefits, although it cannot be used for primary or secondary prevention of cardiometabolic disease. With the administration of estrogen and, in some cases, progesterone, MHT is aimed at relieving symptoms and improving quality of life. There are several MHT administration forms, such as pills, patches, gels, and spray. Factors, such as MHT onset and time, duration, dose, and administration route, determine its benefits and drawbacks. The choice of MHT type depends on several factors, such as associated risks, indications, and contraindications.^73 , 109^

Beginning MHT within the first ten years from menopause onset leads to higher safety in relation to the risk of cardiometabolic disorders, resulting in lower absolute risk of VTE, CVD, and stroke in the first years of menopause. MHT should be taken for the shortest period of time needed and at the lowest dose, and transdermal route has fewer metabolic effects. Oral MHT in women with baseline thromboembolic risk can increase the risk for VTE and stroke on a dose-dependent way; however, transdermal estradiol (only or combined with micronized progesterone) is considered safer. The vaginal route can be used, indicated only for the treatment of genitourinary symptoms.^73 , 109^

Randomized and controlled clinical trials have suggested that MHT could reduce IR and T2DM incidence, improve glucose metabolism, and increase the glucose hepatic production suppression, and oral preparations have a higher potential to improve glucose metabolism and reduce the risk of developing T2DM.^155 , 248^Usually, oral 17β-estradiol is preferred in women with T2DM, because that route has more beneficial effects in glucose metabolism. Oral estrogens are also suggested to women with low risk of CVD in perimenopause or menopause of recent onset. Oral micronized progesterone or dydrogesterone and oral or transdermal norethisterone are the most used progestagens for postmenopausal women with T2DM and intact uterus. The administration of MHT to postmenopausal women with T2DM can be safe and effective if the therapeutic regimen is properly selected. However, MHT is not recommended for primary prevention of T2DM or cardiometabolic disorders.^73 , 109 , 117^

Testosterone replacement therapy is not indicated to improve cardiometabolic or musculoskeletal health, VMS, or mood changes. Sufficient solid studies on the impact of androgens on cardiometabolic health are not available. In addition, there are few clinical studies on hormone implants for MHT, and most of them address testosterone implants. These studies have small case series and/or low level of evidence (retrospective or observational studies). This impedes the understanding of the cardiometabolic effects of such implants, as well as of their risks for breast and endometrial cancers; therefore, such implants are not recommended for MHT.^73,109,1173^

### 8.4. Stratification of Cardiometabolic Risk in Menopause

In menopause and postmenopause, there is an abrupt drop in estrogen levels, an essential hormone for female cardiovascular protection. Some of the changes resulting from this hormone deficiency are as follows: increase in LDL-c, total cholesterol, apolipoprotein B, and triglyceride levels; dysfunctional HDL-c, whose ability to promote cholesterol efflux is reduced, leading to partial loss of its cardiovascular protection; glucose intolerance and increased risk for T2DM; accumulation of visceral fat and ectopic fat deposits in the liver and heart, exacerbating subclinical inflammation; and simultaneous increase of abdominal obesity, hypertension, dyslipidemia, and hyperglycemia.^2 , 249^

In the United States, between 2013 and 2017, there was a 7% increase in CVD among middle-aged women, attributed mainly to the increase in obesity and prevalence of cardiometabolic RFs. It is worth noting that women with history of T2DM, SAH, and tobacco use have significantly higher relative risk for CVDs as compared to men with the same CVRFs. For example, T2DM increases CVR by 3 to 7 times in women as compared to 2 to 3 times in men, possibly due to higher BMI, higher systemic inflammation, more deficient glycemic control, and higher burden of RFs at the time of female diagnosis.^2 , 250^

In addition, estrogen loss causes endothelial dysfunction and impairment of vascular integrity, resulting in: reduced flow-mediated dilation; increased arterial stiffness, identified as increased pulse-wave velocity; carotid intimal thickening, which is a marker of subclinical atherosclerosis; increased coronary artery calcium score (CAC); and presence of breast arterial calcification. The CAC obtained on computed tomography without contrast material is currently recognized as a strong marker of atherosclerotic burden and important predictor of risk for IHD, reclassifying the CVR obtained by use of traditional RFs. Studies have shown that, although women have fewer coronary calcifications than men of same age, the presence or increase of CAC is associated with higher relative risk in women. In addition, even in women considered of "low risk" according to Framingham score, a detectable CAC (>0) increases in up to five times the CVR. Breast arterial calcification incidentally detected on mammography also emerges as an independent risk marker of CVD, with 2.4-fold increased risk. In several cases, its detection justifies intensification of RF screening and complementary assessment with CAC.^208 , 209^

In addition, biomarkers have a relevant role in the cardiometabolic risk assessment of women in menopause transition, menopause, and postmenopause. The following biomarkers stand out: us-CRP, an independent predictor of CVR (usually >3 mg/L or 0.3 mg/dL) even in women with normal lipid levels, integrating Reynolds Risk Score; elevated serum levels of triglycerides (≥175 mg/dL), Lp(a) (≥50 mg/dL or ≥125 nmol/L) and apolipoprotein B (≥130 mg/dL), which are worsening factors; and high-sensitivity troponins and natriuretic peptides, which have prognostic value for future cardiovascular events, although not yet routinely recommended by the ACC/AHA guidelines for population screening of asymptomatic patients.^2 , 117 , 249^

Before initiating MHT, it is essential to consider the patient's total CVR. The initial assessment includes: complete lipid profile (total cholesterol, LDL-c, HDL-c, and triglycerides), fasting glycemia, HbA1c, and mammography.^226^In the absence of specific scores for perimenopause and postmenopause, traditional scores are used and can be refined by the identification of enhancing RFs and markers of subclinical atherosclerosis. Patients with T2DM, CKD, familial hypercholesterolemia, or severe SAH are automatically considered at high or very high risk.^209 , 250^This initial assessment using the CVR score is important to define if MHT can be prescribed and its best administration route, because, in cases of moderate CVR, the transdermal route should be preferentially chosen.^226^

Factors, such as age of menopause onset, stress, anxiety, depression, and quality of sleep, need to be considered for CVR reclassification, although their measurement can be hindered by menopausal symptoms.^109 , 226^

In addition, a sedentary lifestyle in postmenopause leads to worse physical fitness and poorer cardiometabolic control, in addition to higher incidence of fractures and mortality. Tobacco use increases the risk of early menopause and the likelihood of CVD, stroke, osteoporosis, T2DM, and all-cause mortality.^109 , 226^

Stratification of CVR should be performed, followed by dietary and lifestyle counseling. The management of cardiometabolic RFs in menopause should always be individualized, with focus on hypertension, T2DM, and dyslipidemia. When prescribed to control menopausal symptoms or prevent osteoporosis, MHT can also have a beneficial, even though indirect, effect on cardiometabolic RFs.^109 , 226^

## 9. Cardiometabolic Disorders in Women

The incidence of T2DM and obesity has increased considerably in past decades. Projections indicate that there will be more than 600 million individuals with T2DM in 2045.^251^Obesity and T2DM are associated with several other cardiometabolic disorders. Although both sexes are affected by obesity and T2DM, some studies have indicated differences between men and women regarding the prevalence, diagnosis, treatment, and complications of obesity and T2DM. Evidence available on those differences does not imply sex-specific therapeutic and diagnostic recommendations. However, adequate understanding of those differences is crucial for women's clinical management.^252 , 253^

### 9.1. Obesity and Metabolic Syndrome

Obesity is the direct cause of or contributes to the development of several clinical conditions, such as T2DM, MASLD, sleep apnea, articular diseases, several types of cancer, SAH, and HF, in addition to increasing the risk of cardiovascular mortality.^254^The increase in cardiovascular mortality is independently associated with obesity, even in individuals without other metabolic changes.^255^Body weight is maintained by a balance between energy intake and energy expenditure,^256 , 257^and, when there is excessive energy, it is stored as fat in adipose cells, usually in the subcutaneous tissue. There are factors that limit the physiological storage of fat, which then begins to accumulate ectopically in other tissues, such as liver, pancreas, kidneys, muscles, and epicardium.^258^Obesity has a strong genetic burden^259 , 260^and is influenced by life habits; its prevalence, however, has been increasing in women and men across the world.^261^In Brazil, VIGITEL data from 2023 indicate a prevalence of obesity of 24.8% in women and 23.8% in men,^262^and, when overweight individuals are considered, 59.6% of women and 63.4% of men have that condition. ( Figure 9.1 ).

**Figure 9.1:** Obesity and its cardiometabolic consequences in women.

Women usually have a higher percentage of body fat and tendency towards fat accumulation in the subcutaneous tissue of lower limbs, while men have higher visceral fat amounts.^263^These differences in body fat distribution are associated with sex hormones and vary throughout a woman's reproductive life. Thus, after menopause, women experience body fat redistribution, with visceral fat increase.^264^The CVR accompanies these differences in hormones and body composition, and women's classic cardiovascular protection during menacme disappears in menopause.^265 , 266^In addition, gestational weight gain and its maintenance in postpartum are important RFs for obesity.^267^The POS is associated with the presence of obesity, increased IR, and other MS components.

The World Health Organization's definition of obesity takes into consideration the BMI, independently of gender, and is subdivided into grade 1 (BMI: 30.0-34.99 kg/m²), grade 2 (BMI: 35.0-39.99 kg/m²), and grade 3 (BMI over 40.0 kg/m²). Recently, a new classification for obesity diagnosis and staging has been proposed,^268^dividing it in pre-clinical and clinical and considering BMI an inadequate method for obesity diagnosis alone. However, this new classification has not been totally accepted by scientific societies. To our knowledge, BMI is adequate for the diagnosis of obesity in individuals with BMI over 30 kgm/m². For those with lower BMI (below 30 or even below 25 kg/m²), however, excessive adiposity and its metabolic consequences can occur. In such cases, it is better to use other tools for the identification of excessive fat, such as simple measurements of waist and waist-to-hip and waist-to-height ratios, as described in chapter 5. Waist measurements vary between women and men, 88 cm being the cut-off point of waist for Brazilian women. The cut-off point of WHR is 0.85 for women, while 0.5 is the cut-off point of WHtR for men and women.^269^Another more accurate way to measure adiposity is body composition assessment using bioimpedance or DEXA (dual-energy X-ray absorptiometry). However, further studies confirming that its systematized use changes management and has measurable benefits to the management of patients with obesity are required. Anthropometric measures addressed in chapter 5 are important to establish the diagnosis of excessive body adiposity and to assess the disease's severity, in addition to being parameters of response to treatment.

### 9.2. Type 2 Diabetes Mellitus

There are differences between the sexes regarding T2DM diagnosis and epidemiology, and they can reflect biological, social, and behavioral factors. Usually, men are diagnosed earlier and at lower BMI values, while women tend to develop the disease later, often after menopause, when the hormonal protection of estrogens decreases.^270 - 277^In addition, there is evidence that women can remain longer with underdiagnosed hyperglycemia because of different patterns of symptoms and lower sensitivity of some diagnostic criteria, such as fasting glycemia.^278^

The prevalence of T2DM was estimated as 8.8% of the world population in 2017, slightly higher among men than among women (9.1% *versus* 8.4%).^251^Despite that difference, women with T2DM have higher risk of cardiovascular complications, such as cerebrovascular disease, and early mortality, which can be related to inequities in access to diagnosis and treatment.^279^In addition, socioeconomic and cultural factors influence those disparities, affecting women's screening and search for care in different regions. Therefore, understanding the sex-related differences in T2DM diagnosis and prevalence is essential for the implementation of more equitable and effective strategies of prevention and care.

Differences in T2DM pathophysiology involving hormonal, genetic, and metabolic factors influence susceptibility to T2DM and its progression. Usually, women have higher IR, especially in the musculoskeletal tissue, while men tend to have higher visceral fat deposition, which is strongly associated with metabolic dysfunction.^276 , 280^After menopause, the decline in estrogen levels in women contributes to increase central adiposity and worsens insulin sensitivity, changing the inflammatory and lipid profiles.^281^In men, testosterone reduction also associates with IR, although with different mechanisms, involving smaller muscle mass and changes in glucose hepatic metabolism.^282^In addition, differences in gene expression related to glucose transportation, inflammation, and energy metabolism suggest a biological basis for such disparities.^280^

Systematic reviews with metanalysis have shown sex-related heterogeneity in the contribution of each RF for cardiovascular outcomes. While BP, cholesterol, and BMI seem to contribute equivalently to the risk of coronary and cerebrovascular diseases,^278 , 283 , 284^T2DM contributes to higher risk in women.^285^The risk of coronary disease is usually lower in women, but, in the presence of T2DM, such differences disappear.^285^T2DM poses a relative risk of 44% for coronary disease and of 27% for cerebrovascular disease to women; however, women's absolute risk is similar to that of men with T2DM.^286^The higher burden of comorbidities, including RF clustering, as well as hormonal and behavioral issues might contribute to that difference.^287 , 288^

Studies have shown that women less frequently adhere to drug treatment and self-care, which can be related to psychosocial barriers, less social support, and higher prevalence of depression.^276 , 289^There is evidence that women are less likely to achieve the goals of glycemic, BP, and lipid control, even with treatment similar to that of men.^290^In addition, pharmacokinetic and pharmacodynamic differences affect response to antidiabetic drugs.

It is worth noting the psychosocial and behavioral aspects of T2DM in the female sex. The cognitive capacity of all patients with T2DM should be monitored throughout life, and female sex is a RF for cognitive dysfunctions. Depression also is more frequent in individuals with T2DM, with predominance in the female sex.^291 - 293^Women with T2DM have higher incidence of sexual dysfunction, whose occurrence is influenced by both organic (such as autonomic neuropathy) and behavioral factors.

Figure 9.2 summarizes some differences between the sexes regarding T2DM.

**Figure 9.2:** Differences in T2DM between women and men: earlier diagnosis in men and higher cardiovascular and psychosocial risks in women. BMI: body mass index; CVD: cardiovascular disease.

### 9.3. Metabolic Dysfunction-Associated Steatotic Liver Disease

The most prevalent liver disease worldwide is MASLD,^294 - 296^characterized by excessive fat accumulation in hepatocytes. It consists in a spectrum of hepatic manifestations associated with metabolic and cardiovascular disorders, such as obesity and/or unfavorable fat distribution, IR, SAH, dyslipidemia, and T2DM.^297^MASLD is recognized as the hepatic manifestation of MS, being strongly associated with IHD, which is the major cause of mortality in the population with MASLD.^297^

The natural history of MASLD consists of stages, such as steatosis (when there is only excessive fat in the liver, exceeding 5% of the hepatic parenchyma, with minimum inflammation) and steatohepatitis (when there is lobular inflammation and hepatocyte ballooning, with or without fibrosis).^298^Independently of sex, individuals with metabolic dysfunction-associated steatohepatitis (MASH) can progress with different grades of fibrosis, cirrhosis, and complications, such as portal hypertension or hepatocellular carcinoma.^295 , 298^

MASLD is estimated to affect at least 30% of the Western population.^299^In individuals with overweight and obesity, the global prevalence of MASLD is 50.7%,^300^and, in those with T2DM, its estimated prevalence is 65.3%.^299^Although obesity is more common in women, MASLD is more prevalent in men, and the risk of MASLD in women increases after menopause.^301^It is estimated that, in the general population, women have a 19%-lower risk of hepatic steatosis as compared to men; steatohepatitis rates are similar in both sexes, and women have 37% more risk of advanced fibrosis.^302^This higher risk for progression occurs especially in women over the age of 50 years, and it is worth noting the possible participation of sex hormones in the etiopathogenesis of MASLD.^302^

Menopause, history of early menarche,^303^and POS are associated with increased female susceptibility to MASLD.^304^Women have a higher mortality rate due to cirrhosis as compared to men, and MASLD is a major cause of liver transplantation in women without hepatocellular carcinoma.^305^

One justification of the differences in MASLD prevalence over the course of a woman's life is the influence of female sex hormones, particularly estrogen, on hepatic metabolism and on body fat distribution pattern.^301^Activation of estrogen receptor alpha (ERα) in the liver reduces the synthesis, uptake, and storage of triglycerides, and simultaneously favors the catabolism and export of lipids, effects that together protect non-menopausal women against MASLD.^306^In addition, the gynoid fat distribution pattern, characterized by greater gluteofemoral fat concentration and smaller visceral fat accumulation, is the typical fat distribution of women in menacme^301^and is associated with a lower risk of MASLD and MS.^295 , 301^However, after menopause, estrogen decline promotes fat redistribution, favoring centralization and, thus, a more android pattern,^301^known to be associated with MASLD and MS.^295 , 301^

The diagnosis of MASLD consists in the presence of hepatic steatosis associated with at least one criterion for MS, in the absence of secondary causes of steatosis.^295 , 298^Steatosis can be inferred using traditional imaging methods, such as ultrasonography, computed tomography, and magnetic resonance, which can also evidence signs of cirrhosis and portal hypertension. However, it is worth noting that such methods can identify neither MASH nor fibrosis at early stages.^297^Liver biopsy differentiates precisely patients with steatosis from those with MASH, but, because it is an invasive method, it is mainly used in situations in which there are doubts about the liver disease etiology.^295 , 297 , 298^

The diagnosis of MASLD is based on the identification of fibrosis, for which some tools, such as clinical-laboratory risk scores for advanced fibrosis and elastographies, are useful. Of those scores, Fibrosis-4 (FIB-4) stands out, calculated based on age, platelet count, and serum level of aminotransferases; the interpretation of the result does not depend on sex.^295 , 298^When applying the cut-off point of 1.3, from which patients at higher risk of advanced fibrosis are identified, the test has high sensitivity and excellent negative predictive value.^298 ,907, 308^For example, for a woman with FIB-4 ≤ 1.3, the likelihood of advanced fibrosis is very low.

Both ultrasound and magnetic resonance elastographies can estimate liver stiffness and, thus, the presence and amount of fibrosis, including in its initial stages. Transient elastography using the Fibroscan^®^method is the most validated in the literature and recommended by national^295 , 298 , 307 , 308^and international^309^guidelines. MASLD should be actively screened in individuals at higher risk of fat accumulation in the liver and progression to more severe forms of liver disease, aiming at identifying those with significant fibrosis. The groups of risk comprise postmenopausal women, individuals with glucose homeostasis changes (prediabetes or T2DM), excessive weight, MS, and positive family history for cirrhosis and hepatocellular carcinoma. One rational way to perform population screening is to calculate FIB-4 in individuals at risk and perform elastography in those with FIB-4 > 1.3.^295 , 298 , 309^

It is worth noting that the isolated assessment of serum levels of aminotransferases has low accuracy to identify patients with MASH and fibrosis. However, elevated levels of those enzymes indicate the need for screening other liver diseases, especially chronic viral hepatitis and alcoholic hepatopathy (excessive alcohol consumption: >20 g/day for women and >30 g/day for men).^295 , 298 , 310 - 321^

### 9.4. Chronic Kidney Disease

Chronic kidney disease, mainly diabetic kidney disease (DKD), is a worldwide public health problem that affects millions of individuals. We address briefly the potential differences between genders, and women are a population of special interest because of their biological and social characteristics.

Chronic kidney disease is defined as kidney injury or kidney function reduction for over 3 months, which can progress to need for renal replacement therapy. The progression of CKD is frequently insidious. It is estimated that 425 million individuals have DM, and approximately 30% of those with T1DM and 40% of those with T2DM will develop CKD at different stages.^322^The major global cause of renal replacement therapy is DM, followed by SAH,^323^and its incidence has been increasing.^324^North American data have shown higher prevalence of DKD in women (14.8% x 12.6% in men).^325^However, it seems that men are at higher risk for CKD relapse and progression to advanced stages of disease as compared to women.^326 - 328^These data are heterogeneous and can be related to hormonal changes, such as menopause, T2DM duration and age of onset, and the criteria used for diagnosis.^329^Studies have shown higher velocity of estimated glomerular filtration rate loss in elderly and menopausal women, in addition to inconsistency of results related to MHT and renal function changes.^330^Several studies have shown that men are more likely to develop albuminuric DKD as compared to women, in whom the most common form is nonalbuminuric. In a large Italian cohort, moderate to severe albuminuria was present in 29.8% of men as compared to 18.3% of women.^331^

Despite the report of differences between men and women regarding kidney hemodynamics in DM and mechanisms associated, such as higher frequency of glomerular efferent arteriole vasoconstriction in the female sex, their clinical significance remains uncertain.^332 - 337^

Current literature data do not allow the use of different criteria for screening, follow-up, and treatment of DKD between men and women. Thus, the following recommendations should be used for both sexes:^323^

1. The first screening for DKD should be performed with a random urine sample to determine the albumin/creatinine ratio and with estimated glomerular filtration rate determined with serum creatinine, using the 2021 CKD-EPI equation or Schwartz equation for children. In T2DM, screening should begin at the time of diagnosis. In T1DM, screening should begin from puberty onset on or at the age of 10 years in patients with at least 5 years from diagnosis and repeated annually. Every abnormal albumin/creatinine ratio test should be confirmed at least in two out of three samples repeated within three to six months because of daily variability.
2. In individuals with T1DM or T2DM, the HbA1c goal of 6.5-7% should be pursued when estimated glomerular filtration rate >60 mL/min/1.73m² and albumin/creatinine ratio >30 mg/g, to reduce the progression of albuminuria and DKD in the long run. The HbA1c goal of 7-7.9% should be maintained in individuals with T1DM or T2DM when estimated glomerular filtration rate <45 mL/min/1.73m² or if the patient is on dialysis, to prevent excessive mortality.

In conclusion, considering the differences between women and men, as well as the interactions observed with the presence of DM with or without DKD, sex hormones might contribute to sex-related differences in the pathophysiology of DKD beginning and progression.

## 10. Strategies for Addressing Cardiometabolic Disorders in Women

### 10.1. Nonpharmacological Measures

The increasing prevalence of cardiometabolic disorders in women represents one of the major challenges in public health because of the strong link between obesity and CVD. Globally, obesity accounts for 4.7 million deaths annually.^338 , 339^Abdominal fat accumulation contributes to metabolic changes, chronic inflammation, and IR, favoring atherosclerosis, MI, and stroke. According to the Brazilian Ministry of Health and World Health Organization, in Brazil, CVDs are responsible for approximately 28% of female deaths.^340 , 341^

In Brazil, 24.8% of women are obese and 38.7% are overweight, totaling 63% of women above their recommended weight.^262^The ELSA-Brasil study has revealed elevated rates of abdominal obesity among Black (62%) and Mixed-race (59.5%) women, with a general overweight prevalence of 61.8%.^342 , 343^These data show the need for urgent investments and interventions to reverse these pathological processes.

### 10.2. Nutritional Interventions

Nutritional re-education is key for the prevention of cardiometabolic disorders, mainly when tailored to patients’ conditions and habits. Dietary protocols that reduce simple carbohydrates and saturated fats and increase fiber consumption have proven effective in improving glycemia, dyslipidemia, and IR. The Mediterranean diet is widely recognized for its protective effects.^344^

In Brazil, that diet can be adapted to local food, such as olive oil, local nuts, fruits, such as papaya and avocado, freshwater fish, and pulses, such as beans and chickpea. The dietary guide for the Brazilian population emphasizes the consumption of natural food and the respect for regional dietary culture.^345^Adhesion to nutritional plans supervised by specialized professionals results in weight loss and sustained improvement of metabolic parameters, contributing to CVD prevention.^346^A tailor-made approach allows that cultural and regional preferences be respected, because they are relevant factors, considering the wide range of eating habits in Brazil.^109^

### 10.3. Physical Activity

Regular physical activity practice plays an essential complementary role in the treatment of cardiometabolic disorders, mainly during menopause transition and menopause. The combination of aerobic exercises with resistance training promotes significant improvement in body composition, insulin sensitivity, and glycemic control.^347^In addition, it contributes to BP reduction, lipid profile improvement, and systemic inflammation reduction.^348^

Association of individualized nutritional plans with regular physical activity practice enhances the beneficial effects, favoring cardiovascular function and reducing morbidity and mortality.^139^Moreover, physical activity is associated with lower incidence of SAH, dyslipidemia, and T2DM, a reduction in the risk of developing depression and dementia, and improvement of bone mineral density and quality of sleep.^139 , 149^

The current recommendation is moderate aerobic activity for at least 150 minutes per week or vigorous activity for 75 minutes per week associated with muscle resistance exercises at least twice a week.^139 , 350^The prescription should be individualized, considering physical fitness, functional limitations, and context of a woman's life, ensuring adhesion and sustained effects in the long run.^109 , 209^A recent study has shown that, when women and men practiced equivalent doses of physical activity, women derived greater gains in all-cause and cardiovascular mortality reduction as compared to men.^351^

### 10.4. Psychosocial Interventions

Psychosocial stress, such as loneliness, significant losses, and mental disorders, contribute directly to CVR, impairing adhesion to treatment and favoring risk behaviors, such as tobacco use and sedentary lifestyle.^352^Women who participate in integrated psychological support programs have a significant improvement in lifestyle, which results in better clinical outcomes, such as weight reduction and glycemic and lipid profile control.^353^Strategies, such as cognitive-behavioral therapy, are especially effective in such context, and studies have shown their ability to reduce anxiety and depression symptoms, improve quality of life related to cardiovascular health, and increase adhesion to treatment.^353^

Cognitive-behavioral therapy is associated with a lower rate of hospital readmissions and improved self-perception of health in women with CVD.^354^Moreover, a cognitive-behavioral therapy applied to stress management was associated with sustained behavioral changes, higher frequency of physical activity, healthy diet, and smoking cessation.^354^

### 10.5. Tobacco Use and Alcohol Consumption

Tobacco use is an important chronic inflammatory factor, worsened in postmenopausal women.^355^Electronic cigarette, in particular, represents a new threat especially among young individuals and pregnant women, who often consider such devices less harmful.^356^Electronic cigarette use can lead to nicotine levels up to six times higher than those of conventional cigarettes, increasing the risk of dependence, oxidative stress, endothelial dysfunction, and vascular inflammation.^356^Similarly, alcohol consumption significantly affects female health and is associated with higher prevalence of MS, dyslipidemias, and hyperinsulinemia.^357^

Estrogen reduction in menopause increases inflammatory and atherosclerotic susceptibility, endothelial dysfunction, arterial stiffness, and lipid changes, contributing to the pro-atherogenic and procoagulant state.^358^Addition of the inflammatory factor of tobacco use in postmenopause accelerates the atherosclerotic process.

### 10.6. Specific Clinical Conditions

Endometriosis and POS are frequently associated with hormonal imbalances that exacerbate IR, increase the risk of developing T2DM, and promote subclinical inflammation.^355^In POS, hormonal dysregulation affects the metabolism of glycoproteins and intensifies fat accumulation, contributing to an unfavorable lipid profile and increasing CVD risk.^359^In endometriosis, the chronic inflammation associated with the ectopic presence of endometrial tissue worsens local symptoms and impacts negatively the systemic environment, affecting metabolic and vascular regulatory mechanisms.^360^

In conclusion, nonpharmacological interventions have shown clear efficacy in the management of CVRFs.^139^The combination of regular physical activity with nutritional interventions, in addition to psychosocial support, for weight control results in a significant improvement in vascular and metabolic parameters.^361^

The multidisciplinary approach has proven effective, allowing personalization and adaptation of strategies to individual needs, generating sustained lifestyle changes, and reducing morbidity and mortality due to cardiometabolic disorders.^361^

### 10.7. Pharmacological Strategies

#### 10.7.1. Systemic Arterial Hypertension Treatment

Hypertension is one of the most prevalent RFs for CVD in women; its presence is observed in all life phases and increases progressively as age advances.^362^The choice and conduction of the pharmacological strategies should consider the phases of the reproductive cycle, including perimenopause and postmenopause. Certain conditions associated with secondary SAH, such as renovascular disease resulting from fibromuscular dysplasia, Cushing syndrome of endogenous origin, as well as thyroid and parathyroid disorders, are more prevalent among women.^363^

- Angiotensin-converting-enzyme inhibitors and angiotensin receptor blockers: With proven efficacy to reduce BP levels, they offer additional benefits, such as nephroprotection and left ventricular hypertrophy regression. They are recommended as first line drugs, especially for women with diabetes, CKD with albuminuria, HF with reduced ejection fraction, or IHD. In addition, they can be safely used in patients without comorbidities.^214 , 364^The use of angiotensin-converting-enzyme inhibitors (ACEI) and angiotensin receptor blockers II (ARB) in women of reproductive age requires caution because of their teratogenic risk, and it is fundamental not only to exclude the possibility of pregnancy before starting their use but to ensure contraception use during therapy as well.^214^
- Calcium channel blockers: Widely recommended for SAH management in women, they are a therapeutic option in perimenopause and postmenopause for patients without significant comorbidities.^214^It is important to emphasize that calcium channel blockers can be associated with worsening of VMS, such as hot flushes and night sweats in menopausal women. These medications can intensify VMS, with negative impact on quality of life.^214^
- Thiazide diuretics: Effective to control SAH in women with overweight or obesity, especially when edema is associated.^214^Of the major representatives, indapamide stands out due to its additional vasodilating effect and prolonged action, providing sustained BP control and lower metabolic impact as compared to hydrochlorothiazide.^364^Although widely available and accessible, hydrochlorothiazide has a shorter efficacy and higher risk for electrolytic and metabolic disorders.^364^The choice should be individualized, considering clinical profile and therapeutic objectives.
- Spironolactone: This aldosterone antagonist is effective in resistant SAH, when there is higher activation of the renin-angiotensin-aldosterone system.^214^It is also indicated in primary hyperaldosteronism and POS, because of its antiandrogenic action.^214 , 364^It has a favorable metabolic profile, with low impact on glycemia and lipids, which is relevant in women with increased CVR.^214^It is necessary to monitor renal function and potassium levels, mainly in elderly female patients or those with CKD, because of the risk of hyperkalemia.^364^

The hypertensive disorders of pregnancy were discussed in chapter 7, as was their therapeutic approach. Figure 10.1 shows the implications of these disorders over the course of a woman's life.

**Figure 10.1:** Implications of hypertensive disorders of pregnancy over the course of a woman's life. DM: diabetes mellitus; HF: heart failure; IHD: ischemic heart disease; SAH: systemic arterial hypertension. Source: Davis et al.^365^

#### 10.7.2. Management of Dyslipidemias

Dyslipidemia is particularly important in menopause transition. The drop in estrogen levels is related to relevant metabolic changes, such as IR, body fat redistribution with predominant abdominal accumulation, and lipid profile changes.^366^Menopause associates with significant elevations in the levels of total cholesterol, LDL-c, apolipoprotein B, triglycerides, and Lp(a), in addition to a possible reduction in the protective antiatherogenic effect of HDL-c.^109^Levels of LDL-c ≥130 mg/dL are considered elevated, while HDL-c levels <50 mg/dL represent an additional RF for CVD, especially when associated with other MS components.^109^This CVR increase should be considered in the individualized therapeutic management, and dyslipidemia treatment should be based not only on the levels of LDL-c, HDL-c, and triglycerides, but also on the patient's global CVR profile.^109 , 212^

#### 10.7.3. Oral Lipid-Lowering Drugs

Statins are the first-line therapy for dyslipidemia. They are effective to reduce cardiovascular events and atherosclerosis in women at high risk, with benefits comparable to those observed in men.^367^They should be indicated for both primary and secondary prevention, with LDL-c goals defined according to CVR: < 100 mg/dL for intermediate risk; < 70 mg/dL for high risk; and < 50 mg/dL for very high risk.^367 , 368^In women of reproductive age, statin use should be individualized for those at high risk, being contraindicated during pregnancy and lactation.^369^

Ezetimibe, when associated with statins, enhances LDL-c reduction and contributes to atherosclerosis regression and endothelial function improvement, maintaining safety and efficacy similar in both sexes.^367^It is particularly useful in women with intolerance to elevated doses of statins or with insufficient response to monotherapy.

Although HDL-c levels over 50 mg/dL are desirable in women, LDL-c reduction remains the therapeutic priority.^367^Hypertriglyceridemia, which is frequent especially in menopause, should be treated when triglycerides exceed 200 mg/dL and must be treated when over 500 mg/dL.^367^Of fibrates, fenofibrate is the preferential option, because of its safety profile and additional metabolic benefits in women.^109^

PCSK9 inhibitors, such as alirocumab, evolocumab, and inclisiran, are indicated for patients with elevated LDL-c levels who do not meet their goals with statins or are intolerant to them, including cases of familial hypercholesterolemia.^368^Inclisiran, an RNA silencer, stands out due to its semestral posology after the induction dose, with good adhesion and sustained efficacy. These medications should not be used during pregnancy or lactation and have similar efficacy in women and men to reduce cardiovascular events.^368^

The maternal and fetal impacts of atherogenic lipid profiles during pregnancy were described in chapter 7 and are summarized in Figure 10.2. The recommendations for addressing dyslipidemias in pregnancy are shown in Figure 10.3. .

**Figure 10.2:** Maternal and fetal impacts of atherogenic lipid profiles during pregnancy.

**Figure 10.3:** Recommendations for the treatment of dyslipidemia during pregnancy.

#### 10.7.4. Diabetes *Mellitus* Control

Although there is not sufficient evidence available for female-sex-specific therapeutic recommendations, sex-specific hormonal, behavioral, and social factors should be considered in the management of T2DM and its complications. In addition, it is fundamental to raise awareness of these differences among health professionals and patients to ensure more equitable and effective care.

Therefore, women's T2DM management requires a comprehensive approach that contemplates clinical and metabolic heterogeneity. The key objective is to meet specific goals to reduce cardiovascular and metabolic morbidity and mortality. The most relevant goals are HbA1c <7% and control of body weight and abdominal circumference, which are factors closely related to IR and CVR.^369^

Women have a different progression of prediabetes to T2DM, frequently associated with higher indices of obesity and increased risk of metabolic complications. Thus, the management should contemplate not only glycemic control, but also specific strategies to reduce IR and prevent associated comorbidities.^109 , 209^

Regarding the drugs used to treat T2DM, some considerations are worth noting: women can have higher risk of hypoglycemia with sulfonylureas and different response to glitazones, related to kidney function and body composition.^182^However, men can have higher response to weight loss induced by SGLT2 inhibitors, partially due to the difference in body fat distribution. In addition, SGLT2 inhibitors are associated with a higher rate of genital fungal infections, especially in women.^291^Regarding treatment with GLP-1 analogues, special attention should be given to women on oral contraception. The American Diabetes Association recommends that women on oral contraception should choose a non-oral contraceptive method or add a barrier method of contraception during the first four weeks of tirzepatide use (GLP-1 analogue), because of its effects on gastric emptying, with potential consequences to the pharmacokinetics of oral contraceptives.^292^Without implying sex-specific therapeutic recommendations, the following considerations are worthy of note:

- Metformin: remains the first choice for T2DM treatment, especially in asymptomatic cases, in POS, in the absence of established CVD or kidney disease, and in individuals at low global CVR.^369^
- SGLT2 inhibitors: show important benefits in glycemia reduction and offer cardiovascular and renal protection in women with elevated CVR, HFpEF, HF with reduced ejection fraction, and albuminuric CKD.^369^
- Pioglitazone: is a particularly useful thiazolidinedione for women with IR, such as POS, and prediabetes or T2DM with atherogenic metabolic profile. In addition to improving insulin sensitivity, it has beneficial effects on lipid profile and vascular inflammation. However, it should be carefully used, considering risks, such as weight gain, bone mass loss, and fluid retention, especially in women at high risk for HF.^369^

#### 10.7.5. Management of Obesity

Obesity has a challenging and complex management and should ideally be addressed by a multiprofessional team. The treatment should consider minimizing the stigma related to obesity and requires special attention in women because of their higher vulnerability.^214^It is important to establish with the patient feasible therapeutic targets associated with clinical benefits, as well as to use strategies to approach weight loss, such as those to fight tobacco use (5 As: ask, assess, advice, agree, assist *).* ^271^The Brazilian Society for the Study of Obesity and Metabolic Syndrome (ABESO) has proposed a classification of obesity based on body weight history and therapeutic goals derived from that assessment, thus aiming at a goal consisting in the proportion of weight loss in relation to maximum BMI in life. The non-realistic goal of BMI normalization to <25 kg/m^2^is not recommended.^252^Goals are shown in Table 10.1 .

**Table 10.1:** Proposed classification of ‘reduced’ and ‘controlled’ obesity based on maximum BMI

Maximum BMI	Unchanged *	Reduced *	Controlled *
30–40 kg/m²	< 5%	5–9.9%	> 10%
40–50 kg/m²	< 10%	10–14.9%	> 15%

* Percent reductions in body weight in relation to maximum BMI in life.^252^

As already mentioned, nonpharmacological measures, such as calorie restriction diet, regular physical exercise practice, and programs including psychosocial approach, are fundamental to manage metabolic diseases, notably obesity. Calorie restriction is more relevant for weight reduction, but physical exercise practice is essential to maintain weight loss and prevent muscle mass loss that usually accompanies weight loss.^271^However, the benefits of nonpharmacological measures tend to decrease with time,^272^and no benefit to long-term cardiovascular outcomes is found with that intervention for women and men. A subgroup analysis of the Look AHEAD clinical trial with patients who lost at least 10% of their body weight in the first year has shown a 20% reduction in the primary outcome (major adverse cardiovascular events and hospitalization due to angina).^273^

There are several pharmacological treatments for obesity with varied effects on body weight, but only most recent treatments associated with a reduction in outcomes. The SELECT trial was the first to show cardiovascular benefits in that scenario.^274^The use of semaglutide, a GLP-1 agonist, subcutaneously, at the weekly dose of 2.4 mg to patients with overweight and established CVD for approximately 3 years reduced body weight by 8.51% and resulted in a 28% decrease in the risk for major adverse cardiovascular events. The subgroup analysis of the women included (n=4872, 28% of the sample) showed a reduction of HR 0.84 (95%CI: 0.66-1.07), while men had 0.79 (95%CI: 0.70-0.90), with no report if there was a statistically significant difference in the results between the groups analyzed. The numerical differences found might be related to the lower power of the subgroup analysis of women because of its reduced sample size. Several other more potent antiobesity medications, leading to body weight reduction of up to 25%, are being studied.^275^However, the results of phase 3 studies with cardiovascular outcomes have not been made available.

#### 10.7.6. Management of Metabolic Dysfunction-Associated Steatotic Liver Disease

The treatment of MASLD consists in lifestyle change with focus on body weight reduction of at least 5%, because weight loss is the most effective measure for histological improvement of MASLD.^295 , 298^Alcohol consumption should be limited, as well as the intake of fructose used in ultra-processed foods and sugary drinks. In addition to lifestyle change, women with more advanced forms of MASLD can benefit from pharmacological therapies that act on MASH and/or liver fibrosis. Some examples of such drugs are as follows: resmetirom, semaglutide, pioglitazone, tirzepatide, vitamin E, and SGLT2 inhibitors.

Resmetirom, a selective agonist of thyroid hormone receptor β (THR- β), was the first drug to be approved by a regulatory agency for the treatment of non-cirrhotic MASH, with moderate to advanced fibrosis (compatible with stages F2 and F3). In the MAESTRO-NASH,^310^phase 3 study that enrolled 966 participants with MASH treated for 52 weeks (322 in the 80-mg resmetirom group, 323 in the 100-mg resmetirom group, and 321 in the placebo group), resmetirom was associated with fibrosis improvement as compared to placebo. There was no subgroup analysis regarding sex. Semaglutide, a GLP-1 receptor agonist, also improved histological results in a population with MASH and fibrosis F2-F3. In the interim analysis of ESSENCE,^311^a phase 3 study that enrolled 800 participants treated for 70 weeks (57.1% were women and 55.9% had T2DM), there was significant improvement of MASH without fibrosis worsening and significant reduction in fibrosis without MASH worsening (primary outcomes) with the use of semaglutide at the weekly dose of 2.4 mg *versus* placebo.

Pioglitazone, a selective agonist of the peroxisome proliferator-activated receptor gamma (PPARγ), is recommended to treat MASH and/or fibrosis in individuals with T2DM,^295 , 298 , 308^because most studies comparing pioglitazone and placebo have shown benefits in inflammation and histological changes.^312^However, there was no sub-analysis regarding sex in most of them. It is worth noting that pioglitazone can worsen HF symptoms, because of fluid retention and, specifically in women, it is associated with increased risk of bone fractures and weight gain.^312 , 313^

Tirzepatide, a co-agonist of glucose-dependent insulinotropic polypeptide (GIP) and GLP-1 receptors already approved in some countries as an antidiabetic and antiobesity agent, has also been assessed to treat individuals with MASH and fibrosis F2-F3. In the SYNERGY-NASH,^314^a phase 2b placebo-controlled study, tirzepatide promoted MASH resolution without fibrosis worsening in 61% of the individuals assessed after 52 weeks of treatment (57% of patients were women, but no subanalysis was conducted in that group).

In the PIVENS study,^315^conducted in individuals with MASH and no T2DM, vitamin E use (800 IU/day) for two years improved liver disease activity, which was histologically estimated using the NAS score (Non-Alcoholic Steatohepatitis Activity Score), without fibrosis increase as compared to placebo (43% vs. 19%; p < 0.001).

Finally, although studies of histological outcomes related to MASLD with the use of SGLT2 inhibitors are scarce, there is evidence of reduction in liver enzymes, liver fat, and liver stiffness assessed with elastography as a result of the use of those drugs in a population with T2DM.^316 - 318^Thus, some guidelines for the management of MASLD recommend that treatment with SGLT2 inhibitors be considered for individuals with T2DM and MASH and/or fibrosis, without sex-related difference regarding outcomes.^295 , 298 , 308^

When the combination of lifestyle change and pharmacotherapy fails, individuals with MASLD associated with fibrosis and obesity from class II on should be considered for bariatric surgery.^319^The benefits of that surgery for MASLD have been consistent in several studies assessing different surgical techniques and, although MASH reduction is evident by the end of the first postoperative year, significant benefits for fibrosis require longer, as shown in studies with follow-up of 5 to 6 years.^320 , 321^

#### 10.7.7. Management of Chronic Kidney Disease

Current data in the literature do not allow the use of different criteria for screening, diagnosis, and treatment of CKD, especially DKD, and, thus, we recommend that the Brazilian Society of Diabetes criteria be used. Further research is necessary to identify clinically relevant physiological differences between sexes, aimed at identifying new therapies that change clinical outcomes.

The sex-related differences in albuminuria can influence treatment, with therapies targeted at reducing proteinuria, such as renin-angiotensin-aldosterone system blockade, SGLT2 inhibitors, and mineralocorticoid receptor antagonists.^331^

Large randomized clinical trials using SGLT2 inhibitors have shown that women and men seem to benefit equally, although only 28.5% to 36.9% of those studies’ participants were women. An analysis of the EMPA-REG OUTCOME, CANVAS, DECLARE TIMI-58, and CREDENCE trials has shown that there was no sex-related difference in the reduction of major adverse cardiovascular events or adverse events from SGLT2 inhibitors, such as amputation, fracture, and urinary tract infection, although women were more likely to have genital infection.^370^

Despite the equal benefit from SGLT2 inhibitors independently of sex, women are less likely to receive a prescription of an SGLT2 inhibitor, even when diagnosed with DKD, HF with reduced ejection fraction, or atherosclerotic CVD. This deprives many women from the cardiovascular and renal benefits of those drugs.

Table 10.2 shows a summary of the drug classes for treatment of DKD and sex-related differences.

**Table 10.2:** Sex-related differences in the therapies for diabetic kidney disease

Therapy	Sex differences
SGLT2 inhibitors	No difference in cardiovascular or renal benefit. Increased risk of diabetic ketoacidosis in women with T1DM on off-label use, maybe related to higher ketogenesis in women. Increased risk of genital fungal infections in women.
GLP-1 agonists and dipeptidyl peptidase-4 inhibitors	No difference between sexes. Theoretical sex-related difference in vascular response to nitric oxide.
Mineralocorticoid receptor antagonist	No difference between sexes in randomized clinical trials.
Endothelin receptor antagonist	Sex-based differences in endothelin receptor expression. No difference in randomized clinical trials.
Metformin	Greater gastrointestinal adverse effects in women, with potential improvement at lower doses.
RAAS blockade	In women, estrogen decreases renin, ACE, and Ang II, and increases angiotensinogen and Ang 1-7. Contraindicated in pregnancy.
Menopausal hormone therapy	Estradiol, progesterone, or combined hormonal therapy reduce albuminuria in postmenopausal women. Controversial data.

Adapted from Sridhar et al.^338^ACE: angiotensin-converting enzyme; Ang 1-7: angiotensin 1-7; Ang II: angiotensin II; GLP-1: glucagon-like peptide 1; RAAS: renin-angiotensin-aldosterone system; SGLT2: sodium-glucose cotransporter type 2; T1DM: type 1 diabetes mellitus.

#### 10.7.8. Role of GLP-1 Analogues in Women's Cardiometabolic Treatment

The GLP-1 analogues promote glucose-dependent insulin secretion, reduce appetite, and delay gastric emptying. They contribute to effective glycemic control and improvement of CVR markers.^369^

#### 10.7.9. Specificities in Women

Female hormonal factors and body fat distribution contribute to a more severe expression of cardiometabolic syndrome. Studies have shown that GLP-1 analogues have particularly beneficial effects, with higher impact on weight reduction and improvement of inflammatory and lipid parameters in women as compared to men. This can be partially explained by different hormonal responses and higher sensitivity to the anorexigenic effects of those drugs.^371 , 372^

#### 10.7.10. Semaglutide

Semaglutide is a GLP-1 receptor agonist, with consolidated efficacy in glycemic control and weight reduction. In overweight or obese patients, semaglutide has shown significant reductions in body weight and HbA1c, even in the absence of T2DM.^371^In patients with T2DM, it reduced by 26% the risk of major cardiovascular adverse events.^135^In individuals with previous atherosclerotic CVD, BMI ≥ 27, and no history of T2DM, semagludite reduced by 20% the combined risk of major cardiovascular adverse events and all-cause mortality.^274^In addition, significant renal effects have been shown in patients with T2DM and CKD. There was a 24% reduction in the risk of severe kidney events and death due to renal or cardiovascular causes in the group treated with semaglutide, with smaller annual decline in estimated glomerular filtration rate.^373^Recent evidence has shown that its oral use in patients with diabetes also contributes to reduce cardiovascular events, being useful in clinical contexts where the injectable use is less feasible.^374^

#### 10.7.11. Tirzepatide

Tirzepatide, a dual GIP and GLP-1 receptor agonist, represents an advance in cardiometabolic disease management. Its use has shown significant reductions in HbA1c levels (up to 2.4%) and body weight (up to 20%), in addition to lipid profile improvement and reduction in inflammatory markers.^375 , 376^These characteristics make this dual GIP/GLP1 agonist a promising alternative, especially in women with central obesity and IR.

#### 10.7.12. Future Perspectives

A recent study assessing the efficacy of the weekly use of 2.4 mg of semaglutide as compared to 5-15 mg of tirzepatide to treat obesity has shown the superiority of tirzepatide for both weight loss and metabolic control, with comparable cardiovascular effects and safety profile. However, the subgroup of women treated with semaglutide showed higher proportional weight loss as compared to men and improvement of cardiometabolic RFs after 72 weeks of treatment. Planning and final therapeutic decision should always be individual and based on each patient's physiological, hormonal, and metabolic characteristics.^376^

### 10.8. Specific pharmacological considerations

#### 10.8.1. Drug interactions

Women with chronic conditions, such as osteoporosis, depression, and anxiety, are frequently exposed to polypharmacy, increasing the risk of relevant drug interactions, because of lower renal clearance and higher body fat proportion. Of the interactions with highest impact, selective serotonin reuptake inhibitors stand out. They can inhibit CYP3A4 and interfere with the metabolism of lipophilic statins, increasing the risk of myopathy.^367^

#### 10.8.2. Importance of Weight Control

Interventions that reduce body weight have a direct impact on lipid profile improvement, BP control, and insulin sensitivity. Of such interventions, GLP-1 agonists stand out, showing consistent effects on weight reduction with additional benefits to the prevention of major cardiovascular adverse events. Management of obesity should be understood as a fundamental component of the therapeutic strategy for cardiometabolic disorders.^117 , 274 , 373 - 376^

#### 10.8.3. Individualized Approach

The clinical heterogeneity among women requires personalized approaches for the management of cardiometabolic disorders that consider not only physiological parameters, such as age and presence of comorbidities, but also psychosocial factors, reproductive history, and individual preferences. Therapeutic individualization, thus, represents an essential pillar for woman-centered care, promoting more effective and safe interventions throughout a woman's healthcare journey.^377 , 378^

### 10.9. Surgical Treatment

#### 10.9.1. Bariatric Surgery

Bariatric surgery is a widely acknowledged strategy across the globe, with perioperative mortality ranging from 0.03% to 0.2%.^379^In Brazil, approximately 70% of the patients undergoing the procedure are women.^380^Its benefits comprise a significant improvement of MS and associated comorbidities, such as T2DM, sleep obstructive apnea, SAH, dyslipidemia, Pickwick syndrome, MASLD, and gastroesophageal reflux disease.^381^The indication follows guidelines that recommend surgery to patients with BMI ≥ 35 kg/m² and associated comorbidities, or isolated BMI ≥ 40 kg/m², independently of the presence of other diseases.^382^

Bariatric surgery has been associated with a lower incidence of major cardiovascular adverse events in patients with CVD and obesity in a cohort of 2638 patients followed up for 4.6 years, with the highest benefit found in the groups with HF and IHD.^383^

After the procedure, women show a slightly smaller weight loss than men. The weight reduction can lead to an abrupt SHBG increase, testosterone decrease, and FSH elevation, improving ovulatory dysfunction and menstrual irregularity, thus favoring spontaneous conception at reproductive age.^384^

Contraindications to bariatric surgery are as follows: severe psychiatric disease without control; moderate to severe dementias; alcohol or illegal drug dependence; severe IHD or other severe heart diseases; and portal hypertension with esophageal varices. In patients with BMI > 50 kg/m², the surgical risk is high because of the higher incidence of comorbidities and anatomical complexity, which result in longer surgery duration, higher perioperative morbidity, and longer hospitalizations, according to some studies.^382^

Figure 10.4 summarizes the strategies to approach women's cardiometabolic disorders, emphasizing the importance of a woman-centered multidisciplinary approach, involving the promotion of healthy habits, individualized screening of RFs and their control, integrated clinical management, and inclusion of psychosocial aspects in the cardiometabolic assessment.

**Figure 10.4:** Strategies to approach women's cardiometabolic disorders. ACEI: angiotensin-converting-enzyme inhibitor; ARB: angiotensin receptor blocker; CCB: calcium channel blocker; CKD: chronic kidney disease; CV: cardiovascular; CVD: cardiovascular disease; CVR: cardiovascular risk; DLP: dyslipidemia; T2DM: type 2 diabetes mellitus; GLP-1: glucagon-like peptide 1; HFpEF: heart failure with preserved ejection fraction; HFrEF: heart failure with reduced ejection fraction; MACE: major adverse cardiovascular events; MASLD: metabolic dysfunction-associated steatotic liver disease; MRA: mineralocorticoid receptor antagonist; MS: metabolic syndrome; POS: polycystic ovary syndrome; RAAS: renin-angiotensin-aldosterone system; SAH: systemic arterial hypertension; SGLT2: sodium-glucose cotransporter type 2; VMS: vasomotor symptoms.

## 11. Recommendations for the Management of Cardiometabolic Disorders in Women

For the recommendations provided at the end of this chapter, a systematic review was conducted (Supplement 1) with ten PICO (Population, Intervention, Comparison, and Outcome) questions. This systematic review included systematic reviews, metanalyses, multicenter randomized controlled trials, and guidelines. The following databases were searched: PubMed/MEDLINE, Embase, Cochrane Library, LILACS, and BVS. The GRADE (Grading of Recommendations Assessment, Development and Evaluation) approach, a system for assessing the certainty of evidence and the strength of recommendation in systematic reviews and clinical practice guidelines was used. The GRADE approach classifies evidence into levels (high, moderate, low, or very low) and, based on that classification, determines the direction (AGAINST or IN FAVOR) and the strength of recommendation (STRONG or WEAK) as follows.

- High: There is high confidence that effect estimates are close to the true effect.
- Moderate: There is moderate confidence in effect estimates. Future studies are likely to impact confidence in effect estimates.
- Low: Confidence in effect estimates is limited.
- Very low: There is uncertainty regarding effect estimates.

Figure 11.1 summarizes the structure of the systematic review supporting this position statement.

**Figure 11.1:** Summarizes the structure of the systematic review supporting this position statement.

Although the importance of cardiometabolic disorders in women has been increasingly acknowledged, there is significant lack of evidence from multicenter randomized controlled trials specifically addressing the treatment of those conditions in the female sex. This limitation impairs the elaboration of clinical recommendations based on solid evidence adapted to women's particularities. It is worth noting that women continue to be under-represented in clinical trials of cardiovascular diseases and cardiometabolic disorders. In recent analyses, female participation in large multicenter randomized controlled trials has ranged from 27% to 38% of participants, a smaller proportion than the prevalence of those disorders in the female population.^385 , 386^Only one third of studies has reported analyses of women-specific results, hindering the efficacy and safety assessment of treatments in that group. In addition, the scarcity of multicenter randomized controlled trials focused on women determines that many clinical recommendations be based on indirect evidence or evidence extrapolated from studies whose sample is mostly male, which might not reflect the best management of cardiometabolic disorders in women.^387^

There is strong correlation between cardiometabolic disorders and inflammatory conditions over the course of a woman's life, such as POS, GD, and preeclampsia, which predispose to increased risk for AMI, stroke, and IHD, as well as later repercussion on the mother-child dyad. Recent study with 12,480 mother-child dyads has shown that maternal cardiometabolic RFs were significantly associated with 4.88% and 1.90% increases in systolic and diastolic BP of the child, respectively. The combination of hypertensive disorders of pregnancy with pregestational obesity or GD was significantly associated with higher BP between the ages of 2 years and 18 years, emphasizing the importance of the early management of cardiometabolic disorders in women's childhood, adolescence, and reproductive period.^388^

Sex-related differences regarding cardiometabolic disorders and inflammatory diseases have suggested that sex hormones regulate women's inflammatory pathways. There are changes in women's inflammatory signaling that can account for differences in hypertension, atherosclerosis, obesity, CKD, and MASLD. Knowing the specific mechanisms that boost women's chronic inflammatory conditions, such as POS, GD, and postmenopausal hypertension, will support the multidisciplinary approach necessary to decrease the burden of CVDs in women.^30^

Several interventions have a combined effect on chronic diseases, especially the cardiometabolic ones. A recent study has assessed the associations between physical activity pattern and incidence of 678 conditions in 89,573 participants (mean age, 62 ± 8 years; 56% women) in the UK Biobank prospective cohort study, who used an accelerometer for one week from June 2013 to December 2015. Both physical activities concentrated over 1 or 2 days and more regular activity patterns were associated with a similarly lower risk for more than 200 diseases, particularly lower risk of cardiometabolic disorders.^389^

In what follows, we present the recommendations with relatively robust evidence for cardiometabolic disorders in women.

## CURRENT RECOMMENDATIONS FOR CARDIOMETABOLIC DISORDERS IN WOMEN

DIET^390 - 398^

**Table:** 

Recommendations – IN FAVOR	Strength of recommendation	Certainty of evidence
For women with overweight, obesity, metabolic syndrome, hypertension, dyslipidemia, MASLD, and T2DM, comprehensive lifestyle interventions are recommended using Mediterranean diet, DASH, intermittent energy restriction diet, diets with high protein content and calorie restriction aimed at an average body weight reduction of 5-10%.	STRONG	HIGH

T2DM: type 2 diabetes^mellitus^; DASH: Dietary Approaches to Stop Hypertension; MASLD: metabolic dysfunction-associated steatotic liver disease.

PHYSICAL ACTIVITY^399 - 408^

**Table:** 

Recommendations – IN FAVOR	Strength of recommendation	Certainty of evidence
Regular practice of physical activity with the initial goal of 150 minutes per week of aerobic exercises and resistance training two to three times per week are recommended for perimenopausal women with overweight, obesity, metabolic syndrome, T2DM, hypertension, and dyslipidemia. For weight loss ≥5%, it should be increased to 300 minutes per week, given that visceral fat reduction is related to short- and midterm improvement in cardiometabolic outcomes.	STRONG	MODERATE

T2DM: type 2 diabetes mellitus.

ALCOHOL CONSUMPTION AND TOBACCO USE^356 , 409 - 417^

**Table:** 

Recommendations – IN FAVOR	Strength of recommendation	Certainty of evidence
Alcohol consumption increases the risk of anemia and gestational diabetes, while tobacco use doubles the chance of low weight at birth and increases the chances of infertility and early menopause. Alcohol consumption, even at moderate amounts, and tobacco use by women can elevate blood pressure, increase serum levels of LDL-c and triglycerides, decrease serum HDL-c levels, and increase the risk of stroke, heart failure, insulin resistance, and T2DM. Non-pregnant women should not smoke and consume <140 g of alcohol per week.	STRONG	HIGH

T2DM: type 2 diabetes mellitus.

OBESITY^274 , 418 - 425^

**Table:** 

Recommendations – IN FAVOR	Strength of recommendation	Certainty of evidence
Lifestyle changes associated with the use of GLP-1 agonists and SGLT2 inhibitors are recommended for the treatment of obesity and T2DM in women, with beneficial effects on weight reduction and metabolic parameters.	STRONG	HIGH

T2DM: type 2 diabetes mellitus; GLP-1: glucagon-like peptide-1; SGLT2: sodium-glucose cotransporter type 2.

## CURRENT RECOMMENDATIONS FOR CARDIOMETABOLIC DISORDERS IN WOMEN

DIABETES^420 , 426 - 432^

**Table:** 

Recommendations – IN FAVOR	Strength of recommendation	Certainty of evidence
Lifestyle changes associated with the use of GLP-1 agonists and SGLT2 inhibitors are recommended for the treatment of T2DM in women, with beneficial effects on weight reduction and metabolic parameters, such as glycated hemoglobin reduction. Metformin should not be used as a first-line agent for the management of diabetes in pregnancy, and, when used to treat POS and induce ovulation, it should be discontinued up to the end of the first trimester, because it crosses the placental barrier.	STRONG	HIGH

T2DM: type 2 diabetes mellitus; POS: polycystic ovary syndrome; GLP-1: glucagon-like peptide-1; SGLT2: sodium-glucose cotransporter type 2.

DYSLIPIDEMIA^366^

**Table:** 

Recommendations – IN FAVOR	Strength of recommendation	Certainty of evidence
In women at very high cardiovascular risk on secondary prevention, in addition to lifestyle changes, high-potency statins alone or in combination with ezetimibe should be used to reduce LDL-c ≥ 50%, targeting at LDL-c < 50 mg/dL and non-HDL-c < 80 mg/dL, independently of baseline LDL-c level.	STRONG	HIGH
In women at high cardiovascular risk on secondary prevention, in addition to lifestyle changes, high-potency statins alone or in combination with ezetimibe should be used to reduce LDL-c ≥ 50%, targeting at LDL-c < 70 mg/dL and non-HDL-c < 100 mg/dL, independently of baseline LDL-c level.	STRONG	HIGH

ARTERIAL HYPERTENSION^209 , 211 , 214^

**Table:** 

Recommendations – IN FAVOR	Strength of recommendation	Certainty of evidence
Body weight control to achieve healthy values of BMI (20–25 kg/m2) and waist circumference (<80 cm in women) is recommended to reduce BP and cardiovascular risk. The practice of low-to-moderate intensity exercises is recommended for all pregnant women without contraindications to reduce the risk of gestational hypertension and preeclampsia.	STRONG	HIGH
In women with chronic or gestational hypertension, drug treatment is recommended for those with confirmed systolic BP ≥ 140 mm Hg or diastolic BP ≥ 90 mm Hg.	STRONG	HIGH

BMI: body mass index; BP: blood pressure.

METABOLIC DYSFUNCTION-ASSOCIATED STEATOTIC LIVER DISEASE^309 , 311 , 314 , 302 , 414 , 433 , 434^

**Table:** 

Recommendations – IN FAVOR	Strength of recommendation	Certainty of evidence
EIn women with MASLD, the following are recommended: lifestyle changes (weight loss, dietary changes, physical exercises, tobacco and alcohol use cessation); optimized management of comorbidities, such as incretin-based therapies (ex: semaglutide, tirzepatide) for T2DM or obesity (when indicated); and bariatric surgery in the presence of severe obesity.	STRONG	MODERATE

T2DM: type 2 diabetes mellitus; MASLD: metabolic dysfunction-associated steatotic liver disease.

## CURRENT RECOMMENDATIONS FOR CARDIOMETABOLIC DISORDERS IN WOMEN

CHRONIC KIDNEY DISEASE^435 - 441^

**Table:** 

Recommendations – IN FAVOR	Strength of recommendation	Certainty of evidence
Pregnancy is one of the major causes of acute kidney injury in women of reproductive age and, along with preeclampsia, can lead to subsequent CKD. CKD has a negative effect on pregnancy, even at very initial stages, and the risks increase with CKD progression and concomitance of T2DM. Monitoring renal function markers during pregnancy and following years is recommended.	STRONG	MODERATE

T2DM: type 2 diabetes mellitus; CKD: chronic kidney disease.

SURGICAL THERAPY * – BARIATRIC SURGERY^442 - 448^

* *(Roux-en-Y gastric bypass, vertical gastrectomy, laparoscopic adjustable gastric banding, duodenal jejunal bypass liner/biliopancreatic diversion)*

**Table:** 

Recommendations – IN FAVOR	Strength of recommendation	Certainty of evidence
Bariatric surgery is recommended for women with BMI ≥ 35 kg/m² and history of diabetes, MASLD, or high risk for cardiovascular events, as well as for those with BMI ≥ 40 kg/m², independently of comorbidities, to improve cardiometabolic parameters (lipid and glycemic profile) and inflammatory markers.	STRONG	HIGH

MASLD: metabolic dysfunction-associated steatotic liver disease; BMI: body mass index.

POLYCYSTIC OVARY SYNDROME^43 , 65 , 97 , 141 , 156 , 159 , 172 , 265 , 304 , 444^

**Table:** 

Recommendations – IN FAVOR	Strength of recommendation	Certainty of evidence
POS is associated with anovulation, hyperandrogenism, and insulin resistance, increasing the risk of cardiovascular diseases and T2DM. Women with POS should undergo complete assessment of lipid profile and glycemic status at the time of diagnosis. Treatment includes, in addition to lifestyle changes, insulin sensitizers, such as metformin or myoinositol, and, for hirsutism and irregular cycles, combined oral contraceptives. Antiobesity agents, such as liraglutide, semaglutide, and GLP-1 agonists and orlistat, can also be indicated for weight control.	STRONG	MODERATE

POS: polycystic ovary syndrome; T2DM: type 2 diabetes *mellitus* ; GLP-1: glucagon-like peptide-1.

ENDOMETRIOSIS^203 - 206^

**Table:** 

Recommendations – IN FAVOR	Strength of recommendation	Certainty of evidence
Endometriosis is associated with a chronic inflammatory process with increased oxidative stress and elevation in cardiovascular risk factors, higher risk of hypertension, lipid changes, coronary artery disease, heart failure, and stroke. Hormonal therapies and ovarian stimulation for in vitro fertilization can also elevate the risk of thromboembolism. Lifestyle changes can help reduce cardiovascular risk.	WEAK	WEAK

## SUPPLEMENT

**1:** Which biomarkers help the diagnosis of cardiometabolic disorders in women?

Database	Search strategy
MEDLINE/PubMed	("Biomarkers"[Mesh] OR "biological markers"[tiab] OR biomarkers[tiab] OR "molecular markers"[tiab] OR "diagnostic markers"[tiab] OR "metabolic markers"[tiab] OR "cardiometabolic biomarkers"[tiab]) AND ("Metabolic Syndrome"[Mesh] OR "Cardiovascular Diseases"[Mesh] OR "Glucose Intolerance"[Mesh] OR "Hypertension"[Mesh] OR "Dyslipidemias"[Mesh] OR "Insulin Resistance"[Mesh] OR "Type 2 Diabetes Mellitus"[Mesh] OR "cardiometabolic risk"[tiab] OR "cardiometabolic abnormalities"[tiab] OR "metabolic syndrome"[tiab] OR "insulin resistance"[tiab] OR "glucose intolerance"[tiab] OR dyslipidemia*[tiab] OR hypertension[tiab] OR "type 2 diabetes"[tiab] OR "cardiovascular disease"[tiab]) AND ("Women"[Mesh] OR "Female"[Mesh] OR women[tiab] OR woman[tiab] OR female*[tiab]) Filters applied: in the last 5 years, Randomized Controlled Trial, Systematic Review.
Embase	('biological marker'/exp OR 'biomarker'/exp OR 'biological marker':ti, ab OR biomarkers:ti, ab OR 'molecular marker':ti, ab OR 'diagnostic marker':ti, ab OR 'metabolic marker':ti, ab OR 'cardiometabolic biomarker':ti, ab) AND ('cardiometabolic disorder'/exp OR 'metabolic syndrome'/exp OR 'cardiovascular disease'/exp OR 'glucose intolerance'/exp OR 'hypertension'/exp OR 'dyslipidemia'/exp OR 'insulin resistance'/exp OR 'type 2 diabetes mellitus'/exp OR 'cardiometabolic risk':ti, ab OR 'cardiometabolic abnormalit*':ti, ab OR 'metabolic syndrome':ti, ab OR 'insulin resistance':ti, ab OR 'glucose intolerance':ti, ab OR dyslipidemi*:ti, ab OR hypertension:ti, ab OR 'type 2 diabetes':ti, ab OR 'cardiovascular disease':ti, ab) AND ('female'/exp OR women:ti, ab OR woman:ti, ab OR female*:ti, ab)
Cochrane	([mh "Biomarkers"] OR "biological markers":ti, ab OR biomarkers:ti, ab OR "molecular markers":ti, ab OR "diagnostic markers":ti, ab OR "metabolic markers":ti, ab OR "cardiometabolic biomarkers":ti, ab) AND ([mh "Metabolic Syndrome"] OR [mh "Cardiovascular Diseases"] OR [mh "Glucose Intolerance"] OR [mh "Hypertension"] OR [mh "Dyslipidemias"] OR [mh "Insulin Resistance"] OR [mh "Type 2 Diabetes Mellitus"] OR "cardiometabolic risk":ti, ab OR "cardiometabolic abnormalities":ti, ab OR "metabolic syndrome":ti, ab OR "insulin resistance":ti, ab OR "glucose intolerance":ti, ab OR dyslipidemia*:ti, ab OR hypertension:ti, ab OR "type 2 diabetes":ti, ab OR "cardiovascular disease":ti, ab) AND ([mh "Women"] OR [mh "Female"] OR women:ti, ab OR woman:ti, ab OR female*:ti, ab)
BVS	(TW: "Biomarcadores" OR "Biomarkers" OR "Biomarcadores" OR "Marcadores Biológicos" OR "Biological Markers" OR "Marcadores Biológicos" OR "Marcadores moleculares" OR "Molecular Markers" OR "Marcadores diagnósticos" OR "Diagnostic Markers" OR "Marcadores metabólicos" OR "Metabolic Markers" OR "Marcadores cardiometabólicos" OR "Cardiometabolic Biomarkers") AND (TW: "Anormalidades Cardiometabólicas" OR "Cardiometabolic Abnormalities" OR "Anormalidades Cardiometabólicas" OR "Riesgo Cardiometabólico" OR "Cardiometabolic Risk" OR "Risco Cardiometabólico" OR "Síndrome Metabólica" OR "Metabolic Syndrome" OR "Síndrome Metabólica" OR "Resistência à Insulina" OR "Insulin Resistance" OR "Resistencia a la Insulina" OR "Intolerância à Glicose" OR "Glucose Intolerance" OR "Intolerancia a la Glucosa" OR "Dislipidemia" OR "Dyslipidemia" OR "Dislipidemia" OR "Hipertensão" OR "Hypertension" OR "Hipertensión" OR "Diabetes tipo 2" OR "Type 2 Diabetes" OR "Diabetes tipo 2" OR "Doenças Cardiovasculares" OR "Cardiovascular Diseases" OR "Enfermedades Cardiovasculares") AND (TW: "Mulheres" OR "Women" OR "Mujeres" OR "Feminino" OR "Female" OR "Femenino")

## SUPPLEMENT

**2:** Does menopausal hormone therapy influence cardiometabolic parameters?

Database	Search strategy
MEDLINE/PubMed	("Hormone Replacement Therapy"[Mesh] OR "Estrogen Replacement Therapy"[Mesh] OR "menopausal hormone therapy"[tiab] OR "hormone replacement therapy"[tiab] OR HRT[tiab] OR "estrogen therapy"[tiab] OR "estradiol therapy"[tiab] OR "postmenopausal hormone therapy"[tiab]) AND ("Metabolic Syndrome"[Mesh] OR "Cardiovascular Diseases"[Mesh] OR "Blood Pressure"[Mesh] OR "Insulin Resistance"[Mesh] OR "Lipid Metabolism"[Mesh] OR "Cholesterol"[Mesh] OR "Triglycerides"[Mesh] OR "Glucose Metabolism Disorders"[Mesh] OR "cardiometabolic parameters"[tiab] OR "cardiometabolic profile"[tiab] OR "insulin sensitivity"[tiab] OR "lipid profile"[tiab] OR "blood pressure"[tiab] OR "glucose levels"[tiab] OR "cholesterol levels"[tiab] OR triglycerides[tiab]) Filters applied: in the last 5 years, Randomized Controlled Trial, Systematic Review.
Embase	('hormone replacement therapy'/exp OR 'estrogen replacement therapy'/exp OR 'menopausal hormone therapy':ti, ab OR 'hormone replacement therapy':ti, ab OR HRT:ti, ab OR 'estrogen therapy':ti, ab OR 'estradiol therapy':ti, ab OR 'postmenopausal hormone therapy':ti, ab) AND ('metabolic syndrome'/exp OR 'cardiovascular disease'/exp OR 'blood pressure'/exp OR 'insulin resistance'/exp OR 'lipid metabolism'/exp OR 'cholesterol'/exp OR 'triglyceride'/exp OR 'glucose metabolism disorder'/exp OR 'cardiometabolic parameter*':ti, ab OR 'cardiometabolic profile':ti, ab OR 'insulin sensitivity':ti, ab OR 'lipid profile':ti, ab OR 'blood pressure':ti, ab OR 'glucose level*':ti, ab OR 'cholesterol level*':ti, ab OR triglyceride*:ti, ab)
Cochrane	([mh "Hormone Replacement Therapy"] OR [mh "Estrogen Replacement Therapy"] OR "menopausal hormone therapy":ti, ab OR "hormone replacement therapy":ti, ab OR HRT:ti, ab OR "estrogen therapy":ti, ab OR "estradiol therapy":ti, ab OR "postmenopausal hormone therapy":ti, ab) AND ([mh "Metabolic Syndrome"] OR [mh "Cardiovascular Diseases"] OR [mh "Blood Pressure"] OR [mh "Insulin Resistance"] OR [mh "Lipid Metabolism"] OR [mh "Cholesterol"] OR [mh "Triglycerides"] OR [mh "Glucose Metabolism Disorders"] OR "cardiometabolic parameters":ti, ab OR "cardiometabolic profile":ti, ab OR "insulin sensitivity":ti, ab OR "lipid profile":ti, ab OR "blood pressure":ti, ab OR "glucose levels":ti, ab OR "cholesterol levels":ti, ab OR triglycerides:ti, ab)
BVS	("Terapia Hormonal da Menopausa" OR "Menopausal Hormone Therapy" OR "Terapia Hormonal de la Menopausia" OR "Terapia de Reposição Hormonal" OR "Hormone Replacement Therapy" OR "Terapia de Reemplazo Hormonal" OR "Terapia Estrogênica" OR "Estrogen Therapy" OR "Terapia con Estrógenos" OR "Terapia com Estradiol" OR "Estradiol Therapy" OR "Terapia con Estradiol" OR HRT) AND ("Parâmetros Cardiometabólicos" OR "Cardiometabolic Parameters" OR "Parámetros Cardiometabólicos" OR "Perfil Cardiometabólico" OR "Cardiometabolic Profile" OR "Perfil Cardiometabólico" OR "Síndrome Metabólica" OR "Metabolic Syndrome" OR "Síndrome Metabólica" OR "Sensibilidade à Insulina" OR "Insulin Sensitivity" OR "Sensibilidad a la Insulina" OR "Perfil Lipídico" OR "Lipid Profile" OR "Perfil Lipídico" OR "Pressão Arterial" OR "Blood Pressure" OR "Presión Arterial" OR "Níveis de Glicose" OR "Glucose Levels" OR "Niveles de Glucosa" OR "Colesterol" OR "Cholesterol" OR "Colesterol" OR "Triglicerídeos" OR "Triglycerides" OR "Triglicéridos" OR "Doenças Cardiovasculares" OR "Cardiovascular Diseases" OR "Enfermedades Cardiovasculares")

## SUPPLEMENT

**3:** Do lifestyle changes improve cardiometabolic disorders in women?

Database	Search strategy
MEDLINE/PubMed	("Life Style"[Mesh] OR "Lifestyle"[tiab] OR "lifestyle change"[tiab] OR "behavior change"[tiab] OR "lifestyle intervention"[tiab] OR "behavioral intervention"[tiab]) AND ("Metabolic Syndrome"[Mesh] OR "Cardiovascular Diseases"[Mesh] OR "Insulin Resistance"[Mesh] OR "Glucose Intolerance"[Mesh] OR "Dyslipidemias"[Mesh] OR "Obesity"[Mesh] OR "Hypertension"[Mesh] OR "Type 2 Diabetes Mellitus"[Mesh] OR "Physical Activity"[Mesh] OR "Exercise"[Mesh] OR "Motor Activity"[Mesh] OR "Diet"[Mesh] OR "Nutrition Therapy"[Mesh] OR "Healthy Diet"[tiab] OR "Physical Activity"[tiab] OR exercise[tiab] OR "psychosocial intervention"[tiab] OR "smoking cessation"[tiab] OR smoking[tiab] OR tobacco[tiab] OR alcohol[tiab] OR "alcohol consumption"[tiab] OR "cardiometabolic abnormalities"[tiab] OR "cardiometabolic risk"[tiab]) AND ("Women"[Mesh] OR "Female"[Mesh] OR women[tiab] OR woman[tiab] OR female*[tiab]) Filters applied: in the last 5 years, Randomized Controlled Trial, Systematic Review.
Embase	('life style'/exp OR 'lifestyle change':ti, ab OR 'behavior change':ti, ab OR 'lifestyle intervention':ti, ab OR 'behavioral intervention':ti, ab) AND ('metabolic syndrome'/exp OR 'cardiovascular disease'/exp OR 'insulin resistance'/exp OR 'glucose intolerance'/exp OR 'dyslipidemia'/exp OR 'obesity'/exp OR 'hypertension'/exp OR 'type 2 diabetes mellitus'/exp OR 'physical activity'/exp OR 'exercise'/exp OR 'diet'/exp OR 'nutrition therapy'/exp OR 'healthy diet':ti, ab OR 'psychosocial intervention':ti, ab OR 'smoking cessation':ti, ab OR smoking:ti, ab OR tobacco:ti, ab OR alcohol:ti, ab OR 'alcohol consumption':ti, ab OR 'cardiometabolic risk':ti, ab OR 'cardiometabolic abnormalit*':ti, ab) AND ('female'/exp OR women:ti, ab OR woman:ti, ab OR female*:ti, ab)
Cochrane	("lifestyle" OR "lifestyle change" OR "lifestyle intervention" OR "behavior change" OR "behavioral intervention") AND ("cardiometabolic abnormalities" OR "cardiometabolic risk" OR "metabolic syndrome" OR "insulin resistance" OR "glucose intolerance" OR "dyslipidemia" OR "obesity" OR "hypertension" OR "type 2 diabetes" OR "cardiovascular disease" OR "physical activity" OR "exercise" OR "diet" OR "nutrition" OR "healthy diet" OR "psychosocial intervention" OR "smoking" OR "smoking cessation" OR "tobacco" OR "alcohol" OR "alcohol consumption") AND (women OR woman OR female OR females)
BVS	(TW: "Estilo de Vida" OR "Lifestyle" OR "Estilo de Vida" OR "Mudança de Estilo de Vida" OR "Lifestyle Change" OR "Cambio de Estilo de Vida" OR "Intervenção de Estilo de Vida" OR "Lifestyle Intervention" OR "Intervención de Estilo de Vida" OR "Mudança Comportamental" OR "Behavior Change" OR "Cambio de Comportamiento" OR "Intervenção Comportamental" OR "Behavioral Intervention" OR "Intervención Conductual") AND (TW: "Anormalidades Cardiometabólicas" OR "Cardiometabolic Abnormalities" OR "Anormalidades Cardiometabólicas" OR "Risco Cardiometabólico" OR "Cardiometabolic Risk" OR "Riesgo Cardiometabólico" OR "Síndrome Metabólica" OR "Metabolic Syndrome" OR "Síndrome Metabólica" OR "Resistência à Insulina" OR "Insulin Resistance" OR "Resistencia a la Insulina" OR "Intolerância à Glicose" OR "Glucose Intolerance" OR "Intolerancia a la Glucosa" OR "Dislipidemia" OR "Dyslipidemia" OR "Dislipidemia" OR "Obesidade" OR "Obesity" OR "Obesidad" OR "Hipertensão" OR "Hypertension" OR "Hipertensión" OR "Diabetes Tipo 2" OR "Type 2 Diabetes" OR "Diabetes Tipo 2" OR "Doenças Cardiovasculares" OR "Cardiovascular Diseases" OR "Enfermedades Cardiovasculares" OR "Atividade Física" OR "Physical Activity" OR "Actividad Física" OR "Exercício" OR "Exercise" OR "Ejercicio" OR "Dieta" OR "Diet" OR "Dieta" OR "Nutrição" OR "Nutrition" OR "Nutrición" OR "Dieta Saudável" OR "Healthy Diet" OR "Dieta Saludable" OR "Intervenção Psicossocial" OR "Psychosocial Intervention" OR "Intervención Psicosocial" OR "Tabagismo" OR "Smoking" OR "Tabaquismo" OR "Cessação do Tabagismo" OR "Smoking Cessation" OR "Cese del Tabaquismo" OR "Álcool" OR "Alcohol" OR "Alcohol") AND (TW: "Mulheres" OR "Women" OR "Mujeres" OR "Feminino" OR "Female" OR "Femenino")

## SUPPLEMENT

**4:** Which drugs help the treatment of cardiometabolic disorders in women with obesity and diabetes?

Database	Search strategy
MEDLINE/PubMed	("Drug Therapy"[Mesh] OR "Pharmaceutical Preparations"[Mesh] OR "Hypoglycemic Agents"[Mesh] OR "Antihyperglycemic Agents"[Mesh] OR "Antihypertensive Agents"[Mesh] OR "Lipid Regulating Agents"[Mesh] OR medication*[tiab] OR drug*[tiab] OR pharmacotherapy[tiab] OR "glucose-lowering agents"[tiab] OR "antidiabetic drugs"[tiab] OR "insulin sensitizers"[tiab] OR "weight loss drugs"[tiab]) AND (("Metabolic Syndrome"[Mesh] OR "Cardiovascular Diseases"[Mesh] OR "Insulin Resistance"[Mesh] OR "Glucose Intolerance"[Mesh] OR "Hypertension"[Mesh] OR "Dyslipidemias"[Mesh] OR "cardiometabolic risk"[tiab] OR "cardiometabolic abnormalities"[tiab] OR "insulin resistance"[tiab] OR "metabolic syndrome"[tiab] OR "blood pressure"[tiab] OR "glucose intolerance"[tiab] OR cholesterol[tiab] OR "lipid profile"[tiab]) AND ("Women"[Mesh] OR "Female"[Mesh] OR women[tiab] OR woman[tiab] OR female*[tiab])) AND ("Obesity"[Mesh] OR "Obesity, Morbid"[Mesh] OR "Diabetes Mellitus, Type 2"[Mesh] OR obesity[tiab] OR overweight[tiab] OR "morbid obesity"[tiab] OR "type 2 diabetes"[tiab] OR T2DM[tiab]) Filters applied: in the last 5 years, Randomized Controlled Trial, Systematic Review.
Embase	('drug therapy'/exp OR 'pharmaceutical preparation'/exp OR 'hypoglycemic agent'/exp OR 'antihyperglycemic agent'/exp OR 'antihypertensive agent'/exp OR 'lipid regulating agent'/exp OR medication*:ti, ab OR drug*:ti, ab OR pharmacotherapy:ti, ab OR 'glucose lowering agent':ti, ab OR 'antidiabetic drug':ti, ab OR 'insulin sensitizer':ti, ab OR 'weight loss drug':ti, ab) AND (('metabolic syndrome'/exp OR 'cardiovascular disease'/exp OR 'insulin resistance'/exp OR 'glucose intolerance'/exp OR 'hypertension'/exp OR 'dyslipidemia'/exp OR 'cardiometabolic risk':ti, ab OR 'cardiometabolic abnormalit*':ti, ab OR 'blood pressure':ti, ab OR cholesterol:ti, ab OR 'lipid profile':ti, ab) AND ('female'/exp OR women:ti, ab OR woman:ti, ab OR female*:ti, ab)) AND ('obesity'/exp OR 'morbid obesity'/exp OR 'type 2 diabetes mellitus'/exp OR obesity:ti, ab OR overweight:ti, ab OR 'morbid obesity':ti, ab OR 'type 2 diabetes':ti, ab OR T2DM:ti, ab)
Cochrane	([mh "Drug Therapy"] OR [mh "Pharmaceutical Preparations"] OR [mh "Hypoglycemic Agents"] OR [mh "Antihyperglycemic Agents"] OR [mh "Antihypertensive Agents"] OR [mh "Lipid Regulating Agents"] OR medication*:ti, ab OR drug*:ti, ab OR pharmacotherapy:ti, ab OR "glucose-lowering agents":ti, ab OR "antidiabetic drugs":ti, ab OR "insulin sensitizers":ti, ab OR "weight loss drugs":ti, ab) AND (([mh "Metabolic Syndrome"] OR [mh "Cardiovascular Diseases"] OR [mh "Insulin Resistance"] OR [mh "Glucose Intolerance"] OR [mh "Hypertension"] OR [mh "Dyslipidemias"] OR "cardiometabolic risk":ti, ab OR "cardiometabolic abnormalities":ti, ab OR "insulin resistance":ti, ab OR "metabolic syndrome":ti, ab OR "blood pressure":ti, ab OR "glucose intolerance":ti, ab OR cholesterol:ti, ab OR "lipid profile":ti, ab) AND ([mh "Women"] OR [mh "Female"] OR women:ti, ab OR woman:ti, ab OR female*:ti, ab)) AND ([mh "Obesity"] OR [mh "Obesity, Morbid"] OR [mh "Diabetes Mellitus, Type 2"] OR obesity:ti, ab OR overweight:ti, ab OR "morbid obesity":ti, ab OR "type 2 diabetes":ti, ab OR T2DM:ti, ab)
BVS	(TW: "Medicamentos" OR "Drugs" OR "Medicamentos" OR "Terapia Medicamentosa" OR "Drug Therapy" OR "Tratamiento Farmacológico" OR "Preparações Farmacêuticas" OR "Pharmaceutical Preparations" OR "Preparaciones Farmacéuticas" OR "Agentes Hipoglicemiantes" OR "Hypoglycemic Agents" OR "Agentes Hipoglucemiantes" OR "Agentes Antihipertensivos" OR "Antihypertensive Agents" OR "Agentes Antihipertensivos" OR "Agentes Reguladores de Lipídios" OR "Lipid Regulating Agents" OR "Agentes Reguladores de Lípidos" OR "Sensibilizadores de Insulina" OR "Insulin Sensitizers" OR "Sensibilizadores de Insulina" OR "Medicamentos para Emagrecimento" OR "Weight Loss Drugs" OR "Medicamentos para Adelgazar") AND (TW: "Anormalidades Cardiometabólicas" OR "Cardiometabolic Abnormalities" OR "Anormalidades Cardiometabólicas" OR "Risco Cardiometabólico" OR "Cardiometabolic Risk" OR "Riesgo Cardiometabólico" OR "Síndrome Metabólica" OR "Metabolic Syndrome" OR "Síndrome Metabólica" OR "Resistência à Insulina" OR "Insulin Resistance" OR "Resistencia a la Insulina" OR "Dislipidemia" OR "Dyslipidemia" OR "Dislipidemia" OR "Hipertensão" OR "Hypertension" OR "Hipertensión" OR "Diabetes Tipo 2" OR "Type 2 Diabetes" OR "Diabetes Tipo 2" OR "Obesidade" OR "Obesity" OR "Obesidad" OR "Sobrepeso" OR "Overweight" OR "Sobrepeso" OR "Doenças Cardiovasculares" OR "Cardiovascular Diseases" OR "Enfermedades Cardiovasculares") AND (TW: "Mulheres" OR "Women" OR "Mujeres" OR "Feminino" OR "Female" OR "Femenino")

## SUPPLEMENT

**5:** Does the treatment of infertility associate with cardiometabolic disorders in women?

Database	Search strategy
MEDLINE/PubMed	("Infertility, Female"[Mesh] OR "Infertility"[Mesh] OR infertility[tiab] OR "fertility treatment*"[tiab] OR "Assisted Reproductive Techniques"[Mesh] OR "assisted reproductive technolog*"[tiab] OR ART[tiab] OR "In Vitro Fertilization"[Mesh] OR "in vitro fertilization"[tiab] OR IVF[tiab] OR "Intracytoplasmic Sperm Injection"[Mesh] OR ICSI[tiab] OR "Ovulation Induction"[Mesh] OR "ovulation induction"[tiab] OR clomiphene[tiab] OR letrozole[tiab] OR gonadotropin*[tiab]) AND ("Cardiometabolic Diseases"[Mesh] OR "Metabolic Syndrome"[Mesh] OR "Insulin Resistance"[Mesh] OR "Dyslipidemias"[Mesh] OR "Hypertension"[Mesh] OR "Obesity"[Mesh] OR "Diabetes Mellitus, Type 2"[Mesh] OR cardiometabolic[tiab] OR "cardio-metabolic"[tiab] OR "metabolic syndrome"[tiab] OR "insulin resistance"[tiab] OR dyslipidemia[tiab] OR hyperlipidemia[tiab] OR hypertension[tiab] OR "blood pressure"[tiab] OR obesity[tiab] OR overweight[tiab] OR BMI[tiab] OR "body mass index"[tiab] OR "type 2 diabetes"[tiab] OR diabetes[tiab]) AND ("Women"[Mesh] OR "Female"[Mesh] OR women[tiab] OR woman[tiab] OR female*[tiab]) Filters applied: in the last 5 years, Randomized Controlled Trial, Systematic Review.
Embase	('female infertility treatment'/exp OR 'infertility'/exp OR 'assisted reproductive technology'/exp OR 'in vitro fertilization'/exp OR 'intracytoplasmic sperm injection'/exp OR 'ovulation induction'/exp OR infertility:ti, ab OR 'fertility treatment*':ti, ab OR 'assisted reproductive technolog*':ti, ab OR ART:ti, ab OR 'in vitro fertilization':ti, ab OR IVF:ti, ab OR 'intracytoplasmic sperm injection':ti, ab OR ICSI:ti, ab OR 'ovulation induction':ti, ab OR clomiphene:ti, ab OR letrozole:ti, ab OR gonadotropin*:ti, ab) AND ('cardiometabolic disorder'/exp OR 'metabolic syndrome'/exp OR 'insulin resistance'/exp OR 'dyslipidemia'/exp OR 'hypertension'/exp OR 'obesity'/exp OR 'type 2 diabetes mellitus'/exp OR cardiometabolic:ti, ab OR 'cardio metabolic':ti, ab OR dyslipidemia*:ti, ab OR hyperlipidemia:ti, ab OR hypertension:ti, ab OR 'blood pressure':ti, ab OR BMI:ti, ab OR obesity:ti, ab OR overweight:ti, ab OR diabetes:ti, ab OR 'type 2 diabetes':ti, ab) AND ('female'/exp OR women:ti, ab OR woman:ti, ab OR female*:ti, ab)
Cochrane	([mh "Infertility, Female"] OR [mh "Infertility"] OR infertility:ti, ab OR "fertility treatment*":ti, ab OR [mh "Assisted Reproductive Techniques"] OR "assisted reproductive technolog*":ti, ab OR ART:ti, ab OR [mh "In Vitro Fertilization"] OR "in vitro fertilization":ti, ab OR IVF:ti, ab OR [mh "Intracytoplasmic Sperm Injection"] OR ICSI:ti, ab OR [mh "Ovulation Induction"] OR "ovulation induction":ti, ab OR clomiphene:ti, ab OR letrozole:ti, ab OR gonadotropin*:ti, ab) AND ([mh "Cardiometabolic Diseases"] OR [mh "Metabolic Syndrome"] OR [mh "Insulin Resistance"] OR [mh "Dyslipidemias"] OR [mh "Hypertension"] OR [mh "Obesity"] OR [mh "Diabetes Mellitus, Type 2"] OR cardiometabolic:ti, ab OR "cardio-metabolic":ti, ab OR "metabolic syndrome":ti, ab OR "insulin resistance":ti, ab OR dyslipidemia:ti, ab OR hyperlipidemia:ti, ab OR hypertension:ti, ab OR "blood pressure":ti, ab OR obesity:ti, ab OR overweight:ti, ab OR BMI:ti, ab OR "body mass index":ti, ab OR "type 2 diabetes":ti, ab OR diabetes:ti, ab) AND ([mh "Women"] OR [mh "Female"] OR women:ti, ab OR woman:ti, ab OR female*:ti, ab)
BVS	("Infertilidade" OR "Infertility" OR "Infertilidad" OR "Tratamento da Infertilidade" OR "Infertility Treatment" OR "Tratamiento de la Infertilidad" OR "Tecnologias de Reprodução Assistida" OR "Assisted Reproductive Technologies" OR "Tecnologías de Reproducción Asistida" OR "Fertilização in vitro" OR "In Vitro Fertilization" OR "Fertilización in vitro" OR "Injeção Intracitoplasmática de Espermatozoides" OR "Intracytoplasmic Sperm Injection" OR "Inyección intracitoplasmática de espermatozoides" OR "Indução da Ovulação" OR "Ovulation Induction" OR "Inducción de la Ovulación" OR Clomifeno OR Clomiphene OR Clomifeno OR Letrozol OR Letrozole OR Letrozol OR Gonadotrofinas OR Gonadotropins OR Gonadotropinas) AND ("Anormalidades Cardiometabólicas" OR "Cardiometabolic Abnormalities" OR "Anormalidades Cardiometabólicas" OR "Risco Cardiometabólico" OR "Cardiometabolic Risk" OR "Riesgo Cardiometabólico" OR "Síndrome Metabólica" OR "Metabolic Syndrome" OR "Síndrome Metabólica" OR "Resistência à Insulina" OR "Insulin Resistance" OR "Resistencia a la Insulina" OR "Dislipidemia" OR "Dyslipidemia" OR "Dislipidemia" OR "Hipertensão" OR "Hypertension" OR "Hipertensión" OR "Obesidade" OR "Obesity" OR "Obesidad" OR "Sobrepeso" OR "Overweight" OR "Sobrepeso" OR "Diabetes Tipo 2" OR "Type 2 Diabetes" OR "Diabetes Tipo 2" OR "Doenças Cardiovasculares" OR "Cardiovascular Diseases" OR "Enfermedades Cardiovasculares") AND ("Mulheres" OR "Women" OR "Mujeres" OR "Feminino" OR "Female" OR "Femenino")

## SUPPLEMENT

**6:** Which drugs for the treatment of hypertension and dyslipidemia influence cardiometabolic disorders in women?

Database	Search strategy
MEDLINE/PubMed	("Drug Therapy"[Mesh] OR "Drugs"[Mesh] OR "Antihypertensive Agents"[Mesh] OR "Hypolipidemic Agents"[Mesh] OR medication*[tiab] OR drug*[tiab] OR "pharmacologic treatment"[tiab] OR pharmacotherapy[tiab]) AND ("Hypertension"[Mesh] OR "Antihypertensive Agents"[Mesh] OR hypertension[tiab] OR "high blood pressure"[tiab] OR "blood pressure control"[tiab] OR "Dyslipidemias"[Mesh] OR "Hyperlipidemias"[Mesh] OR dyslipidemia[tiab] OR hyperlipidemia[tiab] OR "lipid-lowering"[tiab] OR statins[tiab] OR "ACE inhibitors"[tiab] OR ARBs[tiab] OR "beta blockers"[tiab] OR diuretics[tiab] OR "calcium channel blockers"[tiab]) AND ("Cardiometabolic Diseases"[Mesh] OR "Metabolic Syndrome"[Mesh] OR "Insulin Resistance"[Mesh] OR "Diabetes Mellitus, Type 2"[Mesh] OR "Obesity"[Mesh] OR cardiometabolic[tiab] OR "metabolic syndrome"[tiab] OR "insulin resistance"[tiab] OR "glucose intolerance"[tiab] OR hyperglycemia[tiab] OR "type 2 diabetes"[tiab] OR obesity[tiab] OR overweight[tiab] OR "cardiovascular risk"[tiab]) AND ("Women"[Mesh] OR "Female"[Mesh] OR women[tiab] OR woman[tiab] OR female*[tiab]) Filters applied: in the last 5 years, Randomized Controlled Trial, Systematic Review.
Embase	('drug therapy'/exp OR 'drug'/exp OR 'antihypertensive agent'/exp OR 'hypolipidemic agent'/exp OR medication*:ti, ab OR drug*:ti, ab OR 'pharmacologic treatment':ti, ab OR pharmacotherapy:ti, ab) AND ('hypertension'/exp OR 'antihypertensive agent'/exp OR hypertension:ti, ab OR 'high blood pressure':ti, ab OR 'blood pressure control':ti, ab OR 'dyslipidemia'/exp OR 'hyperlipidemia'/exp OR dyslipidemia:ti, ab OR hyperlipidemia:ti, ab OR 'lipid lowering':ti, ab OR statins:ti, ab OR 'ACE inhibitors':ti, ab OR ARBs:ti, ab OR 'beta blockers':ti, ab OR diuretics:ti, ab OR 'calcium channel blockers':ti, ab) AND ('cardiometabolic disorder'/exp OR 'metabolic syndrome'/exp OR 'insulin resistance'/exp OR 'type 2 diabetes mellitus'/exp OR 'obesity'/exp OR cardiometabolic:ti, ab OR 'metabolic syndrome':ti, ab OR 'insulin resistance':ti, ab OR 'glucose intolerance':ti, ab OR hyperglycemia:ti, ab OR 'type 2 diabetes':ti, ab OR obesity:ti, ab OR overweight:ti, ab OR 'cardiovascular risk':ti, ab) AND ('female'/exp OR women:ti, ab OR woman:ti, ab OR female*:ti, ab)
Cochrane	([mh "Drug Therapy"] OR [mh "Drugs"] OR [mh "Antihypertensive Agents"] OR [mh "Hypolipidemic Agents"] OR medication*:ti, ab OR drug*:ti, ab OR "pharmacologic treatment":ti, ab OR pharmacotherapy:ti, ab) AND ([mh "Hypertension"] OR [mh "Antihypertensive Agents"] OR hypertension:ti, ab OR "high blood pressure":ti, ab OR "blood pressure control":ti, ab OR [mh "Dyslipidemias"] OR [mh "Hyperlipidemias"] OR dyslipidemia:ti, ab OR hyperlipidemia:ti, ab OR "lipid-lowering":ti, ab OR statins:ti, ab OR "ACE inhibitors":ti, ab OR ARBs:ti, ab OR "beta blockers":ti, ab OR diuretics:ti, ab OR "calcium channel blockers":ti, ab) AND ([mh "Cardiometabolic Diseases"] OR [mh "Metabolic Syndrome"] OR [mh "Insulin Resistance"] OR [mh "Diabetes Mellitus, Type 2"] OR [mh "Obesity"] OR cardiometabolic:ti, ab OR "metabolic syndrome":ti, ab OR "insulin resistance":ti, ab OR "glucose intolerance":ti, ab OR hyperglycemia:ti, ab OR "type 2 diabetes":ti, ab OR obesity:ti, ab OR overweight:ti, ab OR "cardiovascular risk":ti, ab) AND ([mh "Women"] OR [mh "Female"] OR women:ti, ab OR woman:ti, ab OR female*:ti, ab)
BVS	(TW: "Medicamentos" OR "Drugs" OR "Medicamentos" OR "Terapia Medicamentosa" OR "Drug Therapy" OR "Tratamiento Farmacológico" OR "Agentes Anti-hipertensivos" OR "Antihypertensive Agents" OR "Agentes Antihipertensivos" OR "Agentes Hipolipemiantes" OR "Hypolipidemic Agents" OR "Agentes Hipolipemiantes" OR "Tratamento Farmacológico" OR "Pharmacologic Treatment" OR "Tratamiento Farmacológico") AND TW: ("Hipertensão" OR "Hypertension" OR "Hipertensión" OR "Pressão Alta" OR "High Blood Pressure" OR "Presión Alta" OR "Controle da Pressão Arterial" OR "Blood Pressure Control" OR "Control de la Presión Arterial" OR "Dislipidemia" OR "Dyslipidemia" OR "Dislipidemia" OR "Hiperlipidemia" OR "Hyperlipidemia" OR "Hiperlipidemia" OR "Redução de Lipídios" OR "Lipid-Lowering" OR "Reducción de Lípidos" OR Estatinas OR Statins OR Estatinas OR "Inibidores da ECA" OR "ACE Inhibitors" OR "Inhibidores de la ECA" OR BRA OR ARBs OR BRA OR "Betabloqueadores" OR "Beta Blockers" OR "Betabloqueadores" OR Diuréticos OR Diuretics OR Diuréticos OR "Bloqueadores dos Canais de Cálcio" OR "Calcium Channel Blockers" OR "Bloqueadores de los Canales de Calcio") AND (TW: "Anormalidades Cardiometabólicas" OR "Cardiometabolic Disorders" OR "Trastornos Cardiometabólicos" OR "Síndrome Metabólica" OR "Metabolic Syndrome" OR "Síndrome Metabólica" OR "Resistência à Insulina" OR "Insulin Resistance" OR "Resistencia a la Insulina" OR "Diabetes Tipo 2" OR "Type 2 Diabetes" OR "Diabetes Tipo 2" OR "Obesidade" OR "Obesity" OR "Obesidad" OR "Sobrepeso" OR "Overweight" OR "Sobrepeso" OR "Risco Cardiovascular" OR "Cardiovascular Risk" OR "Riesgo Cardiovascular") AND (TW: "Mulheres" OR "Women" OR "Mujeres" OR "Feminino" OR "Female")

## SUPPLEMENT

**7:** Should bariatric surgery be indicated for the treatment of cardiometabolic disorders in women?

Database	Search strategy
MEDLINE/PubMed	("Bariatric Surgery"[Mesh] OR "Gastric Bypass"[Mesh] OR "Gastrectomy"[Mesh] OR "bariatric surgery"[tiab] OR "gastric bypass"[tiab] OR "sleeve gastrectomy"[tiab] OR "Roux-en-Y"[tiab] OR "metabolic surgery"[tiab] OR "weight loss surgery"[tiab]) AND ("Cardiometabolic Diseases"[Mesh] OR "Metabolic Syndrome"[Mesh] OR "Insulin Resistance"[Mesh] OR "Diabetes Mellitus, Type 2"[Mesh] OR "Obesity"[Mesh] OR "Dyslipidemias"[Mesh] OR "Hypertension"[Mesh] OR cardiometabolic[tiab] OR "metabolic syndrome"[tiab] OR "insulin resistance"[tiab] OR "type 2 diabetes"[tiab] OR hyperglycemia[tiab] OR dyslipidemia[tiab] OR hyperlipidemia[tiab] OR hypertension[tiab] OR "blood pressure"[tiab] OR "cardiovascular risk"[tiab]) AND ("Women"[Mesh] OR "Female"[Mesh] OR women[tiab] OR woman[tiab] OR female*[tiab]) Filters applied: in the last 5 years, Randomized Controlled Trial, Systematic Review.
Embase	('bariatric surgery'/exp OR 'gastric bypass'/exp OR 'gastrectomy'/exp OR 'bariatric surgery':ti, ab OR 'gastric bypass':ti, ab OR 'sleeve gastrectomy':ti, ab OR 'roux-en-y':ti, ab OR 'metabolic surgery':ti, ab OR 'weight loss surgery':ti, ab) AND ('cardiometabolic disorder'/exp OR 'metabolic syndrome'/exp OR 'insulin resistance'/exp OR 'type 2 diabetes mellitus'/exp OR 'obesity'/exp OR 'dyslipidemia'/exp OR 'hypertension'/exp OR cardiometabolic:ti, ab OR 'metabolic syndrome':ti, ab OR 'insulin resistance':ti, ab OR 'type 2 diabetes':ti, ab OR hyperglycemia:ti, ab OR dyslipidemia:ti, ab OR hyperlipidemia:ti, ab OR hypertension:ti, ab OR 'blood pressure':ti, ab OR 'cardiovascular risk':ti, ab) AND ('female'/exp OR women:ti, ab OR woman:ti, ab OR female*:ti, ab)
Cochrane	([mh "Bariatric Surgery"] OR [mh "Gastric Bypass"] OR [mh "Gastrectomy"] OR "bariatric surgery":ti, ab OR "gastric bypass":ti, ab OR "sleeve gastrectomy":ti, ab OR "Roux-en-Y":ti, ab OR "metabolic surgery":ti, ab OR "weight loss surgery":ti, ab) AND ([mh "Cardiometabolic Diseases"] OR [mh "Metabolic Syndrome"] OR [mh "Insulin Resistance"] OR [mh "Diabetes Mellitus, Type 2"] OR [mh "Obesity"] OR [mh "Dyslipidemias"] OR [mh "Hypertension"] OR cardiometabolic:ti, ab OR "metabolic syndrome":ti, ab OR "insulin resistance":ti, ab OR "type 2 diabetes":ti, ab OR hyperglycemia:ti, ab OR dyslipidemia:ti, ab OR hyperlipidemia:ti, ab OR hypertension:ti, ab OR "blood pressure":ti, ab OR "cardiovascular risk":ti, ab) AND ([mh "Women"] OR [mh "Female"] OR women:ti, ab OR woman:ti, ab OR female*:ti, ab)
BVS	(TW: "Cirurgia Bariátrica" OR "Bariatric Surgery" OR "Cirugía Bariátrica" OR "Bypass Gástrico" OR "Gastric Bypass" OR "Bypass Gástrico" OR "Gastrectomia Vertical" OR "Sleeve Gastrectomy" OR "Gastrectomía Vertical" OR "Roux-en-Y" OR "Roux-en-Y" OR "Cirurgia Metabólica" OR "Metabolic Surgery" OR "Cirugía Metabólica" OR "Cirurgia para Perda de Peso" OR "Weight Loss Surgery" OR "Cirugía para Pérdida de Peso") AND (TW: "Anormalidades Cardiometabólicas" OR "Cardiometabolic Abnormalities" OR "Anormalidades Cardiometabólicas" OR "Risco Cardiometabólico" OR "Cardiometabolic Risk" OR "Riesgo Cardiometabólico" OR "Síndrome Metabólica" OR "Metabolic Syndrome" OR "Síndrome Metabólica" OR "Resistência à Insulina" OR "Insulin Resistance" OR "Resistencia a la Insulina" OR "Diabetes Tipo 2" OR "Type 2 Diabetes" OR "Diabetes Tipo 2" OR "Obesidade" OR "Obesity" OR "Obesidad" OR "Dislipidemia" OR "Dyslipidemia" OR "Dislipidemia" OR "Hipertensão" OR "Hypertension" OR "Hipertensión" OR "Risco Cardiovascular" OR "Cardiovascular Risk" OR "Riesgo Cardiovascular") AND (TW: "Mulheres" OR "Women" OR "Mujeres" OR "Feminino" OR "Female")

## SUPPLEMENT

**8:** Which interventions improve cardiometabolic disorders in women with chronic kidney disease?

Database	Search strategy
MEDLINE/PubMed	("Life Style"[Mesh] OR "Exercise"[Mesh] OR "Diet Therapy"[Mesh] OR "Drug Therapy"[Mesh] OR intervention*[tiab] OR treatment*[tiab] OR therapy[tiab] OR management[tiab] OR lifestyle[tiab] OR diet[tiab] OR nutrition[tiab] OR "physical activity"[tiab] OR exercise[tiab] OR medication*[tiab] OR pharmacologic*[tiab]) AND ("Cardiometabolic Diseases"[Mesh] OR "Metabolic Syndrome"[Mesh] OR "Insulin Resistance"[Mesh] OR "Glucose Intolerance"[Mesh] OR "Dyslipidemias"[Mesh] OR "Hypertension"[Mesh] OR "Type 2 Diabetes Mellitus"[Mesh] OR "Obesity"[Mesh] OR cardiometabolic[tiab] OR "metabolic syndrome"[tiab] OR "insulin resistance"[tiab] OR "glucose intolerance"[tiab] OR dyslipidemia[tiab] OR hyperglycemia[tiab] OR "type 2 diabetes"[tiab] OR hypertension[tiab] OR "cardiovascular risk"[tiab]) AND ("Renal Insufficiency, Chronic"[Mesh] OR "Kidney Diseases"[Mesh] OR "chronic kidney disease"[tiab] OR CKD[tiab] OR "chronic renal failure"[tiab] OR "end-stage renal disease"[tiab] OR ESRD[tiab] OR "kidney dysfunction"[tiab]) AND ("Women"[Mesh] OR "Female"[Mesh] OR women[tiab] OR woman[tiab] OR female*[tiab]) Filters applied: in the last 5 years, Randomized Controlled Trial, Systematic Review.
Embase	('life style'/exp OR 'exercise'/exp OR 'diet therapy'/exp OR 'drug therapy'/exp OR intervention*:ti, ab OR treatment*:ti, ab OR therapy:ti, ab OR management:ti, ab OR lifestyle:ti, ab OR diet:ti, ab OR nutrition:ti, ab OR 'physical activity':ti, ab OR exercise:ti, ab OR medication*:ti, ab OR pharmacologic*:ti, ab) AND ('cardiometabolic disorder'/exp OR 'metabolic syndrome'/exp OR 'insulin resistance'/exp OR 'dyslipidemia'/exp OR 'hypertension'/exp OR 'type 2 diabetes mellitus'/exp OR 'obesity'/exp OR cardiometabolic:ti, ab OR 'metabolic syndrome':ti, ab OR 'insulin resistance':ti, ab OR hyperglycemia:ti, ab OR 'cardiovascular risk':ti, ab) AND ('chronic kidney disease'/exp OR 'renal insufficiency, chronic'/exp OR 'chronic kidney disease':ti, ab OR CKD:ti, ab OR 'chronic renal failure':ti, ab OR 'end stage renal disease':ti, ab OR ESRD:ti, ab OR 'kidney dysfunction':ti, ab OR 'kidney disease':ti, ab) AND ('female'/exp OR women:ti, ab OR woman:ti, ab OR female*:ti, ab)
Cochrane	([mh "Life Style"] OR [mh "Exercise"] OR [mh "Diet Therapy"] OR [mh "Drug Therapy"] OR intervention*:ti, ab OR treatment*:ti, ab OR therapy:ti, ab OR management:ti, ab OR lifestyle:ti, ab OR diet:ti, ab OR nutrition:ti, ab OR "physical activity":ti, ab OR exercise:ti, ab OR medication*:ti, ab OR pharmacologic*:ti, ab) AND ([mh "Cardiometabolic Diseases"] OR [mh "Metabolic Syndrome"] OR [mh "Insulin Resistance"] OR [mh "Glucose Intolerance"] OR [mh "Dyslipidemias"] OR [mh "Hypertension"] OR [mh "Type 2 Diabetes Mellitus"] OR [mh "Obesity"] OR cardiometabolic:ti, ab OR "metabolic syndrome":ti, ab OR "insulin resistance":ti, ab OR "glucose intolerance":ti, ab OR dyslipidemia:ti, ab OR hyperglycemia:ti, ab OR "type 2 diabetes":ti, ab OR hypertension:ti, ab OR "cardiovascular risk":ti, ab) AND ([mh "Renal Insufficiency, Chronic"] OR [mh "Kidney Diseases"] OR "chronic kidney disease":ti, ab OR CKD:ti, ab OR "chronic renal failure":ti, ab OR "end-stage renal disease":ti, ab OR ESRD:ti, ab OR "kidney dysfunction":ti, ab) AND ([mh "Women"] OR [mh "Female"] OR women:ti, ab OR woman:ti, ab OR female*:ti, ab)
BVS	(TW: "Intervenções" OR "Interventions" OR "Intervenciones" OR "Tratamento" OR "Treatment" OR "Tratamiento" OR "Terapia" OR "Therapy" OR "Terapia" OR "Manejo" OR "Management" OR "Manejo" OR "Estilo de Vida" OR "Lifestyle" OR "Estilo de Vida" OR "Exercício" OR "Exercise" OR "Ejercicio" OR "Atividade Física" OR "Physical Activity" OR "Actividad Física" OR "Dieta" OR "Diet" OR "Dieta" OR "Nutrição" OR "Nutrition" OR "Nutrición" OR "Terapia Nutricional" OR "Nutrition Therapy" OR "Terapia Nutricional" OR "Medicamentos" OR "Medication" OR "Medicamentos" OR "Tratamento Farmacológico" OR "Pharmacologic Treatment" OR "Tratamiento Farmacológico") AND (TW: "Doença Renal Crônica" OR "Chronic Kidney Disease" OR "Enfermedad Renal Crónica" OR "Insuficiência Renal Crônica" OR "Chronic Renal Failure" OR "Insuficiencia Renal Crónica" OR "Doença Renal Terminal" OR "End-Stage Renal Disease" OR "Enfermedad Renal Terminal" OR CKD OR ERC OR "Disfunção Renal" OR "Kidney Dysfunction" OR "Disfunción Renal" OR "Doença Renal" OR "Kidney Disease" OR "Enfermedad Renal") AND (TW: "Anormalidades Cardiometabólicas" OR "Cardiometabolic Disorders" OR "Trastornos Cardiometabólicos") AND ("Mulheres" OR "Women" OR "Mujeres")

## SUPPLEMENT

**9:** Which interventions improve cardiometabolic disorders in women Metabolic Dysfunction–Associated Steatotic Liver Disease (MASLD)?

Database	Search strategy
MEDLINE/PubMed	("Life Style"[Mesh] OR "Exercise"[Mesh] OR "Diet Therapy"[Mesh] OR "Drug Therapy"[Mesh] OR intervention*[tiab] OR treatment*[tiab] OR therapy[tiab] OR management[tiab] OR lifestyle[tiab] OR diet[tiab] OR nutrition[tiab] OR "physical activity"[tiab] OR exercise[tiab] OR medication*[tiab] OR pharmacologic*[tiab]) AND ("Cardiometabolic Diseases"[Mesh] OR "Metabolic Syndrome"[Mesh] OR "Insulin Resistance"[Mesh] OR "Glucose Intolerance"[Mesh] OR "Dyslipidemias"[Mesh] OR "Hypertension"[Mesh] OR "Type 2 Diabetes Mellitus"[Mesh] OR cardiometabolic[tiab] OR "metabolic syndrome"[tiab] OR "insulin resistance"[tiab] OR "glucose intolerance"[tiab] OR dyslipidemia[tiab] OR hyperglycemia[tiab] OR "type 2 diabetes"[tiab] OR hypertension[tiab] OR "cardiovascular risk"[tiab]) AND ("Non-alcoholic Fatty Liver Disease"[Mesh] OR "Fatty Liver"[Mesh] OR MASLD[tiab] OR "metabolic dysfunction-associated steatotic liver disease"[tiab] OR NAFLD[tiab] OR "nonalcoholic fatty liver disease"[tiab] OR "fatty liver"[tiab] OR "hepatic steatosis"[tiab] OR steatosis[tiab] OR steatohepatitis[tiab] OR NASH[tiab]) AND ("Women"[Mesh] OR "Female"[Mesh] OR women[tiab] OR woman[tiab] OR female*[tiab]) Filters applied: in the last 5 years, Randomized Controlled Trial, Systematic Review.
Embase	('life style'/exp OR 'exercise'/exp OR 'diet therapy'/exp OR 'drug therapy'/exp OR intervention*:ti, ab OR treatment*:ti, ab OR therapy:ti, ab OR management:ti, ab OR lifestyle:ti, ab OR diet:ti, ab OR nutrition:ti, ab OR 'physical activity':ti, ab OR exercise:ti, ab OR medication*:ti, ab OR pharmacologic*:ti, ab) AND ('cardiometabolic disorder'/exp OR 'metabolic syndrome'/exp OR 'insulin resistance'/exp OR 'dyslipidemia'/exp OR 'hypertension'/exp OR 'type 2 diabetes mellitus'/exp OR cardiometabolic:ti, ab OR 'metabolic syndrome':ti, ab OR 'insulin resistance':ti, ab OR hyperglycemia:ti, ab OR 'cardiovascular risk':ti, ab) AND ('nonalcoholic fatty liver'/exp OR 'fatty liver'/exp OR MASLD:ti, ab OR 'metabolic dysfunction associated steatotic liver disease':ti, ab OR NAFLD:ti, ab OR 'nonalcoholic fatty liver disease':ti, ab OR 'fatty liver':ti, ab OR 'hepatic steatosis':ti, ab OR steatosis:ti, ab OR steatohepatitis:ti, ab OR NASH:ti, ab) AND ('female'/exp OR women:ti, ab OR woman:ti, ab OR female*:ti, ab)
Cochrane	([mh "Life Style"] OR [mh "Exercise"] OR [mh "Diet Therapy"] OR [mh "Drug Therapy"] OR intervention*:ti, ab OR treatment*:ti, ab OR therapy:ti, ab OR management:ti, ab OR lifestyle:ti, ab OR diet:ti, ab OR nutrition:ti, ab OR "physical activity":ti, ab OR exercise:ti, ab OR medication*:ti, ab OR pharmacologic*:ti, ab) AND ([mh "Cardiometabolic Diseases"] OR [mh "Metabolic Syndrome"] OR [mh "Insulin Resistance"] OR [mh "Glucose Intolerance"] OR [mh "Dyslipidemias"] OR [mh "Hypertension"] OR [mh "Type 2 Diabetes Mellitus"] OR cardiometabolic:ti, ab OR "metabolic syndrome":ti, ab OR "insulin resistance":ti, ab OR "glucose intolerance":ti, ab OR dyslipidemia:ti, ab OR hyperglycemia:ti, ab OR "type 2 diabetes":ti, ab OR hypertension:ti, ab OR "cardiovascular risk":ti, ab) AND ([mh "Non-alcoholic Fatty Liver Disease"] OR [mh "Fatty Liver"] OR MASLD:ti, ab OR "metabolic dysfunction-associated steatotic liver disease":ti, ab OR NAFLD:ti, ab OR "nonalcoholic fatty liver disease":ti, ab OR "fatty liver":ti, ab OR "hepatic steatosis":ti, ab OR steatosis:ti, ab OR steatohepatitis:ti, ab OR NASH:ti, ab) AND ([mh "Women"] OR [mh "Female"] OR women:ti, ab OR woman:ti, ab OR female*:ti, ab)
BVS	(TW: "Intervenções" OR "Interventions" OR "Intervenciones" OR "Tratamento" OR "Treatment" OR "Tratamiento" OR "Terapia" OR "Therapy" OR "Terapia" OR "Manejo" OR "Management" OR "Manejo" OR "Estilo de Vida" OR "Lifestyle" OR "Estilo de Vida" OR "Exercício" OR "Exercise" OR "Ejercicio" OR "Atividade Física" OR "Physical Activity" OR "Actividad Física" OR "Dieta" OR "Diet" OR "Dieta" OR "Nutrição" OR "Nutrition" OR "Nutrición" OR "Terapia Nutricional" OR "Nutrition Therapy" OR "Terapia Nutricional" OR "Medicamentos" OR "Medication" OR "Medicamentos" OR "Tratamento Farmacológico" OR "Pharmacologic Treatment" OR "Tratamiento Farmacológico") AND (TW: "Doença Hepática Esteatótica Metabólica" OR "Metabolic Dysfunction-Associated Steatotic Liver Disease" OR "Enfermedad Hepática Esteatótica Metabólica" OR MASLD OR "Doença Hepática Gordurosa Não Alcoólica" OR "Nonalcoholic Fatty Liver Disease" OR "Enfermedad Hepática Grasa No Alcohólica" OR NAFLD OR "Esteatose Hepática" OR "Fatty Liver" OR "Esteatosis Hepática" OR "Esteato-hepatite" OR "Steatohepatitis" OR "Esteatohepatitis" OR NASH) AND (TW: "Anormalidades Cardiometabólicas" OR "Cardiometabolic Disorders" OR "Trastornos Cardiometabólicos") AND ("Mulheres" OR "Women" OR "Mujeres")

## SUPPLEMENT

**10:** Do women with endometriosis and polycystic ovary syndrome need to check their cardiovascular risk factors regularly to prevent cardiovascular diseases?

Database	Search strategy
MEDLINE/PubMed	("Endometriosis"[Mesh] OR endometriosis[tiab]) OR ("Polycystic Ovary Syndrome"[Mesh] OR "Stein-Leventhal Syndrome"[Mesh] OR "ovarian dysfunction"[tiab] OR PCOS[tiab] OR "polycystic ovary syndrome"[tiab]) AND ("Risk Factors"[Mesh] OR "Cardiovascular Risk"[tiab] OR "Metabolic Syndrome"[Mesh] OR "Insulin Resistance"[Mesh] OR "Obesity"[Mesh] OR "Hypertension"[Mesh] OR "Dyslipidemias"[Mesh] OR "risk factor*"[tiab] OR "metabolic risk"[tiab] OR "insulin resistance"[tiab] OR dyslipidemia[tiab] OR obesity[tiab] OR overweight[tiab] OR "blood pressure"[tiab] OR hypertension[tiab] OR inflammation[tiab]) AND ("Cardiovascular Diseases"[Mesh] OR "cardiovascular disease*"[tiab] OR CVD[tiab] OR "heart disease*"[tiab] OR "cardiac disease*"[tiab] OR atherosclerosis[tiab] OR "coronary artery disease"[tiab] OR "myocardial infarction"[tiab] OR stroke[tiab] OR "ischemic heart disease"[tiab]) Filters applied: in the last 5 years, Randomized Controlled Trial, Systematic Review.
Embase	('endometriosis'/exp OR endometriosis:ti, ab) OR ('polycystic ovary syndrome'/exp OR 'stein leventhal syndrome'/exp OR PCOS:ti, ab OR 'polycystic ovary syndrome':ti, ab OR 'ovarian dysfunction':ti, ab) AND ('risk factor'/exp OR 'cardiovascular risk':ti, ab OR 'metabolic syndrome'/exp OR 'insulin resistance'/exp OR 'obesity'/exp OR 'hypertension'/exp OR 'dyslipidemia'/exp OR 'blood pressure':ti, ab OR inflammation:ti, ab) AND ('cardiovascular disease'/exp OR 'heart disease':ti, ab OR 'cardiac disease':ti, ab OR atherosclerosis:ti, ab OR 'coronary artery disease':ti, ab OR 'myocardial infarction':ti, ab OR stroke:ti, ab OR 'ischemic heart disease':ti, ab OR CVD:ti, ab)
Cochrane	([mh "Endometriosis"] OR endometriosis:ti, ab) OR ([mh "Polycystic Ovary Syndrome"] OR [mh "Stein-Leventhal Syndrome"] OR "ovarian dysfunction":ti, ab OR PCOS:ti, ab OR "polycystic ovary syndrome":ti, ab) AND ([mh "Risk Factors"] OR "cardiovascular risk":ti, ab OR [mh "Metabolic Syndrome"] OR [mh "Insulin Resistance"] OR [mh "Obesity"] OR [mh "Hypertension"] OR [mh "Dyslipidemias"] OR "risk factor*":ti, ab OR "metabolic risk":ti, ab OR "insulin resistance":ti, ab OR dyslipidemia:ti, ab OR obesity:ti, ab OR overweight:ti, ab OR "blood pressure":ti, ab OR hypertension:ti, ab OR inflammation:ti, ab) AND ([mh "Cardiovascular Diseases"] OR "cardiovascular disease*":ti, ab OR CVD:ti, ab OR "heart disease*":ti, ab OR "cardiac disease*":ti, ab OR atherosclerosis:ti, ab OR "coronary artery disease":ti, ab OR "myocardial infarction":ti, ab OR stroke:ti, ab OR "ischemic heart disease":ti, ab)
BVS	(TW: "Endometriose" OR "Endometriosis" OR "Endometriosis") OR ("Síndrome dos Ovários Policísticos" OR "Polycystic Ovary Syndrome" OR "Síndrome de Ovario Poliquístico" OR SOP OR PCOS OR "Síndrome de Stein-Leventhal" OR "Stein-Leventhal Syndrome" OR "Síndrome de Stein-Leventhal" OR "Disfunção Ovariana" OR "Ovarian Dysfunction" OR "Disfunción Ovárica") AND (TW: "Fatores de Risco" OR "Risk Factors" OR "Factores de Riesgo" OR "Risco Cardiovascular" OR "Cardiovascular Risk" OR "Riesgo Cardiovascular" OR "Síndrome Metabólica" OR "Metabolic Syndrome" OR "Síndrome Metabólica" OR "Resistência à Insulina" OR "Insulin Resistance" OR "Resistencia a la Insulina" OR "Dislipidemia" OR "Dyslipidemia" OR "Dislipidemia" OR "Obesidade" OR "Obesity" OR "Obesidad" OR "Sobrepeso" OR "Overweight" OR "Sobrepeso" OR "Pressão Arterial" OR "Blood Pressure" OR "Presión Arterial" OR "Hipertensão" OR "Hypertension" OR "Hipertensión" OR "Intolerância à Glicose" OR "Glucose Intolerance" OR "Intolerancia a la Glucosa" OR "Hiperglicemia" OR "Hyperglycemia" OR "Hiperglucemia" OR "Inflamação" OR "Inflammation" OR "Inflamación") AND (TW: "Doenças Cardiovasculares" OR "Cardiovascular Diseases" OR "Enfermedades Cardiovasculares" OR "Doença Isquêmica do Coração" OR "Ischemic Heart Disease" OR "Enfermedad Isquémica del Corazón" OR "Doença Arterial Coronariana" OR "Coronary Artery Disease" OR "Enfermedad de las Arterias Coronarias" OR "Aterosclerose" OR "Atherosclerosis" OR "Aterosclerosis" OR "Infarto do Miocárdio" OR "Myocardial Infarction" OR "Infarto de Miocardio" OR "Acidente Vascular Cerebral" OR "Stroke" OR "Accidente Cerebrovascular")

## List of Abbreviations and Acronyms

–ACEI: angiotensin-converting-enzyme inhibitors
–AMI: acute myocardial infarction
–ARB: angiotensin receptor blocker
–BMI: body mass index
–BP: blood pressure
–CAC: coronary artery calcium score
–CBG: cortisol-binding-globulin
–CHIP: clonal hematopoiesis of indeterminate potential
–CKD: chronic kidney disease
–COC: combined oral contraceptive
–CRP: C-reactive protein
–CVD: cardiovascular disease
–CVR: cardiovascular risk
–CVRF: cardiovascular risk factor
–DALYs: Disability-Adjusted Life Years
–DCM/SBC: Womens’ Cardiology Department of the Brazilian Society of Cardiology
–DKD: diabetic kidney disease
–DM: diabetes *mellitus*
–eNOS: endothelial nitric oxide synthase
–ESC: European Society of Cardiology
–FAI: perivascular fat attenuation index
–FEBRASGO: Brazilian Federation of the Societies of Gynecology and Obstetrics
–FIB-4: Fibrosis-4
–FSH: follicle-stimulating hormone
–GD: gestational diabetes
–GIP: glucose-dependent insulinotropic polypeptide
–GLP-1: glucagon-like peptide-1
–GnRH: gonadotrophin-releasing hormone
–GWG: gestational weight gain
–HbA1c: glycated hemoglobin
–HDL-c: high-density lipoprotein cholesterol
–HF: heart failure
–HFpEF: heart failure with preserved ejection fraction
–HOMA-IR: homeostasis model assessment of insulin resistance
–IHD: ischemic heart disease
–IL: interleukin
–INOCA: ischemia with no obstructive coronary arteries
–IP-10: interferon-gamma-induced protein 10
–IR: insulin resistance
–LDL-c: low-density lipoprotein cholesterol
–LH: luteinizing hormone
–LNG-IUS: levonorgestrel intrauterine system
–Lp(a): lipoprotein a
–MASH: metabolic dysfunction-associated steatohepatitis
–MASLD: metabolic dysfunction-associated steatotic liver disease
–MCP-1: monocyte chemoattractant protein 1
–MHT: menopausal hormone therapy
–MI: myocardial infarction
–MINOCA: myocardial infarction with no obstructive coronary arteries
–MS: metabolic syndrome
–NF-κB: nuclear factor kappa B
–NO: nitric oxide
–OGTT: oral glucose tolerance test
–POS: polycystic ovary syndrome
–RF: risk factor
–ROS: reactive oxygen species
–RR: relative risk
–SAH: systemic arterial hypertension
–SBEM: Brazilian Society of Endocrinology and Metabolism Study
–sEng: soluble endoglin
–sFlt-1: soluble Fms-like tyrosine kinase-1
–SGLT2: sodium-glucose cotransporter type 2
–SHBG: sexual-hormone-binding-globulin
–T1DM: type 1 diabetes *mellitus*
–T2DM: type 2 diabetes *mellitus*
–TNF-α: tumor necrosis factor alpha
–us-CRP: ultrasensitive C-reactive protein
–VCAM-1: vascular cell adhesion molecule
–VEGF: vascular endothelial growth factor
–VLDL-c: very-low-density lipoprotein cholesterol
–VMS: vasomotor symptoms
–VTE: venous thromboembolism
–WHI: Women's Health Initiative
–WHR: waist-to-hip ratio
–WHtR: waist-to-height ratio
